# Supplementary material for: A high-quality bonobo genome refines the analysis of hominid evolution
Source: Nature. 2021 May 5;594(7861):77–81. doi: 10.1038/s41586-021-03519-x (PMC8172381; doi:10.1038/s41586-021-03519-x)
Supplement: Supplementary file 1 — The Supplementary Information file includes: Supplementary Figures 1-11, Supplementary Discussion, and Supplementary References. [file 41586_2021_3519_MOESM1_ESM.pdf]

nature portfolio

https://doi.org/10.1038/s41586-021-03519-x

Supplementary information

A high-quality bonobo genome refines the analysis of hominid evolution

In the format provided by the authors and unedited

1. SUPPLEMENTARY FIGURES

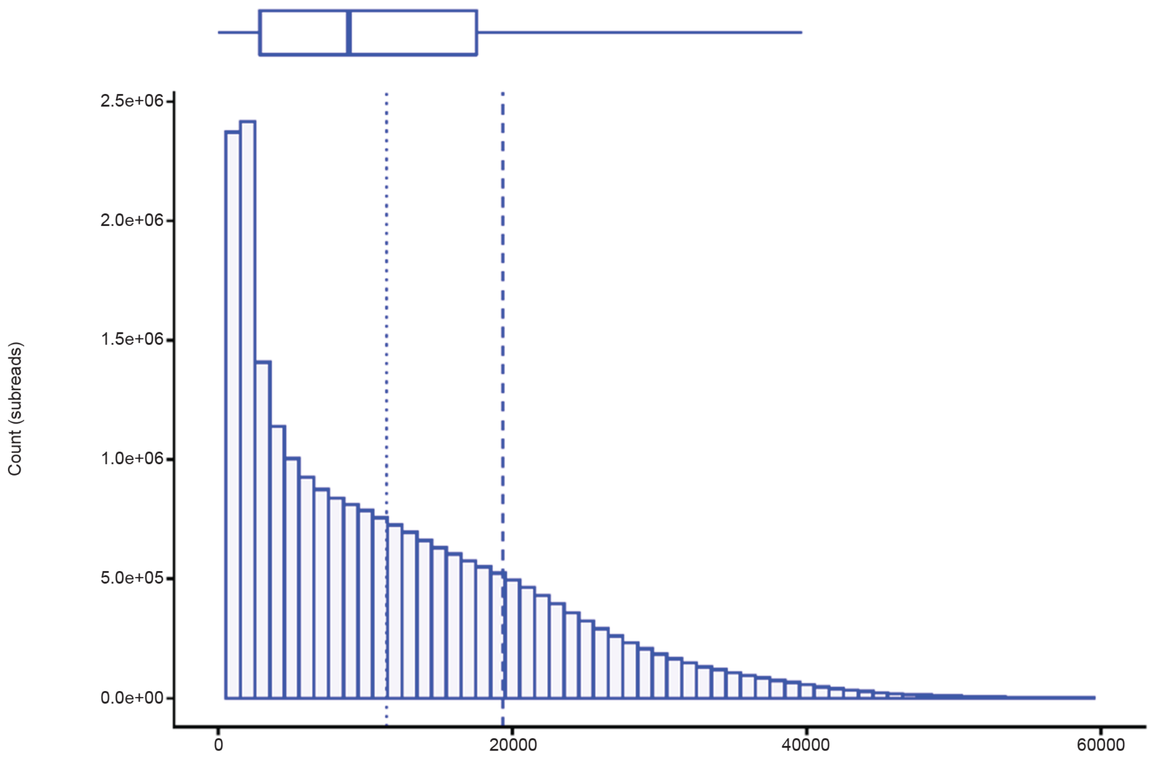

Supplementary Figure 1. Distribution of subread lengths for bonobo sequencing data. Marginal boxplot indicates quartiles with an average subread length of 11.4 kbp (vertical dotted) and an N50 subread length of 19.3 kbp (vertical dashed). The box shows the first quartile to the third quartile. A vertical line within the box shows the median. The whisker represents range. The boxplot was generated using the R package ggplot2 function geom_boxplot.

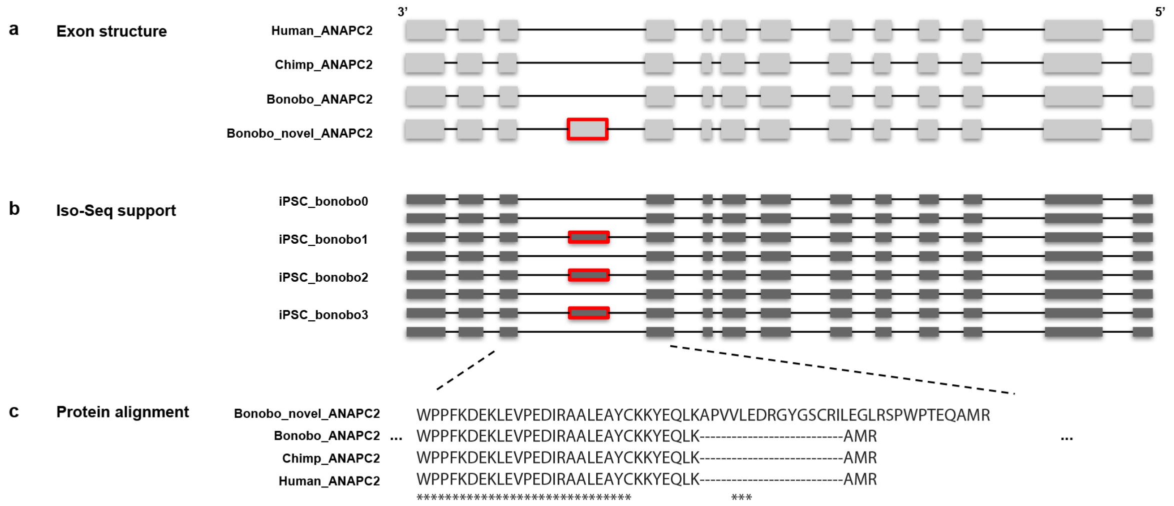

Supplementary Figure 2. A bonobo isoform of *ANAPC2* contains a novel exon predicted by the AugustusPB mode of CAT, which is supported by bonobo Iso-Seq data from iPSC tissue. The exon is not seen in the human or chimpanzee annotations. **a,** The exon structure of this gene is shown for human, chimpanzee, and bonobo. The novel exon is alternatively spliced, seen in one isoform of *ANAPC2* in bonobo and not the other. **b,** A sample of Iso-Seq reads from the bonobo iPSC tissue shows support for alternative splicing in *ANAPC2*. **c,** A protein alignment of the gene is shown for human, chimpanzee, and bonobo.

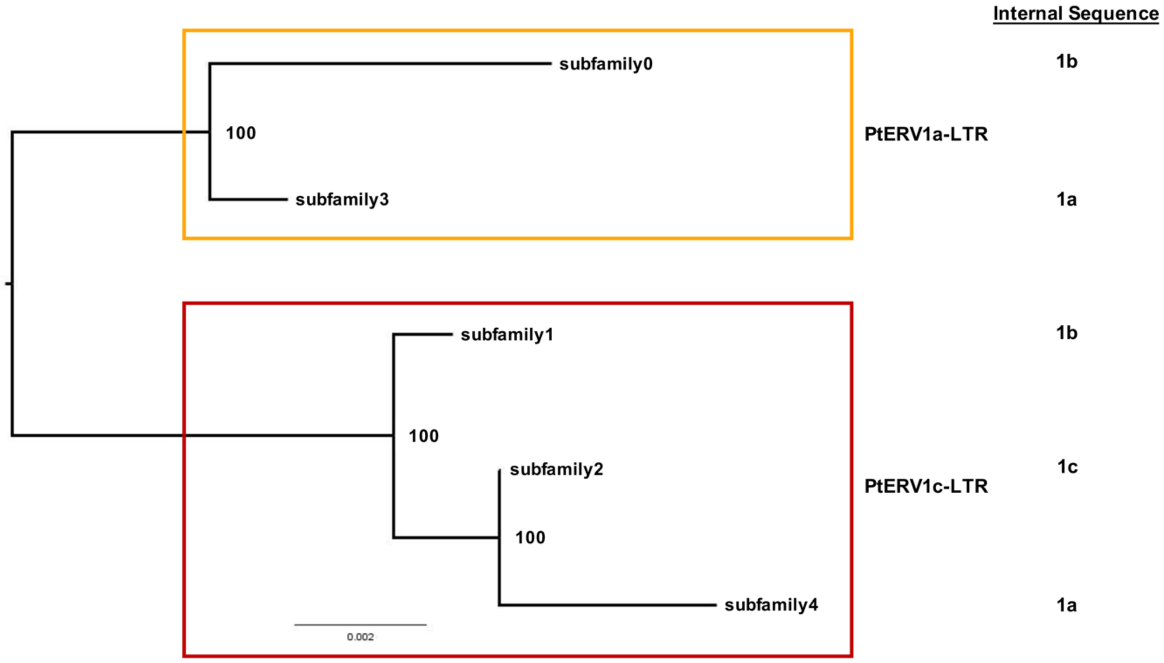

Supplementary Figure 3. PtERV analysis in the bonobo genome. The neighbor-joining tree of PtERV subfamilies is rooted by the midpoint. The red box indicates subfamilies grouped together by the presence of a PtERV1c 5' and 3' flanking LTR, while the orange box indicates subfamilies flanked by PtERV1a LTRs. The internal sequence for each subfamily is indicated on the right-hand side of the tree. The number at each node indicates bootstrap support value from 1000 replicates.

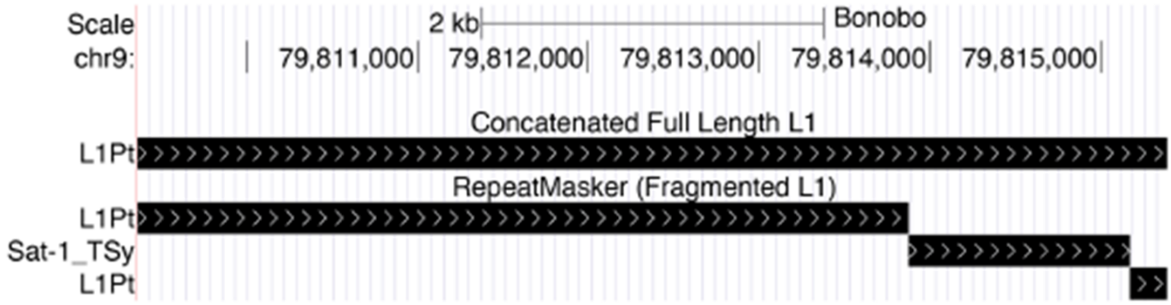

Supplementary Figure 4. Artifactual annotation of Sat-1_Tsy elements. RepeatMasker correctly annotates subparts of full-length L1s but incorrectly annotates the middle of these elements as a tarsier-specific satellite element (Sat-1_Tsy), preventing subsequent joining of the subparts (Fragmented L1 track). By joining Sat-1_Tsy elements with L1 subparts and using the 3' UTR annotation to classify full-length elements (Concatenated Full Length L1 track), we produced full-length L1s consistent with annotations in other great apes.

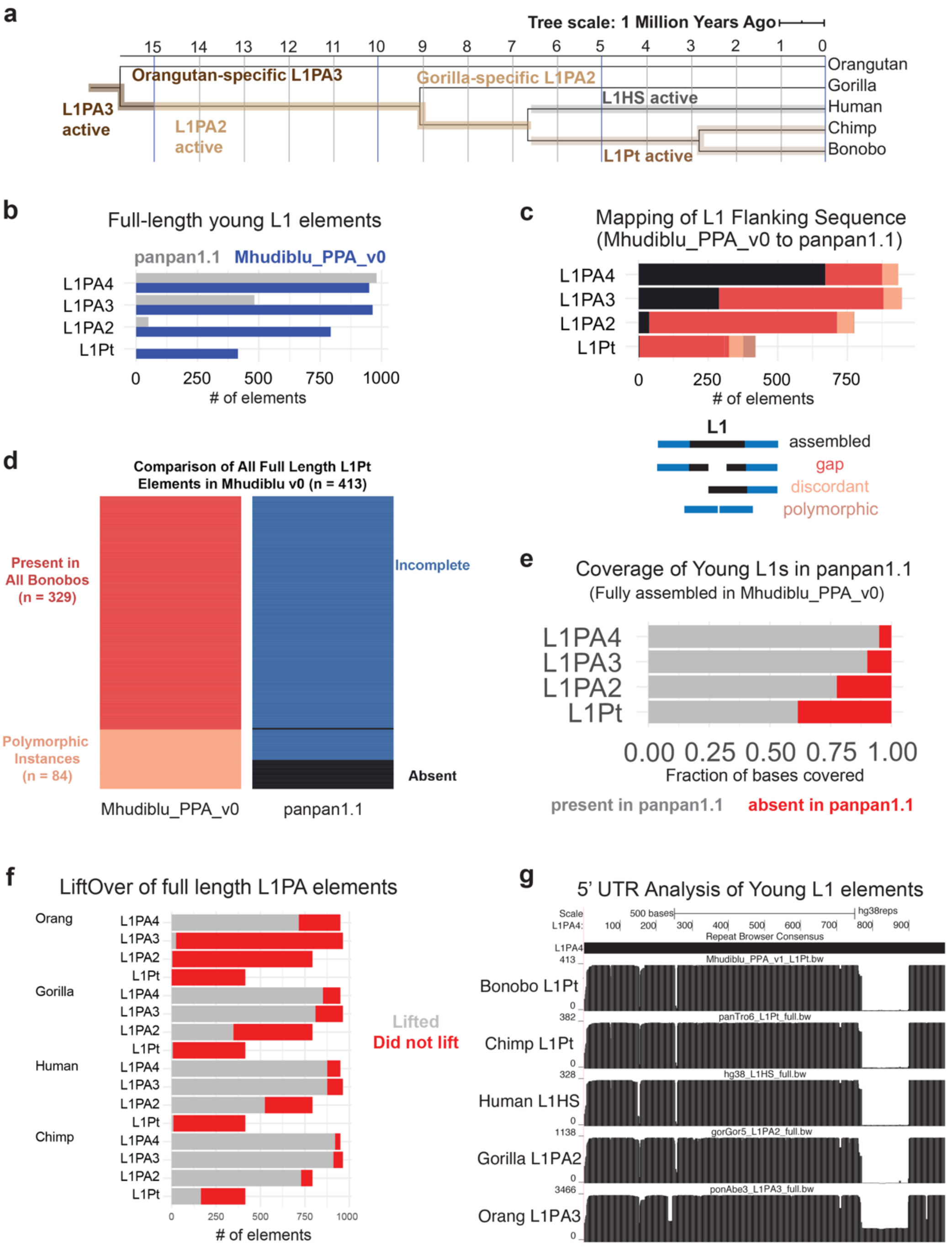

Supplementary Figure 5. L1 elements in Mhudiblu_PPA_v0. a, Phylogeny of recently active L1s in great apes: L1PA3 (active in great ape ancestor and immediately after orangutan divergence), L1PA2 (active in human/chimpanzee/bonobo/gorilla ancestor), L1HS (active in human), and L1Pt (active in *Pan* lineage). **b,** Counts of full-length (>6,000 nt) L1PA elements identified in RepeatMasker annotations of each assembly. **c,** Full-length L1 repeats are more complete in Mhudiblu_PPA_v0 compared to panpan1.1. Sequence flanking the L1 insert can either map concordantly between the two assemblies (~6,000 nt apart (black)), concordantly but with an internal gap in panpan1.1 (red), discordantly (pink), or adjacently (brown). Younger families (L1Pt) show greater disparity and are more likely to be completely represented in Mhudiblu_PPA_v0. **d,** Comparison and genotyping of L1Pt elements identified in the Mhudiblu_v0 assembly (n = 413). We identified 413 L1Pt elements in the assembly; genotyping with data from 10 other bonobo individuals showed that 329 of the insertions were fixed for insertion presence in the population while 84 elements displayed insertion polymorphism in these 10 bonobo samples. Almost all (327) of the fixed insertions had incomplete but identifiable syntenic fragments in the panpan1.1 genome. Of the polymorphic elements, fragmented

versions of 43 elements were also found in the panpan1.1 genome while 41 elements were absent (black). **e,** Fraction of total bases in full-length L1PA elements present in both Mhudiblu_PPA_v0 and panpan1.1 (gray) and those present only in Mhudiblu_PPA_v0. **f,** liftOver of L1PA elements to other great apes displays the expected evolutionary patterns with elements present in the great ape common ancestor (L1PA) mostly lifting (gray) across all species and elements specific to the pan lineage absent (red) in all species except chimpanzee and bonobo. **g,** Mapping of the 5' UTRs of the youngest L1PA families in each primate to the ancestral L1PA4 consensus demonstrates that bonobo L1Pt elements have evolved consistently with other active primate L1s.

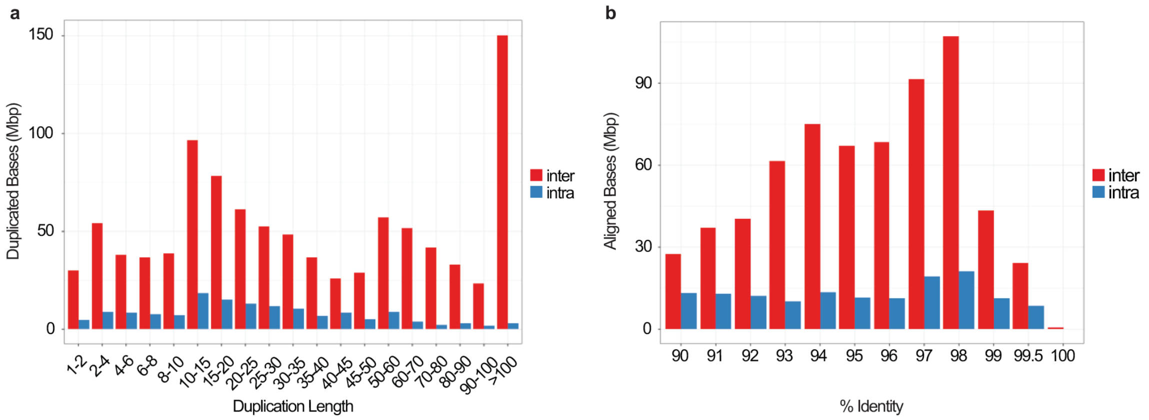

Supplementary Figure 6. Distribution of SDs in bonobo assembly. The length (**a**) and sequence identity (**b**) distribution of interchromosomal (red) and intrachromosomal (blue) SDs (>1 kbp in length and >90% identical) are shown for Mhudiblu_PPA_v0.

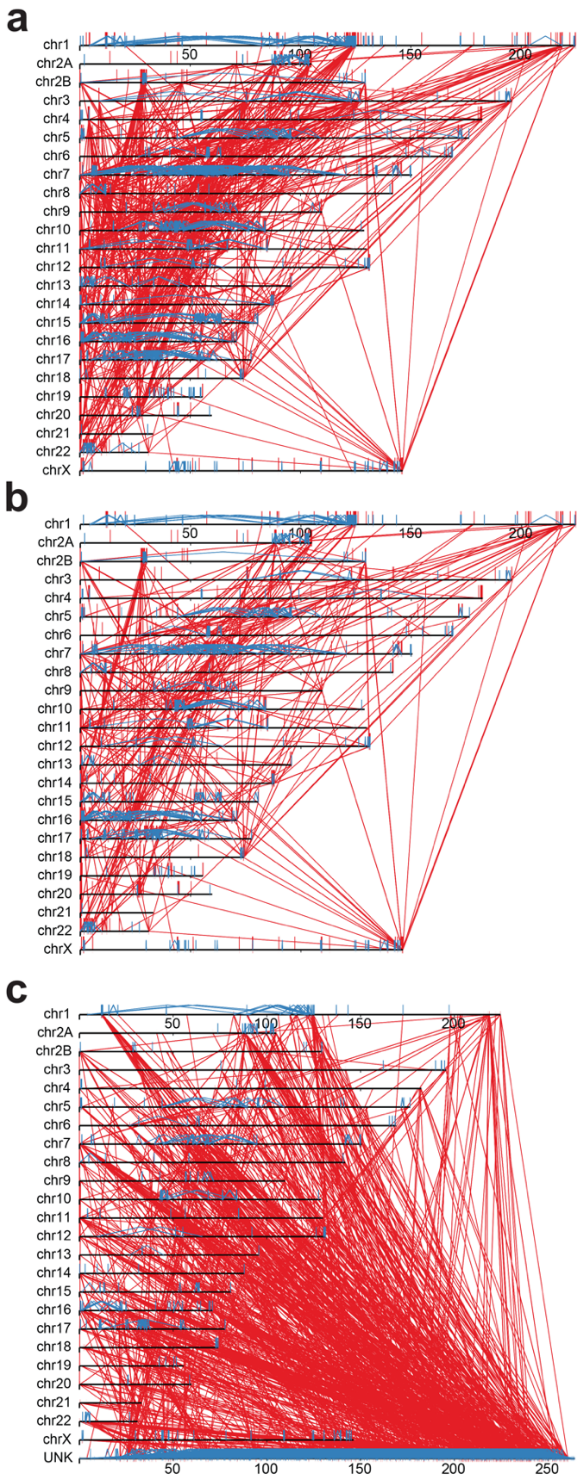

Supplementary Figure 7. Pattern of SDs in bonobo assembly. Interchromosomal (red) and intrachromosomal (blue) distribution patterns of SD pairwise alignments are depicted for **a,** SDs ≥10 kbp and 90% identical, **b,** SDs ≥10 kbp and 95% identical, and **c,** pattern of the largest and most identical (≥10 kbp and ≥98%) intrachromosomal (blue) and interchromosomal (red) SDs in the bonobo genome including unplaced contigs (designed here UNK or the "unknown chromosome"). The 4,271 unplaced contigs are organized from largest to smallest (left to right) as though they constituted a single chromosome. Chromosomal assignments are based on ape phylogenetic nomenclature.

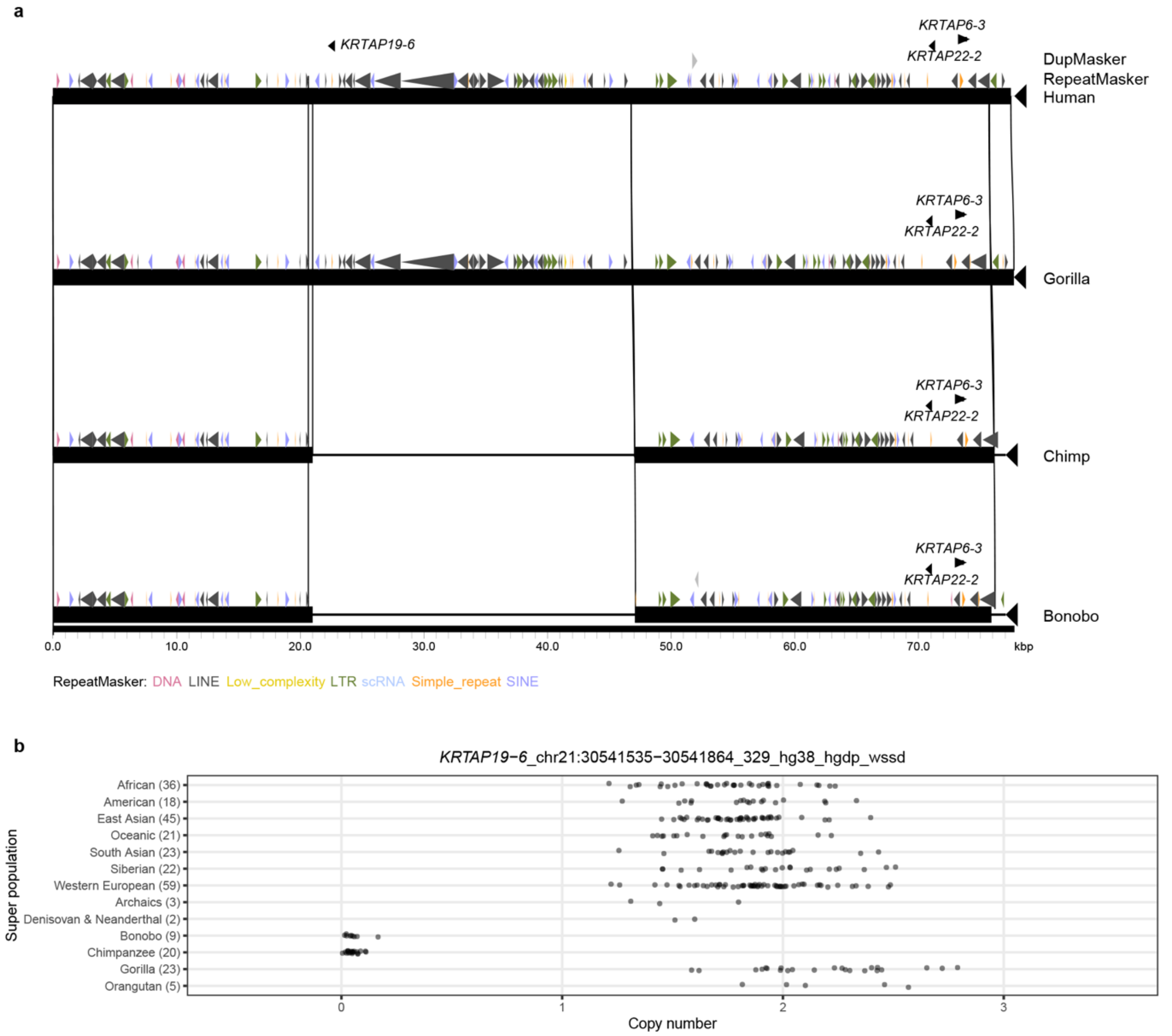

Supplementary Figure 8. Loss of keratin-associated gene in chimpanzee and bonobo lineages. a, A 25.7 kbp deletion results in the complete loss of the hair keratin-associated protein (KRTAP19-6) in bonobo and chimpanzee. **b,** Sequence read-depth genotyping of deletion in human and ape Illumina WGS data (number of samples) confirms a *Pan*-specific loss fixed in both bonobo and chimpanzee.

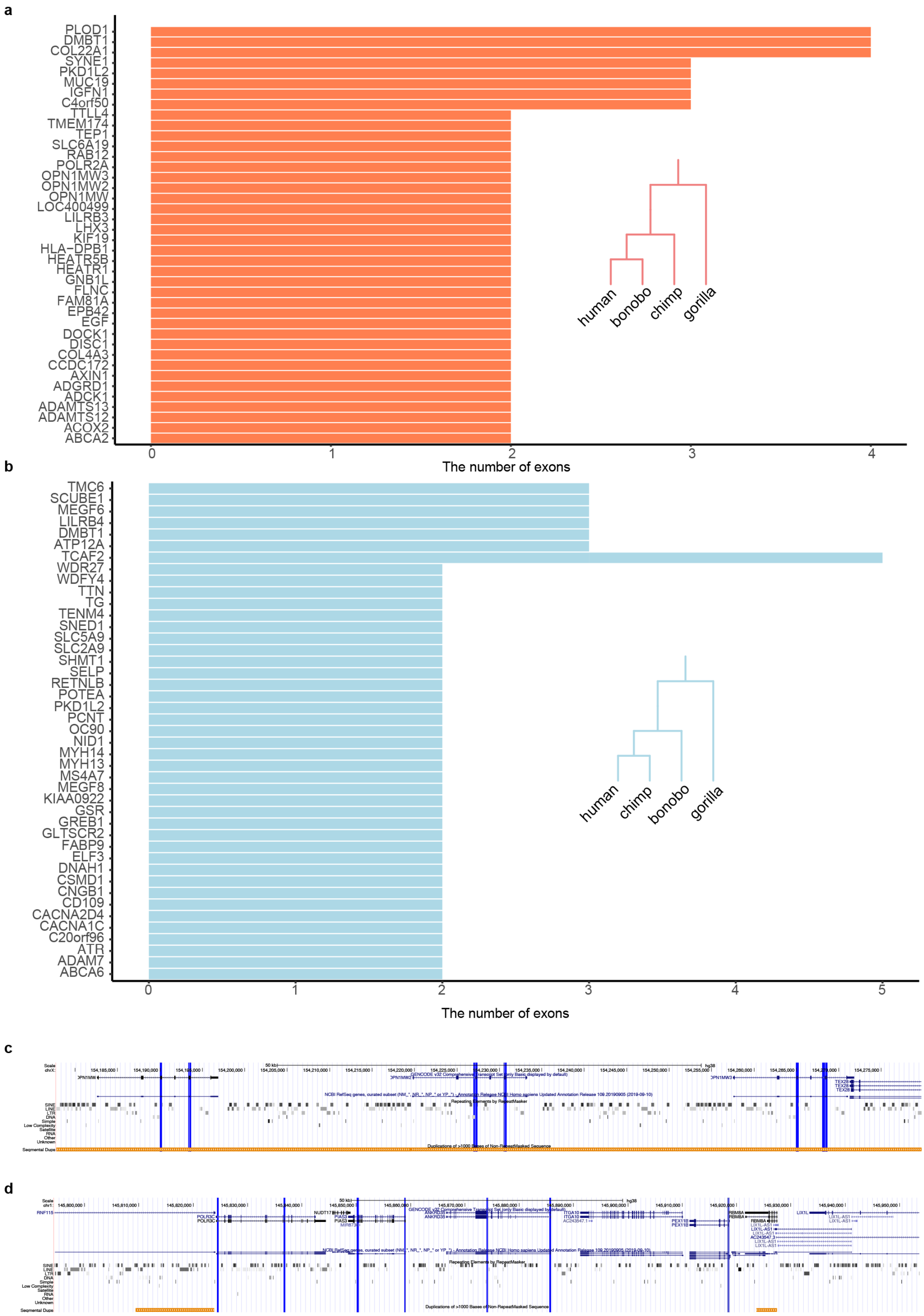

Supplementary Figure 9. Clustered ILS signals over genes. a, Multiple exons under human–bonobo ILS (40 genes). **b,** Multiple exons under human–chimpanzee ILS (44 genes). **c,** An example of a cluster of multiple genes showing human–bonobo ILS. **d,** An example of a cluster of multiple genes under human–chimpanzee ILS.

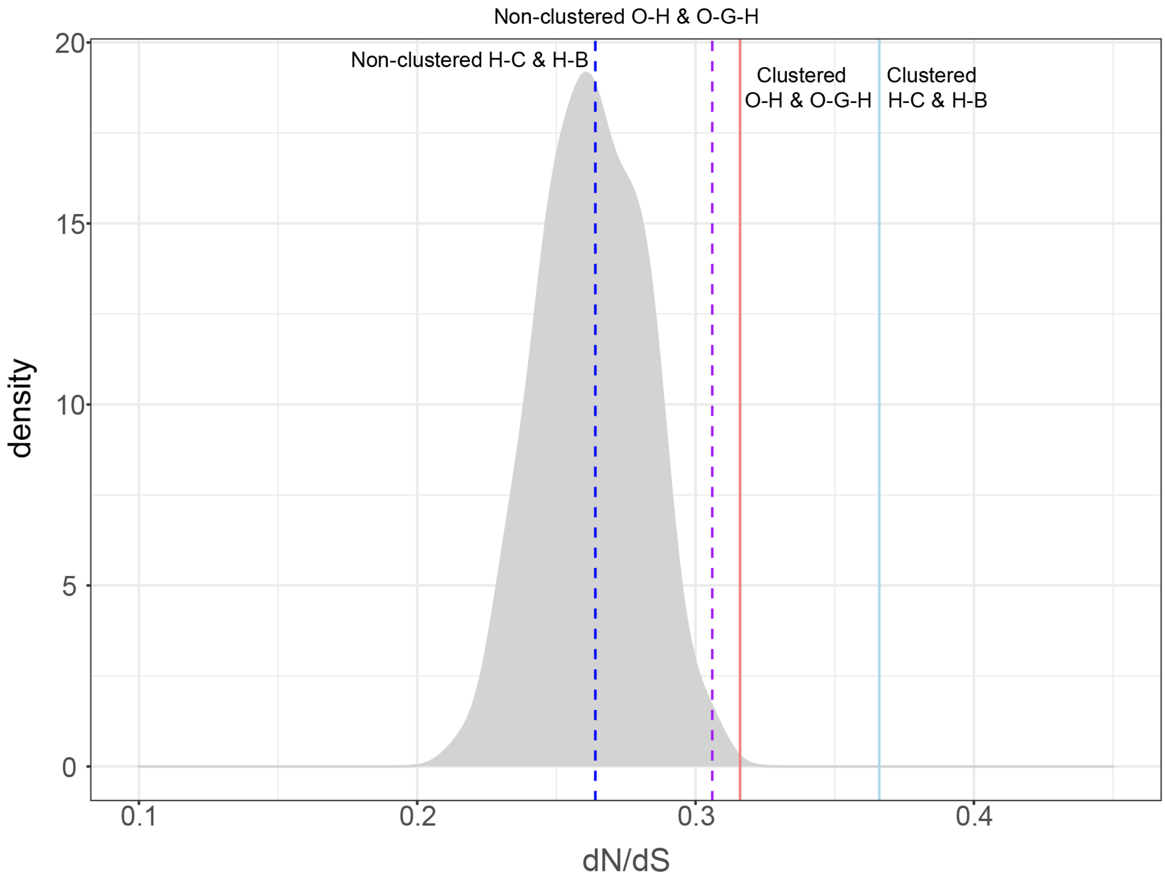

Supplementary Figure 10. Elevated dN/dS in clustered sites of ILS. The null distribution (gray) is based on calculation of mean dN/dS for 1000 genes drawn randomly from the genome (100 simulations; mean: 0.263). The blue solid and dashed lines represent the mean dN/dS for clustered H-C & H-B ILS (mean: 0.366, p< 2.2e-16) and non-clustered H-C & H-B sites (mean=0.264, p=0.45), respectively. The solid orange and dashed purple lines represent mean dN/dS of the clustered O-H & O-G-H ILS (mean=0.316, p< 2.2e-16) and the non-clustered O-H & O-G-H ILS (mean=0.306, p< 2.2e-16). Significance performed using the t test in R and confirmed by simulation of gene dN/dS values.

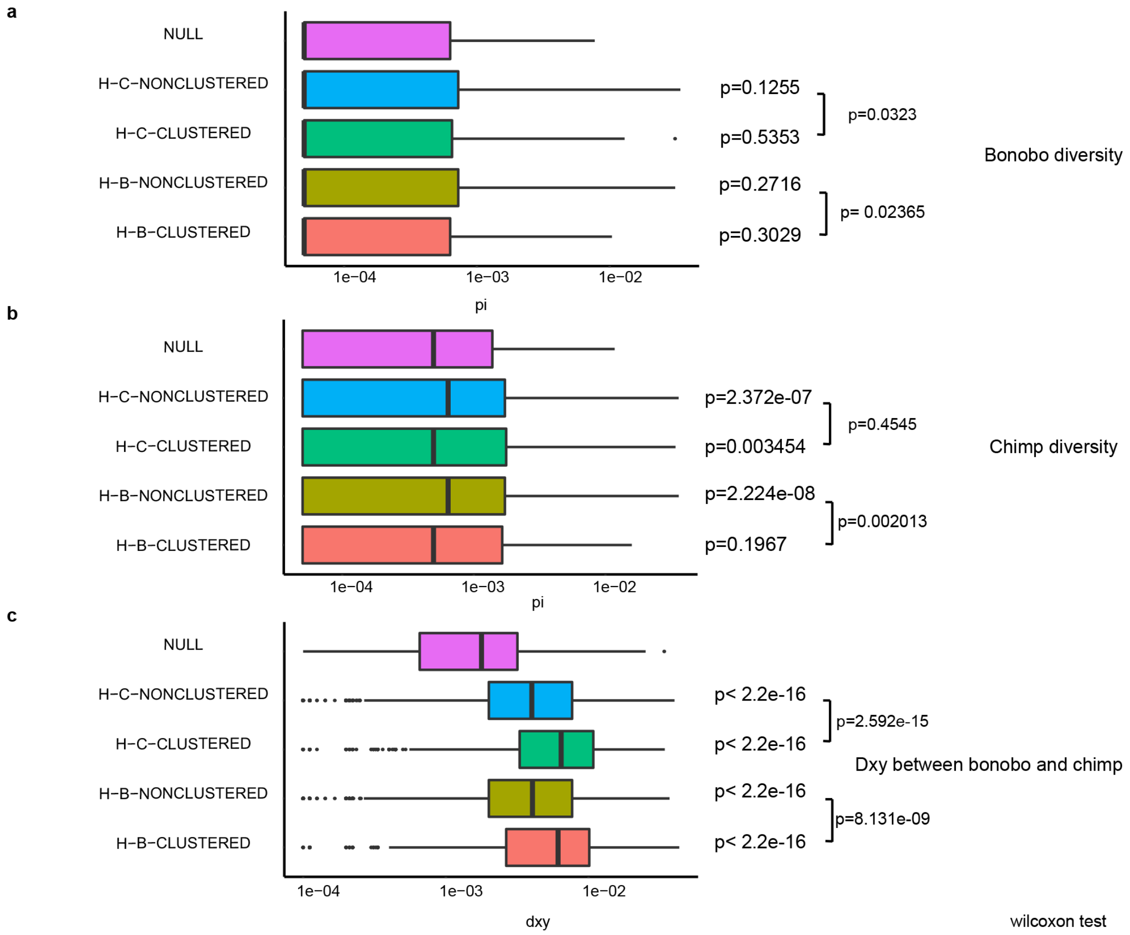

Supplementary Figure 11. Tests for balancing selection and ILS. Genetic diversity (pi,dxy) is compared for clustered and non-clustered ILS segments and a genome-wide null for **a,** bonobo, **b,** chimpanzee, and **c,** between the species (dxy The schematic shows the mean with 95% confidence intervals with boxplot (log scale x-axis); p-value between ILS segments and the to the right of each boxplot (Wilcoxon rank test); and the p-value between clustered and non-clustered paired bracket (Wilcoxon rank test). The NULL was constructed based on 3,000 randomly sampled 500 bp segments from a total 2,443,769 aligned segments.

2. SUPPLEMENTARY DISCUSSION

High-quality hominid genomes are a critical resource for understanding the genetic differences that make us human as well as the diversification of the *Pan* lineage over the last two million years of evolution. Using multiple genomic technologies, we successfully constructed a high-quality bonobo genome without guidance from human or other great ape genomes. Compared to the previous bonobo reference genome, 99% of the gaps are closed, most gene models are complete, the majority of full-length MEIs are identified, and we have improved by >6-fold our understanding of bonobo SD content. Among those SDs that remain collapsed in the assembly, we have successfully sequence resolved ~65% of them, annotating some of the most recently duplicated bonobo genes for the first time. As a result, we present the most complete set of functional changes in the bonobo lineage, including exon and gene losses, structural rearrangements like inversions, and gene family expansions.

Notably, the bonobo represents the last of the great ape genomes to be sequenced using long-read sequencing technology. Its sequence will facilitate more systematic genetic comparisons between human, chimpanzee, gorilla, and orangutan without the limitations of technological differences in sequencing and assembly of the original reference[1-5]. As an example, bonobo sequencing and comparison to other ape genomes reclassifies 23 deletions and 21 insertions as no longer human-specific, including five that are predicted to be near genes (**Supplementary Data**). More importantly, the improved references allow for a more complete investigation of genomic regions under positive and balancing selection in the *Pan* lineage. In the case of major histocompatibility complex, we effectively close 289 of the 291 gaps across this region and, while we still detect strong signatures of balancing selection, we no long identify potential selective sweeps in the top 1% of regions as reported previously[2] (**Extended Data Fig. 8 and Supplementary Data Fig. 42**).

Comparisons among long-read assembled ape genomes allowed us to systematically classify all gene family expansions and contractions that have occurred on the *Pan* lineage since divergence from other apes. While relatively few in number, members of eukaryotic initiation factors (*EIF*) gene have been targets of recent and independent duplication events. For example, two loci (*EIF4A3* and *EIF3C*) have been specifically targeted for expansion in the common ancestor of chimpanzee and bonobo with independent expansions continuing to occur since the speciation of the two lineages (**Fig. 2 and Extended Data Fig. 5**). Our analysis shows that these novel duplicate genes produce full-length transcripts that maintain an open reading frame with a relatively small number of amino acid differences between human and chimpanzee orthologs (**Fig. 2 and Extended Data Fig. 5**). While the function of these additional genes remains to be investigated in the chimpanzee lineage, EIF4A3 proteins encode DEAD box RNA helicases implicated in regulation of splicing while EIF3C proteins encode subunits of a multimeric complex associated with translation initiation and start codon recognition[6]. Moreover, *EIF4A3* in has been associated with intellectual disability and Richieri-Costa-Pereira syndrome when overexpressed in human and it is critically important in developing the cerebral cortex when underexpressed in mouse.

Comparing the chimpanzee and bonobo genomes, we identify 148 fixed insertions/deletions that potentially affect gene expression and five deletions that have the potential to disrupt or alter the protein-coding sequence. While few in number, some of the latter suggest fundamental genetic differences between bonobo and chimpanzee. For example, we identified a 147 bp deletion that is predicted to result in a 49 amino acid loss corresponding to exon 2 of *ADAR1*—an RNA-specific adenosine deaminase important for adenosine-to-inosine editing of viruses and host molecules. Based on human RefSeq annotation, the location of the deletion may also drive preferential usage of a different *ADAR* isoform. Iso-Seq analysis of bonobo, however, (**Supplementary Data Fig. S26**) confirms a full-length cDNA with the 49 amino acid deletion occurring near the nuclear export signal domain and between two Z-DNA binding domains of *ADAR1* (**Supplementary Data Fig. S26**). While the effect of the deletion on transport, DNA binding, or RNA editing ability awaits experimentation, it is intriguing that previous comparative studies have suggested positive selection of *ADAR1* in bonobo when compared to other mammalian lineages[7]. Such a change may be related to the different levels of serotonergic innervation observed in bonobo versus chimpanzee neural circuits[8]. The gene itself has been implicated in a variety of biological activities ranging from recoding neurotransmitter function to suppression of innate immunity[9].

Another interesting example of bonobo–chimpanzee differences is the fixed large deletion (41.5 kbp) that completely ablates the gene *SAMD9* (sterile alpha motif domain containing 9), specifically in the bonobo lineage (**Extended Data Fig. 6).** In humans, *SAMD9* is recognized as a cell proliferation gene and its loss is associated with a variety of hematological malignancies[10] as well as life-threatening normophosphatemic familiar tumoral calcinosis disease[11]. Remarkably, both it and its ancient paralog *SAMD9L* have been subject to positive selection during mammalian evolution but only in the house mouse has the loss of *SAMD9* been reported as unique to rodents[12] suggesting not only pleiotropic adaptive evolution but recurrent and independent deletion of this gene in both lineages. Notably, both *SAMD9* and *SAMD9L* have been reported as restriction factors for poxviruses, and the resistance to this infection is differential depending on the presence of both or only one of these two paralogues. The difference between human and mouse has been posited to be the result of species differences in response to host-pathogen interactions[13].

Similarly, a 24.3 kbp deletion completely removes the gene *LYPD8* (LY6/PLAUR domain containing 8) in bonobo. Studies in human and mouse indicate that *LYPD8* is important in maintaining gut homeostasis by promoting the segregation of flagellated microbiota and colonic epithelia[14,15] and, thereby, preventing colonization of pathogenic bacteria in the colonic epithelia[16]. Its complete loss in humans has been predicted to contribute to disease and disease susceptibility[17,18]. The complete loss of *LYPD8* in the bonobo lineage, however, may suggest differences in the molecular mechanisms of gut homeostasis maintenance, especially as it relates to changes in diet and gut microbiota diversity[19]. Although bonobo and chimpanzee share omnivorous and frugivorous diets, some studies have shown food preferences in wild bonobos due to the ecological habitat changes arising during their speciation from chimpanzee[20]. Alternatively, it is tempting to speculate that some of the gene family expansions (e.g., defensins) that have occurred specifically since bonobo diverged from chimpanzee may have compensated for the loss of these and other

genes possibly in response to evolution of the innate immune system and differences in infectious disease exposures[21].

The availability of other high-quality African ape genomes allowed us to revisit ILS at a fine-scale level of resolution because ~75% of the genome could now be systematically compared phylogenetically. Because there is limited evidence of introgression between human and bonobo/chimpanzee ancestral populations after the split between bonobo and chimpanzee[22], we regarded all inconsistent tree topologies as ILS for simplicity[2,23]. We predict that a significantly greater fraction (~5.1%) of the human genome is closer to chimpanzee/bonobo when compared to previous studies (3.3%)[2]. We estimate that >36.5% of the hominid genome shows ILS if we consider a deeper phylogeny including gorilla and orangutan due, in part, to the large effective population size of the common ancestor of hominids (**Supplementary Data**).

Like the previous analysis, we found ILS depleted for genes and enriched for intergenic and repetitive regions of the genome. However, unlike previous studies, we show that the regions of the genome subject to ILS are not randomly distributed. We specifically show that 26% of the ILS regions are clustered and that exons underlying these clustered ILS signals show elevated rates of amino acid replacement. These findings support a previous study in gorilla that showed a subtler correlation where genes with higher dN/dS values are enriched in ILS segments[3]. In that study, however, they explained the observation as a result of stronger purifying selection in non-ILS sites or background selection reducing the effective population size and as a result a depletion of ILS (**Supplementary Data**). Our genome-wide exon analyses specifically show that only a subset of clustered ILS exons are driving this effect and that these genes are enriched for glycoprotein and EGF-lke calcium signaling functions due to the action of either relaxed or positive selection for genes in these pathways.

References

1 Kronenberg, Z. N. *et al.* High-resolution comparative analysis of great ape genomes. *Science* **360**, doi:10.1126/science.aar6343 (2018).

2 Prufer, K. *et al.* The bonobo genome compared with the chimpanzee and human genomes. *Nature* **486**, 527-531, doi:10.1038/nature11128 (2012).

3 Scally, A. *et al.* Insights into hominid evolution from the gorilla genome sequence. *Nature* **483**, 169-175, doi:10.1038/nature10842 (2012).

4 Consortium, T. C. S. a. A. Initial sequence of the chimpanzee genome and comparison with the human genome. *Nature* **437**, 69-87, doi:10.1038/nature04072 (2005).

5 Locke, D. P. *et al.* Comparative and demographic analysis of orang-utan genomes. *Nature* **469**, 529-533, doi:10.1038/nature09687 (2011).

6 Wolf, D. A., Lin, Y., Duan, H. & Cheng, Y. eIF-Three to Tango: emerging functions of translation initiation factor eIF3 in protein synthesis and disease. *J Mol Cell Biol* **12**, 403-409, doi:10.1093/jmcb/mjaa018 (2020).

7 Forni, D. *et al.* Diverse selective regimes shape genetic diversity at ADAR genes and at their coding targets. *RNA Biol* **12**, 149-161, doi:10.1080/15476286.2015.1017215 (2015).

8 Stimpson, C. D. *et al.* Differential serotonergic innervation of the amygdala in bonobos and chimpanzees. *Soc Cogn Affect Neurosci* **11**, 413-422, doi:10.1093/scan/nsv128 (2016).

9 Heraud-Farlow, J. E. & Walkley, C. R. What do editors do? Understanding the physiological functions of A-to-I RNA editing by adenosine deaminase acting on RNAs. *Open Biol* **10**, 200085, doi:10.1098/rsob.200085 (2020).

10 Davidsson, J. *et al.* SAMD9 and SAMD9L in inherited predisposition to ataxia, pancytopenia, and myeloid malignancies. *Leukemia* **32**, 1106-1115, doi:10.1038/s41375-018-0074-4 (2018).

11 Hershkovitz, D. *et al.* Functional characterization of SAMD9, a protein deficient in normophosphatemic familial tumoral calcinosis. *J Invest Dermatol* **131**, 662-669, doi:10.1038/jid.2010.387 (2011).

12 Lemos de Matos, A., Liu, J., McFadden, G. & Esteves, P. J. Evolution and divergence of the mammalian SAMD9/SAMD9L gene family. *BMC Evol Biol* **13**, 121, doi:10.1186/1471-2148-13-121 (2013).

13 Meng, X. *et al.* A paralogous pair of mammalian host restriction factors form a critical host barrier against poxvirus infection. *PLoS Pathog* **14**, e1006884, doi:10.1371/journal.ppat.1006884 (2018).

14 Ara, T. *et al.* Intestinal goblet cells protect against GVHD after allogeneic stem cell transplantation via Lypd8. *Sci Transl Med* **12**, doi:10.1126/scitranslmed.aaw0720 (2020).

15 Okumura, R. & Takeda, K. Maintenance of gut homeostasis by the mucosal immune system. *Proc Jpn Acad Ser B Phys Biol Sci* **92**, 423-435, doi:10.2183/pjab.92.423 (2016).

16 Okumura, R. *et al.* Lypd8 inhibits attachment of pathogenic bacteria to colonic epithelia. *Mucosal Immunol* **13**, 75-85, doi:10.1038/s41385-019-0219-4 (2020).

17 Cryan, J. F. *et al.* The Microbiota-Gut-Brain Axis. *Physiol Rev* **99**, 1877-2013, doi:10.1152/physrev.00018.2018 (2019).

18 Foster, J. A. & McVey Neufeld, K. A. Gut-brain axis: how the microbiome influences anxiety and depression. *Trends Neurosci* **36**, 305-312, doi:10.1016/j.tins.2013.01.005 (2013).
19 Zmora, N., Suez, J. & Elinav, E. You are what you eat: diet, health and the gut microbiota. *Nat Rev Gastroenterol Hepatol* **16**, 35-56, doi:10.1038/s41575-018-0061-2 (2019).
20 Rafert, J. & E.O., V. *Bonobo Husbandry Manual*. Vol. Bonobo Nutrition – relation of captive diet to wild diet. 3.1-3.18 (1997).
21 Rausell, A. *et al.* Common homozygosity for predicted loss-of-function variants reveals both redundant and advantageous effects of dispensable human genes. *Proc Natl Acad Sci U S A* **117**, 13626-13636, doi:10.1073/pnas.1917993117 (2020).
22 Patterson, N., Richter, D. J., Gnerre, S., Lander, E. S. & Reich, D. Genetic evidence for complex speciation of humans and chimpanzees. *Nature* **441**, 1103-1108, doi:10.1038/nature04789 (2006).
23 de Manuel, M. *et al.* Chimpanzee genomic diversity reveals ancient admixture with bonobos. *Science* **354**, 477-481, doi:10.1126/science.aag2602 (2016).
