# Supplementary material for: A high-quality bonobo genome refines the analysis of hominid evolution
Source: Nature. 2021 May 5;594(7861):77–81. doi: 10.1038/s41586-021-03519-x (PMC8172381; doi:10.1038/s41586-021-03519-x)
Supplement: Supplementary file 2 — Reporting Summary [file 41586_2021_3519_MOESM2_ESM.pdf]

nature research

Corresponding author(s): Mario VENTURA & Evan E. EICHLER

Last updated by author(s): Mar 30, 2021

Reporting Summary

Nature Research wishes to improve the reproducibility of the work that we publish. This form provides structure for consistency and transparency in reporting. For further information on Nature Research policies, see our Editorial Policies and the Editorial Policy Checklist.

Statistics

For all statistical analyses, confirm that the following items are present in the figure legend, table legend, main text, or Methods section.

n/a	Confirmed	
☐	☒	The exact sample size (*n*) for each experimental group/condition, given as a discrete number and unit of measurement
☐	☒	A statement on whether measurements were taken from distinct samples or whether the same sample was measured repeatedly
☐	☒	The statistical test(s) used AND whether they are one- or two-sided<br>*Only common tests should be described solely by name; describe more complex techniques in the Methods section.*
☐	☒	A description of all covariates tested
☐	☒	A description of any assumptions or corrections, such as tests of normality and adjustment for multiple comparisons
☐	☒	A full description of the statistical parameters including central tendency (e.g. means) or other basic estimates (e.g. regression coefficient) AND variation (e.g. standard deviation) or associated estimates of uncertainty (e.g. confidence intervals)
☐	☒	For null hypothesis testing, the test statistic (e.g. *F*, *t*, *r*) with confidence intervals, effect sizes, degrees of freedom and *P* value noted<br>*Give P values as exact values whenever suitable.*
☐	☒	For Bayesian analysis, information on the choice of priors and Markov chain Monte Carlo settings
☐	☒	For hierarchical and complex designs, identification of the appropriate level for tests and full reporting of outcomes
☒	☐	Estimates of effect sizes (e.g. Cohen's *d*, Pearson's *r*), indicating how they were calculated

Our web collection on statistics for biologists contains articles on many of the points above.

Software and code

Policy information about availability of computer code

Data collection	Pacific Bioscience Sequel II Instrument Control SW (v7.1 or v8.0) and Leica Application Suite X (v3.7).
Data analysis	We applied Falcon (git id 53444482, dgordon branch available on 2017.06.13) to assemble the bonobo genome from SMRT sequence reads. The assembly was error-corrected using Quiver (version 0.7.6) and then further error-corrected using Pilon (version 1.21). The contigs were placed into scaffolds using the HybridScaffolds suite (pipeline version 4573) from the BioNano Genomics. Access software (pipeline version 4573, and RefAligner version 7376). To assign each contig/scaffold into unique groups corresponding to individual chromosomal homologues we used SaaRclust (github daewoooo branch available on Mar 3, 2019, version 0.99). We aligned available Strand-seq data to the Mhudiblu assembly (v0) using the BWA aligner (version 0.7.17-r1188) with default parameters for paired-end mapping. Subsequently, we used sambamba (version 0.6.8) in order to mark duplicated reads and SAMtools (version 1.9) to sort and index the final BAM file for each Strand-seq library. Segmental Duplication Assembler (SDA) (github mrvollger branch available on Mar 31, 2020) was used to identify and unpack collapsed SDs in the bonobo assembly. Repeat content of the assembled genome was analyzed using RepeatMasker (RepeatMasker-Open-4.1.0) and the Dfam3 repeat library. We assigned lineage-specific Alu and full-length LINE, SVA_D and PTERV elements to subfamilies by applying COSEG (www.repeatmasker.org/COSEGDownload.html) to determine the lineage specific subfamily composition. Genome annotation was performed using the Comparative Annotation Toolkit (CAT) v2.1. Insertions and deletions were detected in bonobo, chimpanzee and gorilla using PBSV (version 2.2.0), Sniffles (version 1.0.10) and Smartie-sv (github zeeev branch available on Mar 8, 2018) and genotyped using Paragraph (version 2.4a) against a panel of 27 Illumina WGS genomes. We searched for evidence of ILS among the chimpanzee, gorilla and human lineages applying Prank (v.140110) to construct multiple sequence alignments and using ete3 module to identify segments under ILS. All statistics analyses were performed in R (3.5.3). We applied minimiro (github mrvollger branch available on Aug 4, 2020) for plotting synteny. Splign (NCBI updated on 02/23/15) was used for gene annotation. QV analyses were run with Merqury (version 1.0). Custom codes used in this study are available at GitHub (https://github.com/EichlerLab and https://github.com/MaoYafei).

For manuscripts utilizing custom algorithms or software that are central to the research but not yet described in published literature, software must be made available to editors and reviewers. We strongly encourage code deposition in a community repository (e.g. GitHub). See the Nature Research guidelines for submitting code & software for further information.

Data

Policy information about availability of data

All manuscripts must include a data availability statement. This statement should provide the following information, where applicable:

- Accession codes, unique identifiers, or web links for publicly available datasets
- A list of figures that have associated raw data
- A description of any restrictions on data availability

All the data and accession codes have been reported in the manuscript

Field-specific reporting

Please select the one below that is the best fit for your research. If you are not sure, read the appropriate sections before making your selection.

☐ Life sciences ☐ Behavioural & social sciences ☒ Ecological, evolutionary & environmental sciences

For a reference copy of the document with all sections, see nature.com/documents/nr-reporting-summary-flat.pdf

Ecological, evolutionary & environmental sciences study design

All studies must disclose on these points even when the disclosure is negative.

Study description	We sequenced and assembled a new bonobo reference genome using a multiplatform approach. The genome is more contiguous and accurate allowing more comprehensive sequence alignement. We discovered new species specific structural variants including gene family expansions and deletions in the ape lineage. We provide a more complete view of incomplete lineage sorting and its non-random clustering during ape genome evolution.
Research sample	We sequence a bonobo (Pygmy chimpanzee) immortalised cell line (Carbone #601152). The source of the cells was an EBV transformed lymphoblast cell line from a single female bonobo, Mhudiblu. Pygmy chimpanzee was chosen because of its importance for inferring species specific changes in both human and chimpanzee lineages. Together with chimpanzee, bonobos represent the closest great apes to human genome. The sample we sequenced is representative of Pan paniscus.
Sampling strategy	No sample size calculation was performed. We were searching for genomics and transcriptomics similarities/differences between Pan paniscus and other great ape genomes. For this purpose, deep whole genome long-read sequencing with the Pacific Biosciences Sequel II platform was performed and variants were then genotyped on a population of samples to confirm fixed or polymorphic status.
Data collection	Sequencing data for assembly were collected using Pacific Bioscience Sequel II Instrument Control SW (v7.1 or v8.0); while cytogenetics data were generated using a Leica fluorescence microscope and Leica Application Suite X (v3.7).
Timing and spatial scale	No Timing or spatial scale was applied
Data exclusions	No data were specifically excluded
Reproducibility	Computational experiments are deterministic and are, therefore, reproducible. Despite this expected reproducibility, computational experiments were performed multiple times with different parameters and followed up with experimental validation. All attempts at replication were successful.
Randomization	No randomization was performed, being a single genome sequenced and assembled.
Blinding	No blinding was requested, being a single genome sequenced and assembled.

Did the study involve field work? ☐ Yes ☒ No

Reporting for specific materials, systems and methods

We require information from authors about some types of materials, experimental systems and methods used in many studies. Here, indicate whether each material, system or method listed is relevant to your study. If you are not sure if a list item applies to your research, read the appropriate section before selecting a response.

Materials & experimental systems

n/a	Involved in the study
☒	☐ Antibodies
☐	☒ Eukaryotic cell lines
☒	☐ Palaeontology and archaeology
☒	☐ Animals and other organisms
☒	☐ Human research participants
☒	☐ Clinical data
☒	☐ Dual use research of concern

Methods

n/a	Involved in the study
☒	☐ ChIP-seq
☒	☐ Flow cytometry
☒	☐ MRI-based neuroimaging

Eukaryotic cell lines

Policy information about cell lines

Cell line source(s)

Mhudiblu (SAMN11123633), PPA Lb502 (SAMN01920504), chimpanzee (Pan troglodytes; Clint; S006007), gorilla (Gorilla gorilla; GGO9), Orangutan (Pongo abelii; Susie; PR01109) and human normal donor (with signed personal consent). All the origin of the great apes individuals we tested are reported at this link: https://www.biologiaevolutiva.org/greatape/samples.html and are available upon request according to CITES restrictions.
Mhudiblu (SAMN11123633) immortalized by EBV transformed lymphoblast cell line (Carbone #601152), was originally isolated from a single female bonobo (Pan paniscus), Mhudiblu (a.k.a. Mhudibluy, ISIS 601152, born April 2001 at San Diego Zoo or Muhdeblu when she was transferred to the Wuppertal Zoo in Germany).
PPA Lb502 (SAMN01920504), immortalized by EBV transformed lymphoblast cell line was obtained from a captive born animal and donated by Prof. Mariano Rocchi, University of Bari (Italy). GGO9 fibroblast cell lines were donated by Prof. Mariano Rocchi, University of Bari (Italy).
Chimpanzee (Pan troglodytes; Clint; S006007) fibroblast cells were originally obtained from a male Western chimpanzee named Clint (now deceased) at the Yerkes National Primate Research Center (Atlanta, GA) and immortalized with EBV.
Orangutan (Pongo abelii; Susie; PR01109) fibroblast cells were originally obtained from a female Sumatran orangutan named Susie (now deceased) at the Gladys Porter Zoo (Brownsville, TX), immortalized with EBV, and stored at the Coriell Institute for Medical Research (Camden, NJ).

Authentication

Mhudiblu was sequenced with Illumina whole-genome sequencing to confirm species and karyotyped. The other cell lines were authenticated via standard karyotype analysis.

Mycoplasma contamination

All the cell lines tested negative for Mycoplasma

Commonly misidentified lines
(See ICLAC register)

None
