# Supplementary material for: A high-quality bonobo genome refines the analysis of hominid evolution
Source: Nature. 2021 May 5;594(7861):77–81. doi: 10.1038/s41586-021-03519-x (PMC8172381; doi:10.1038/s41586-021-03519-x)
Supplement: Supplementary file 3 — Supplementary Data [file 41586_2021_3519_MOESM4_ESM.pdf]

SUPPLEMENTARY DATA

Table of Contents

1. Bonobo genome sequencing **3**
1.1 DNA source and karyotype analysis 3
1.2 PacBio genome library preparation and sequencing 3
1.3 Illumina whole-genome sequencing (WGS) 4
1.4 Iso-Seq whole-transcriptome sequencing 4

2. Genome assembly and AGP construction **4**
2.1 Genome assembly 4
2.2 Bonobo BAC library construction and clone sequencing 5
2.3 Scaffold and chromosome construction 5
2.4 Chromosomal backbone in bonobo genome 7
2.5 Validation of inversion calls 12
2.6 Mhudiblu assembly versus other great apes 16

3. Assembly quality and accuracy analyses **17**
3.1 Contiguity assessment using chimpanzee BAC-end sequence 17
3.2 Scaffolding and contiguity assessment using Strand-seq 17
3.3 Illumina-based sequence accuracy 27
3.4 BAC-based sequence accuracy 28
3.5 Bionano Genomics: optical maps and variant calling 30
3.6 Gap analysis and comparison to previous bonobo assembly 30
3.7 Divergent regions between Mhudiblu_PPA_v0 and panpan1.1 and SD overlap 32
3.8 Orientation differences between Mhudiblu_PPA_v0 and panpan1.1 35
3.9 Strand-seq analysis of panpan1.1 assembly 38
3.10 Summary of Mhudiblu assembly quality 39

4. Bonobo genome assembly analyses **40**
4.1 SD analyses 40
4.2 Collapsed SD analyses 41
4.3 Gene annotation analyses 41
4.4 Creation of Mhudiblu_PPA_v1 assembly 44

5. Incomplete lineage sorting (ILS) analysis **52**
5.1 Genome-wide ILS analyses 52
5.2 Effective population size of Pan and Pan/Homo ancestral groups 54
5.3 ILS analysis of protein-coding exons 55
5.4 Evolutionary modeling of ILS as a Poisson process and ILS desert analysis 56
5.5 ILS interdistance simulation 60
5.6 Deeper phylogenetic ILS and selection 60

6. Small structural variant (SV) analyses **66**
6.1 Discovery and genotyping of SVs in bonobo, chimpanzee and gorilla 66
6.2 SV annotations 67
6.3 SV intersection with ILS regions 68
6.4 WSSD read-depth genotyping 69

6.5 Bonobo SVs and human-specific SVs ... 74
6.6 Lineage-specific SVs disrupting exons or regulatory elements with HiFi read validation ... 75
6.7 Indel gene frameshift analyses with HiFi read validation ... 78

7. Mobile element insertion (MEI) analyses **82**
7.1 Transposable elements in Mhudiblu_PPA_v0 versus other primates ... 82
7.2 Annotation of full-length L1 elements ... 89
7.3 New bonobo assembly reveals previously hidden active young L1s ... 89
7.4 Polymorphism of MEI families in chimpanzee and bonobo ... 90
7.5 Summary of MEI analysis ... 92

8. Bonobo genomic diversity analysis and bonobo archaic introgressed regions analysis **94**
8.1 Genomic diversity among bonobo, chimpanzee and human ... 94
8.2 Bonobo archaic introgressed regions analysis ... 95
8.3 100 neurobehavioral genes intersection with bonobo-specific SVs and ILS ... 98

9. Selection analysis with new sequenced assemblies using bonobo and chimpanzee WGS **99**
9.1 Tajima's D and SweepFinder2 analyses ... 99
9.2 dN/dS positive selection ... 101
9.3 Comparison of candidate genes among positive selection tests ... 104
9.4 MHC locus selection. Mhudiblu_PPA_v0 and panpan1.1 comparison ... 105

References **107**

1. Bonobo genome sequencing

1.1 DNA source and karyotype analysis

We isolated and sequenced the genome of a single female bonobo (*Pan paniscus*), Mhudiblu (a.k.a. Mhudibluy, ISIS 601152, born April 2001 at San Diego Zoo or Muhdeblu when she was transferred at the Wuppertal Zoo in Germany). DNA was isolated from an EBV transformed lymphoblast cell line (Carbone #601152), per the protocol in Kronenberg et al.[1]. Karyotype analysis (Cell Line Genetics) of the Mhudiblu cell line source (**Supplementary Data Fig. S1**) confirmed a normal bonobo female karyotype (48, XX) based on an analysis of metaphase after the fourth passage. Cytogenetic analysis was performed on 20 G-banded metaphase cells from the bonobo cell line and 17 cells demonstrated the normal karyotype. Three cells show potential non-clonal chromosome aberrations reflecting low-level mosaicism and likely artefacts of culture (46 XX,-20,22; 48 XX, 21+mar; 48, XX, t(1p; 19p)).

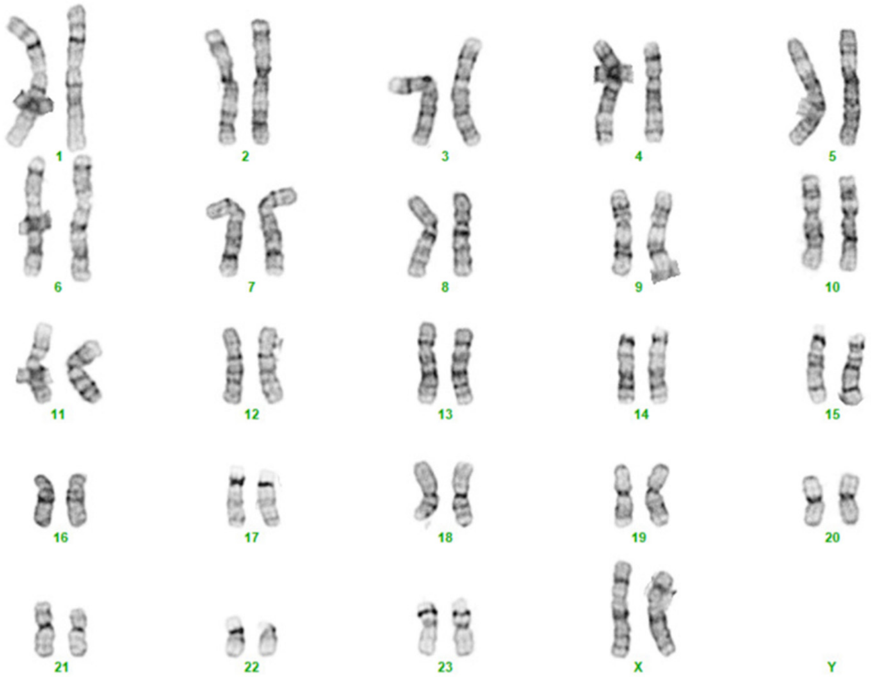

Supplementary Data Figure S1. Bonobo Mhudiblu karyotype. Chromosomal banding based on analysis of 20 cells from bonobo lymphocyte suspension culture. Chromosome assignment from bonobo classical nomenclature as opposed to phylogenetic nomenclature.

1.2 PacBio genome library preparation and sequencing

DNA fragment libraries (20-40 kbp inserts) were prepared as previously described[1] with the following modifications: DNA was sheared at the 45 kbp setting for size selection at 20 kbp, or 50 kbp setting for size selection at 30 kbp. Libraries were made at the University of Washington and sequenced at the University of Washington and Ontario Institute for Cancer Research. Loading concentrations were titrated empirically for each

library, averaging 150 pM for >20 kbp size-selected libraries, and 260 pM for >30 kbp size-selected libraries. Mhudiblu was sequenced using long-read PacBio RS II sequencing chemistry to a coverage of 74X (reads of insert [ROI], 3.2 Gbp estimated genome size) or 86X (subread, 3.2 Gbp estimated genome size), on 220 single-molecule, real-time (SMRT) cells, producing 17 million total reads with 23 million subreads (**Supplementary Table 3 and Supplementary Fig. 1**).

1.3 Illumina whole-genome sequencing (WGS)

We prepared libraries from the same cell-isolated genomic DNA using the Illumina TruSeq PCR-Free library preparation kit targeting a 550 bp insert size. We generated paired-end 250 bp WGS data on a HiSeq 2500 platform in Rapid Mode (ACCESSION: SRR11975117). Overall coverage based on mapping to chimpanzee reference Clint_PTRv2 was 40.3-fold sequence coverage.

1.4 Iso-Seq whole-transcriptome sequencing

We prepared full-length non-chimeric (FLNC) cDNA from both induced pluripotent stem cell (iPSC) and derived neuronal progenitor cell (NPC) lines[2] and extracted RNA as described previously[1]. We prepared and sequenced Iso-Seq libraries as described[3] with the following modification: in lieu of strict size fractionation, we performed sequential 0.4X/1X AMPure PB bead washes where each fraction was sequenced separately. Sequencing was performed on the Sequel platform with Sequel 3.0 chemistry. We generated two SMRT cells (1M) per sample for a total of four cells. Collected data was optionally demultiplexed, then analyzed with circular consensus sequencing (CCS) and the Iso-Seq analysis pipeline to generate FLNC reads ensuring each has a poly-A tail, plus a single 3' and 5' primer signal for downstream analysis. CCS was generated from the raw subreads with a requirement of at least 1 sequence pass and at least 0.9 identity (--minPasses 1 --min-rq 0.9). LIMA (demultiplex barcoding) was used to generate the FLNC reads from the CCS resulting in the production of 867,690 sequenced FLNC reads with an average size of 2,240 bp, an overall median quality score of 17.32, and an average of 30 passes per molecule for bonobo iPSC- and NPC-derived libraries (**Supplementary Table 8**).

2. Genome assembly and AGP construction

Note: For consistency, NCBI reference genome nomenclature has been used throughout the manuscript and corresponds to the following UCSC IDs (NCBI/UCSC): panpan1.1/panPan2, Mhudiblu_PPA_v0/panPan3, Clint_PTRv2/panTro6, Kamilah_GGO_v0/gorGor6, Susie_PABv2/ponAbe3, and GRCh38/hg38.

2.1 Genome assembly

We applied Falcon (Git id 53444482 dgordon branch available on 2017.06.13) to assemble the bonobo genome from SMRT sequence reads with length cutoff of 15 kbp. The coverage of reads ≥15 kbp is 55.7X (3.2 Gbp estimated genome size). The assembly contains 3.015 Gbp distributed amongst 4,975 contigs with an N50 of 16.580

Mbp (**Supplementary Table 4**). There were 1,088 contigs greater than 100 kbp. The assembly was error corrected using Quiver[4] and then further error corrected using Pilon[5] with 33-fold Illumina paired-end reads (assuming 3.2 Gbp genome size) (SRA ID: SRR11975117). We also applied an in-house FreeBayes-based[6] indel correction pipeline optimized to improve continuous long-read (CLR) assemblies as described in Kronenberg et al.[1] Bionano Genomics optical mapping was used to detect putative misjoins within contigs and these contigs were cut at these points. The resulting assembly is described in **Supplementary Table 4**.

2.2 Bonobo BAC library construction and clone sequencing

We constructed a large-insert BAC library (VMRC74) from bonobo Mhudiblu cell line DNA using a previously described protocol[7]. Plug DNA was partially digested with *EcoRI*, electro-eluted, ligated, and transformed into E. coli cells. We selected 350,000 clones (10-fold coverage) placing into 96-well plates using a Norgren picker and stamped onto Performa II Genetix nylon filters. The average insert size of VMRC74 was estimated at 103 kbp. We randomly chose 17 clones for PacBio insert sequencing[8] and for assessment of sequence accuracy (**section 3.4**).

2.3 Scaffold and chromosome construction

The contigs from the assembly were ordered and oriented into scaffolds using Bionano Genomics optical maps. The Bionano Genomics Saphyr System was used to generate optical molecules using two nicking endonuclease enzymes, Nb.BssSI and Nt.BspQI, and *de novo* assembled into maps for each enzyme. The contigs were aligned to the consensus maps and placed into scaffolds using the HybridScaffolds suite from the Bionano Genomics Access software (pipeline version 4573 and RefAligner version 7376). HybridScaffolds placed 769 contigs of Mhudiblu_PPA_v0 into 149 scaffolds. Overall, scaffold N50 was 70.7 Mbp (**Supplementary Data Table S1**) similar to the 121 scaffolds with N50 of 60 Mbp obtained for chimpanzee and 73 scaffolds with N50 of 102 Mbp obtained for orangutan[1].

We constructed a chromosomal-level AGP (a golden path) for Mhudiblu_PPA_v0 without guidance from the human reference genome based on a FISH BAC clone-order framework[9] integrated with Bionano Genomics optical maps of scaffolded contigs. After sequence contigs (>150 kbp) were scaffolded by Bionano Genomics (above), we used FISH probes to assign and order scaffolds into chromosomes. Fully sequenced large-insert BACs from *Pan troglodytes* library CHORI-251 assisted in guiding this process. We then generated chromosome assemblies using the same approach described for the other ape reference genomes[1]. Briefly, BAC sequences were mapped to the scaffolds using BLASR based on which scaffolds were grouped into 24 categories—one for each chromosome and an unplaced group. Scaffolds were thus grouped into chromosome groups. This approach successfully placed 87 scaffolds into 24 chromosomal bins.

Within each chromosome bin, the order of mapping of each set of BAC sequences is known (http://www.biologia.uniba.it/5-bonobo/). We use this prior knowledge to lay out scaffold sets into a meaningful order of sequences. Multiple BAC alignments within each scaffold makes it possible to determine the orientation of the scaffolds (increasing or decreasing distance of probe mappings). We ordered all chromosomes by using the data from the FISH alignments. PacBio read depth and BAC-end sequence (BES) from *Pan troglodytes* library CHORI-251 were used to validate the order and orientation of the resulting chromosome assemblies; 87 Bionano Genomics scaffolds representing 637/769 contigs in total could be placed into chromosomes. This represents 2,787,284 kbp of the bonobo genome. In total, 324 BACs with FISH mappings were aligned to Bionano Genomics Mhudiblu_PPA_v0 scaffolds. The BAC order data for each chromosome were obtained for the autosomes from a previously defined chromosomal backbone (http://www.biologia.uniba.it/5-bonobo/) and for the X chromosome from Stanyon et al., 2008[10]; the sequences were obtained from NCBI. For each chromosome, fully sequenced BACs, if present, were also used to anchor scaffolds. The number of BACs per chromosome is shown in **Supplementary Data Table S2**.

Supplementary Data Table S1. Bonobo genome scaffold statistics

Number of scaffolds	149
Total size of scaffolds	2,839,690,581
Longest scaffold	158,698,778
Shortest scaffold	82,286
Bases in scaffolds > 1 kbp. % of all scaffolds	2,803,145,545 (100.0%)
Bases in scaffolds > 10 kbp. % of all scaffolds	2,803,145,545 (100.0%)
Bases in scaffolds > 100 kbp. % of all scaffolds	2,793,836,132 (99.7%)
Bases in scaffolds > 1 Mbp. % of all scaffolds	2,672,612,435 (95.3%)
Bases in scaffolds > 10 Mbp. % of all scaffolds	1,946,871,690 (69.5%)
Mean scaffold size	19,058,326
Median scaffold size	2,735,219
N50 scaffold length	70,689,685
L50 scaffold count	15

Supplementary Data Table S2. AGP construction using FISH-anchored BACs

chromosome	#probe	chromosome	#probe
chr1	20	chr12	13
chr2a	13	chr13	11
chr2b	16	chr14	9
chr3	20	chr15	11
chr4	21	chr16	10
chr5	18	chr17	13
chr6	14	chr18	9
chr7	15	chr19	8
chr8	14	chr20	8
chr9	13	chr21	5
chr10	13	chr22	5
chr11	13	chrX	32

See Supplementary Data Table S6 for complete list of probes

2.4 Chromosomal backbone in bonobo genome

To detect Mbp-scale structural variants (SVs) distinguishing human and bonobo genomes, we tested all 23 bonobo autosomes using 292 human BAC clones as probes in serial FISH experiments (**Supplementary Data Table S3**). Briefly, we performed three- or four-color experiments on metaphases using overlapping windows of BAC clones so that the last probe in a set was also the first one of the next set. Probes were selected to uniformly cover all the genomic regions: BACs were more than 3 Mbp and less than 20 Mbp apart (**Fig. 1a**). We selected a higher density of probes in those regions associated with evolutionary breakpoints in other great apes. We FISH mapped all BACs on both bonobo (from a *Pan paniscus* lymphoblast cell line, LB502) and human (obtained from PHA-stimulated peripheral lymphocytes of normal donors) metaphases: hybridizations on human chromosomes were used as a control for probe order, while results on bonobo metaphases allowed us to evaluate the presence of SV events differentiating bonobo and human karyotypes (FISH experiments were performed with minor modifications following the protocol of Lichter. 1990[11]). All FISH results are available online: http://www.biologia.uniba.it/5-bonobo/.

We also performed subsequent FISH experiments with both human (RPCI-11) and chimpanzee (CHORI-251) BAC clones to map scaffolds >500 kbp in length that were initially unassigned. The procedure successfully placed 11 previously unassigned scaffolds (totaling 60 Mbp) and correctly determined the orientation of 3 scaffolds (7 Mbp) enabling the discovery of novel structural differences with respect to the human genome (GRCh38) (**Supplementary Data Table S4**).

Supplementary Data Table S3. Human BAC clones used to build the bonobo chromosomal backbone (#1-292) and to anchor chromosome X scaffolds (#293-324)

#	Clone	Mapping (GRCh38/hg38)	#	Clone	Mapping (GRCh38/hg38)
1	RP11-421C4	chr1:1322240-1507586	163	RP11-479D10	chr9:129826205-129996381
2	RP11-265F14	chr1:15431609-15612015	164	RP11-644H13	chr9:137640660-137825275
3	RP11-266K22	chr1:31249293-31410118	165	RP11-69C17	chr10:1978746-2181918
4	RP11-55M23	chr1:54975756-55146649	166	RP11-348M15	chr10:12003121-12192936
5	RP11-316C12	chr1:71360573-71566819	167	RP11-344N19	chr10:23613726-23787065
6	RP11-254E16	chr1:84301524-84451179	168	RP11-1055G9	chr10:30791665-30986608
7	RP11-138K16	chr1:99490195-99666174	169	RP11-669H9	chr10:43002529-43175542
8	RP11-284N8	chr1:110553130-110746313	170	RP11-122B11	chr10:53728003-53887300
9	RP11-192J8	chr1:117825239-117989455	171	RP11-749A7	chr10:61771768-61945315
10	RP11-114O18	chr1:119990856-120141146	172	RP11-640K24	chr10:73313882-73483445
11	RP11-293N20	chr1:145784569-145960930	173	RP11-179J5	chr10:82650035-82843561
12	RP11-98F1	chr1:155303245-155307239	174	RP11-684J19	chr10:94073324-94255622
13	RP11-655L16	chr1:160688858-160865459	175	RP11-653D19	chr10:104192043-104365508
14	RP11-332H17	chr1:170006601-170114647	176	RP11-1114E11	chr10:117300906-117466815
15	RP11-152A16	chr1:179104010-179299901	177	RP11-92A10	chr10:130322577-130477598
16	RP11-553K8	chr1:198514843-198725175	178	RP11-1021K7	chr11:484063-678676
17	RP11-57I17	chr1:207554753-207751766	179	RP11-765A24	chr11:12460671-12630203
18	RP11-324K19	chr1:220963475-220979024	180	RP11-822E5	chr11:24384788-24584759
19	RP11-499N12	chr1:228718470-228882077	181	RP11-999E19	chr11:36060533-36250546
20	RP11-385F5	chr1:236522901-236736658	182	RP11-697C24	chr11:48068985-48257221
21	RP11-457A20	chr2:4372378-4546927	183	RP11-1065B14	chr11:60120979-60320208
22	RP11-496P1	chr2:14094885-14295327	184	RP11-378K8	chr11:69389098-69570465
23	RP11-527P23	chr2:22676756-22860422	185	RP11-831B21	chr11:79550819-79734961
24	RP11-322P19	chr2:33375074-33568483	186	RP11-625B1	chr11:91667610-91853548
25	RP11-339H12	chr2:43111652-43327183	187	RP11-1044B1	chr11:105734023-105946755
26	RP11-542O24	chr2:53234784-53406706	188	RP11-486A21	chr11:115131973-115319404
27	RP11-511I11	chr2:63003656-63160828	189	RP11-705A7	chr11:127192575-127378055
28	RP11-434P11	chr2:73589868-73802940	190	RP11-1077I24	chr11:134623632-134819369
29	RP11-495B16	chr2:82531044-82635830	191	RP11-691J6	chr12:5220573-5404703
30	RP11-685C7	chr2:89089140-89303283	192	RP11-1006F8	chr12:16185450-16364080
31	RP11-351J10	chr2:95578909-95745563	193	RP11-877E17	chr12:25941824-26119806
32	RP11-519H15	chr2:106864448-107054396	194	RP11-956A19	chr12:32129954-32319969
33	RP11-67L14	chr2:112784597-112938794	195	RP11-490D11	chr12:41432820-41600134
34	RP11-1146A22	chr2:116341854-116514929	196	RP11-845M18	chr12:52123910-52282204
35	RP11-350P7	chr2:122282704-122456370	197	RP11-766N7	chr12:64820603-65004855
36	RP11-313N8	chr2:127127260-127340322	198	RP11-461F16	chr12:76839268-76981608
37	RP11-458A7	chr2:132800148-132955682	199	RP11-1129M3	chr12:88764863-88916000
38	RP11-1140A6	chr2:136039918-136187124	200	RP11-746J15	chr12:100789743-100936263
39	RP11-379G6	chr2:144483981-144677309	201	RP11-932J23	chr12:112859733-113027570
40	RP11-357O18	chr2:154475573-154662959	202	RP11-344G11	chr12:126012839-126159489
41	RP11-1146M20	chr2:164831901-164968029	203	RP11-867C16	chr12:132274613-132459012
42	RP11-504O20	chr2:175810252-175986630	204	RP11-110K18	chr13:19932076-20095942
43	RP11-335G13	chr2:185917041-186097255	205	RP11-473H7	chr13:31191691-31377829
44	RP11-449J2	chr2:195627117-195787163	206	RP11-374E11	chr13:42274008-42442731
45	RP11-1030A22	chr2:205364097-205548033	207	RP11-996I3	chr13:48353718-48558786
46	RP11-804M4	chr2:215270891-215367518	208	RP11-705O23	chr13:55406852-55587663
47	RP11-573O16	chr2:225495420-225682687	209	RP11-412L6	chr13:62487280-62654174
48	RP11-785G17	chr2:236367560-236369102	210	RP11-316L8	chr13:73948661-74134795
49	RP11-463B12	chr2:239971859-240143278	211	RP11-351H1	chr13:84924636-85110426
50	RP11-151A4	chr3:619490-778737	212	RP11-721F14	chr13:96942592-97125228
51	RP11-933I8	chr3:4370218-4592064	213	RP11-925H8	chr13:108355585-108533621
52	RP11-732C9	chr3:12425258-12632543	214	RP11-330H4	chr13:113977137-114153357
53	RP11-421B21	chr3:15147209-15324532	215	RP11-463G16	chr14:20343832-20489572
54	RP11-109D5	chr3:25481081-25680896	216	RP11-426H12	chr14:30877667-31057180
55	RP11-491D6	chr3:37034150-37136520	217	RP11-625F13	chr14:42093383-42258989
56	RP11-395P16	chr3:47584747-47778906	218	RP11-876B21	chr14:52882149-53072472
57	RP11-380J21	chr3:64182355-64200696	219	RP11-698P9	chr14:63434726-63600201

58	RP11-634L22	chr3:75320385-75496761	220	RP11-653K5	chr14:74291709-74476901
59	RP11-655A17	chr3:87099698-87270490	221	RP11-799P8	chr14:81481287-81656060
60	RP11-454H13	chr3:101597159-101805358	222	RP11-91C7	chr14:91013123-91156019
61	RP11-760N7	chr3:119250208-119430254	223	RP11-90G22	chr14:100674834-100852920
62	RP11-21N8	chr3:130609089-130759665	224	RP11-441B20	chr15:25109084-25277009
63	RP11-702M4	chr3:139730332-139918147	225	RP11-360J18	chr15:29751155-29944864
64	RP11-680B3	chr3:148849430-148970703	226	RP11-1056G8	chr15:31762281-31962123
65	RP11-498P15	chr3:162329841-162447611	227	RP11-1078O1	chr15:35692886-35873288
66	RP11-526M23	chr3:166898263-167089798	228	RP11-133K1	chr15:40224159-40320632
67	RP11-796F15	chr3:177530862-177712507	229	RP11-490E13	chr15:45918730-46096611
68	RP11-693H4	chr3:186483083-186640024	230	RP11-235L4	chr15:50637437-50822809
69	RP11-313F11	chr3:195995779-195996379	231	RP11-1072A24	chr15:55720489-55901226
70	RP11-61B7	chr4:49539-246359	232	RP11-1107A19	chr15:73994195-74138045
71	RP11-963C8	chr4:8612511-8691523	233	RP11-351I10	chr15:83875250-84053640
72	RP11-362I16	chr4:22391588-22554788	234	RP11-806N11	chr15:99956784-100150259
73	RP11-418L2	chr4:29119999-29288895	235	RP11-292B10	chr16:3548331-3774065
74	RP11-822G2	chr4:38985913-39141900	236	RP11-352C16	chr16:13521525-13678658
75	RP11-439B18	chr4:45547930-45735280	237	RP11-450G5	chr16:24174446-24362603
76	RP11-317G22	chr4:48892516-49076721	238	RP11-939G23	chr16:30974373-31190025
77	RP11-365H22	chr4:51793952-51971936	239	RP11-352B15	chr16:35674953-35852363
78	RP11-1043B22	chr4:55494794-55667260	240	RP11-627O2	chr16:46809993-46987723
79	RP11-323K3	chr4:62614130-62834699	241	RP11-497D8	chr16:54886483-55073381
80	RP11-669F1	chr4:68215077-68346492	242	RP11-843B10	chr16:63864336-64038109
81	RP11-367P3	chr4:84898851-85121692	243	RP11-652E7	chr16:73931479-74125635
82	RP11-10L7	chr4:88263111-88375401	244	RP11-757F20	chr16:83883892-84060839
83	RP11-499E18	chr4:102294504-102458418	245	RP11-411G7	chr17:590738-722442
84	RP11-510D4	chr4:117501162-117677187	246	RP11-769H22	chr17:8005136-8167132
85	RP11-758B24	chr4:131534979-131719365	247	RP11-908P24	chr17:12681620-12870341
86	RP11-780M14	chr4:143735009-143836046	248	RP11-385D13	chr17:15523701-15591491
87	RP11-663M18	chr4:158820725-158900932	249	RP11-765A10	chr17:21016312-21191717
88	RP11-453M2	chr4:172250454-172295292	250	RP11-28A22	chr17:34491422-34648144
89	RP11-335L23	chr4:182979094-183156816	251	RP11-102M17	chr17:42303173-42459295
90	RP11-462G22	chr4:189510076-189666453	252	RP11-671B19	chr17:47273653-47434196
91	RP11-58A5	chr5:4912581-5070042	253	RP11-170D6	chr17:52207751-52367817
92	RP11-1078G18	chr5:14781638-14946577	254	RP11-619I22	chr17:59716487-59816059
93	RP11-875A6	chr5:18275727-18467473	255	RP11-450M16	chr17:64157370-64318016
94	RP11-8O18	chr5:22523208-22682048	256	RP11-449L23	chr17:73397229-73600150
95	RP11-94E6	chr5:33701408-33890153	257	RP11-1033I8	chr17:82035100-82251150
96	RP11-159F24	chr5:43493046-43509046	258	RP11-78H1	chr18:2146811-2317215
97	RP11-948O11	chr5:54033664-54201967	259	RP11-104G22	chr18:7285085-7438360
98	RP11-298P6	chr5:64774758-64926575	260	RP11-345O3	chr18:12860898-13035434
99	RP11-580F18	chr5:75508740-75665530	261	RP11-10G8	chr18:21440480-21597043
100	RP11-117J12	chr5:88082372-88242440	262	RP11-104N11	chr18:37602650-37774743
101	RP11-297G19	chr5:93984648-94102204	263	RP11-61D1	chr18:54394432-54532766
102	RP11-1147K5	chr5:96710693-96863658	264	RP11-765G2	chr18:63861286-64052185
103	RP11-326M11	chr5:105830574-105992277	265	RP11-53N15	chr18:74214074-74377910
104	RP11-3B10	chr5:112628475-112798814	266	RP11-87C15	chr18:79957305-80115348
105	RP11-409A17	chr5:125647552-125770678	267	RP11-75H6	chr19:951642-1144487
106	RP11-6N3	chr5:137317779-137492878	268	RP11-777K22	chr19:8975082-9140467
107	RP11-654C10	chr5:155775061-155969395	269	RP11-207I16	chr19:15292240-15475552
108	RP11-1056G6	chr5:179757833-179933208	270	RP11-965D17	chr19:23564999-23757798
109	RP11-945M14	chr6:306719-532394	271	RP11-615P5	chr19:34190261-34375875
110	RP11-4A24	chr6:12129844-12295102	272	RP11-108I20	chr19:41954335-42138189
111	RP11-656I18	chr6:29214636-29423125	273	RP11-690A4	chr19:51819780-51992885
112	RP11-1147I20	chr6:41994215-42149073	274	RP11-5D4	chr19:57590944-57777116
113	RP11-709D10	chr6:50762551-50938607	275	RP11-300H9	chr20:310751-482704
114	RP11-346M3	chr6:61688524-61862715	276	RP11-690B9	chr20:11064481-11266057
115	RP11-415D17	chr6:75351620-75504848	277	RP11-796K22	chr20:20991004-21190952

116	RP11-1142J10	chr6:85495076-85657004	278	RP11-313J23	chr20:31447535-31604032
117	RP11-451P21	chr6:96433571-96592305	279	RP11-826B14	chr20:37270638-37487146
118	RP11-696I24	chr6:111639299-111835447	280	RP11-1151C1	chr20:47466105-47627843
119	RP11-769G14	chr6:127448902-127609607	281	RP11-948A3	chr20:57666378-57866556
120	RP11-762O13	chr6:141622365-141806308	282	RP11-939M14	chr20:63195934-63386036
121	RP11-905B16	chr6:156579997-156757646	283	RP11-1084C3	chr21:21315934-21502425
122	RP11-597K5	chr6:168638841-168796056	284	RP11-833P5	chr21:27130252-27315090
123	RP11-383J8	chr7:1585465-1801430	285	RP11-369E2	chr21:33147828-33313752
124	RP11-1080O3	chr7:6385924-6607593	286	RP11-433L22	chr21:39566417-39730691
125	RP11-314M16	chr7:20004657-20184195	287	RP11-433E24	chr21:46472858-46667726
126	RP11-589I24	chr7:29865719-30052786	288	RP11-481H20	chr22:19202188-19392674
127	RP11-420P20	chr7:40242116-40421435	289	RP11-799F16	chr22:26262121-26466650
128	RP11-719L20	chr7:53243936-53402954	290	RP11-639B9	chr22:34862829-35019819
129	RP11-118D11	chr7:67413330-67571355	291	RP11-714P2	chr22:41144226-41313040
130	RP11-1H6	chr7:79618378-79786037	292	RP11-1109B4	chr22:48293106-48466768
131	RP11-380E8	chr7:89646719-89795313	293	RP11-800K15	chrX:552370-733500
132	RP11-282M13	chr7:102863346-103030180	294	RP11-458E23	chrX:10297383-10473812
133	RP11-1143O12	chr7:113009272-113168783	295	RP11-450P7	chrX:21605746-21729931
134	RP11-458H8	chr7:123957697-124165422	296	RP11-450E21	chrX:33496543-33600659
135	RP11-1029H2	chr7:134920744-135120470	297	RP11-64P15	chrX:33598661-33764658
136	RP11-638B18	chr7:145985934-146148457	298	RP11-1078G21	chrX:33734303-33926309
137	RP11-656C10	chr7:157027825-157181025	299	RP11-825L2	chrX:34142911-34329485
138	RP11-1072H3	chr8:536256-718397	300	RP11-281B1	chrX:34212157-34396522
139	RP11-637G16	chr8:10687998-10860537	301	RP11-910L4	chrX:34256179-34431083
140	RP11-908D16	chr8:19929747-20103598	302	RP11-831J15	chrX:34370279-34523237
141	RP11-380E12	chr8:32365511-32543118	303	RP11-11OE4	chrX:34984911-35142166
142	RP11-384C8	chr8:42319315-42501360	304	RP11-384A17	chrX:43624554-43777466
143	RP11-661F19	chr8:51140235-51321571	305	RP11-552J9	chrX:52644060-52654900
144	RP11-348C4	chr8:61621792-61825414	306	RP11-358I8	chrX:56967666-57168360
145	RP11-1144P22	chr8:71613982-71762370	307	RP11-978L24	chrX:62468155-62689174
146	RP11-643A12	chr8:78108603-78285312	308	RP11-148E15	chrX:63250997-63415254
147	RP11-1019O18	chr8:86325047-86494339	309	RP11-135B16	chrX:63323714-63491626
148	RP11-662P7	chr8:97679282-97840303	310	RP11-213M6	chrX:63788411-63951464
149	RP11-367G7	chr8:108895586-109096988	311	RP11-151C15	chrX:63871479-64047605
150	RP11-760H22	chr8:119911541-120116448	312	RP11-754F6	chrX:63896531-64055711
151	RP11-350K18	chr8:139910353-140112191	313	RP11-346J4	chrX:64035058-64229126
152	RP11-1107A23	chr9:603423-821929	314	RP11-625B4	chrX:70718332-70881288
153	RP11-77E14	chr9:7681920-7835211	315	RP11-395L12	chrX:82072641-82121408
154	RP11-639K17	chr9:17672399-17865762	316	RP11-483J19	chrX:93400906-93553285
155	RP11-1006E22	chr9:27152246-27341370	317	RP11-449F11	chrX:97834914-97997899
156	RP11-419G16	chr9:37991366-38207398	318	RP11-426L6	chrX:105800412-105955839
157	RP11-876N18	chr9:69027005-69232028	319	RP5-874H6	chrX:112851291-112872996
158	RP11-791A8	chr9:78955125-79149703	320	RP11-243N2	chrX:116121430-116285910
159	RP11-1111A4	chr9:89000184-89176424	321	RP11-488B15	chrX:126036256-126187831
160	RP11-718P15	chr9:98082680-98251852	322	RP11-535K18	chrX:136141306-136323713
161	RP11-358A7	chr9:107864248-108052170	323	RP11-478P19	chrX:144540160-144715534
162	RP11-64P14	chr9:122502713-122691033	324	RP11-402H20	chrX:154980097-155159955

Supplementary Data Table S4. FISH mapping of bonobo scaffolds

Mhudiblu scaffold ID	Mapping (GRCh38/hg38)	Size of interest (Mb)	Reason	FISH exp	internal probe 1	Mapping (GRCh38/hg38)	internal probe 2	Mapping (GRCh38/hg38)	external probe	Mapping (GRCh38/hg38)
Super_Scaffold_14	chr10:48000000-49500000	1.5	inversion / possible misassembly	Interphase FISH	RP11-370D10	chr10:48338924-48500801	RP11-565O15	chr10:49058717-49229763	CH251-489I21	chr10:49674448-49862901
Super_Scaffold_67	chr15:85248920-101851334	16.6	unoriented scaffold	Metaphase FISH	CH251-170C20	chr15:88801267-88970314	CH251-495P14	chr15:92927348-93114429		
Super_Scaffold_48	chr1:1364198-13248630	11.9	unoriented scaffold	Metaphase FISH	CH251-21P16	chr1:2463000-2782080	CH251-200P24	chr1:10965706-11160834		
Super_Scaffold_49	chr4:23681-3898272	3.9	unoriented scaffold	Metaphase FISH	CH251-2A18	chr4:59494-254060	CH251-63H1	chr4:3586412-3761067		
Super_Scaffold_54	chr7:63811262-67266938	3.5	unoriented scaffold	Metaphase FISH	CH251-232G19	chr7 63885916 64078087	CH251-552E11	chr7 66907924 67082002		
Super_Scaffold_29	chr7:40050000-44005000	4.00	inversion / possible misassembly	Metaphase FISH	RP11-321C5	chr7:39703279-39863591	RP11-643N15	chr7:43296178-43507286	RP11-1152C21	chr7:35647439-35803586
Super_Scaffold_78	chr8:305413-12508674	12.2	unoriented scaffold	Metaphase FISH	CH251-75K11	chr8:788708-932681	CH251-82K16	chr8 5915039 6062027		
Super_Scaffold_90	chr8:8218451-11980130	3.8	unoriented scaffold	Metaphase FISH	WI2-0785E15	chr8:8344222-8387247	WI2-3642O12	chr8:11391604-11435344		
Super_Scaffold_95	chr13:45650000-46500000	0.8	inversion	Interphase FISH	RP11-947C16	chr13:45443682-45629849	RP11-179M2	chr13:46009783-46189439	RP11-1148O18	chr13:44852639-45026480
Super_Scaffold_92	chr16:29554878-33598151	4.00	unoriented scaffold	Interphase FISH	WI2-2372K22	chr16:29640341-29683607	WI2-0669B08	chr16:30580941-30619130	WI2-0500J03	chr16:28992621-29032184
Super_Scaffold_88	chr17:16821376-18372611	1.6	unoriented scaffold	Interphase FISH	RP11-356G14	chr17:17016660-17203604	RP11-809H20	chr17:17690706-17862265	RP11-468C12	chr17:16474507-16639090
Super_Scaffold_57	chr17:18559715-20335528	1.8	unoriented scaffold	NA						
Super_Scaffold_80	chr19:481453-1127106	0.6	unoriented scaffold	Interphase FISH	WI2-3236K5	chr19:482617-525499	RP11-878J15	chr19:959522-1144510	WI2-0624B16	chr19:1623162-1665835
Super_Scaffold_41	chr12:132475300-133249611	0.8	unoriented scaffold	Interphase FISH	RP11-148L11	chr12:132462330-132666776	RP11-394D10	chr12:132947431-133121501	RP11-375D22	chr12:132027654-132222209

2.5 Validation of inversion calls

There are 39 regions that have long been known as differing in orientation among human, chimpanzee, and bonobo karyotypes (**Supplementary Data Table S5**). In addition, Porubsky et al.[12] recently detected 216 regions showing inverted orientation in bonobo with respect to the human genome: 128/216 were annotated as homozygous inversions, while 88/216 were heterozygous inversions. We compared them with the 39 known inversions and found a perfect overlapping for 23/216 events (21 homozygous and 2 heterozygous). We also removed from further analysis all regions composed by more than 80% of segmental duplications (SDs) or repeats (17/128 homozygous and 69/88 heterozygous), obtaining a total of 107 regions to be studied.

Supplementary Data Table S5. Known inversion events regarding chimpanzee, bonobo and human

	#	Mapping (GRCh38/hg38)	Inversion ID	References	Notes	In the ancestor
	1	chr1:87288446-145415657	chrlpericen[a]	Szamalek et al., 2006		HSA
	2	chr1:147079442-147925603	chr1_inv3	Catacchio et al., 2018	Human build38 ref wrong orientation or minor allele	HSA-PTR-GGO; ILS or recurrent inv in PTR
	3	chr2:99548000-102250000	2q11.2	Kronenberg et al., 2018		PTR
	4	chr2:106500000-108800000	2q12.2-q13	Kronenberg et al., 2018		ND
	5	chr4:4247000-8757000	4p16.1	Kronenberg et al., 2018	Same as chr4_inv5 (chr4:4182444-9339607) validated as not inverted in Chimp by Catacchio et al., 2018	GA polymorphic
	6	chr4:44809658-85037105	chr4_inv1[a]	Catacchio et al., 2018		PTR
	7	chr5:18553211-96585715	chr5_inv1[a]	Catacchio et al., 2018		PTR
	8	chr5:99582578-100374690	chr5_inv2	Catacchio et al., 2018	Partially overlapping with 5q21.1 (chr5:99400000-100200000) predicted in Chimp by Kronenberg et al., 2018; polymorphic in PTR (Catacchio et al., 2018)	ND
	9	chr5:99584037-100069297	chr5_inv3	Catacchio et al., 2018		ND
	10	chr7:5997690-6732324	chr7_inv9	Catacchio et al., 2018	Polymorphic in human (Feuk et al., 2005; Sanders et al., 2016); same as 7p22.1 (chr7:5892000-6834000) validated in Chimp by Kronenberg et al., 2018	HSA
	11	chr7:39545072-43961659	chr7_inv5	Catacchio et al., 2018	Partially overlapping with 7p14-13 (chr7:39000000-44000000) validated in Chimp by Kronenberg et al., 2018	HSA
	12	chr7:53188941-53862225	chr7_inv10	Catacchio et al., 2018	Partially overlapping with 7p12.1-p11.2 (chr7:52914843-54475123) validated in Chimp by Kronenberg et al., 2018	HSA
	13	chr7:71693970-74869950	chr7_inv6	Catacchio et al., 2018		PTR
	14	chr9:38733849-86315785	chr9_missed[a]	Catacchio et al., 2018		PTR
A	15	chr10:46870207-47457081	chr10_inv6	Catacchio et al., 2018	Polymorphic in human (Catacchio et al., 2018; Sanders et al., 2016); partially overlapping with 10q11.22 (chr10:46500000-47500000) validated in Chimp by Kronenberg et al., 2018	HSA
	16	chr10:79500000-80250000	10q22.3	Kronenberg et al., 2018	Polymorphic in human (Sanders et al., 2016)	ND
	17	chr12:20822597-67987723	chr12_inv1[a]	Catacchio et al., 2018		PTR
	18	chr13:45376000-46463000	13q14.13	Kronenberg et al., 2018		PTR/GGO
	19	chr13:52242000-52507000	13q14.13	Kronenberg et al., 2018		HSA
	20	chr15:1-31438802	chr15_missed[a]	Catacchio et al., 2018		PTR
	21	chr15:28852754-30406229	chr15_inv1	Catacchio et al., 2018		HSA-PTR-GGO; ILS or recurrent inv in PTR
	22	chr15:82300000-84500000	15q25.2	Kronenberg et al., 2018		Non-human Ape
	23	chr16:14960000-15083000	16p13.11	Kronenberg et al., 2018		ND
	24	chr16:1808000-2152000	16p13.3	Kronenberg et al., 2018		ND
	25	chr16:28781227-30210335	chr16_inv3	Catacchio et al., 2018		PTR
	26	chr16:34938757-46474677	chrXVIpericen[a]	Goidts et al., 2006		PTR
	27	chr16:70075634-74327699	chr16_inv1	Catacchio et al., 2018	Partially overlapping with 16q22.1-q23.1 (chr16:70000000-75000000) validated in Chimp by Kronenberg et al., 2018	HSA-PTR-GGO; ILS or recurrent inv in PTR
	28	chr17:8027676-49543141	chr17_inv1[a]	Catacchio et al., 2018		PTR
	29	chr18:112546-15275658	chr18_inv1[a]	Catacchio et al., 2018		HSA
	30	chr19:36331795-37251831	chr19_inv2	Catacchio et al., 2018	Partially overlapping with 19q13.12 (chr19:35957317-37537550) validated in Chimp by Kronenberg et al., 2018	PTR
	31	chr20:25500000-26000000	20p11.21-p11.1	Kronenberg et al., 2018		ND
	32	chrX:120000000-120500000	Xq24	Kronenberg et al., 2018		ND
	33	chrX:52074000-52180000	Xp11.22	Kronenberg et al., 2018		ND
	1	chr2:94725912-111484366	chr2_inv1	Catacchio et al., 2018		HSA-PTR
	2	chr8:8242347-12174746	chr8_inv2	Catacchio et al., 2018	Polymorphic in human (Giglio et al., 2001; Antonacci et al., 2009; Sanders et al., 2016)	HSA-PTR
	3	chr9:49963-67830571	chr9_inv1	Catacchio et al., 2018		HSA-PTR
B	4	chr16:28378167-29034255	chr16_inv7	Catacchio et al., 2018		HSA-PTR
	5	chr16:29640910-30210335	chr16_inv8	Catacchio et al., 2018	Two independent inversions involving this region occurred during primates evolution, therefore this region in chimpanzee appears to be in the opposite orientation respect to humans	HSA-PTR
C	1	NA	IIq pericentric inversion	Dutrillaux et al., 1975; Stanyon et al., 1986		PPA

[a] >10 Mbp inversions; A) Regions in opposite orientation between human and chimpanzee; B) Regions inverted in the human-chimpanzee ancestor; C) Bonobo-specific inversion.

Among the 107 filtered calls detected by strand-sequencing (Strand-seq), we found seven events where Strand-seq data itself were not able to determine inversion multiplicity. Three of these events correspond to pericentric inversions for which the Strand-seq was unable to span the centromere and only one side of the inversion was called (Strand-seq_chr9_inv2, Strand-seq_chr10_inv2, and Strand-seq_chr16_inv2). In two cases the inversion called is, instead, a direct region flanked by two real inversion events (Strand-seq_chr15_inv1 and Strand-seq_chr7_inv5) and twice two big regions composed by smaller events were called as a whole inversion (Strand-seq_chr1_inv8 and Strand-seq_chr7_inv4). The four latter were manually curated and allowed us to detect four additional inversions (Strand-seq_chr1_inv8a, Strand-seq_chr7_inv4a and b, and Strand-seq_chr10_inv2a) (**Supplementary Table 40**). Overall, we studied the 150 (39 + 107 + 4) regions by FISH, Bionano Genomics optical maps, and BES mapping from chimpanzee, based on the detection limit of each method.

Thirty-five out of 150 inversions were tested by FISH in bonobo, chimpanzee and human, using interphase three-color FISH for inversions between 400 kbp and 2 Mbp in size and metaphase two-color FISH for inversions larger than 2 Mbp (**Supplementary Table 40** and **Supplementary Data Table S6**). Forty-five out of 150 inversions were detected by Bionano Genomics automated (**section 3.5**) or manual SV callsets. We investigated the BES pair mapping profiling of the tested inversions by downloading the BES from the chimpanzee CHORI-251 library and mapping them to the human reference genome, GRCh38. BACs spanning inversion breakpoints are discordant for ends mapping too far apart and/or with an incorrect orientation when mapped to the human reference genome[13]. Forty out of 150 had BACs spanning at least one breakpoint. Of these, 31 showed discordant clones supporting the inversion and 9 showed concordant clones mapping at the inversion breakpoints (**Supplementary Table 40**).

In summary, human and chimpanzee chromosomes have long been known to differ by nine large (>10 Mbp) pericentric inversions, two of which are specific to the human lineage, while the remaining seven occurred in the *Pan* ancestor[9,14-17]. A higher quality assembly of the chimpanzee identified an additional 24 smaller inversions (<5 Mbp) distinguishing human and chimpanzee plus five additional regions found to have inverted in the human–chimpanzee ancestor[1,17]. The only inversion reported to date as distinguishing bonobo and chimpanzee karyotypes (therefore specific to the bonobo lineage) is a pericentric inversion of chromosome 2B[12,18,19]; thus, in total, there are 39 known inversions differentiating human, chimpanzee, and bonobo karyotypes (**Supplementary Data Table S5**). Additionally, single-cell Strand-seq recently identified 216 inversions[12] ranging in size from 1.5 kbp to 78 Mbp. After manual curation, including removing inversions composed of ≥80% SDs, all remaining Strand-seq and known inversions (150) were further tested using experimental methods as well as literature searches (**Supplementary Table 40**). Based on our analyses, we confirm all nine larger inversions in bonobo and create a FISH-based chromosomal backbone for our bonobo assembly (Fig. 1a and b). We identify 17 fixed inversions differentiating

bonobo from chimpanzee of which 11 are bonobo specific (**Supplementary Table 39**) and 22 regions likely represent bonobo inversion polymorphisms (**Supplementary Table 40**).

Supplementary Data Table S6. Clones used for FISH assays

Inversion	Mapping (GRCh38/hg38)	Size (Mb)	Cytogenetic test	internal probe 1	Mapping (GRCh38/hg38)	internal probe 2	Mapping (GRCh38/hg38)	external probe	Mapping (GRCh38/hg38)
Strand-seq_chr1_inv5	chr1:113089220-120178667	7.1	Metaphase FISH	RP11-351P7	chr1:113746443-113897381	RP11-192J8	chr1:117825240-117989455		
chr1_inv3	chr1:147079442-147925603	0.8	Interphase FISH	WI2-1991P06	chr1:147119447-147157022	WI2-1864C10	chr1:147877335-147920034	WI2-3559A01	chr1:145694865-145735595
2q11.2	chr2:99548000-102250000	2.7	Metaphase FISH	CH251-302J20	chr2:99533973-99708550	CH251-485H3	chr2:101788610-101967530		
2q12.2-q13	chr2:106500000-109110711	2.6	Interphase FISH	RP11-642E23	chr2:107623070-107790013	RP11-465O13	chr2:108316738-108461239	RP11-884F5	chr2:108810690-108952704
			Interphase FISH	RP11-519H15	chr2:106864449-107052396	RP11-642E23	chr2:107623070-107790013	RP11-798K13	chr2:105993683-106171495
			Interphase FISH	RP11-519H15	chr2:106864449-107052396	RP11-884F5	chr2:108810690-108952704	RP11-707I7	chr2:109520258-109693005
4p16.1	chr4:4247000-8757000	4.5	Metaphase FISH	WI2-0485P10	chr4:4748998-4791735	WI2-2655L19	chr4:8226254-8265237		
chr5_inv2	chr5:99582578-100374690	0.8	Interphase FISH	RP11-467C9	chr5:100165949-100300209	RP11-350L5	chr5:99590473-99730988	RP11-368A20	chr5:100723546-100904478
chr7_inv5	chr7:39545072-43961659	4.4	Metaphase FISH	RP11-321C5	chr7:39703279-39863591	RP11-643N15	chr7:43296178-43507286	RP11-1152C21	chr7:35647439-35803586
chr7_inv6	chr7:71693970-74869950	3.2	Interphase FISH	RP11-460F3	chr7:71906422-72099037	RP11-351B3	chr7:74291333-74485290	WI2-3210F8	chr7:71630295-71670184
chr7_inv9	chr7:5997690-6732324	0.7	Interphase FISH	RP11-805P12	chr7:6032671-6213301	RP11-978D4	chr7:6584576-6779703	RP11-1061P7	chr7:7037263-7221665
chr7_inv10	chr7:53188941-53862225	0.7	Interphase FISH	RP11-1056A8	chr7:53219276-53393831	RP11-775N3	chr7:53698860-53847188	RP11-478G18	chr7:54169444-54328931
Strand-seq_chr7_inv4a	chr7:67264518-71693970	4.4	Interphase FISH	RP11-118D11	chr7:67413331-67571355	WI2-3210F8	chr7:71630296-71670184	RP11-351B3/RP11-460F3	chr7:74291334-74485290/chr7:71906422-72099037
Strand-seq_chr7_inv4b	chr7:75634093-77002296	1.4	Interphase FISH	RP11-845K6	chr7:75588268-75772261	RP11-951G4	chr7:76139940-76340373	RP11-378A14	chr7:77311288-77485298
chr8_inv2	chr8:8242347-12174746	3.9	Metaphase FISH	WI2-0785E15	chr8:8344222-8387247	WI2-3642O12	chr8:11391604-11435344		
Strand-seq_chr10_inv2a	chr10:38982495-42370343	3.4	Metaphase and Interphase FISH	RP11-951A24	chr10 38995549 39175661	RP11-419K10	chr10 42990376 43181269	RP11-359B21	chr10 37823956 37981389
chr10_inv6	chr10:46870207-47457081	0.6	Interphase FISH	WI2-1893P04	chr10:47015953-47060596	WI2-3172G20	chr10:47386006-47427580	WI2-2905C01	chr10:48215634-48255065
13q14.13	chr13:45376000-46463000	1	Interphase FISH	RP11-947C16	chr13:45443682-45629849	RP11-179M2	chr13:46009783-46189439	RP11-1148O18	chr13:44852639-45026480
chr15_inv1	chr15:28852754-30406229	1.6	Interphase FISH	WI2-1722N20	chr15:28921466-28964295	RP11-300A12	chr15:29494056-29668994	RP11-640H21	chr15:27894428-28091240
15q25.2	chr15:82300000-84500000	2.2	Interphase FISH	CH251-511D5	chr15:82584677-82755246	CH251-66E11	chr15:83237838-83435975	CH251-321P13	chr15:81962155-82119471
chr16_inv1	chr16:70075634-74327699	4.3	Interphase FISH	WI2-2368K2	chr16:70302736-70337924	WI2-1279O11	chr16:73674254-73714730	RP11-779G13	chr16:69651397-69803431
chr16_inv3	chr16:28781227-30210335	1.4							
chr16_inv8	chr16:29640910-30210335	0.6	Interphase FISH	WI2-2372K22	chr16:29640341-29683607	WI2-0475F01	chr16:30118974-30154488	WI2-0669B08	chr16:30580940-30619130
Strand-seq_chr17_inv2	chr17:16717000-20564685	3.8	Metaphase FISH	RP11-356G14	chr17:17016660-17203604	RP11-732I21	chr17:20125869-20307735		
chr19_inv2	chr19:36331795-37251831	0.9	Interphase FISH	RP11-1148D20	chr19:36359500-36519499	RP11-1088H16	chr19:37017955-37186306	RP11-587I9	chr19:37699822-37923408

2.6 Mhudiblu assembly versus other great apes

Comparison among our Mhudiblu_PPA_v0 and the most recently assembled great ape genomes was performed by retrieving all statistical data from the NCBI assembly (**Supplementary Data Table S7**). ScaffoldN50 and the number of contigs clearly show a relevant difference between the old bonobo release and Mhudiblu_PPA_v0 and show the high quality of the new genome comparable to the other available great ape genomes (**Supplementary Data Fig. S2**).

Supplementary Data Table S7. Comparative analysis on genome assemblies

	panpan1.1	Mhudiblu_PPA_v0	Clint_PTRv2	Kamilah_GGO_v0	GRCh38.p13
**Total sequence length**	3,286,643,938	3,051,901,337	3,050,398,082	3,044,872,214	3,099,706,404
**Total ungapped length**	2,725,937,204	3,015,350,297	3,018,592,990	2,999,027,915	2,948,583,725
**Gaps between scaffolds**	734	64	86	220	349
**Number of scaffolds**	10,984	4,357	4,432	5,706	472
**Scaffold N50**	8,197,324	68,246,502	53,103,722	26,116,462	67,794,873
**Scaffold L50**	94	16	19	35	16
**Number of contigs**	121,356	4,976	5,061	6,345	998
**Contig N50**	66,676	16,579,680	12,268,567	9,522,971	57,879,411
**Contig L50**	11,048	48	67	74	18
**Number of component sequences**	121,337	4,976	5,254	6,345	35,613

Data collected from the most recent great ape assemblies on NCBI assembly.

a

b

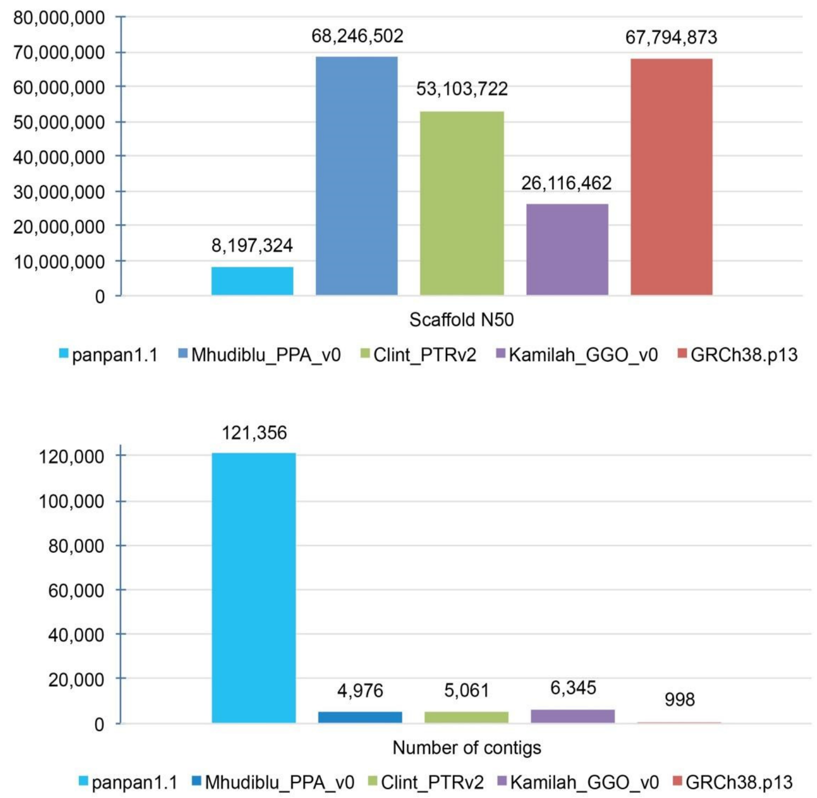

Supplementary Data Figure S2. Comparison among great ape genomes. a, Scaffold N50 and **b,** number of contigs reported**.**

3. Assembly quality and accuracy analyses

3.1 Contiguity assessment using chimpanzee BAC-end sequence

Since the bonobo genome is largely syntenic to chimpanzee (*Pan troglodytes*), we established a lower bound of contiguity by first mapping Sanger-sequenced BES to Mhudiblu_PPA_v0 contigs. We mapped 86,476 available paired-end BES from the Clint chimpanzee BAC library (CHORI-251) to the bonobo assembly. For the assayable regions of the assembly (2.86 Gbp with BES mappings), the aligned high-quality BES data (2.59 Gbp of Sanger PHRED>40) from CHORI-251 showed that 93.42% of the bonobo assembly was concordantly spanned by chimpanzee BES. A similar analysis of the chimpanzee genome assembly (Clint_PTRv2, after using Bionano Genomics data to cut some contigs but before scaffolding into chromosomes) using CHORI-251 showed 94.25% concordance (**Supplementary Data Table S8**).

Supplementary Data Table S8. Bonobo and chimpanzee assembly concordance of BAC end sequence mappings

**Assembly feature**	**Bonobo**	**Chimpanzee**
Total bases assessed for concordance*	2,783,477,718	2,792,082,718
Bases spanned by concordant best*	2,600,560,867	2,631,646,902
Proportion of bases spanned by concordant best	93.42%	94.25%

Clint BAC end sequences (CHORI-251) mapped against bonobo Mhudiblu_PPA_v0 contig assembly and against Clint_PTRv2 after using Bionano data to cut some contigs but before scaffolding into chromosomes. *Contigs greater than 300 kbp.

3.2 Scaffolding and contiguity assessment using Strand-seq

We applied Strand-seq[20,21] in order to assign each contig/scaffold into unique groups corresponding to individual chromosomal homologues using SaaRclust[22,23]. Due to CITES (Convention on International Trade in Endangered Species) restrictions on the transport of bonobo cell lines between laboratories, we generated Strand-seq data from a different bonobo individual (Ulindi)[12].

In order to recluster all scaffolds into the Strand-seq–based whole-chromosome scaffolds, we first aligned available Strand-seq data (generated from Ulindi) to the Mhudiblu assembly (Mhudiblu_PPA_v0) using the BWA aligner (version 0.7.17-r1188) with default parameters for paired-end mapping. Subsequently, we used sambamba (version 0.6.8) in order to mark duplicated reads and SAMtools (version 1.9) to sort and index the final BAM file for each Strand-seq library. Next, we used SaaRclust function ‘scaffoldDenovoAssembly’ on such BAM files using the following parameters: bin.size = 200000, step.size = 200000, prob.th=0.25, bin.method = 'dynamic', min.contig.size = 100000, min.region.to.order = 500000, ord.method = 'greedy', num.clusters = 150, remove.always.WC = TRUE, desired.num.clusters = 25. To provide an extra validation on detected misassemblies, we ran breakpointR[24] on the same BAM files using the following parameters: windowsize = 500000, binMethod = 'size', pairedEndReads = TRUE, pair2frgm = FALSE, chromosomes = [scaffolds >= 1Mb], min.mapq = 10, filtAlt =

TRUE, background = 0.1, minReads = 50. Misassemblies are visible as recurrent changes in strand state across multiple Strand-seq libraries (**Supplementary Data Fig. S3**). To further detect and validate misoriented regions, we created a 'composite file' that groups directional reads across all available Strand-seq libraries using the breakpointR function 'synchronizeReadDir'[21,25]. Next we used the breakpointR function 'runBreakpointr' to detect regions that are homozygous ('ww'; 'HOM') or heterozygous inverted ('wc', 'HET')[24] using following parameters: bamfile = <composite_file>, pairedEndReads = FALSE, chromosomes = [scaffolds >= 1Mb], windowsize = 50000, binMethod = "size", background = 0.1, minReads = 50, genoT = 'binom'. In order to obtain the best possible breakpoint of predicted misassemblies, we used the primatR[18] function 'refineBreakpoints' to refine each detected breakpoint to a narrow interval where strand state changes across multiple Strand-seq libraries, (used parameters: lookup.bp = 500000, pairedEndReads = TRUE, min.mapq = 10, genot.region.ends = TRUE).

Using the above-mentioned procedures, we were able to assign each contig/scaffold to a unique chromosome cluster representative of the bonobo species in comparison to GRCh38 (**Supplementary Data Fig. S4, left**). The procedure correctly clusters chromosome 2A and 2B as shown by two color clusters mapped to chromosome 2 in comparison to GRCh38. In addition, we assigned extra genomic regions, missing in the primary assembly (Mhudiblu_PPA_v0), represented by unassigned scaffolds (**Supplementary Data Fig. S4, right,** e.g., colored in orange for chromosomes 2, 4, 16, and X). In total, we identified an additional 298 of such previously unassigned scaffolds (corresponding to ~96 Mbp of sequence) to chromosomal clusters (as reported by SaaRclust) and used Strand-seq/SaaRclust to aid in predicting a directionality and relative position of these unassigned scaffolds (**Supplementary Data Fig. S5**; **Supplementary Data Fig. S6**). Of those, orthogonal data supported the placement, order, and orientation of 36 Mbp of sequence from 61 contigs from 56 scaffolds onto the ordered and oriented chromosomes (**Supplementary Table 33**) and 12.5 Mbp from 108 scaffolds (125 contigs) on unlocalized sequences for specific chromosomes in Mhudiblu_PPA_v1 (**Supplementary Data Table S9**; **section 4.4**). Proper scaffold orientations are confirmed by known large-scale inversions as shown by mapping bonobo scaffolds to GRCh38 (**Supplementary Data Fig. S6a**). Observed inverted regions are also supported by Strand-seq read directionality as compared to GRCh38 (**Supplementary Data Fig. S6b**).

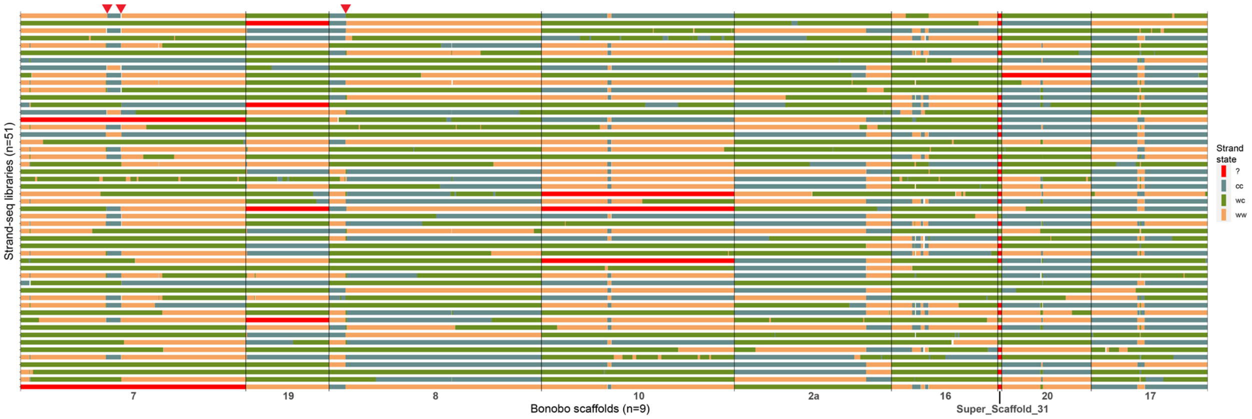

Supplementary Data Figure S3. Recurrent strand state changes in putative assembly errors (n = 9). Regions separated by vertical black lines represent individual scaffolds that contain putative assembly errors while each row represents a single Strand-seq library (n = 51). Horizontal bars along each row are colored based on the three possible strand states (WW - only Watson reads; CC - only Crick reads; WC mixture of Watson and Crick reads mapped in a given region). Red bars represent regions where genotyping could not be reliably determined. Red arrowheads on top of the tile plot show a few examples of recurrent strand state changes that are indicative of an assembly error.

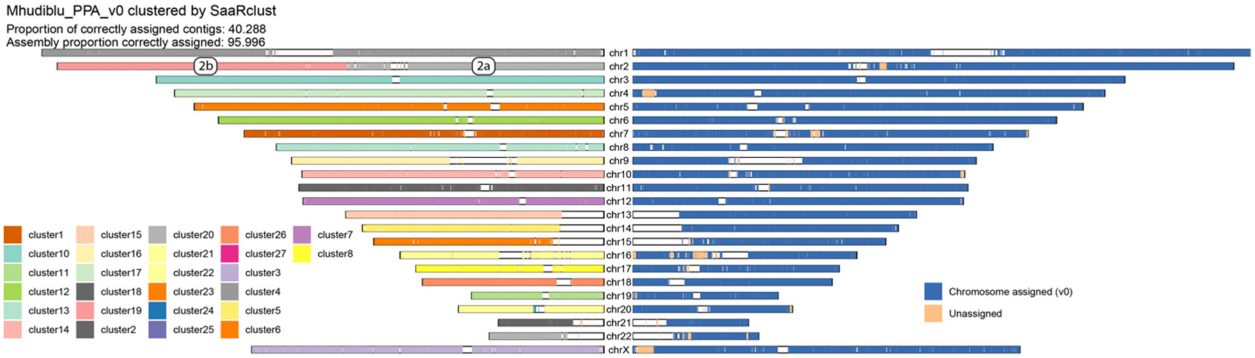

Supplementary Data Figure S4. Clustering of bonobo contigs/scaffolds into chromosomes. LEFT: Each scaffolded genomic region represents a range defined by mapping coordinates on GRCh38. Such genomic regions are then colored based on cluster identity determined by SaaRclust. In an ideal scenario there is a single color for each chromosome. RIGHT: Genomic regions assigned to full chromosomal scaffolds are colored blue while regions additionally assigned to chromosomal scaffolds using Strand-seq are colored orange.

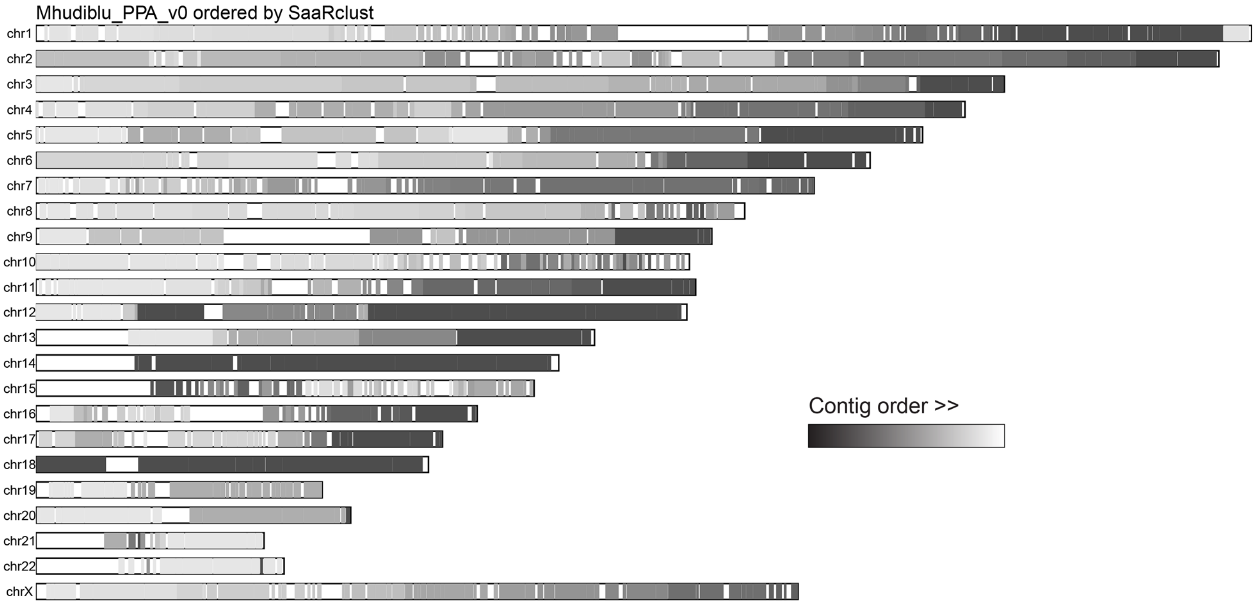

Supplementary Data Figure S5. Prediction of order of unassigned contigs within original scaffolds. Each scaffold is plotted as a rectangle based on the mapping to GRCh38. Each scaffold is colored based on the predicted order within each chromosomal cluster, which is reflected by the shades of gray going from dark to light gray. Ideally we observe colors going always from dark to light gray or vice versa and thus being in agreement with scaffold order with respect to GRCh38.

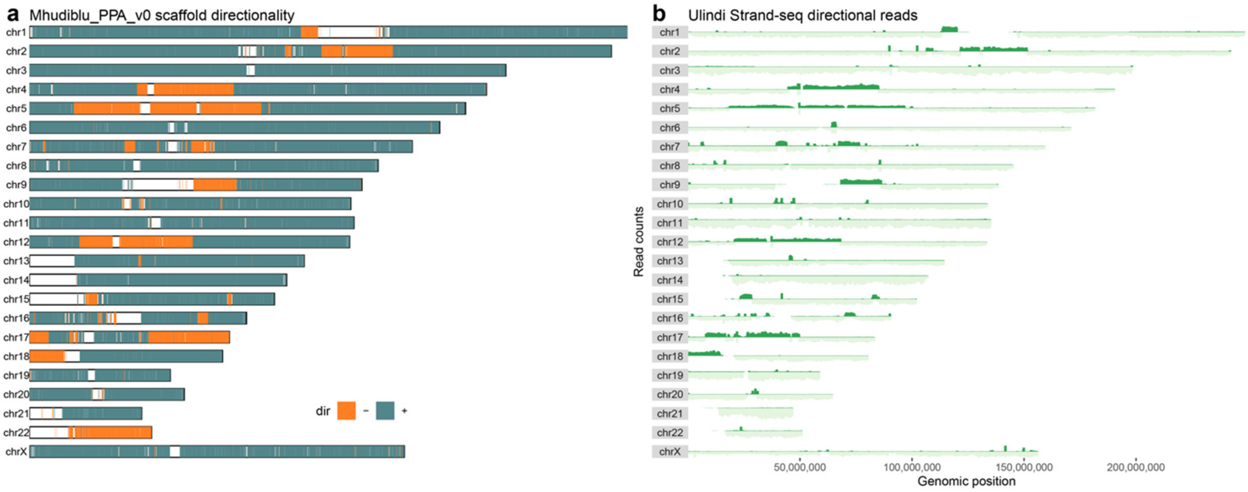

Supplementary Data Figure S6. Assignment of proper orientation to bonobo scaffolds. a, Each scaffolded genomic region represents a range based on mapping coordinates on GRCh38. Each genomic range is colored based on the directionality ('+' positive strand, '-' negative strand) it maps to GRCh38. **b,** Strand-seq directional reads have been binned into 200 kbp bins and the number of reads mapped in forward (reference orientation - light color) and reverse (inverted orientation - dark color) orientation to GRCh38 are depicted as a length of a bar along each chromosome.

Supplementary Data Table S9. Scaffolds and contigs assigned to chr*_random

chr	Scaffold	Contig	Length
chr1	001257F_83971_qpd_scaf	001257F_83971_qpd	84011
chr1	001774F_57796_qpd_scaf	001774F_57796_qpd	57826
chr1	001782F_57334_qpd_scaf	001782F_57334_qpd	57469
chr1	001919F_52499_qpd_scaf	001919F_52499_qpd	52494
chr1	002185F_44506_qpd_scaf	002185F_44506_qpd	44463
chr1	002447F_38334_qpd_scaf	002447F_38334_qpd	38395
chr1	003957F_13054_qpd_scaf	003957F_13054_qpd	13130
chr10	000650F_193269_qpd_scaf	000650F_193269_qpd	192753
chr10	001129F_98482_qpd_scaf	001129F_98482_qpd	97937
chr10	001315F_81000_qpd_scaf	001315F_81000_qpd	81227
chr10	001467F_75244_qpd_scaf	001467F_75244_qpd	75240
chr10	001732F_59636_qpd_scaf	001732F_59636_qpd	59768
chr10	001837F_55529_qpd_scaf	001837F_55529_qpd	55543
chr10	002023F_48974_qpd_scaf	002023F_48974_qpd	48502
chr10	002112F_46267_qpd_scaf	002112F_46267_qpd	46359
chr10	002804F_31894_qpd_scaf	002804F_31894_qpd	32035
chr10	003378F_22085_qpd_scaf	003378F_22085_qpd	22075
chr10	003471F_20629_qpd_scaf	003471F_20629_qpd	20643
chr11	000061F_40364_qpd_scaf	000061F_40364_qpd	40600
chr11	001182F_93050_qpd_scaf	001182F_93050_qpd	92931
chr11	002358F_40047_qpd_scaf	002358F_40047_qpd	40011
chr11	002897F_30202_qpd_scaf	002897F_30202_qpd	30163
chr11	003907F_13652_qpd_scaf	003907F_13652_qpd	13691
chr12	004317F_7585_qpd_scaf	004317F_7585_qpd	7655
chr13	4776_72175_qpd_scaf	4776_72175_qpd	72155
chr14	002335F_40593_qpd_scaf	002335F_40593_qpd	40671
chr15	004073F_11515_qpd_scaf	004073F_11515_qpd	11492
chr15	4772_49244_qpd_scaf	4772_49244_qpd	49257
chr16	001553F_66747_qpd_scaf	001553F_66747_qpd	66887
chr16	001662F_62537_qpd_scaf	001662F_62537_qpd	61525
chr16	001745F_59063_qpd_scaf	001745F_59063_qpd	59107
chr16	002118F_46099_qpd_scaf	002118F_46099_qpd	46129
chr16	002143F_45431_qpd_scaf	002143F_45431_qpd	45576
chr16	004398F_6372_qpd_scaf	004398F_6372_qpd	6439
chr16	Super_Scaffold_103	000697F_176032_qpd	176713
chr16	Super_Scaffold_103	001516F_68765_qpd	68919
chr16	Super_Scaffold_103	000831F_111941_qpd	111912
chr16	Super_Scaffold_103	000367F_534804_qpd	535922
chr16	Super_Scaffold_200000111530	001007F_92105_qpd	92139
chr16	Super_Scaffold_200000111530	000554F_250573_qpd	251472
chr16	Super_Scaffold_200000128750	000473F_318250_qpd	319313
chr16	Super_Scaffold_200000128750	000579F_238345_qpd	237397
chr16	Super_Scaffold_31	000327F_636639_qpd	638128
chr16	Super_Scaffold_31	000816F_146127_qpd	146498
chr16	Super_Scaffold_31	000545F_257181_qpd	257550
chr16	Super_Scaffold_31	000335F_635813_qpd	637846
chr16	Super_Scaffold_31	000425F_397723_qpd	398099
chr16	Super_Scaffold_31	000807F_147450_qpd	147899
chr16	Super_Scaffold_85	000514F_275345_qpd	274918
chr17	000247F_1669657_qpds_283170_293160_scaf	000247F_1669657_qpds_283170_293160	9991
chr17	000252F_1459253_qpds_570022_596419_scaf	000252F_1459253_qpds_570022_596419	26398
chr17	000627F_206041_qpd_scaf	000627F_206041_qpd	206578
chr17	000915F_125549_qpd_scaf	000915F_125549_qpd	125816
chr17	001404F_75423_qpd_scaf	001404F_75423_qpd	75572
chr17	001427F_74019_qpd_scaf	001427F_74019_qpd	74016
chr17	001433F_73616_qpd_scaf	001433F_73616_qpd	73917
chr17	001593F_64894_qpd_scaf	001593F_64894_qpd	64754
chr17	001730F_59677_qpd_scaf	001730F_59677_qpd	59873
chr17	001999F_49501_qpd_scaf	001999F_49501_qpd	50053
chr17	003478F_20535_qpd_scaf	003478F_20535_qpd	19870
chr17	Super_Scaffold_200000608	000747F_179840_qpd	180413
chr17	Super_Scaffold_200000608	000607F_209608_qpd	209160

chr18	001515F_68868_qpd_scaf	001515F_68868_qpd	69051
chr18	004369F_6738_qpd_scaf	004369F_6738_qpd	6796
chr18	004471F_5174_qpd_scaf	004471F_5174_qpd	5151
chr18	4853_26985_qpd_scaf	4853_26985_qpd	27045
chr19	001562F_41893_qpd_scaf	001562F_41893_qpd	42022
chr19	002432F_38641_qpd_scaf	002432F_38641_qpd	38110
chr19	003155F_25892_qpd_scaf	003155F_25892_qpd	25849
chr19	004286F_7935_qpd_scaf	004286F_7935_qpd	7926
chr20	000047F_41273_qpd_scaf	000047F_41273_qpd	41190
chr20	000812F_136366_qpd_scaf	000812F_136366_qpd	136094
chr20	001623F_63985_qpd_scaf	001623F_63985_qpd	63951
chr20	002921F_29825_qpd_scaf	002921F_29825_qpd	29797
chr20	Super_Scaffold_100000557	000875F_137984_qpd	138522
chr20	Super_Scaffold_100000557	000556F_247874_qpds_1_140479	140479
chr22	000183F_3473307_qpds_2999505_3017434_scaf	000183F_3473307_qpds_2999505_3017434	17930
chr22	000757F_158789_qpd_scaf	000757F_158789_qpd	158647
chr22	000769F_156510_qpd_scaf	000769F_156510_qpd	155723
chr22	Super_Scaffold_69	000391F_467569_qpd	467996
chr22	Super_Scaffold_69	002022F_48999_qpd	48988
chr3	000016F_64668_qpd_scaf	000016F_64668_qpd	64613
chr3	000064F_53603_qpd_scaf	000064F_53603_qpd	53560
chr3	000421F_408941_qpd_scaf	000421F_408941_qpd	410052
chr4	000068F_50224_qpd_scaf	000068F_50224_qpd	50159
chr4	002753F_32937_qpd_scaf	002753F_32937_qpd	32991
chr4	003283F_23829_qpd_scaf	003283F_23829_qpd	23858
chr5	001448F_72668_qpd_scaf	001448F_72668_qpd	72969
chr5	001902F_52948_qpd_scaf	001902F_52948_qpd	52965
chr6	000380F_487661_qpd_scaf	000380F_487661_qpd	489234
chr6	000461F_122836_qpd_scaf	000461F_122836_qpd	123501
chr6	001289F_76169_qpd_scaf	001289F_76169_qpd	77230
chr6	001787F_57159_qpd_scaf	001787F_57159_qpd	57142
chr6	002313F_41069_qpd_scaf	002313F_41069_qpd	41085
chr6	002670F_34490_qpd_scaf	002670F_34490_qpd	34533
chr6	003229F_24680_qpd_scaf	003229F_24680_qpd	23659
chr6	003249F_24277_qpd_scaf	003249F_24277_qpd	24290
chr6	004323F_7399_qpd_scaf	004323F_7399_qpd	7400
chr6	004373F_6657_qpd_scaf	004373F_6657_qpd	6659
chr7	000161F_18856_qpd_scaf	000161F_18856_qpd	18918
chr7	001865F_54049_qpd_scaf	001865F_54049_qpd	54053
chr7	002169F_44800_qpd_scaf	002169F_44800_qpd	44990
chr7	002217F_43869_qpd_scaf	002217F_43869_qpd	43972
chr7	002374F_39707_qpd_scaf	002374F_39707_qpd	39649
chr7	002547F_36551_qpd_scaf	002547F_36551_qpd	36598
chr7	002741F_33146_qpd_scaf	002741F_33146_qpd	33160
chr7	004114F_10916_qpd_scaf	004114F_10916_qpd	10923
chr8	002669F_34509_qpd_scaf	002669F_34509_qpd	34556
chr8	003024F_9210_qpd_scaf	003024F_9210_qpd	9222
chr8	003263F_24166_qpd_scaf	003263F_24166_qpd	24217
chr8	003552F_19048_qpd_scaf	003552F_19048_qpd	19114
chr9	000291F_932118_qpds_895609_933459_scaf	000291F_932118_qpds_895609_933459	37851
chr9	001580F_65381_qpd_scaf	001580F_65381_qpd	65502
chr9	Super_Scaffold_100000797	000796F_149964_qpd	149259
chr9	Super_Scaffold_66	000450F_342920_qpds_1_187399	187399
chr9	Super_Scaffold_66	000553F_251115_qpd	252554
chr9	Super_Scaffold_66	000793F_151710_qpd	151981
chr9	Super_Scaffold_66	000678F_170635_qpd	171746
chr9	Super_Scaffold_66	000448F_345655_qpd	346654
chrX	001713F_60256_qpd_scaf	001713F_60256_qpd	60345
chrX	001939F_51787_qpd_scaf	001939F_51787_qpd	51783
chrX	002697F_33994_qpd_scaf	002697F_33994_qpd	33976
chrX	003248F_24303_qpd_scaf	003248F_24303_qpd	24292
chrX	004311F_7630_qpd_scaf	004311F_7630_qpd	7620
chrX	4865_42434_qpd_scaf	4865_42434_qpd	42581

SaaRclust is also able to report potential assembly errors[26]. Initially we identified 24 putative genome assembly errors (**Supplementary Data Table S10**) distributed among nine different scaffolds (Mhudiblu_PPA_v0) (**Supplementary Data Table S11**) that were confirmed by the recurrent change in Strand-seq strand state (**Supplementary Data Fig. S3**). To ensure the highest quality of our assembly, we sought to identify the full spectrum of regions that were homozygous inverted and thus represented either incorrectly oriented genomic segments or unresolved homozygous inversions[23]. In total, we identified 29 such regions (**Supplementary Data Fig. S7**; **Supplementary Data Table S12**). All confirmed assembly errors have been corrected in assembly version 1 (**Supplementary Data Table S13**; **Supplementary Data Fig. S8**; **section 4.4).**

Supplementary Data Table S10. Breakpoints of putative assembly errors

seqnames	start	end	genoT	start.CI	end.CI	break.ID	Valid
**chr7**	6135178	6145106	cc-ww	6110220	6155143	chr7:6016360-6016361	TRUE
**chr7**	6605801	6607404	ww-wc	6574649	6613487	chr7:6765137-6765138	TRUE
**chr7**	6680646	6684023	wc-cc	6657632	6688533	chr7:6765137-6765138	FALSE
**chr7**	57487299	57493388	wc-cc	57468518	57499059	chr7:57483782-57483783	FALSE
**chr7**	68062678	68063927	cc-wc	68043788	68075144	chr7:67726915-67726916	FALSE
**chr19**	649981	649989	cc-ww	646035	676078	chr19:588223-588224	TRUE
**chr8**	11869486	11871780	wc-cc	11868250	11874245	chr8:11698272-11698273	FALSE
**chr10**	44083746	44083845	cc-wc	44078955	44094723	chr10:44052290-44052291	FALSE
**chr10**	44199773	44239849	wc-ww	44199500	44257533	chr10:44052290-44052291	TRUE
**chr10**	46791209	46793524	ww-cc	46757444	46803680	chr10:46894351-46894352	TRUE
**chr2a**	87979362	87981295	cc-ww	87973137	87987704	chr2a:88001763-88001764	TRUE
**chr16**	13792488	13804297	ww-cc	13724584	13810830	chr16:13945669-13945670	TRUE
**chr16**	19512748	19513886	ww-wc	19511169	19515001	chr16:19638041-19638042	TRUE
**chr16**	19729331	19729604	wc-cc	19668940	19732026	chr16:19638041-19638042	FALSE
**chr16**	19512748	19513886	ww-wc	19511169	19515001	chr16:22278679-22278680	FALSE
**chr16**	19729331	19729604	wc-cc	19668940	19732026	chr16:22278679-22278680	FALSE
**chr16**	24820872	24821482	ww-wc	24810434	24841126	chr16:25026743-25026744	TRUE
**chr16**	25061826	25061972	wc-cc	25050720	25062687	chr16:25026743-25026744	FALSE
**Super_Scaffold_31**	1669456	1948232	cc-wc	1628252	1949708	Super_Scaffold_31:1960381-1960382	TRUE
**chr20**	25996950	26000412	cc-wc	25974254	26073791	chr20:26273621-26273622	FALSE
**chr20**	27533657	27533723	wc-cc	27533436	27534760	chr20:27738658-27738659	TRUE
**chr17**	30727005	30730805	ww-cc	30726443	30771051	chr17:30985666-30985667	TRUE
**chr17**	35630691	35647492	ww-wc	35629616	35652745	chr17:36028887-36028888	TRUE
**chr17**	35823538	35861727	wc-cc	35814183	35876451	chr17:36028887-36028888	FALSE

Supplementary Data Table S11. Putative assembly errors in bonobo assembly

scaffold	scaffold length	error type	putative misassembled base count
**chr7**	150536359	misorient	10991910
**chr19**	55604062	misorient	588223
**chr8**	141842281	misorient	11698272
**chr10**	128853861	misorient	2842061
**chr2a**	104947789	misorient	16946026
**chr16**	71000456	misorient	8440436
**Super_Scaffold_31**	2667427	misorient	707046
**chr20**	59769695	chimerism	1465037
**chr17**	77747126	misorient	5043221

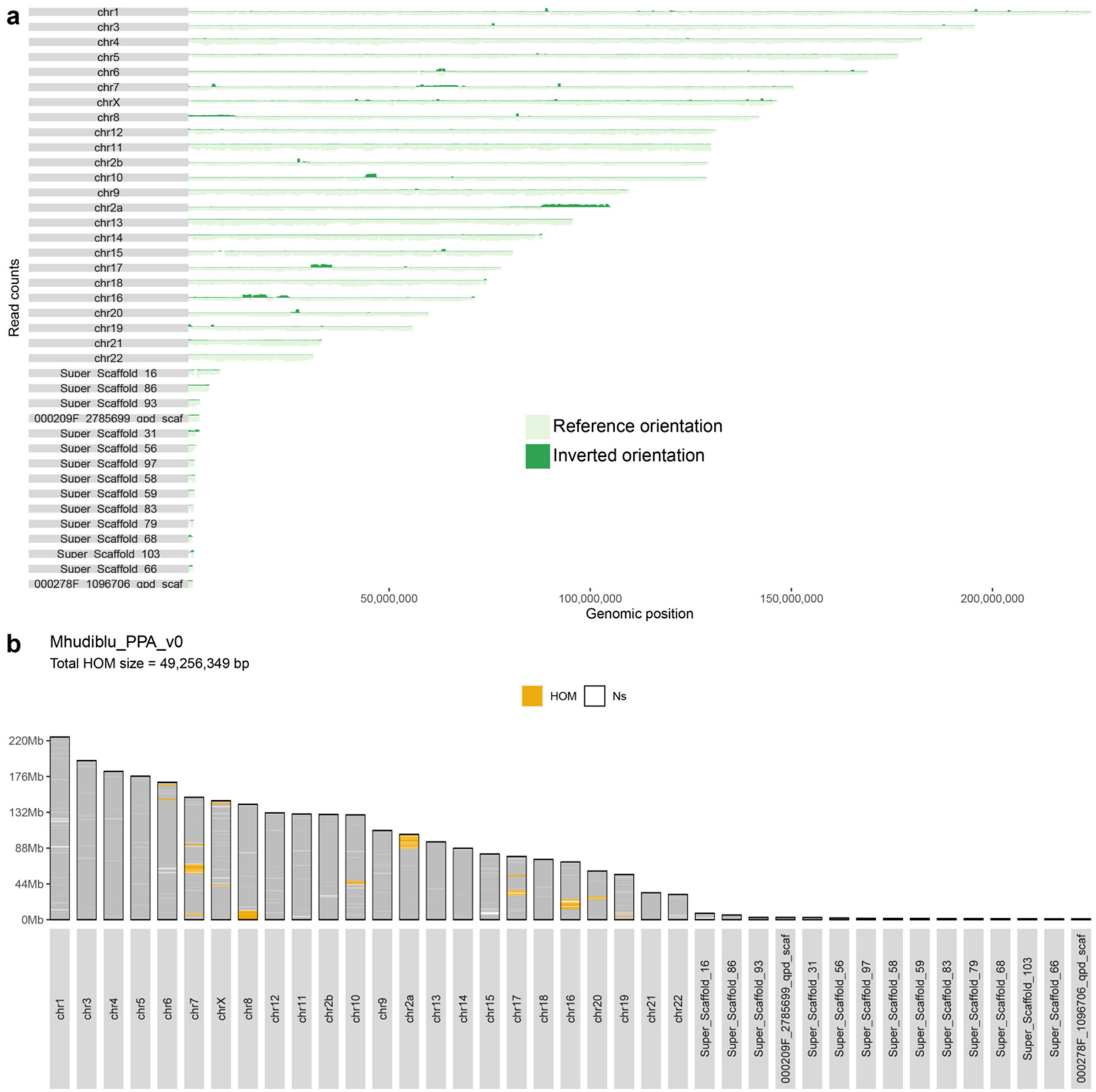

Supplementary Data Figure S7. Homozygous inverted regions in bonobo assembly (Mhudiblu_PPA_v0). a, Strand-seq directional reads have been binned into 200 kbp bins and the number of reads mapped in forward (reference orientation - light color) and reverse (inverted orientation - dark color) orientation to Mhudiblu_PPA_v0 are depicted as a length of a bar along each chromosome. **b,** An ideogram in which regions possessing only inverted reads across all Strand-seq libraries are genotyped as homozygous inverted ('HOM' - orange). Regions of continuous stretches of N's (assembly gaps) are colored in white.

Supplementary Data Table S12. Homozygous switches in Strand-seq read directionality for Mhudlidbu_PPA_v0 (n=29)

seqnames	start	end	width	Ws	Cs	states	ID
chr10	44233998	46778577	2544580	10403	418	ww	Mhudiblu_PPA_v0
chr16	13802968	15353748	1550781	3234	236	ww	Mhudiblu_PPA_v0
chr16	16298346	19512747	3214402	6155	479	ww	Mhudiblu_PPA_v0
chr16	21221215	24628764	3407550	2931	352	ww	Mhudiblu_PPA_v0
chr17	30730806	32421140	1690335	4590	117	ww	Mhudiblu_PPA_v0
chr17	33246164	35631809	2385646	7286	301	ww	Mhudiblu_PPA_v0
chr17	54096373	54170503	74131	269	15	ww	Mhudiblu_PPA_v0
chr19	1	649980	649980	981	52	ww	Mhudiblu_PPA_v0
chr19	6055158	6061902	6745	314	38	ww	Mhudiblu_PPA_v0
chr20	25766700	26031490	264791	322	17	ww	Mhudiblu_PPA_v0
chr20	26495082	27003588	508507	665	38	ww	Mhudiblu_PPA_v0
chr20	27369978	27533656	163679	300	11	ww	Mhudiblu_PPA_v0
chr2a	87979863	88420161	440299	823	187	ww	Mhudiblu_PPA_v0
chr2a	88635202	90100087	1464886	3562	747	ww	Mhudiblu_PPA_v0
chr2a	90251823	91322533	1070711	3460	761	ww	Mhudiblu_PPA_v0
chr2a	91460831	99493870	8033040	20711	4525	ww	Mhudiblu_PPA_v0
chr2a	99789393	103344193	3554801	7818	1265	ww	Mhudiblu_PPA_v0
chr2a	103652673	103739765	87093	151	21	ww	Mhudiblu_PPA_v0
chr2a	103901851	104459258	557408	1377	241	ww	Mhudiblu_PPA_v0
chr6	147764184	147817045	52862	152	4	ww	Mhudiblu_PPA_v0
chr6	165113401	165321236	207836	577	21	ww	Mhudiblu_PPA_v0
chr7	6141370	6603158	461789	1292	265	ww	Mhudiblu_PPA_v0
chr7	57495416	60348096	2852681	2690	119	ww	Mhudiblu_PPA_v0
chr7	60834530	67291673	6457144	8287	281	ww	Mhudiblu_PPA_v0
chr7	92336938	92368153	31216	683	65	ww	Mhudiblu_PPA_v0
chr8	251902	7179435	6927534	7944	1759	ww	Mhudiblu_PPA_v0
chr8	7288740	10966706	3677967	5174	921	ww	Mhudiblu_PPA_v0
chrX	41900160	41909790	9631	309	25	ww	Mhudiblu_PPA_v0
chrX	142650915	142823547	172633	472	18	ww	Mhudiblu_PPA_v0

Supplementary Data Table S13. Remaining HOM inversions in Mhudiblu_PPA_v1

seqnames	start	end	width	Ws	Cs	states	ID
chr10	47092930	49623483	2530554	10426	382	ww	Mhudiblu_PPA_v1
chr15	22259734	22344095	84362	127	42	ww	Mhudiblu_PPA_v1
chr17	56095874	56170004	74131	269	15	ww	Mhudiblu_PPA_v1
chr19	6661711	6668473	6763	320	33	ww	Mhudiblu_PPA_v1
chr19	35870817	35873490	2674	119	29	ww	Mhudiblu_PPA_v1
chr20	25785948	25975497	189550	364	96	ww	Mhudiblu_PPA_v1
chr20	29398208	29485132	86925	298	51	ww	Mhudiblu_PPA_v1
chr2A	17862887	18007468	144582	110	14	ww	Mhudiblu_PPA_v1
chr2A	21346946	21657915	310970	960	43	ww	Mhudiblu_PPA_v1
chr6	149764084	149816945	52862	147	4	ww	Mhudiblu_PPA_v1
chr7	98374949	98409334	34386	683	77	ww	Mhudiblu_PPA_v1
chrX	49795486	49805116	9631	288	17	ww	Mhudiblu_PPA_v1
chrX	152546141	152718773	172633	457	9	ww	Mhudiblu_PPA_v1

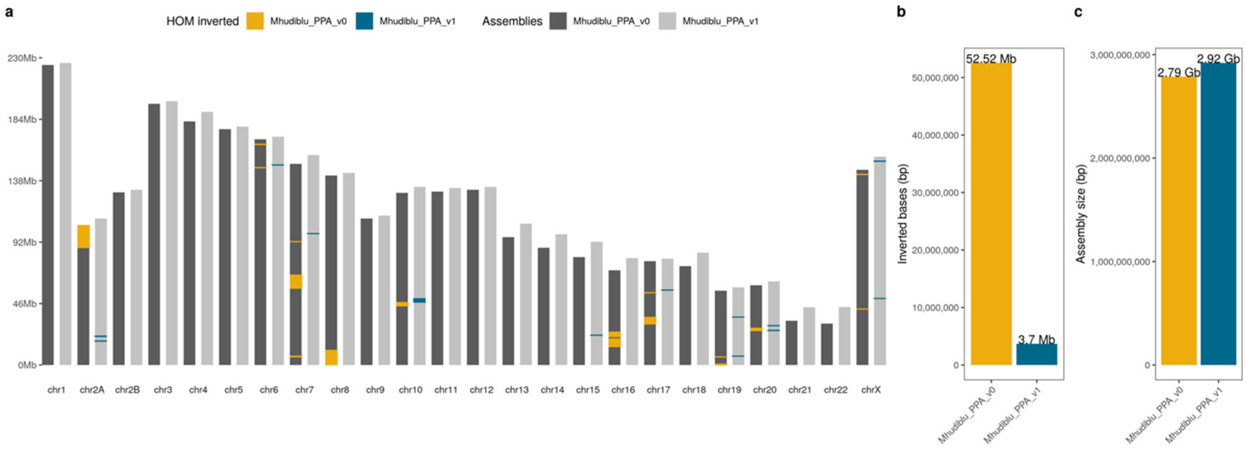

Supplementary Data Figure S8. Comparison of misoriented regions detected in Mhudiblu_PPA_v0 and Mhudiblu_PPA_v1 bonobo assemblies. a, Bonobo Mhudiblu_PPA_v0 assembly plotted as dark gray bars and Mhudiblu_PPA_v1 as light gray bars. Homozygous switches (HOM) in Strand-seq read directionality are highlighted by orange for Mhudiblu_v0 and blue for Mhudiblu_v1. **b,** Total number of Homozygous switches inverted bases in Mhudiblu_v0 (yellow) and Mhudiblu_v1 (blue) assembly. **c,** Total size of the Mhudiblu_v0 (yellow) and Mhudiblu_v1 (blue) assembly. Bonobo Mhudiblu_PPA_v0 assembly is plotted as dark gray bars and Mhudiblu_PPA_v1 as light gray bars. Homozygous switches in Strand-seq read directionality are in yellow for Mhudiblu_v0 and blue for Mhudiblu_v1.

Lastly, we evaluated the inversion status of collapsed regions detected in Mhudiblu_PPA_v0 scaffolds. Of the 718 collapses, we considered only those present in scaffolds ≥1 Mbp (n = 532). Next, we genotyped only collapses that had at least 100 Strand-seq reads mapped to them (n = 114). We found that the majority (n = 76) of these collapses were genotyped as heterozygous, meaning that at least one copy of the ancestral locus resides in the genome in an inverted orientation. The remaining 38 collapses were genotyped as either homozygous reference (n = 35) or homozygous inverted (n = 3), meaning that both the ancestral and duplicated copy have the same directionality (**Supplementary Data Fig. S9**). Size distribution of these regions

suggests that regions with at least one inverted copy tend to be larger than directly oriented duplications (**Supplementary Data Fig. S9**).

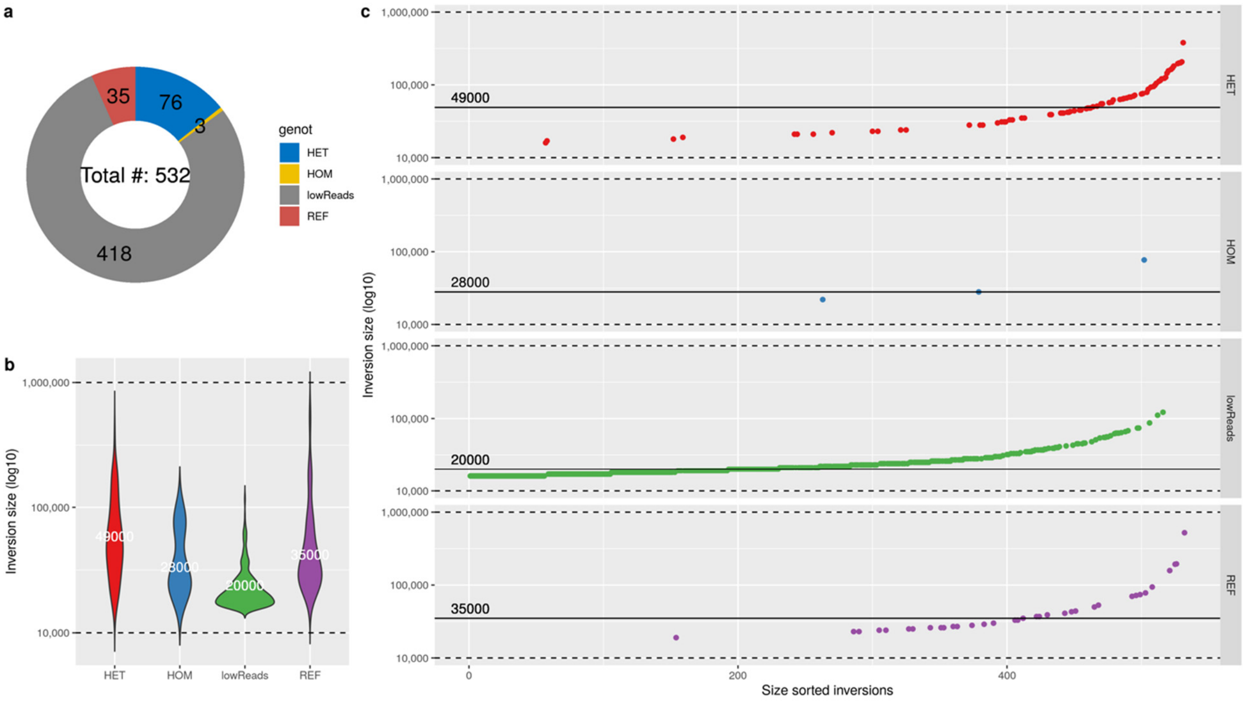

Supplementary Data Figure S9. Genotypes and size distribution for collapsed regions (n = 532). a, A donut plot shows 532 total collapsed regions that are mappable for short Strand-seq reads. We report Strand-seq genotype as: REF - homozygous reference orientation, HOM - homozygous inverted orientation, HET - at least one copy of the region in an inverted orientation, or lowReads - if a region contains less than 100 Strand-seq reads. **b,** & **c,** Size distribution of genotyped collapses represented either as a violin plot (b) or a scatter plot (c). Median inversion size is marked in the middle of each violin as well as a solid line in (c).

3.3 Illumina-based sequence accuracy

We assessed the base-level accuracy of the Mhudiblu_PPA_v0 assembly by applying Merqury[27] to Illumina WGS data from Mhudiblu. The method compares 21 bp k-mers in the Mhudiblu assembly to those present in unassembled Illumina reads; 21 bp k-mers present in the assembly but not in the Illumina reads are considered to contain errors while k-mers found in both the assembly and the short reads are considered valid. Based on this comparison, we estimated an overall sequence accuracy of the Mhudiblu assembly of QV 39, equivalent to 99.99% base call accuracy (**Supplementary Data Fig. S10**).

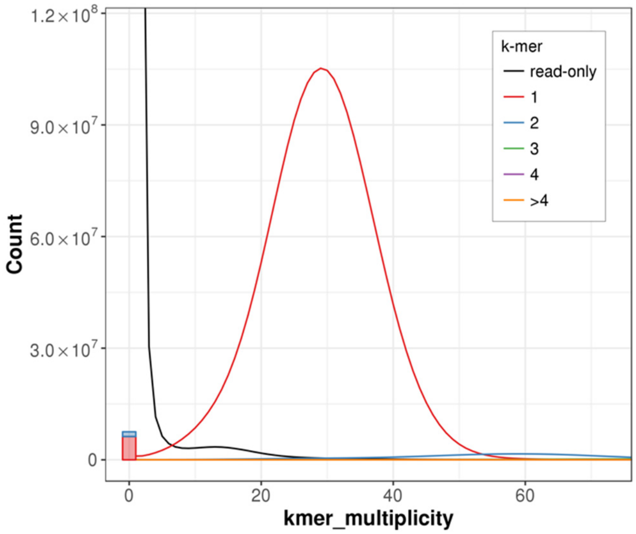

Supplementary Data Figure S10. Merqury k-mer distribution of bonobo assembly. Merqury was run on bonobo genome assembly Mhudiblu_PPA_v0 with the Illumina reads used to polish the assembly. The number of distinct Illumina k-mers ("Count") is compared against its occurrence in Illumina WGS ("kmer multiplicity"). Colored lines indicate the number of times a k-mer is found within the assembly. The black line indicates k-mers unique to Illumina WGS. The blue and red boxes (at kmer_multiplicity = 0) indicate unique assembly k-mers (UAK) not found in the Illumina reads.

3.4 BAC-based sequence accuracy

We sequenced and assembled 17 large-insert BAC clones selected at random from bonobo library VMRC74 constructed from Mhudiblu and compared them to the genome assembly for sequence accuracy and contiguity. All BACs sequenced were completely contiguous with the genome assembly (**Supplementary Data Table S14**, 0 clipped base pairs). Using this approach, we estimate an overall sequence accuracy of QV 32, although there is considerable variability depending on STR content and homopolymer content of regions (**Supplementary Data Table S14**). Of note, we consider this QV estimate a lower bound because we are sequencing only one of two haplotypes and are not correcting for sequence polymorphisms present in Mhudiblu (as such variants sequence differences would be counted as errors). If we limit our analysis to BACs mapping to autozygous regions of the genome (n = 6), our QV estimate rises to 42 consistent with the Illumina-based estimate (**Supplementary Data Table S14**).

Supplementary Data Table S14. BAC-based accuracy and local contiguity analyses

BAC	Accession number	BAC Length	clipped bases	Bonobo FISH Mapping	matches (bp)	mismatches (bp)	deletions (bp)	insertions (bp)	indels (events)	QV1	QV2	QV3
*VMRC74-123A6	AC280330.1	154448	0	5q/6q	154444	0	5	4	9	42	42	100
*VMRC74-123H1	AC280332.1	74896	0	7q/8q	74882	0	1	14	7	37	40	100
VMRC74-145I3	AC280334.1	148250	0	10q/12q	148180	36	16	34	23	32	34	36
VMRC74-188D5	AC280329.1	88519	0	4p/5p	88436	63	23	20	16	29	30	31
**VMRC74-188E6	AC280343.1	178290	0	21p/20p	178139	108	115	43	27	28	31	32
VMRC74-253A10	AC280335.1	67818	0	9p/11p	67753	43	26	22	16	29	31	32
*VMRC74-293A5	AC280331.1	83150	0	5q/6q	83143	0	0	7	7	41	41	100
VMRC74-373B17	AC280339.1	99620	0	1q/1q	99533	32	10	55	12	30	34	35
VMRC74-380E1	AC280344.1	129368	0	2p/3p	129248	29	34	91	48	29	32	36
*VMRC74-484A9	AC280341.1	101255	0	5q/6q	101254	0	3	1	4	44	44	100
VMRC74-484F2	AC280342.1	94070	0	8q/10q	94014	49	8	7	8	32	32	33
VMRC74-493C24	AC280336.1	108757	0	NA	108732	18	2	7	6	36	37	38
VMRC74-493P1	AC280340.1	147460	0	7p/8p	147372	43	37	45	34	31	33	35
VMRC74-517J3	AC280326.1	99205	0	10q/12q	99162	32	126	11	18	28	33	35
VMRC74-526G4	AC280328.1	76755	0	13q/2B	76710	41	8	4	5	32	32	33
*VMRC74-82C8	AC280338.1	64868	0	13q/2B	64867	0	0	1	1	48	48	100
*VMRC74-82F12	AC280337.1	89144	0	4q/5q	89140	0	0	4	4	43	43	100
total					1805009	494	414	370	245	32	34	36

The number of clipped bases represents the number discontinuities between the BAC and genome alignment. All sequence differences were considered in calculations of genome sequence accuracy.
QV1: considers mismatches. inserted bases. and deleted bases as errors. QV2: considers a string of inserted or deleted bases as a single error. no matter how long. and considers mismatches as errors. QV3: only considers mismatches as errors. The BACs with an asterisk are in regions of Mhudiblu homozygosity (i.e., no allelic variation); considering QV for those six BACs gives significantly higher QV estimates (QV1=42. QV2=42. QV3=100) consistent with Illumina-based accuracy estimates. The BAC with two asterisks has a 15 kbp region of high diversity leading to its high discrepancy count. However. if this BAC were excluded from the QV calculations. it would make a difference of less than 1 in the QV values. FISH mapping has been defined following the classical/McConkey nomenclatures (http://www.biologia.uniba.it/5-bonobo/).

3.5 Bionano Genomics: optical maps and variant calling

We restriction digested and labelled high-molecular weight DNA extracted from the Mhudiblu cell line with Nt.BspQI and Nb.BssSI enzymes. We generated over 100-fold coverage of single-molecule data for each assembly and constructed two *de novo* assemblies. We compared the assemblies against the human reference genome GRCh38 and detected 9,211 insertions, 9,554 deletions, and 285 inversions (of which 13 are >5 Mbp, indicated as translocation_intrachr) (**Supplementary Table 54**). The larger events validated by these optical maps include a 40 Mbp inversion on 4p12-4q21.25, a 47 Mbp inversion on 12p12.2-12q15, 41 Mbp inversion on 17p13.1-17q21.33, and 30 Mbp inversion on 2q14.3-2q23.3.

3.6 Gap analysis and comparison to previous bonobo assembly

We systematically compared the previous bonobo assembly, panpan1.1[28], with Mhudiblu_PPA_v0 for the purpose of gap identification and potential sequence accuracy issues. panpan1.1 chromosomal sequences were segmented into 1 kbp non-overlapping segments and aligned against the Mhudiblu_PPA_v0 assembly using BLAT[29] in client/server mode with default parameters. We processed these alignments to identify those 1 kbp segments that uniquely aligned to the Mhudiblu_PPA_v0 genome. The uniquely aligning segments were then used as anchors to create a single set of consistent alignments along each chromosome. Percent identity was calculated for each 1 kbp segment where at least 500 bases of the segment aligned (**Supplementary Data Fig. S11**) and plotted along the chromosome.
Each panpan1.1 gap (693 contig gaps; 107,361 scaffold gaps) was considered “closed” when a single Mhudiblu_PPA_v0 scaffold spanned the panpan1.1 gap. When both of the 1 kbp segments neighboring a scaffold/contig gap were aligned contiguously within the genome and the estimated gap size was within 10,000 bases of the gap size estimated in the panpan1.1 assembly, the corresponding Mhudiblu_PPA_v0 segment was defined as the region within the panpan1.1 gap. Repeat content of the Mhudiblu_PPA_v0 segments was obtained by using the Mhudiblu_PPA_v0 RepeatMasker 3.3.0 (library Dfam3.1) analysis. Coordinates of full-length L1s were also compared with the Mhudiblu_PPA_v0 gap-spanning coordinates to identify those full-length L1s that overlapped gaps in the panpan1.1 assembly (**Supplementary Data Fig. S12, Supplementary Data Tables S15 and S16**).

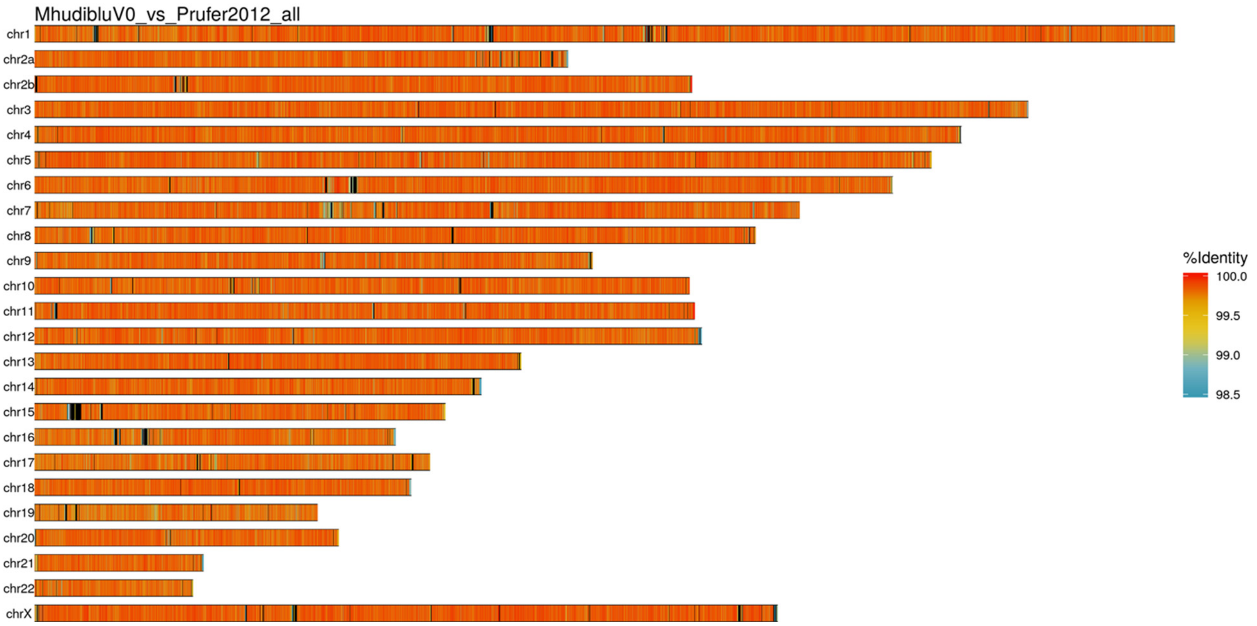

Supplementary Data Figure S11. Percent identity between panpan1.1 and Mhudiblu_PPA_v0. Each vertical line represents 1 kbp of alignment between the Mhudiblu_PPA_v0 and panpan1.1 assemblies and shades of blue to red depict the percent identity. Black lines highlight gaps (continuous stretches of N's) within the Mhudiblu_PPA_v0 assembly.

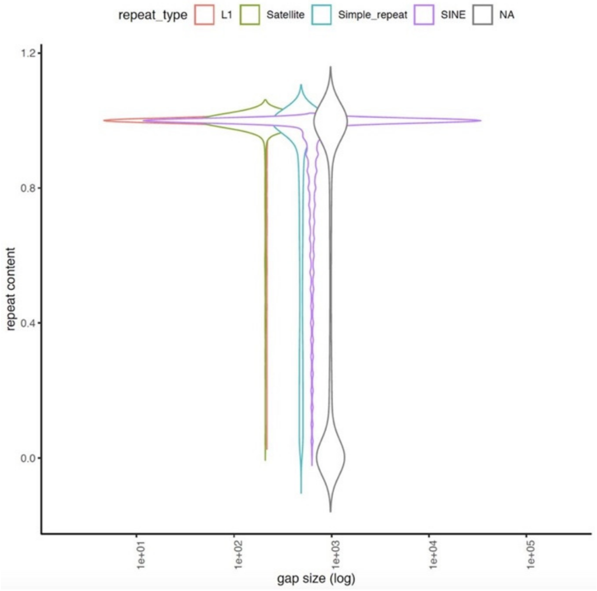

Supplementary Data Figure S12. Repeat content of filled gaps. Full-length L1s, satellites, simple repeats, SINEs, and NA (all other repeat elements including unmasked gaps) are shown. Repeat type was labeled by identifying the repeat nearest to the edge of the filled gap.

Supplementary Data Table S15. Repeat content of filled gaps and Mhudiblu_PPA_v0 chromosomes

Repeat type	Bases in panpan1.1	Bases in panpan1.1 (%)	Bases in filled gaps	gap %	Bases in PPA_v0	Bases in PPA_v0	Enrichment in gaps
SINE	352115212	13.13	12746772	38.73	361767402	13.12	3.02
LINE	602492350	22.46	5586900	16.97	604096139	21.91	0.77
LTR	257529363	9.6	1203661	3.66	256700990	9.31	0.39
RC	469131	0.02	1683	0.01	439330	0.02	0.32
Retroposon	1963713	0.07	1563122	4.75	9934783	0.36	15.45
DNA	107158409	3.99	409066	1.24	104782965	3.8	0.32
Unknown	820010	0.03	9064	0.03	793043	0.03	0.96
Unspecified	21761	0.00	0	0.00	20809	0	0.00
rRNA	153006	0.01	1818	0.01	162356	0.01	0.94
scRNA	169348	0.01	3053	0.01	182945	0.01	1.4
snRNA	440140	0.02	4427	0.01	434780	0.02	0.85
srpRNA	234467	0.01	108739	0.33	4426052	0.16	2.08
tRNA	89670	0.00	2070	0.01	94203	0	1.86
Satellite	7479304	0.28	434419	1.32	10145249	0.37	3.7
Simple_repeat	30670454	1.14	1502234	4.56	35110824	1.27	3.7
Low_complexity	5106073	0.19	341613	1.04	5866616	0.21	5.12
Total	1366912411	50.96	23918641	72.67	1394958486	50.6	1.44

Bases of each repeat type in Mhudiblu_PPA_v0 chromosomes as compared to repeat content of sequence spanning panpan1.1 gaps

Supplementary Data Table S16. Repeat content comparison between the human genome and two bonobo genomes: Mhudiblu_PPA_v0 and panpan1.1

	Human (GRChg38.p12)			Mhudiblu_PPA_v0			panpan1.1		
	number of elements	bases occupied	% of sequence	number of elements	bases occupied	% of sequence	number of elements	bases occupied	% of sequence
**SINEs:**	1892867	416832701	13.46	1762483	386266137	12.81	1663119	356727100	13.09
**ALUs**	1262135	328060827	10.60	1158907	301174656	9.99	1071290	273122767	10.02
**MIRs**	619014	87401339	2.82	592130	83748142	2.78	580594	82278976	3.02
**LINEs:**	1645583	672783752	21.73	1545454	631366606	20.94	1505575	610472599	22.39
**LINE1**	1007495	541107464	17.48	936791	505603384	16.77	908799	486927817	17.86
**LINE2**	541733	114544196	3.70	516989	109369612	3.63	506462	107347086	3.94
**L3/CR1**	69918	12070957	0.39	66362	11523815	0.38	65391	11389234	0.42
**LTR elements:**	790120	290875416	9.40	734122	268591934	8.91	712516	261156420	9.58
**ERVL**	176282	63091907	2.04	163629	58784274	1.95	159254	57515400	2.11
**ERVL-MaLRs**	367532	117737865	3.80	344809	110703494	3.67	335566	108144623	3.97
**ERV_classI**	189588	90776772	2.93	172493	82001513	2.72	165578	79021305	2.90
**ERV_classII**	11740	9979504	0.32	9795	8138434	0.27	9431	7654428	0.28
**Retroposon**	5825	4590833	0.15	4765	4631487	0.15	4930	2025017	0.07
**RC/Helitron**	2387	486584	0.02	2329	475327	0.02	2297	470615	0.02
**DNA elements:**	561489	116363401	3.76	533516	110750047	3.67	519121	108192093	3.97
**hAT-Charlie**	280257	50223720	1.62	265698	47778907	1.58	258160	46603909	1.71
**TcMar-Tigger**	134978	40150817	1.30	129191	38234076	1.27	125238	37311485	1.37
**Unclassified:**	6403	1002337	0.03	5309	946887	0.03	5129	878354	0.03
**Total**	4904676	1502935024	48.55	4587980	1403028425	46.53	4412689	1339922200	49.15
**Small RNA**	13031	1369269	0.04	11752	1191655	0.04	11076	1128576	0.04
**Satellites:**	7985	78950055	2.55	33333	57860274	1.92	11328	12595404	0.46
**Simple Repeats**	105712	6545010	0.21	97650	6136592	0.20	91938	5194771	0.19
**Low Complexity**	715588	39654176	1.28	660690	57031416	1.89	609417	31723051	1.16
**Non-N genome bases**		3095951186			3015531678			2725937204	

3.7 Divergent regions between Mhudiblu_PPA_v0 and panpan1.1 and SD overlap

To better understand whether the primary differences in the two assemblies were in regions of duplication, we compared the chromosomal segments in panpan1.1 that

were diverged (<99% identity) from those in the Mhudiblu_PPA_v0 assembly (**Supplementary Data Table S17**). As in other analyses reported here, the Mhudiblu_PPA_v0 chromosomes were divided into 1 kbp non-overlapping segments and aligned against the full panpan1.1 assembly (including chrUn) using BLAT (in client/server mode using -minIdentity=90). The 1 kbp segment alignments were categorized into the following categories for those considered as not having an alignment to the panpan1.1 assembly: (1) 95% to 99% identity, unique alignment to same chromosome in panpan1.1; (2) 95% to 99%, alignment to same chromosome in panpan1.1; (3) 95% to 99%, any alignment to panpan1.1; (4) <99%, any alignment to panpan1.1; (5) <99%, any alignment to panpan1.1 plus those with no alignment to panpan1.1 including gaps in Mhudiblu_PPA_v0. For segment counts, neighboring segments falling into the same category were merged into a single segment. In addition, the 1 kbp segments were categorized into the following categories for those that were considered to have a valid alignment to the panpan1.1 assembly (1) alignment to any region of the panpan1.1 genome at ≥99%; (2) alignment to the same chromosome in the panpan1.1 genome at ≥99%; and (3) unique alignment to the same chromosome in the panpan1.1 genome at ≥99% where unique is defined as the second best alignment for a given region having a score of <80% of that of the best score for that segment. Enrichment was calculated as the ratio of the values for the non-aligning to their corresponding category of aligning segments.

Supplementary Data Table S17. 1 kbp divergent regions overlapping SDs

Category of 1 kbp segment alignment percent identity	total segments*	total length	segments overlapping SDA+WGAC	length of segments overlapping olap SDA+WGAC	% segments SDA+WGAC	% bases SDA+WGAC	enrichment** % segments SDA+WGAC	enrichment** % bases SDA+WGAC
95% to 99%. unique alignment to same chromosome in panpan1.1a	37877	44859000	3580	5589000	9.5	12.5	0.62	1.01
95% to 99%, alignment to same chromosome in panpan1.1[a]	49190	66049000	6095	11203000	12.4	17.0	0.73	0.69
95% to 99%, alignment to any segment in panpan1.1[c]	54432	77138000	9886	19874000	18.2	25.8	0.88	1.02
<99%, any alignment to panpan1.1[c]	60983	114877000	10318	42354000	16.9	36.9	0.82	1.46
<99%, including gaps in v0 and no alignment to panpan1.1[c]	58637	151811000	7866	69905000	13.4	46	0.65	1.83

*segment count is after merging neighboring 1 kbp segments with <99% identity into a single segment

**enrichment defined as percentage of segments (or bases) with <99% identity divided by percentage of chromosome-specific uniquely aligning segments (or bases) with >=99% identity in a, divided by chromosome-specific aligning segments in b, and divided by all segments in c

3.8 Orientation differences between Mhudiblu_PPA_v0 and panpan1.1

Using methods described above (**section 3.6**), we compared the new bonobo assembly (Mhudiblu_PPA_v0) to the previously published version generated from a different individual, Ulindi (panpan1.1)[28]. Mhudiblu_PPA_v0 adds 74 Mbp of new sequence assigned to chromosome. As expected, contig size has been increased by more than two orders of magnitude and 99.5% of the euchromatic gaps have been closed (**Supplementary Table 7**). In addition, the analysis identified 46 potential inversions between Ulindi and Mhudiblu (**Supplementary Data Fig. S13**). Strand-seq data from Ulindi confirmed that five of these were errors in the original Ulindi (panpan1.1) assembly. With respect to the Ulindi assembly, the Mhudiblu assembly is more comparable with respect to the number of gaps and overall organization to the human reference genome (GRCh38) (**Supplementary Data Fig. S14**) and the Clint_PTRv2 chimpanzee genome assembly, which was generated with long-read sequence data (**Supplementary Data Fig. S15**).

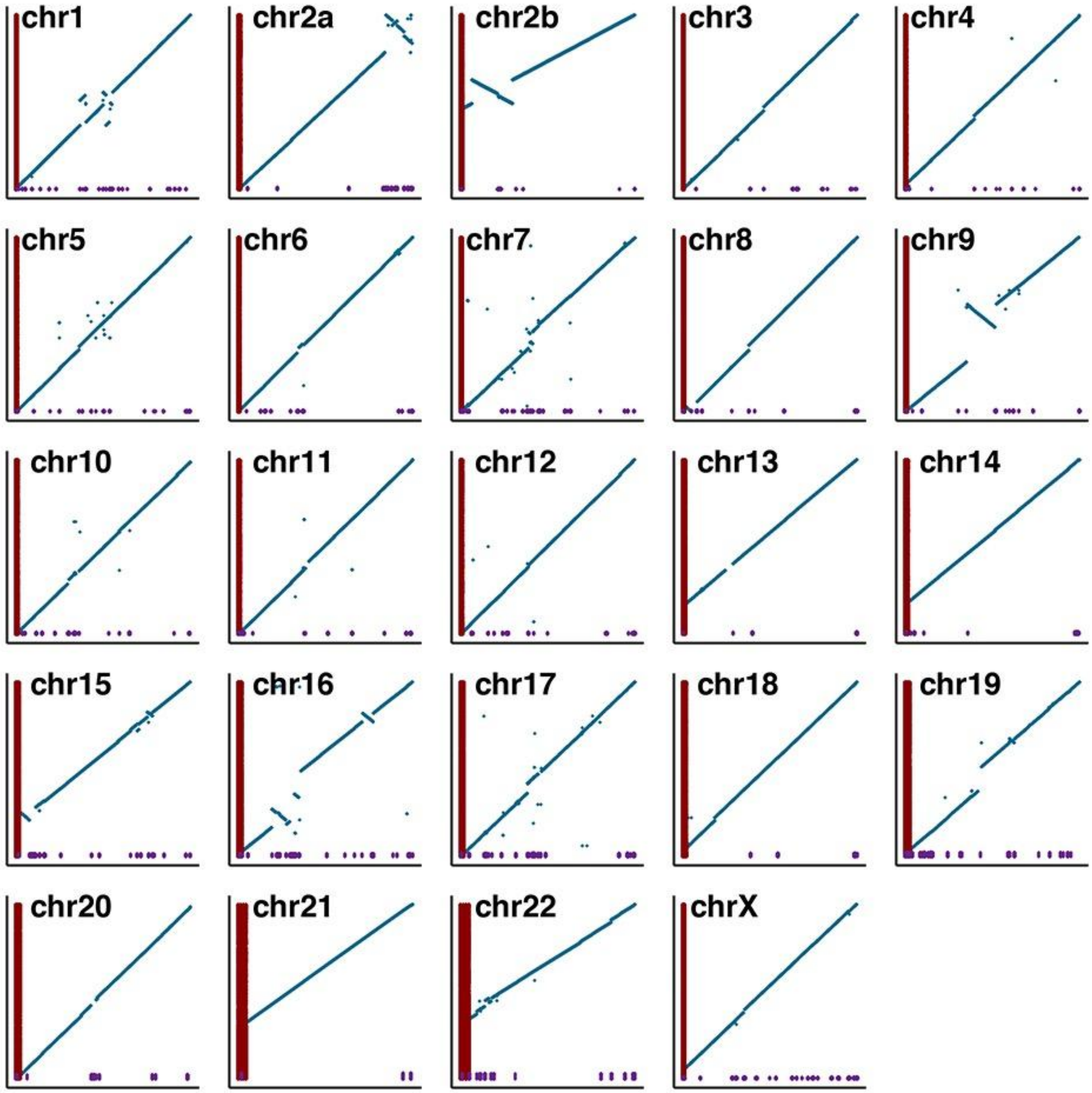

Supplementary Data Figure S13. Gap and orientation differences between bonobo assemblies. The Mhudiblu_PPA_v0 bonobo assembly compared with the bonobo assembly from Prufer et al. (2012). The current bonobo assembly contig gaps are shown along the x-axis in purple. The Prufer et al. (2012) assembly is represented along the y-axis, with the contig gaps shown in red. Alignment between the two genomes is represented in blue with each dot representing 1 kbp of alignment.

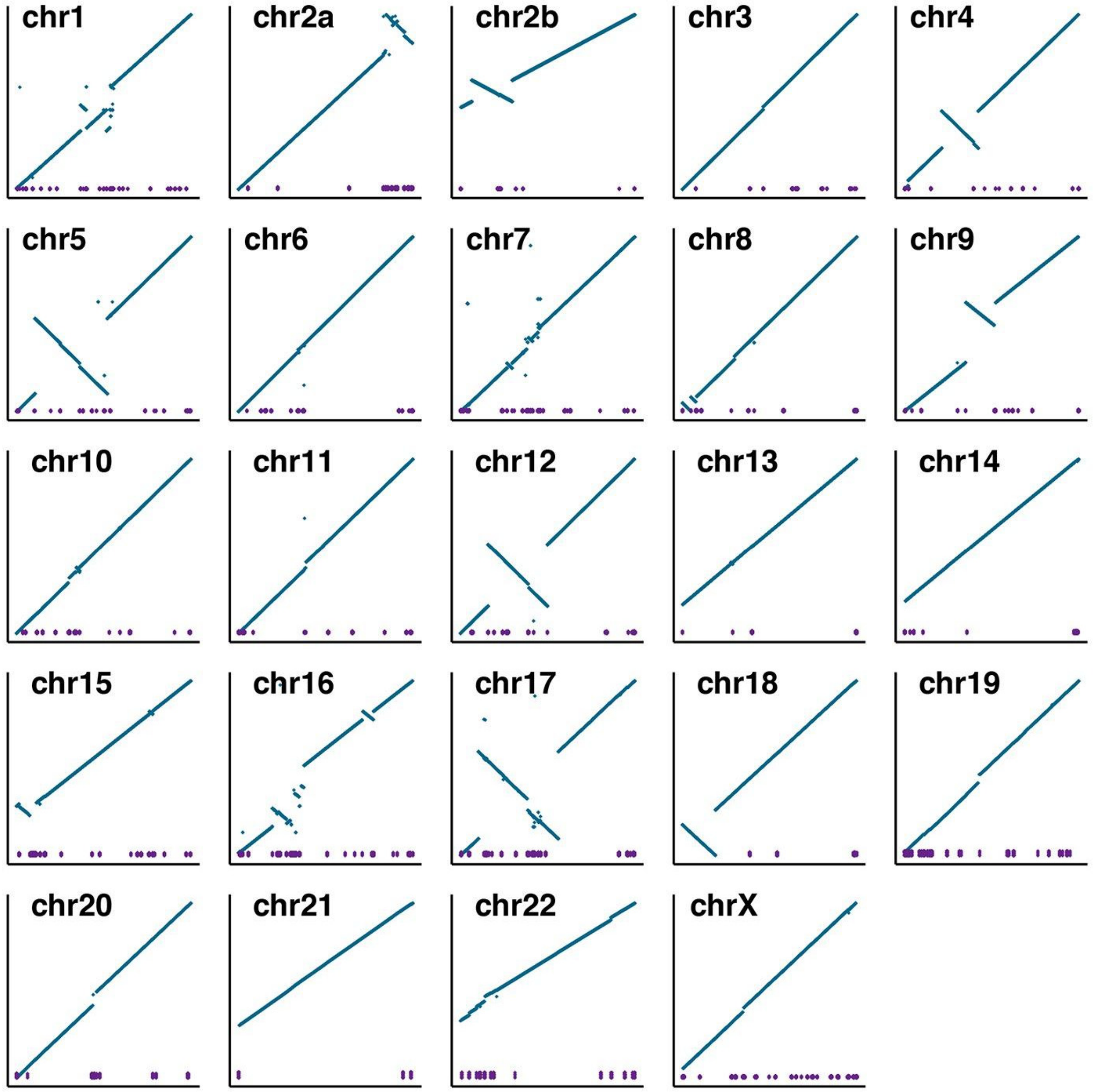

Supplementary Data Figure S14. Comparison of the human and bonobo assemblies. Alignment of the Mhudiblu_PPA_v0 bonobo assembly with the human genome (GRChg38.p12). Mhudiblu_PPA_v0 contig gaps are shown along the x-axis in purple. GRChg38.p12 is represented along the y-axis. Alignment between the two genomes is represented in blue with each dot representing 1 kbp of alignment.

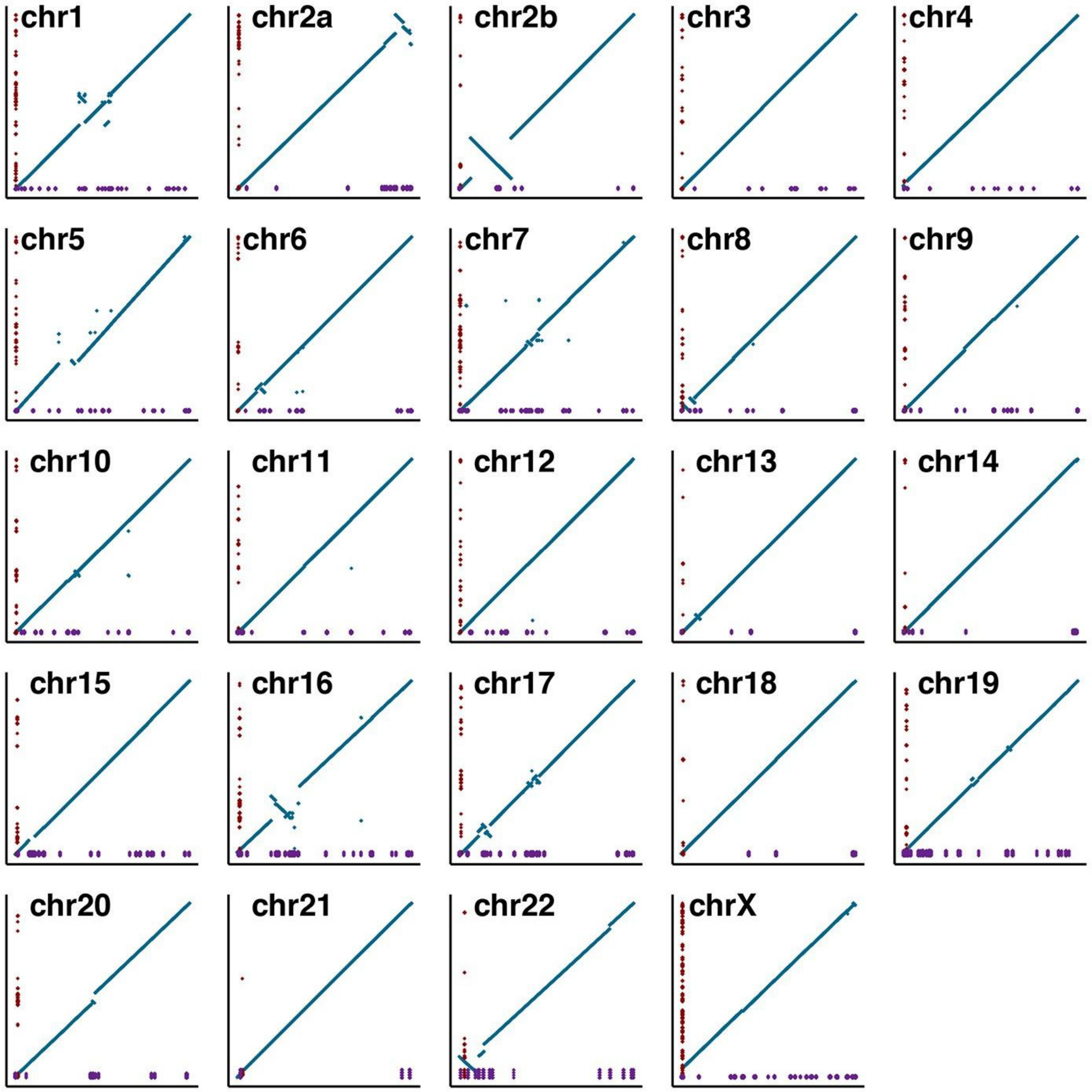

Supplementary Data Figure S15. Comparison of the chimpanzee and bonobo assemblies. Alignment of the Mhudiblu_PPA_v0 bonobo assembly with the chimpanzee genome (Clint_PTRv2). Mhudiblu_PPA_v0 contig gaps are shown along the x-axis in purple. Clint_PTRv2 is represented along the y-axis with gaps shown in red. Alignment between the two genomes is represented in blue with each dot representing 1 kbp of alignment.

3.9 Strand-seq analysis of panpan1.1 assembly

Since the underlying Strand-seq data was generated from the same source (Ulindi) that was used to produce the initial assembly, we evaluated the original assembly for potential orientation errors. The analysis identified 75 homozygous inversions corresponding to 80.14 Mbp of sequence that was incorrectly orientated in the initial draft of the Ulindi assembly (**Supplementary Data Fig. S16**). In addition, the analysis identified 148 heterozygous events that likely correspond to true inversion polymorphisms or collapsed regions in Ulindi assembly. In contrast, a comparable

analysis of the Mhudiblu_PPA_v0 assembly identified 29 homozygous inversions corresponding to 49.25 Mbp and 96 heterozygous events. Because these represent different individuals, we cannot exclude the possibility that homozygous events represent rare polymorphisms over potential orientation errors in the assembly.

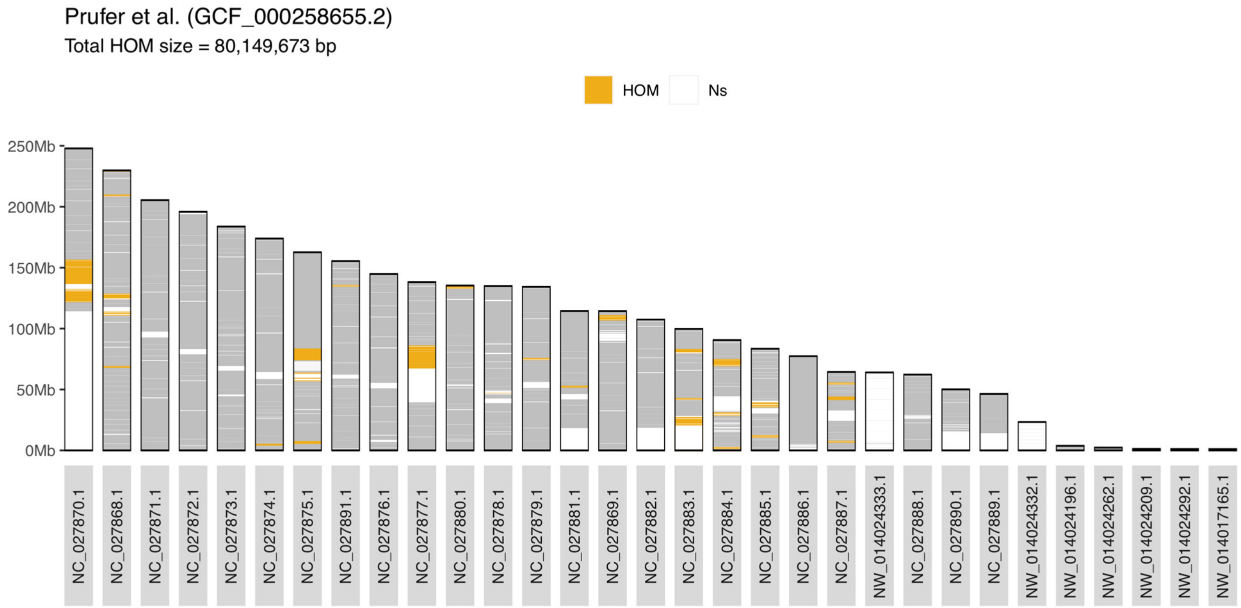

Supplementary Data Figure S16. Misoriented regions detected in panpan1.1 bonobo assembly. Bonobo assembly papan1.1[28] is plotted as light gray bars. Missing sequences (stretches of N's) are highlighted by white bars. Homozygous switches (HOM) in Strand-seq read directionality are highlighted with orange. Such switches in read directionality point to misorientations or genomic inversions.

3.10 Summary of Mhudiblu assembly quality

We initially assigned 2,839 Mbp of the bonobo genome to 149 scaffolds for an overall scaffold N50 of 70.7 Mbp (**Supplementary Data Table S1**). We performed subsequent FISH experiments to map ~67 Mbp contained within unassigned scaffolds >500 kbp in length. The procedure successfully placed an additional 11 previously unassigned scaffolds (totaling 60 Mbp) and correctly determined the orientation of 3 scaffolds (7 Mbp) enabling the discovery of novel structural differences with respect to the human genome (GRCh38) (**Supplementary Data Table S4**). A comparison against BAC-end sequence data from chimpanzee (*Pan troglodytes*) (**Supplementary Data Table S**8) and fully sequenced inserts from a BAC library (VMRC74) generated from Mhudiblu confirms a high degree of local contiguity. We compared the new bonobo assembly (Mhudiblu_PPA_v0) to the previously published version[30].

Based on an analysis of the gaps mapping to ordered and oriented chromosomes that could be tracked between the two assemblies (103,271), we found that >97.5% of the filled gaps are <2 kbp in length and 75% show greater than 70% repeat content (**Extended data Fig. 3, Supplementary Data Table S16**). For example, more than half the closed gaps (51.2% or 52,848 gaps) correspond to SINE repeats (mean repeat size of 257 bp) indicating that Alu repeats were misassembled in the original bonobo assembly. Larger repeats are also now better resolved with 32% (1,910/5,969) of the

full-length L1 repeats mapping to these closed gaps (**Extended Data Fig. 3**). Not surprisingly, gaps (n = 5,034) mapping to or adjacent (<1000 bp) to high-identity SDs tended to be larger in size (1,272 vs. 284 bp) although less abundant (**Extended Data Fig. 3**). In addition, a genome browser is available at UCSC Genome Browser (http://genome.ucsc.edu/cgi-bin/hgGateway?db=panpan3) along with a track hub with more detailed annotation (https://eichlerlab.gs.washington.edu/public/track_hubs/bonobo_chromosomes/hub.txt).

4. Bonobo genome assembly analyses

Among closely related species, such as the great apes, application of long-read sequencing has facilitated the production of genomes without guidance from the human reference genome[1]. The development of such new references, however, is far from an automated process. Although long-read sequencing has driven the development of more contiguous sequence, it still needs to be coupled with other orthogonal technologies, such as Strand-seq[20,31,32], optical mapping[33], and molecular cytogenetics (FISH)[9] in order to generate chromosomal-level assemblies that are not simply "humanized" by alignment to the human reference genome. This is only one of many approaches[34,35] being developed from advances in sequencing technologies to generate complete or nearly complete genome assemblies for the first time.

Such contiguous *ab initio* assemblies are important because studies of great ape genomes are frequently focused on the identification of the most likely functional genetic differences that distinguish apes. Comparisons of these new reference ape genomes, for example, have more than doubled the number of lineage-specific SVs (>50 bp)[1,36,37], including mobile element insertions (MEIs) that disrupt genes[38-40], copy-neutral inversions that alter regulatory landscape[12,17,41], and SDs that have led to gene family expansions important in species adaptation[42-44].

4.1 SD analyses

The original bonobo assembly harbored only a small fraction (~14.7 Mbp) of high-identity SDs with at least 80 Mbp of duplications represented as collapsed and unresolved[30]. To detect sequence-resolved SDs in the new bonobo assembly, we applied the whole-genome analysis comparison (WGAC)[45] method. This method detects duplications by generating pairwise alignments of ≥1 kbp at ≥90% sequence identity, excluding repeat-masked sequence (RepeatMasker 3.3.0 using library 'primates', Dfam3.1). This method identifies a total of 170,830,911 bp of SDs considering both assembled chromosomes and unplaced contigs (87,357,941 bp placed on mapped chromosomes). This predicts 10,704 nonredundant loci (8,175 on just mapped chromosomes) corresponding to 46,680 pairwise alignments ≥1 kbp at ≥90% similar. Of these pairwise alignments, 11,467 map between different chromosomes and 2,913 map within the same chromosome but are located at least 1 Mbp apart giving 14,380 interspersed SDs (**Extended Data Fig. 3 and Supplementary Figs. 6 and 7 and Supplementary Table 23**). Similar to the high-quality human genome and the long-read assembly of the chimpanzee[1], the majority of the alignments (82.2% or 14,380/17,494) are interspersed (i.e., mapping to different chromosomes or are

separated by at least 1 Mbp on a chromosome (**Extended Data Fig. 3 and Supplementary Fig. 7**).

4.2 Collapsed SD analyses

We also assessed the bonobo genome for potential collapsed duplications. Segmental Duplication Assembler (SDA)[46] was used to identify and unpack collapsed SDs in the bonobo assembly (command: SDA denovo --platform subread --pre sda --species bonobo). SDA begins by identifying collapsed regions in the assembly by detecting regions of excess read depth as previously described[47,48]. Using this method, SDA identified 718 collapsed regions. These collapsed regions occupy 24.46 Mbp of the assembly and represent 82.84 Mbp in the bonobo genome when mapped back to the reference. SDA then tries to unpack the collapsed regions by partitioning sequencing read identifying paralogous sequence variant information and assembling each paralogue separately. SDA was able to unpack 15.89 Mbp of the collapsed regions into 1,147 assembled contigs, which represent 55.88 Mbp of sequence in the bonobo genome (**Supplementary Table 24**). In an effort to identify missing genes that expanded on the bonobo lineage, we identified 1,575 Iso-Seq reads that mapped better to 201 loci than the original bonobo genome assembly (**Supplementary Table 25**).

4.3 Gene annotation analyses

Genome annotation was performed using the Comparative Annotation Toolkit (CAT) v2.1[49]. First, whole-genome alignments between the bonobo and human GRCh38 genomes were generated using Cactus v1.0[50,51] along with chimpanzee, gorilla and orangutan. CAT then used the whole-genome alignments to project the GENCODE V33 annotation set[52] from GRCh38 to bonobo. In addition, CAT was given Iso-Seq FLNC data to provide extrinsic hints to the Augustus PB (PacBio) module of CAT, which performs *ab initio* prediction of coding isoforms. CAT was also run with the Augustus Comparative Gene Prediction (CGP) module, which leverages whole-genome alignments to predict coding loci across many genomes simultaneously (Gene Prediction)[53]. CAT then combined these *ab initio* prediction sets with the human gene projections to produce the final gene sets and UCSC assembly hubs used in this project.

We performed a detailed comparison of lineage-specific innovations between human and bonobo and chimpanzee and gorilla CAT annotations and searched for indel differences (**Supplementary Data Fig. S17a**), discontinuous genes in a single or separate contigs (**Supplementary Data Fig. S17b**), and genes missing in the target genomes (**Supplementary Data Fig. S17c** and **d**). We were also able to identify novel gene models for genes thought to be the focus of human-specific adaptations and traits (**Supplementary Figs. 2 and Supplementary Data S18**).

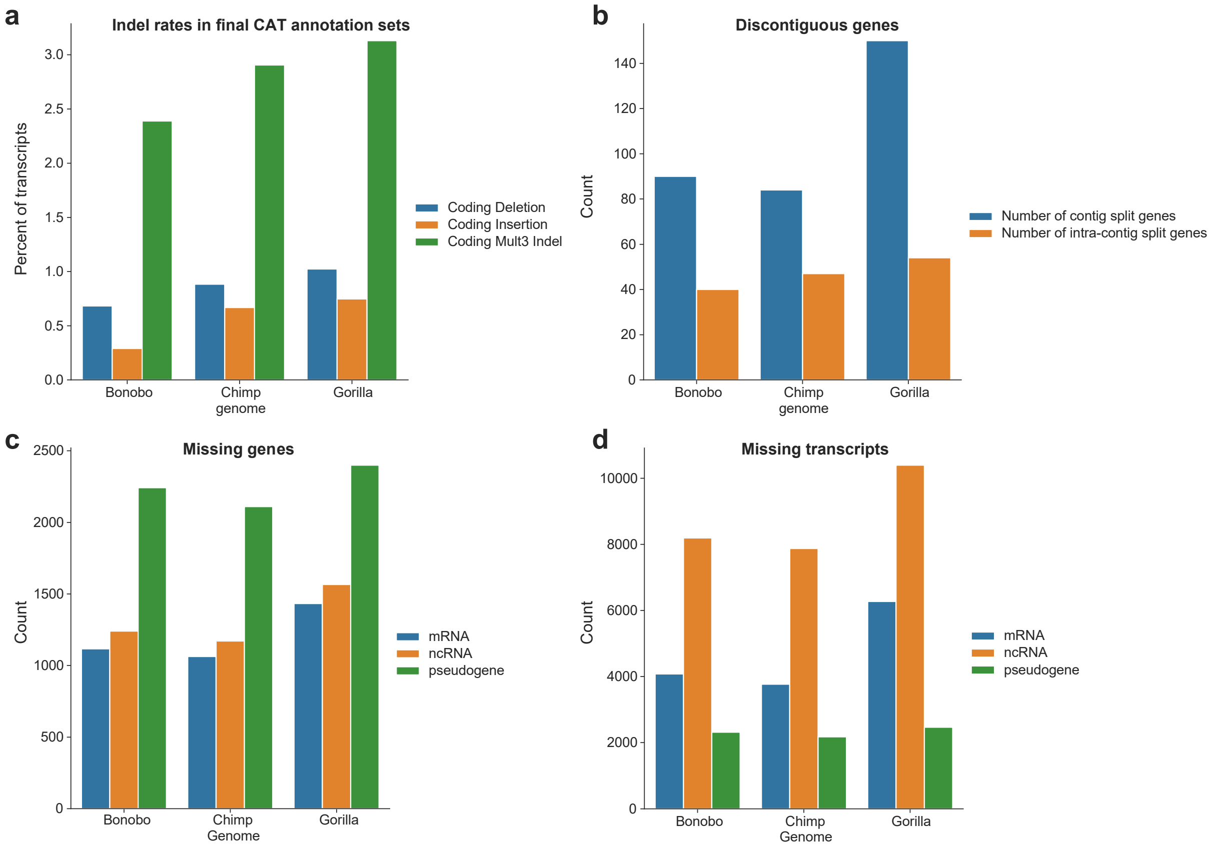

Supplementary Data Figure S17. Gene annotation. a, The number frameshifting and frame-maintaining indel differences seen between the GRCh38 and the target genomes. If selection did not occur, we would expect these three categories to be equal. We observe an enrichment of frame-maintaining indel differences, with the number of differences increasing with phylogenetic distance. We also observe an enrichment of frameshifting deletions relative to insertions, suggesting that there are a small number of assembly errors. **b,** The number of genes that appear to be disjoint within a single contig or present on multiple contigs for each target genome. Genes disjoint on a single contig found in separate whole-genome alignment chains, suggesting a rearrangement such as an inversion. Genes present on separate contigs are possibly signs of assembly errors, or can be caused by translocations. All split gene events are required to be separated by <10 bases in transcript coordinate space. This filter reduces contamination from paralogous alignments. The number of orthologous genes (**c**) or transcripts (**d**) present in the GENCODE V33 annotation of GRCh38 that were not identified in the target genome. The number of missing genes is comparable for bonobo and chimpanzee, and lower than gorilla. Genes can go missing at a handful of steps in the CAT annotation process—initially, they can drop out during the initial alignment and projection, or they can be filtered out due to very low alignment quality. They may also drop out during ortholog resolution, at which point they would be considered candidates for gene family collapse, which could be either biological or a result of collapsed SDs in the assembly.

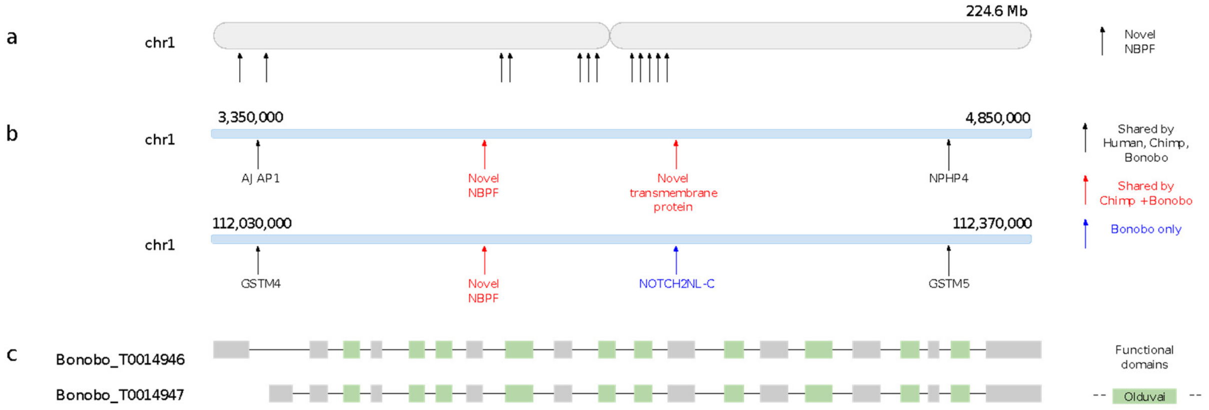

Supplementary Data Figure S18. 12 novel gene annotations with homology to *NBPF* genes predicted by the AugustusPB mode of CAT along chromosome 1 in bonobo genome. a, Locations of the genes occur in clusters along chromosome 1. **b,** Zoomed-in view of the synteny around two of the above novel *NBPF* genes. The top shows a novel *NBPF* between *AJAP1* and *NPHP4* that is shared between chimpanzee and bonobo (but not human), which occurs next to another novel transmembrane protein. The bottom shows a novel *NBPF* between *GSTM4* and *GSTM5*, which occurs in both chimpanzee and bonobo. The bonobo genome additionally has an annotation of *NOTCH2NL-C*, which is not seen in chimpanzee. **c,** Exon structure of two of these *NBPF*s are shown, which contain 10 Olduvai domains.

The NCBI Eukaryotic Genome Annotation Pipeline was also used to annotate genes, transcripts, proteins, and other genomic features on Mhudiblu_PPA_v0. Nearly five billion RNA-seq reads from various regions of the bonobo brain, heart, kidney, liver, testis, dermal fibroblasts, and iPSCs were retrieved from SRA and aligned to the repeat-masked genome using BLAST[54] followed by Splign[55], along with transcripts available in the NCBI databases on May 15, 2020, when the annotation started. This set of transcripts consisted of 218 known (curated) RefSeq transcripts and 191 GenBank transcripts from bonobo, and 74,670 known RefSeq and 322,433 GenBank transcripts from human. In addition, 80 RefSeq and 49 GenBank proteins from bonobo, 144,553 GenBank and 57,310 RefSeq proteins from human, and 21,436 RefSeq and 14,549 GenBank proteins from other primates were aligned to the genome using BLAST and ProSplign. The structure and boundaries of the gene models were derived by Gnomon from these alignments (https://www.ncbi.nlm.nih.gov/genome/annotation_euk/gnomon/ [Accessed: 14th August 2020]). Where alignments did not define a complete model, but the coding propensity of the region was sufficiently high, *ab initio* extension or joining/filling of partial open reading frames in compatible frames was performed by Gnomon, using a hidden Markov model (HMM) trained on bonobo. tRNAs were predicted with tRNAscan-SE:1.23[56] and small noncoding RNAs were predicted by searching the RFAM 12.0 HMMs for eukaryotes using cmsearch from the Infernal package[57]. The annotation of the Mhudiblu_PPA_v0 assembly (Pan Paniscus Annotation Release 104) resulted in 22,366 protein-coding genes, 9,066 noncoding genes, and 6,736 pseudogenes (see details in https://www.ncbi.nlm.nih.gov/genome/annotation_euk/Pan_paniscus/104/).

In summary, we annotated the bonobo assembly for genes using two different approaches. The first involved the NCBI Eukaryotic Genome Annotation Pipeline and is

available as *Pan paniscus* Annotation Release 104. It predicts 22,366 full-length protein-coding genes and 9,066 noncoding genes (https://www.ncbi.nlm.nih.gov/genome/annotation_euk/Pan_paniscus/104/). We also applied the CAT[49], which allowed us to incorporate nearly 857,000 full-length cDNA generated from a bonobo iPSC line and NPCs derived from the same cell line (**Supplementary Table 8**). These Iso-Seq data are particularly useful for validating novel gene models that may have emerged in the bonobo lineage. CAT annotated 20,478 protein-coding and 36,880 noncoding bonobo genes of which 99.5% of the protein-encoding models show no frameshift errors as predicted by Transmap[58]. We find that 38.4% of protein-coding isoforms are more complete when mapped to the new assembly (average increase of 1.5% to 2.1% for NCBI and CAT annotations, respectively) and 59.7% align better to Mhudiblu_PPA_v0 when compared to panPan1.1 (average increase of 0.76%). This level of accuracy, which is comparable to the human and recently released gorilla and chimpanzee genomes[1,36], allows for more detailed investigations of lineage-specific innovations, including gene models that have changed between bonobo and chimpanzee (**Supplementary Data Fig. S17**). We identify 119 genes that have potential frameshifting indels disrupting the primary isoform relative to the human reference (GRCh38) (**Supplementary Table 9**). We note that 90 gene structures are split over multiple contigs (and 40 within contigs) (**Supplementary Table 55**) and 206 protein-coding genes show evidence of being part of gene families that show reduced copy number in this assembly relative to human, with 174 of those showing a 2-to-1 relationship and 19 being 3-to-1 in human when compared to bonobo (**Supplementary Table 10**). In contrast, 1,576 protein-coding genes show evidence of gene family expansion in bonobo, with 959 copied once and 247 copied twice when compared to humans (**Supplementary Table 11**). In other cases, we identified novel gene models for genes thought to be the focus of human-specific adaptations and traits. Such is the case for the neuroblastoma-breakpoint (*NBPF*) gene family[59] where we identified 12 novel *NBPF* bonobo gene family members mapping along chromosome 1 (**Supplementary Data Fig. S18**). CAT predicts 1,736 novel transcripts that did not arise from any previously annotated transcript in the input human annotation. Many of these are relatively short (average length of 209 amino acids), corresponding to one or two exons. However, 342 novel transcript predictions have strong Iso-Seq support and are multi-exonic with at least two exons (**Supplementary Table 12**). CAT predicts 2,334 novel isoforms (**Supplementary Table 13**) relative to the current human annotation, and manual curation of this set identified 65 putatively novel exons with support from full-length cDNA (**Supplementary Table 14**), such as the novel protein-coding exon in *ANAPC2* found in bonobo but not in chimpanzee (**Supplementary Fig. 2**).

4.4 Creation of Mhudiblu_PPA_v1 assembly

We created an upgraded assembly version (Mhudiblu_PPA_v1), which corrected orientation errors and maximized assignment of unplaced contigs to chromosomes as well as attempted to resolve collapsed SDs. The initial Mhudiblu_PPA_v0 assembly was constructed *ab initio*, without guidance from the human GRCh38 reference or chimpanzee reference genome assembly (Clint_PTRv2). The Mhudiblu_PPA_v0 bonobo assembly entailed initially scaffolding all the assembled PacBio contigs (≥150 kbp in length) using Bionano Genomics optical maps (hybrid scaffolds). Scaffolds were

then assigned to bonobo chromosomes using the chromosomal FISH backbone of 324 BAC clones to assign 87 Bionano Genomics scaffolds representing 637/769 contigs or 2,787,283,929 bp of the bonobo genome. Chimpanzee BES data (CHORI-251) were used to map potential contiguous and discordant regions of the genome for further evaluation. Mhudiblu_PPA_v0 was released and annotated under NCBI accession GCA_013052645.1.

Creation of Mhudiblu_PPA_v1 consisted of four finishing steps. First, we applied Strand-seq to correct misassembly/orientation issues and assign unplaced scaffolds to chromosomes (**section 3.2**). To inform this process, we applied a second approach and compared the final assembly to the cytogenetic map, documented inversions[9,12,15-19] (**Supplementary Data table S5**), and panpan1.1, Clint_PTRv2, and GRCh38 and manually investigated potential differences changing only those where there was orthogonal support. For each chromosome, initially, the list of potentially misoriented regions (**Supplementary Data Table S12**) based on Strand-seq data in conjunction with the list of possible breakpoints (**Supplementary Data Table S10**) was reviewed. All Strand-seq informed inversions were introduced at contig boundaries, not within contigs. If the misoriented region was completely contained within a larger contig or spanned the border of two scaffolds but was a small portion of each of the bounding contigs (in 7 of the 8 the size of the inversion was <5% of the contig; in the eighth case the inversion was 30% of the size of the contig), the inversion and/or its potential breakpoints were by definition spanned by a single PacBio read and thus assumed to be polymorphic and not introduced. If the misoriented region spanned a single contig, the contig was inverted as long as a gene (NCBI RefSeq or CAT) did not span either of the bounding gaps. If a gene spanned into a neighboring contig, then the Strand-seq data was examined for the neighboring contig and the quality of the gene annotation was assessed to determine whether to include the neighboring contig in the inversion or break the gene by introducing the inversion at the original inversion breakpoint location. When the misoriented region spanned multiple contigs or when two misoriented regions were situated in neighboring contigs or near a Strand-seq breakpoint (**Supplementary Data Table S10**), it was necessary to determine whether the misoriented region(s) represented a misassembly or one or more inversions. To make that determination and to define the inversion boundaries, we used the cytogenetic map, gene and BAC-end linking information, documented bonobo inversion lists (**Supplementary Data Table S5**), Bionano Genomics data, and alignments to panpan1.1, Clint_PTRv2 and GRCh38.p12 genomes. Further, when two misoriented regions were situated within a contig of one another, the Strand-seq data for the intervening region was reviewed. If the intervening region was heterozygous, it was possible that the neighboring misoriented regions could be combined into a single inversion event.

After introducing the inversions defined by the Strand-seq data, some inconsistencies remained. On chromosome 1, for example, the cytogenetic markers were still not consistent with the order defined by FISH mapping experiments. Based on alignment to the other genomes, we identified a chimeric contig that led to the misassembly (000381F_294411_qpds_149449_295581; **Supplementary Data Fig. S19**). As when defining the inversion events, the gene and BES linking data were used along with

Bionano Genomics data, Strand-seq data, alignments to panpan1.1, GRCh38.p12 and Clint_PTRv2 along with the cytogenetic mapping data to reassess the order and orientation of the scaffolds. On chromosome 1, a total of 29.7 Mbp of contigs were moved from their original location (**Supplementary Table 34**) and one 80 kbp contig was inverted (**Supplementary Table 35**).

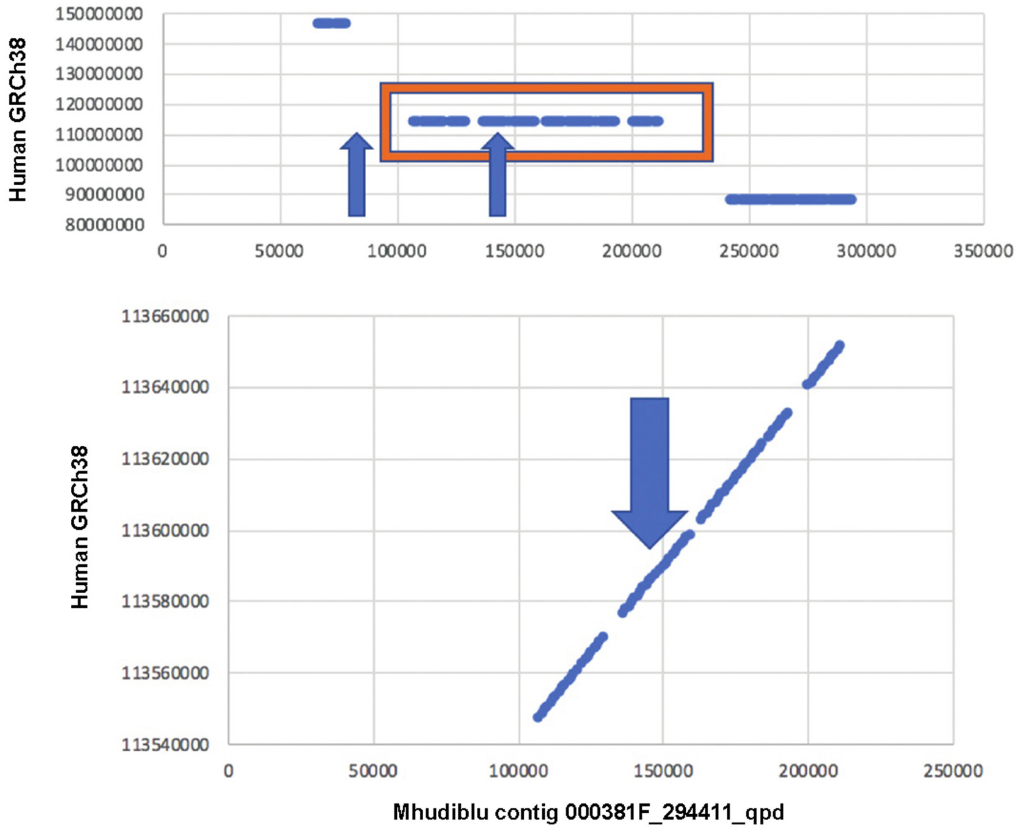

Supplementary Data Figure S19. Example of chimeric contig leading to chromosomal misassembly. Each dot corresponds to a uniquely aligning 1 kbp segment between Mhudiblu_PPA_v0 and the human genome (GRCh38). During the Bionano Genomics scaffolding process, the Mhudiblu assembled contig 000381F_294411_qpd was broken into three pieces at the locations designated by the blue arrows in the top panel (000381F_294411_qpds_1_82286, 000381F_294411_qpds_82287_149448, and 000381F_294411_qpds_149449_295581). The bottom panel shows the region from the red rectangle highlighting the location of the break (blue arrow) between the second and third segments of contig 000381F_294411_qpd. That break created a chimeric contig (000381F_294411_qpds_149449_295581) uniquely aligning to the human genome at 87 Mbp and 113 Mbp, not corresponding to an inversion breakpoint between the bonobo and human genomes. This chimeric contig led, in part, to the Bionano Genomics scaffolding process to misorder the scaffolds along chromosome 1.

On chromosomes 7 and 16, after introducing the inversions, the central complex region still was not consistent with the FISH documented structure for the region. On chromosome 7, by alignment to the other genomes, one primary chimeric contig was identified that had led to the misassembly. After breaking that contig (000369F_517724_qpd), the two pieces were placed in their separate locations. In the case of chromosome 16, the complex repeat structure of the central region presumably

resulted in the misassembly. On both chromosomes, using gene and BES linking, documented inversion data, and alignment and cytogenetic marker information, the sequence was organized to be consistent. In all cases, as much as was possible and when there was doubt, the order and orientation from the Mhudiblu_PPA_v0 assembly was retained. In total, there were 24 scaffolds (**Supplementary Table 36**) that were manually repaired in Mhudiblu_PPA_v1 (see also **Supplementary Table 37**) representing a total of 749 Mbp.

As a part of this process, we identified new scaffolds that could be assigned to chromosomes and ordered and oriented along the chromosomes. When alignments of the Mhudiblu_PPA_v0 unplaced scaffolds to the panpan1.1, GRCh38.p12, and Clint_PTRv2 genomes all confirmed that a scaffold could be inserted into the same location in the Mhudiblu_PPA_v1 chromosomes, and when the Strand-seq clustering data placed that scaffold on that chromosome, the scaffold was added to the ordered and oriented chromosome. Genome alignment data and the orientation information from Strand-seq clustering were used to be confident of the placement and orientation. In total, this approach added 36 Mbp of new sequence corresponding to 33 scaffolds (62 contigs) to the primary assembly (ordered and oriented chromosomes; **Supplementary Table 33**). During this process three contigs totaling 221 kbp that could not be accurately placed were removed from the ordered and oriented chromosomes (**Supplementary Table 38**). Lists of all scaffolds modified (**Supplementary Table 36**) as well as added, moved, and inverted contigs within the ordered and oriented chromosomes are provided (**Supplementary Tables 32-37 Supplementary Data Table S9**).

Additionally, any unlocalized scaffold at least 5 kbp in length with at least 75% of its length assigned to a single chromosome, and for scaffolds larger than 100 kbp assigned to the correct Strand-seq cluster, was assigned to the 'unlocalized scaffolds' for their respective chromosomes (**Supplementary Data Fig. S4, right**) for a total of 108 scaffolds spanning 13.4 Mbp.

Third, we added placeholders for both the centromeres (2 Mbp) and acrocentric regions (10 Mbp each) for each chromosome in the AGP (**Supplementary Data Table S18**). To place each centromere, first, the bounds of the region where the centromere should be placed was determined from the FISH mapping data. Second, RepeatMasker annotations were reviewed to identify the locations of any satellite/centromeric repeats. The centromere was then placed in the contig gap nearest to the centromeric repeats within the bounds defined by the FISH mapping data. For chromosomes 1 and 5, the centromere was inserted between contigs within a Bionano Genomics scaffold. For chromosome 8, a contig was broken for the insertion of the centromere. For other chromosomes, the centromere/short_arm gap was inserted between scaffolds.

Supplementary Data Table S18. Centromere placement (Mhudiblu_PPA_v0 coordinates)

chr		centromere placed at Mhudiblu_v0 contig gap start pos	distance to nearest centromeric repeat	FISH "left" boundary	FISH "right" boundary	centromeric repeat range start	centromeric repeat range end	centromeric repeat range start	centromeric repeat range end	centromeric repeat range start	centromeric repeat range end
**chr1**	centromere	12418438**	0*	121975291	136044402	106468979	106481532	124132360	124230338	203931045	204155971
**chr2a**	centromere	88412395	223	88397225	103669991	88412172	88645334	104842604	104927572		
**chr2b**	centromere	27672997	20	27042325	32937474	27601002	27672977	28162282	28166173		
**chr3**	centromere	90611787	0	87401499	98704282	81050091	81072951	90426944	90861882	122791494	122803786
**chr4**	centromere	72308747	0	72149337	73082221	72143847	73075742				
**chr5**	centromere	63881183**	0	59992930	66863827	63757314	64447214				
**chr6**	centromere	58812812	0	50485291	59357791	58449726	59185674				
**chr7**	centromere	58815635	11	54450204	62625227	58114701	58815624	61102338	61794931		
**chr8**	centromere	42534000***, **	0	41746423	47572299	42490007	42968679				
**chr9**	centromere	56413066	207734	55763008	60089490	56620800	57393462				
**chr10**	centromere	39042101	284	31117694	39975849	284	8120				
**chr11**	centromere	50129857	2	48217686	55351599	48600044	50129855				
**chr12**	centromere	51481570	0	47266347	53902413	50512298	51989612	50115388	50115817		
**chr13**	short_arm	1	297058	1	1255896	29465	30279	297058	298722	4734910	4735771
**chr14**	short_arm	1	214621	1	1216006	214621	215260				
**chr15**	short_arm	6615202	0	4839656	10252953	5193537	5193809	7993961	8003637		
**chr16**	centromere	25059091	1	25180356	26541058	25059192	26721712				
**chr17**	centromere	29766216	0	22295184	30456241	29580548	30037771				
**chr18**	short_arm	1	9781	1	1295472	9781	93798	3101681	3101891		
**chr19**	centromere	24011710	0	22872032	31120878	23336995	24788210	33262746	34736799		
**chr20**	centromere	26348865	0	25998453	27739741	25773041	25990405	26220272	26510119	27033590	27345273
**chr21**	short_arm	1	67576	1	8022645	67576	68033				
**chr22**	short_arm	1	1631675	1	1960149	4248371	4254221	6394988	6396453	(other satellite repeats at 1631675)	
**chrX**	centromere	51119794	0	49433419	51734747	50382068	50895759	51071105	51119787	51704604	51971369

* within the blocks of centromeric repeats, ** centromere required Super_Scaffold break between contigs, *** to place the centromere within the FISH boundaries required breaking a contig, ^ there are multiple blocks of centromeric repeat range start and end when there were multiple blocks of centromeric repeats on that chromosome

Finally, regions of SD collapse were assembled using SDA (**section 4.2**). Briefly, SDA uses correlation clustering to partition mapped PacBio CLRs based on paralogous sequence variants and assembles each paralog separately using either Canu[60] or Wtdbg2[61]. A total of 1,147 assembled paralog contigs totaling 55,883,605 bp were added to the unplaced chromosome. The Mhudiblu_PPA_v1 assembly contains a total of 3.1 Mbp (not including N's; 2.9 Mbp of which are on ordered and oriented chromosomes) organized into 5,526 scaffolds (6,124 contigs) and is available in NCBI under the accession: GCA_013052645.2 (**Supplementary Data Figs. S8 and S20, Supplementary Data Table S19**).

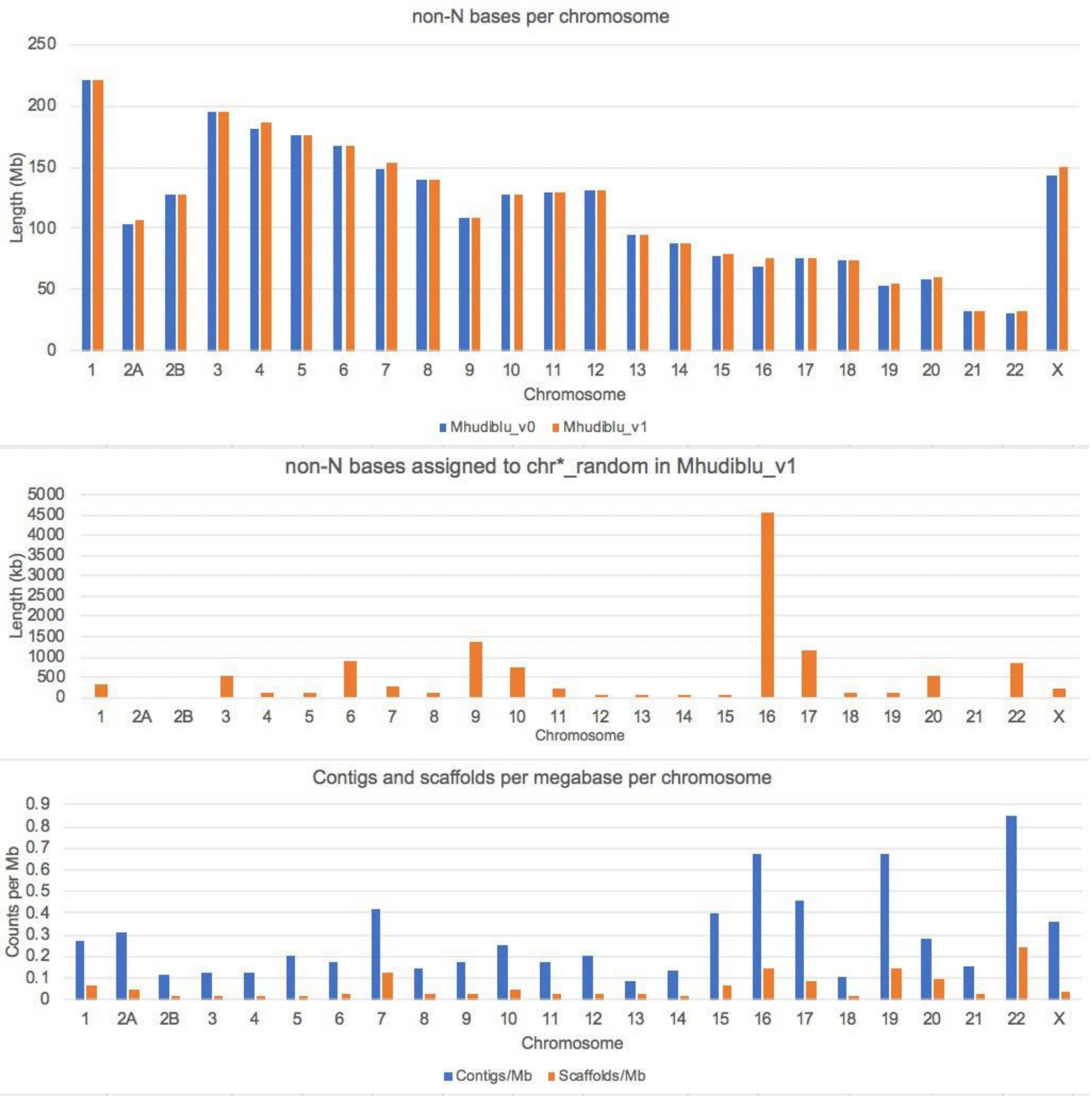

Supplementary Data Figure S20. Number of bases assigned to each chromosome in Mhudiblu_PPA_v0 and Mhudiblu_PPA_v1. The numbers of contigs and scaffolds per megabase per chromosome provide an indication of the complexity of assembly for each chromosome.

Supplementary Data Table S19. Final assembly statistics comparing Mhudiblu_PPA_v0, Mhudiblu_PPA_v1 and Mhudiblu_PPA_v2

	Mhudiblu_PPA_v0	Mhudiblu_PPA_v1 before adding contigs from Segmental Duplication Assembler (SDA)	SDA	Mhudiblu_PPA_v1	Mhudiblu_PPA_v2
**Total scaffolds**	4357	4379	1145	5524	5520
**Ordered/oriented scaffolds**	88	137	0	137	133
**Scaffolds on chr*_random**	0	108	0	108	108
**Scaffolds on chrUn**	4269	4134	1145	5279	5279
**Contigs**	4976	4977	1145	6122	6118
**Ordered/oriented contigs**	641	697	0	697	693
**Contigs on chr*_random**	0	125	0	125	125
**Contigs on ChrUn**	4334	4155	1145	5300	5300
**non-N bases (contigs)**	3,015,350,297	3,015,333,734	55,883,605	3,071,217,339	3,073,752,221
**Scaffold bases (including Ns)**	3,051,901,337	3,049,120,773	55,883,605	3,105,004,378	3,107,539,260
**non-N bases on chromosomes**	2,756,975,881	2,790,338,069	0	2,790,338,069	2,793,604,526
**bases on chromosomes (including Ns)**	2,787,676,126	2,918,899,387	0	2,918,899,387	2,920,672,989
**bases on chr*_random (not including Ns)**	0	12,455,377	0	12,455,377	12,482,156
**Contig N50**	16,579,680	16,579,680		16,070,023	16,076,652
**Contig L50 count**	48	49		50	50
**Scaffold N50**	68,246,502	55,818,576		53,354,638	53,386,619
**Scaffold L50 count**	16	18		19	19

After creating Mhudiblu_PPA_v1, the Strand-seq analysis was run and all remaining issues were examined. Two types of issues remained in Mhudiblu_PPA_v1. First, there were inversions that were smaller than a contig (**Supplementary Data Table S20**), thus spanned by or having their breakpoints spanned by a long read. These types of inversions are expected to be primarily polymorphisms and thus were not changed.

Supplementary Data Table S20. Mhudiblu_PPA_v1 coordinates for strand-seq events smaller than a contig, potentially polymorphic

chr	start	end	width	Ws	Cs	Comments
**chr2a**	17862887	18007468	144582	109	14	144kb in a 458kb contig
**chr6**	149764084	149816945	52862	147	4	52kb in a 91Mb contig
**chr7**	98374949	98409334	34386	683	77	34kb in a 2Mb contig
**chr19**	6661711	6668473	6763	320	33	6kb in a 600kb contig
**chr19**	35870817	35873490	2674	119	29	2kb in a 1.5Mb contig
**chrX**	49795486	49805116	9631	288	17	9.6kb in a 2Mb scaffold
**chrX**	152546141	152718773	172633	457	9	172kb buried in a 3.7Mb contig
**chr15**	22259734	22344095	84362	127	42	84kb straddles boundary of a 103kb and 1.7Mb scaffold

Second, there were initially inversions that had been identified by Strand-seq in Mhudiblu_PPA_v0, but because the breakpoints were not contained within the assembly and no additional data confirmed these inversions, they were not introduced in Mhudiblu_PPA_v1 and are predicted to be polymorphic in bonobo (**Supplementary Data Table S21**).

Supplementary Data Table S21. Mhudiblu_PPA_v1 coordinates for Strand-seq events without additional data confirming the inversion

chr	start	end	width	Ws	Cs	Comments
**chr10**	47092930	49623483	2530554	10426	382	2 contigs, bordering documented inversion chr10_inv6
**chr2a**	21346946	21657915	310970	960	43	2 contigs
**chr20**	25785948	25975497	189550	364	97	189kb straddling two contigs

Finally, after creation of the Mhudiblu_PPA_v1 chromosomal files, alignments were generated against the human genome (**Supplementary Data Fig. S21**).

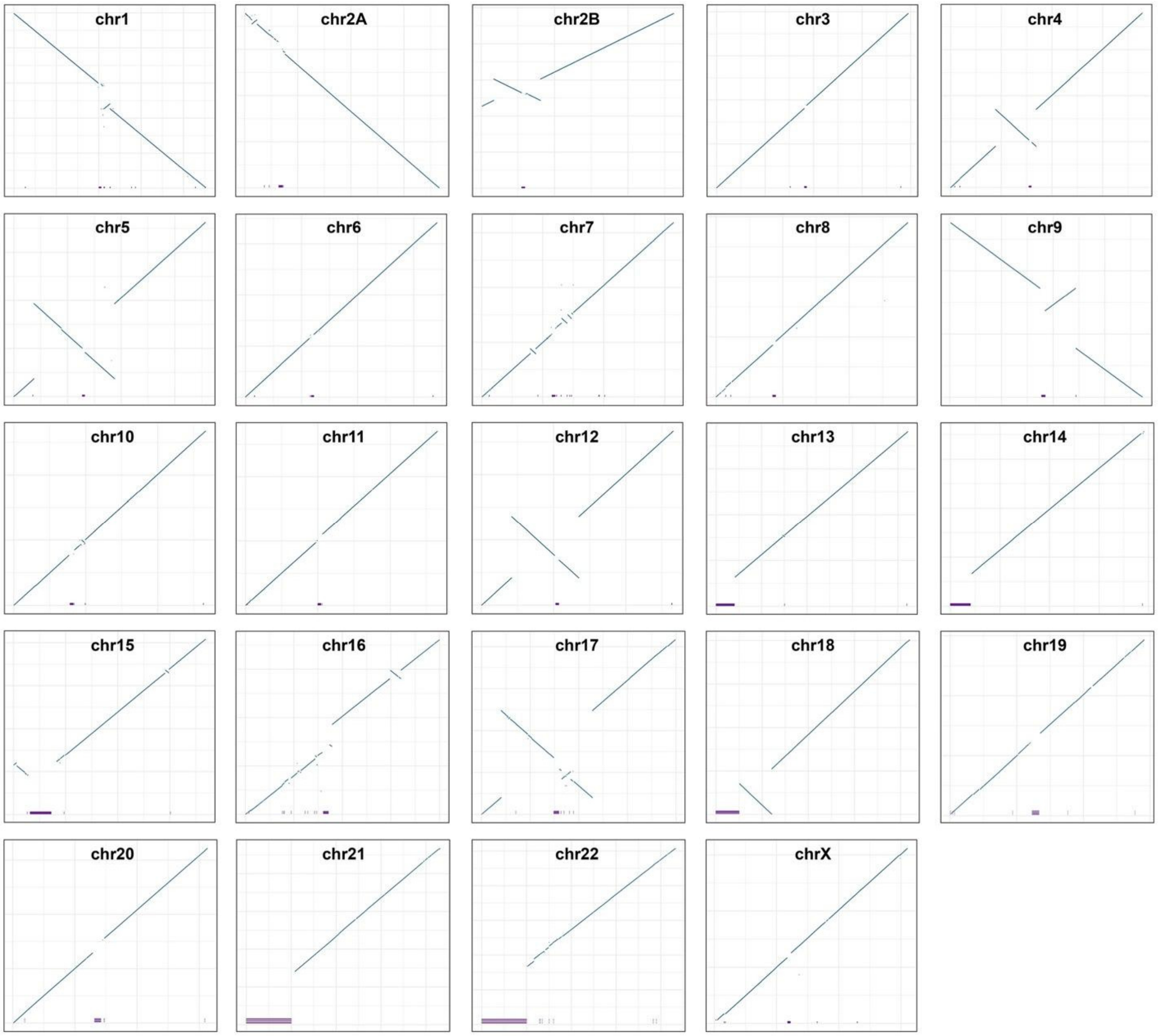

Supplementary Data Figure S21. Comparison of the human and bonobo (Mhudiblu_PPA_v1) assemblies. Alignment of the Mhudiblu_PPA_v1 bonobo assembly with the human genome (GRChg38.p12). The Mhudiblu_PPA_v1 contig gaps are shown along the x-axis in purple. GRChg38.p12 is represented along the y-axis. Alignment between the two genomes is represented in blue with each dot representing 1 kbp of alignment.

We checked that the Mhudiblu_PPA_v1 version of the assembly had not disrupted any of the genes investigated in Mhudiblu_PPA_v0: in the RefSeq gene set, two putative genes of unknown function were, in fact, disrupted (gene_id: LOC117980845, LOC100977127); in the final CAT gene set seven genes were interrupted (gene_id: Bonobo_T0015403, Bonobo_T0015688, Bonobo_T0026896, AC136431.2-201, Bonobo_T0078976, Bonobo_T0091676, PMS2CL-204). Most of these "broken gene models", with the exception of Bonobo_T0026896 or ASH2, do not have strong support and were novel predictions based solely on Augustus PB.

To improve the quality of our assembly, we generated an additional 40-fold high-fidelity (HiFi) sequence data by CCS from the same source genome (Mhudiblu) and used this

to further correct remaining sequencing errors. We used Racon (two rounds) to error correct the genome eliminating ~128,000 remaining errors for an overall accuracy of one error every 12,882 base pairs (improving QV from 39 to 41.1). This improved quality assembly is being released as Mhudiblu_PPA_v2. A fluxogram of the complete process of initial contig assembly (Mhudiblu_PPA_v0), order and orientation (Mhudiblu_PPA_v1), and polishing (Mhudiblu_PPA_v2) is reported in **Extended Data Fig. 1**.

5. Incomplete lineage sorting (ILS) analysis

While there is some evidence of limited gene flow between incipient species as well as potential archaic populations[62], chimpanzee and bonobo have been largely genetically isolated for at least a million years, thus providing a unique framework to understand the rapidity of hominid genetic changes that underlie phenotypic differences between species, such as cognitive development[63], differences in infectious disease[64], and anatomical changes[65]. Chimpanzee is most frequently used as an outgroup for human genetic analyses; however, some phenotypic assessments have suggested that bonobo may in fact be more relevant for some traits, including neuroanatomical specializations[66]. A high-quality genome assembly is critical not only for the comprehensive identification of those genetic differences, but also for our understanding of shared genetic history through processes such as ILS.

5.1 Genome-wide ILS analyses

We searched for evidence of ILS among the chimpanzee, gorilla, and human lineages at different levels of resolution. We downloaded the human (GRCh38), chimpanzee (Clint_PTRv2), and gorilla (Kamilah_GGO_v0) genomes from NCBI. Similar to bonobo, the latter two had been generated with long-read sequence data. We segmented the GRCh38 genome to generate datasets with different window lengths (20 kbp, 10 kbp, 5 kbp, 2 kbp, 1 kbp, and 500 bp). For each segment dataset, we used liftOver (ucsc/20160823)[67] to identify coordinates from bonobo, chimpanzee, and gorilla genomes, respectively. Next, we grouped corresponding human, chimpanzee, bonobo, and gorilla segments and applied Prank (v.140110)[68] to construct multiple sequence alignments (MSAs). Finally, we applied a maximum likelihood (ML) method to reconstruct phylogeny with IQ-Tree (1.6.11) and we selected the gene trees with bootstrap values greater than 50 for the following analysis. **Supplementary Table 48** shows how many gene trees we successfully reconstructed in each dataset.

Next, we regarded gene trees different from the species tree ((gorilla,((bonobo,chimp),human))) as ILS and used the ete3 module to count the number of segments under ILS in python3. All codes were modified from TREEasy[69].

We found that the proportion of ILS in the genome was increasing with the decrease of the segment sizes because large segments probably conceal ILS signals. We also found GC content in small ILS segments (500 bp: 40.54%) is higher than in large ILS segments (20 kbp: 37.7%) and more Alu sequences were observed in small segments. Moreover, we found that intergenic regions have a higher proportion of ILS compared to

intragenic regions and that ILS was rarely observed in the exon sequences (**Table 1**). Namely, as window size decreases the GC, Alu content, and genic content rise (**Table 3**). Irrespective of window size, genic regions remain depleted (>35%) compared to the genome average.

CoalHMM was used to calculate ILS proportion in the previous study[28], but CoalHMM suggests using segments larger than 1 Mbp as input. Then, we concatenated continuous 20 kbp segments into 101 segments greater than 1 Mbp (total: 127 Mbp). The ILS proportion calculated by CoalHMM is similar to our phylogenetic method (**Supplementary Data Table S22**).

Finally, we downloaded coordinates of exon RefSeq, gene annotation, Alu elements, and L1 elements from the GRCh38 UCSC Genome Browser and used BEDTools to count how many base pairs of exon/Alu/L1 overlapping with ILS segments.

Supplementary Data Table S22. ILS analysis on 101 segments with CoalHMM

Threshold (possibility)	(G,((B,H),C)) (%)	(G,((H,C),B)) (%)	ILS (%)
0	2.28	2.26	4.55
0.5	1.84	1.84	3.68
0.95	0.58	0.60	1.17

In addition, we repeated our analysis at a resolution of 500 bp including both orangutan (Susie_PABv2) and gorilla (Kamilah_GGO_v0) genomes. Considering only those tree topologies where there is at least 50% bootstrap support (≥50%), we estimate that >36.5% (**Supplementary Table 52**, **Supplementary Data Fig. S22**) of the genome shows evidence of ILS with 31.92% belongs to two deeper ILS topologies (orangutan,(((bonobo,chimp),gorilla),human)) and (orangutan,((bonobo,chimp),(gorilla,human))). These estimates are consistent with earlier estimates of 30%[70] and ~36%[1]. Interestingly, if we eliminate the requirement of bootstrap support (as was done previously), the estimate of ILS increases to 50.26%.

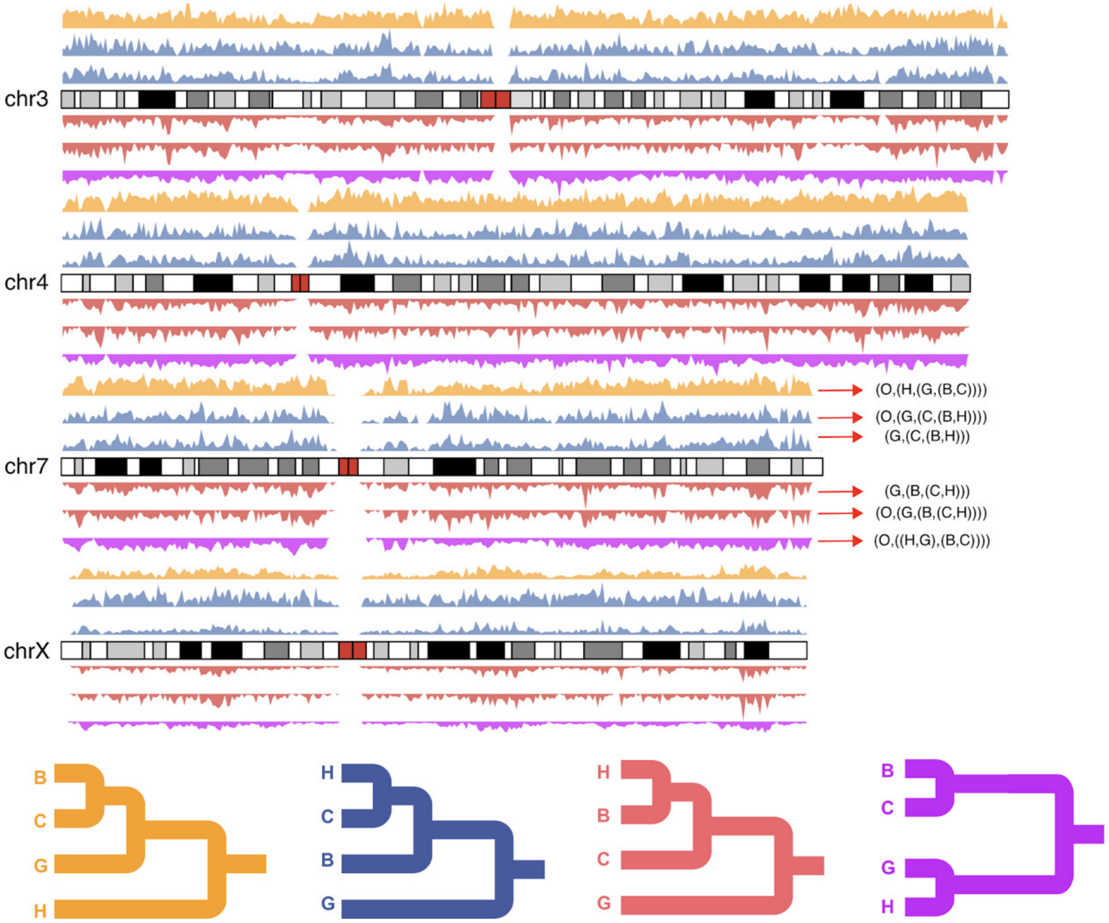

Supplementary Data Figure S22. Chromosome view of ILS. The schematic depicts human chromosomes 3, 4, 7 and X (GRCh38) with distribution of six different ILS shown as density plots. A subset of the major topologies are shown above and below the line (as indicated by color and arrow) and examples are shown with and without using orangutan as an outgroup.

5.2 Effective population size of Pan and Pan/Homo ancestral groups

The relatively high proportion of ILS within the *Pan* genus suggests that the population predating their species divergence was relatively large, with most reductions in population size occurring more recently. To test this, we applied the pairwise sequential Markovian coalescent (PSMC) method using Illumina WGS data from bonobo and chimpanzee (**Supplementary Table 42**) mapped back to the new reference genomes and inferred changes in effective population as well as timing of population expansions (**Extended Data Fig. 2**). We considered the population split of human and chimpanzee between 4-7 million years ago (mya) and 1-2.5 mya for the split of the chimpanzee and bonobo lineages. Using a 25-year generation time and a mutation rate $\mu = 0.5 \times 10^{-9}$ mut (bp x year), we estimate a large population size for the ancestral bonobo/chimpanzee lineage (Ne=~20,000). Similarly, we estimate that Pan-Homo ancestral population size is greater than 50,000. These estimates are similar to those performed on the earlier draft versions of the bonobo and chimpanzee genomes (as reported in Prufer et al. 2011[28] and Prado-Martinez et al. 2013[71]). However, it is important to note that, if the

mutation rate used by Prado-Martinez et al. 2013[71] and Prufer et al. 2011[28] is considered (μ= 1 x 10^{-9} mut (bp x year)), our estimates for the bonobo/chimpanzee population size are lower than those reported (23,000-37,000 and 27,000 ± 400, respectively), as shown in **Supplementary Data Table S23**. This discrepancy is likely due to the different methodologies employed, CoalHMM and CoalILS. We generated PSMC plots for comparison to the earlier work.

Supplementary Data Table S23. Estimates of effective population size (Ne x 104) using PSMC for key temporal intervals

	μ= 0.5 x 10^{-9} mut (bp x year)			μ= 1 x 10^{-9} mut (bp x year)		
	**t0**	**t1 (1Mya < t < 2.5Mya)**	**t2 (4Mya < t < 7Mya)**	**t0**	**t1 (1Mya < t < 2.5Mya)**	**t2 (4Mya < t < 7Mya)**
**Chimpanzee**	1.15 (0.32-1.85)	1.97 (1.42-4.65)	7.43 (4.46-9.96)	0.57 (0.16-0.93)	1.10 (0.71-3.58)	4.8 (4.22-5.22)
**Bonobo**	0.22 (0.1-0.52)	2.22 (0.99-2.76)	9.50 (6.08-13.04)	0.11 (0.05-0.26)	1.66 (1.32-5.16)	5.08 (4.96-5.43)

*t=0 is the final Ne,t1 is the time predating the chimpanzee/bonobo divergence, t2 is the time interval predating the pan/homo divergence. We use a generation length of 25 years. u=mutation rate

5.3 ILS analysis of protein-coding exons

To understand the relationship between ILS and protein-coding exons, we constructed an MSA dataset based on GRCh38 exon RefSeq as described above. We found that 1,446 exons mapped to the topologies of human-bonobo/human-chimpanzee ILS, of which 713 exons were under human–bonobo ILS and 733 exons were under human–chimpanzee ILS (**Supplementary Table 57 and Supplementary Table 49**). Interestingly, we found that 40 genes and 44 genes contain at least two exons under human–bonobo and human–chimpanzee ILS, respectively (**Supplementary Fig. 9**). In particular, we found the genes under human–bonobo ILS were enriched in photoreceptor activity and the genes under human–chimpanzee ILS were enriched in EGF-like domain and transmembrane regions (**Supplementary Table 51**). We also observed some genes that contain multiple exons under ILS were clustered in the genome (**Supplementary Fig. 9, Supplementary Data Table S24**). We also observed the exons under ILS evolved faster than non-ILS exons (**Fig. 3 and Supplementary Data Fig. S23**).

We set the same dN/dS value on all branches and used the branch model to calculate a dN/dS value in codeml with PAML (4.9a)[72]. For non-ILS exons, we randomly sampled 700 non-ILS exons and calculated a mean value of their dN/dS values. Then, we repeated this approach 100 times and generated a distribution of mean dN/dS values of non-ILS exons. For ILS exons, we calculated each dN/dS value of each exon. We selected genes containing at least two exons under ILS for enrichment analysis. Enrichments were performed with David (6.8)[73]. All plotting and t-tests were performed in R (3.4.3).

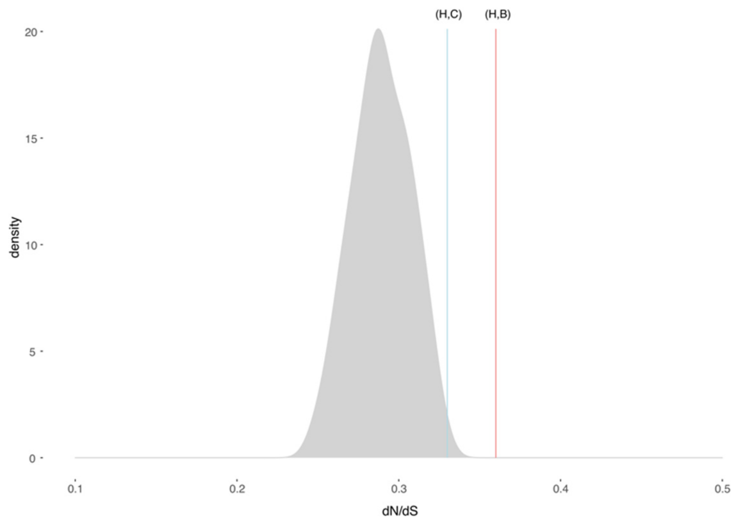

Supplementary Data Figure S23. Distribution of dN/dS values for ILS exons. The black line shows the distribution of dN/dS values for non-ILS exons. The dN/dS value of exons under human–bonobo ILS (red line, P-value = 0.004778) and under human–chimpanzee ILS (blue line, P-value = 0.03924) are significantly shifted from the genome distribution. Significance performed using the one-sample t test in R.

Supplementary Data Table S24. The functional annotation of genes in the clustered segments

Annotation Cluster	Enrichment Score	P_Value
Glycoprotein	5.95	1.00E-10
receptor-mediated endocytosis	2.7	0.00025
CUB domain	2.53	0.0016
Lectin	2.09	0.00087
Cell junction Synapse	1.98	0.00044
EGF-like calcium-binding	1.78	0.00057
G-protein coupled receptor	1.49	0.0085
terminal bouton	1.26	0.0087
FERM domain	1.22	0.039
Sushi	1.14	0.033
Ig-like C2	1.09	0.003
Serine protease	1.02	0.00025

5.4 Evolutionary modeling of ILS as a Poisson process and ILS desert analysis

To investigate the expected length of ILS segments between human and chimpanzee/bonobo, we modeled the evolution of ILS using parametric simulations. Briefly, here we modeled the evolution of a shared segment between two groups following a Poisson process with a rate inversely proportional to (r × t), where r is the recombination rate and t is the sequence divergence time between the two groups.

Because the segment is shared and observed in both groups, under neutrality the length distribution follows the sum of two independent and identical exponential random variables. We performed simulations under a range of realistic parameter values to account for parameter uncertainty. Specifically, each simulation was based on values uniformly drawn for a generation time [11.7-45.4] years per generation[74] and a recombination rate between 1×10^{-8} - 2×10^{-8} per base per generation). Mean and 95% confidence intervals of ILS tract length were computed using 1,000 simulations generated for each of the five different human–chimpanzee/bonobo divergence times (**Supplementary Data Table S25 and Supplementary Data Fig. S24a**).

As part of this analysis, we considered different window sizes ranging from 500 bp to 20 kbp in length (**Table 1**). Because the expected length of sharing a segment between two groups is inversely proportional to the recombination rate and the time since the divergence, for chimpanzee and bonobo, we anticipate ancestral track lengths between the two will be ~450–1040 bp (assuming a mean recombination rate between 1×10^{-8} – 2.3×10^{-8} and bonobo–chimpanzee divergence of ~1.5 million years; **Supplementary Data Fig. S24, Supplementary Data Table S25**). Our data suggest that conditional on the observations of incongruence between gene trees and the species tree, the mean length of ILS tracts for human–chimpanzee/bonobo should be between 372–558 bp (95% C.I.: 90–3,100 bp; **Supplementary Data Fig. S24, Supplementary Data Table S25**).

Supplementary Data Table S25. Mean and confidence intervals of ILS tract length in human-chimpanzee/bonobo

**H-C/B Divergence (million years)***	**4**	**4.5**	**5**	**5.5**	**6**
Expected length	5580357	4960317	4464286	4058442	3720238
2.5 percentile	13516143	12014349	10812914	9829922	9010762
97.5 percentile	3109176	2763712	2487341	2261219	2072784

*Mean and confidence interval of ILS tract length given different human-chimpanzee/bonobo sequence divergence scenarios were computed based on a model of a Poisson process

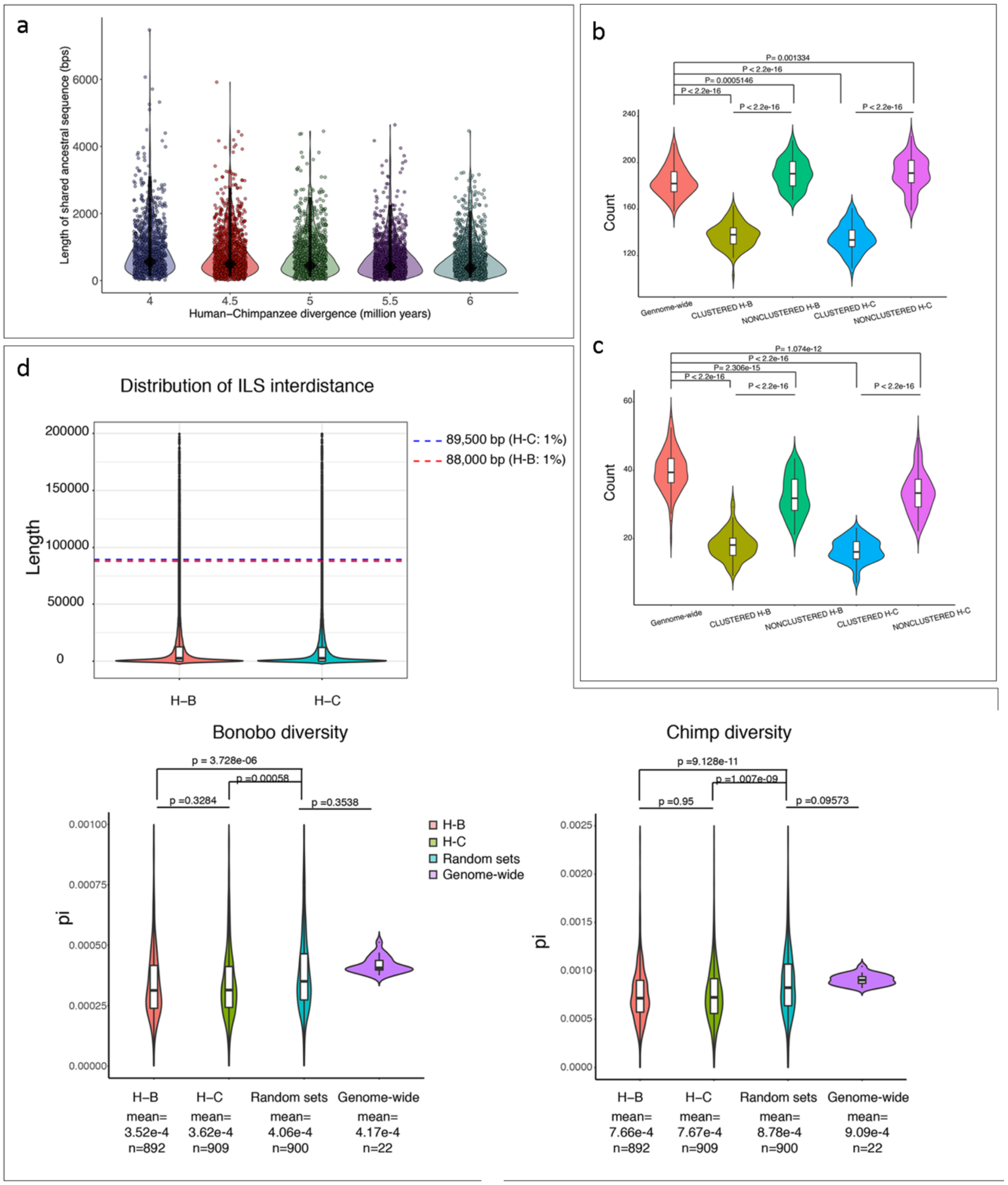

Supplementary Data Figure S24. Expected tract length of ILS with the topology ((human, chimpanzee), bonobo). a, A dot represents the expected length of a simulated ILS sequence under a Poisson process with given values for the sequence divergence time between human and chimpanzee, recombination rate, and generation time. 1,000 simulations were performed for each of the five different human–chimpanzee sequence divergence times. The black diamonds and vertical bars indicate the mean and 95% confidence intervals for ILS tract length (**Supplementary Data Table S25**). **b,** Clustered H-B/H-C ILS are less likely intersected with regulatory elements (ENCODE V3) with respect to genome-wide or non-clustered H-B/H-C. **c,** (Non)clustered H-B/H-C ILS less likely intersected with exons (RefSeq) with respect to genome-wide or non-clustered H-B/H-C. **d,** ILS deserts and reduced genetic diversity. Distribution of ILS deserts was defined as the top 1% of ILS deserts (top panel) for H-B (red) and H-C ILS (blue) regions. Genetic diversity (pi) is compared for bonobo (left) and chimpanzee (right panel) for H-B and H-C deserts to a randomly simulated set and the genome wide average based on autosomal regions. The box shows the first quartile to the third quartile. A vertical line within the box shows the median. The

whisker represents range. The boxplot was generated using the R package ggplot2 function geom_boxplot. Two-sample Wilcoxon test was used to calculate the P values in R.

Since bonobo noncoding regulatory DNA annotations are not available, we intersected both clustered and non-clustered ILS segments with both genes (RefSeq) and ENCODE (V3) regulatory regions based on human annotation.

Using human gene annotation (RefSeq GRCh38), we classify 1.37 Gbp (45.2%) of the genome as intragenic and 1.66 Gbp (54.8%) as intergenic. With respect to chimpanzee/human ILS, we find that 19,607 clustered H-B (total: 29,691) and 19,930 clustered H-C (total: 30,056) correspond to intergenic regions. Based on a null distribution (randomly choose 30,000 segments (500 bp) compute the mean 100 times) (mean=17,384.9), we find that both clustered H-B (19,607 [66%], empirical p=0) and H-C (19,930 [66%], empirical p=0) ILS are more likely to be located in the intergenic regions.

With respect to noncoding regulatory DNA, we considered the 926,536 annotated regulatory elements from ENCODE (V3) database and found that 4,070 clustered H-B and 4,083 clustered H-C are intersected with regulatory elements, respectively. Similarly, we find 13,728 non-clustered H-B and 13,772 non-clustered H-C intersect with regulatory elements, respectively. To ask whether the clustered H-C/H-B are more/less likely to intersect with the regulatory elements with respect to the genome-wide or non-clustered H-C/H-B, we randomly chose 1,000 segments from each type (clustered H-C/H-B, non-clustered H-C/H-B, and genome-wide) and calculated the number of intersections between the 1,000 segments and regulatory elements. We repeated this process 100 times and compared the distributions. We found that clustered H-B ($p<2.2e-16$)/H-C($p<2.2e-16$) segments are less likely to intersect with the regulatory elements with respect to genome-wide or the non-clustered H-B/H-C segments. Yet, interestingly, we found that non-clustered H-B (p=0.00005)/H-C(p=0.001) are more likely to intersect with the regulatory elements with respect to genome-wide (**Supplementary Data Fig. S24b**).

With respect to exons, we repeated the same process using RefSeq definitions. As we expected, the H-B/H-C are less likely to intersect with exons (RefSeq) no matter whether they are clustered or not. Of note, clustered H-B/H-C are less likely to intersect with exons with respect to the non-clustered H-B/H-C (**Supplementary Data Fig. S24c**).

We also searched for regions significantly depleted for ILS (ILS deserts) by calculating the inter-ILS distance and selecting regions within the lowest 1% of that distribution. We identified 892 and 909 ILS deserts (H-B and H-C, respectively). Next we estimated diversity (pi) in both chimpanzee and bonobo comparing it to the genome-wide average. We observed that both H-B and H-C ILS deserts show reduced genetic diversity although are not significantly different from each other. These results are consistent with these regions being targets of selective sweeps or background selection regions in the Pan lineage (**Supplementary Data Fig. S24d**). Thus, we intersected ILS deserts with regions identified by SweepFinder2 (above). We found 40 (p=0.29) and 41 (p=0.23)

bonobo selective sweep regions intersected with H-B and H-C desert regions, respectively; while 55 (p=0.17) and 45 (p=0.61) chimpanzee selective sweep regions intersected with H-B and H-C deserts, respectively. These data suggest that ILS deserts are not more likely to be associated with selective sweeps in bonobo and chimpanzee.

5.5 ILS interdistance simulation

The ILS events for chimpanzee–human and bonobo–human comparisons were projected onto GRCh38 and the genomic distance between regions of ILS was measured genome-wide. ILS interdistance was defined as the distance in base pairs between consecutive ILS events. To avoid inflation of distance estimates across centromeres, we estimated ILS interdistance for all p-arms and q-arms separately. To define a null distribution for ILS interdistance, we permuted the coordinates of ILS sets across the genome while controlling for the size of each ILS and low mappability regions (i.e., where no ILS discovery took place). We performed 400,000 permutations of the ILS coordinates using BEDTools (version 2.28.0). The observed interdistance was compared to the null interdistance to separate the clustered from non-clustered ILS events (**Fig. 3a** and **3b**).

5.6 Deeper phylogenetic ILS and selection

Based on above deeper phylogenetic ILS analysis, we revisited the different classes of ILS and tested whether there was evidence of clustered ILS segments as we had originally observed for chimpanzee, human, and bonobo. Then, we assessed whether those clustered segments showed evidence of positive selection (as well as balancing selection) and whether the clustered sites themselves overlapped more than expected by chance.

We compared the amount of overlap for H-C and H-B classified regions in the original callset and the reclassified ILS segments after inclusion of orangutan as an outgroup. As expected (**Supplementary Data Table S26**), almost all of the original ILS segments (90.9%, 86,342/94,964) overlapped the superset of ILS topologies when orangutan was included. However, the addition of gorilla and orangutan did lead to a reclassification of specific categories due to the presence of additional topologies. The overlap between H-C/H-B ILS topologies before and after inclusion was highly significant (Chi-square tests p<0.0001) as we would have expected.

Supplementary Data Table S26. The number of ILS in without orangutan and with orangutan datasets

	ILS	H-C	H-C*	H-B	H-B**	NON-ILS	Total
Without orangutan	94,964 (3.89%)	47,832 (1.96%)	47,832 (1.96%)	47,132 (1.93%)	47,132 (1.93%)	2,348,805 (96.11%)	2,443,769
With orangutan	886,657 (36.28 %)	26,182 (1.07%)	44,200* (1.81%)	26,056 (1.07%)	43,936** (1.80%)	2,355,112 (63.72%)	2,443,769
Overlapped	86,342 (90.92%)	25,051 (52.37%)	34,384 (71.88%)	25,168 (53.40%)	34,09 (72.33%)		

Based on an analysis of 3,818,646 segments where tree topology could be assigned.
* the number of ILS contain (O,((B,(C,H)),G)), (O,(((G,H),C),B)), (O,(((C,H),G),B)), (O,((B,G),(Cp,H))), and (O,(((C,G),H),B))
** the number of ILS contain (O,(((B,H),C),G)), (O,((B,(G,H)),C)), (O,(((B,H),G),C)), (O,((B,H),(C,G))), and (O,(((B,G),H),C))"

Next, we restricted the clustered analysis to high-confidence ILS segments (bootstrap ≥50) and first tested whether those inter-ILS distances were nonrandomly distributed when compared to the null (**Extended Data Fig. 7**). We considered the four most abundant ILS topologies, namely:

1) O-H: (orangutan,(((bonobo,chimp),gorilla),human)),
2) O-(H,G): (orangutan,((bonobo,chimp),(gorilla,human))),
3) H-B: (orangutan,(((bonobo,human),chimp),gorilla)),
4) H-C: (orangutan,((bonobo,(chimp,human)),gorilla))).

For each topology, we observe a characteristic cluster of ILS segments that deviate significantly from the null and are not randomly distributed in the genome. We note that the proportion of clustered ILS segments differs with older topologies (more ancient ILS) showing a greater fraction of clustered sites. For example, for the O-H and O-(H,G) topologies the proportion of clustered sites is ~32-34% while for H-B and H-C this fraction is 8-10%.

Next, we investigated whether we still observed the elevated dN/dS in clustered ILS. As before, we compared the observed dN/dS values for clustered sites against a simulated set where 1000 genes were chosen at random and a genome-wide distribution was created (**Supplementary Fig. 10**) by repeating the process 100 times to generate a null distribution (mean=0.263). Using a one sample t-test statistic, we observe a significant elevated mean dN/dS in both clustered H-C and H-B ($p < 2.2e-16$, mean=0.366) and in clustered O-H and O-G-H ($p < 2.2e-16$, mean=0.316) when compared to the null. The non-clustered H-C and H-B topologies remain insignificant ($p=0.45$, mean=0.264) although non-clustered O-H and O-G-H sites now show evidence of excess of amino acid replacement ($p < 2.2e-16$, mean=0.306) although that difference is more subtle and occurs within the last 5% of the null distribution.

Based on this phylogenetically deeper analysis of ILS, we grouped the four most abundant ILS topologies and repeated the inter-ILS distance clustering analysis. As expected, the clustering signal became stronger suggesting long-term maintenance of ILS over specific regions of the genome (**Supplementary Data Fig. S25**). A GO analysis[75] of the genes intersecting these combined data showed the most significant signals for immunity (e.g., glycoprotein (p=1.3E-25), immunoglobulin-like fold/ FN3 (p=2.4E-20)), but also genes related to the transporter function (e.g., transmembrane region (p=1.3E-25) and specifically calcium transport (p=3.7E-8)) (**Supplementary Table 53**). Among the former, the major histocompatibility complex (MHC) region is an exemplar (positive control) and we depict the depth and diversity of ILS topologies schematically over that region.

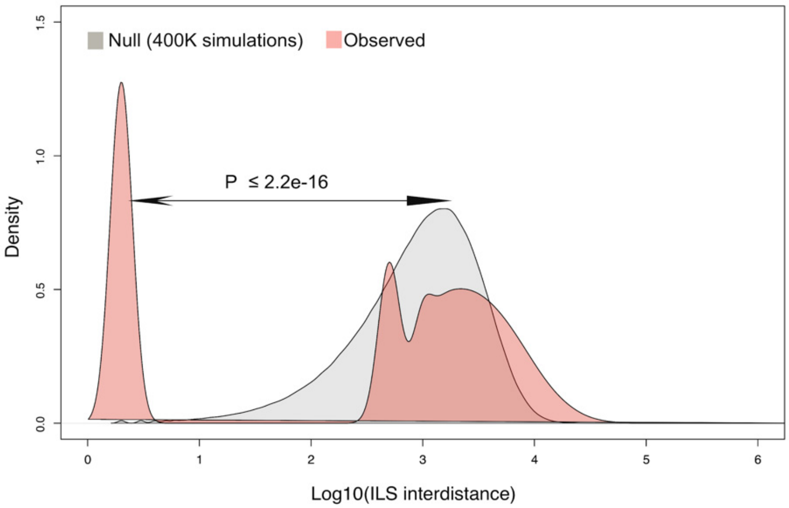

Supplementary Data Figure S25. Clustered ILS sites of main four ILS topologies. The distance between four adjacent main ILS segments (inter-ILS) (500 bp resolution) was calculated and the distribution was compared to a simulated expectation based on a random distribution. Two-sample Wilcoxon test was used to calculate the p-values in R.

We assessed whether there was any evidence of long-term balancing selection corresponding to regions of ILS based on genetic diversity. Here, we focused specifically on the 25,168 (H,C)B and 25,051 (H,B)C segments identified from our more extended ILS analysis (using orangutan as an outgroup as described above). We identified patterns of single-nucleotide variant (SNV) diversity (GATK) genome-wide by mapping WGS data from 10 bonobos and 10 chimpanzees to human GRCh38 (**Supplementary Table 42**). We used these data to calculate genetic diversity (pi) for the bonobo and chimpanzee population and assess stratification using dxy (an absolute measure of genetic divergence between incipient lineages) between bonobo and chimpanzee. We then compared patterns for H-B and H-C ILS segments, a matched randomly chosen subset and genome-wide.

Regions of long-term balancing selection are expected to have unusually high diversity within species and an excess of shared alleles between species. Previous analyses of the trans-species ABO polymorphisms have confirmed such sites through simulation

and suggested that sites of balancing selection are typically small (<4 kbp) due to the action of recombination, although this may in fact aggregate in specific regions[76,77]. We therefore calculated the pi and dxy diversity within 500 bp windows comparing clustered and non-clustered H-B/H-C ILS to a null set drawn from randomly selected genome segments (**Supplementary Fig. 11**).

In general, bonobo sites (H,B),C) sites show little difference between the clustered and non-clustered sites or the null expectation—diversity is exceedingly low in all cases consistent with previous population genetic analyses of this species. In contrast, non-clustered sites in chimpanzee show the greatest population genetic diversity and, in the case of (H,B),C) non-clustered ILS regions, show greater diversity than clustered regions. As expected, both clustered and non-clustered ILS show significantly higher dxy values when compared to the null, although clustered sites showing significantly higher values (**Supplementary Fig. 11**). These findings are consistent with the action of long-term balancing selection resulting in greater polymorphism and higher dxy between two pop/species possibly consistent with long-term maintenance of ancestral polymorphism within the ancestral Pan lineage*.* Because balancing selection is typically associated with noncoding regulatory DNA[78-80], we believe the observation of elevated dN/dS (positive selection) and balancing selection over the noncoding DNA are not mutually exclusive.

We intersected both clustered and non-clustered H-C and H-B 500 bp segments based on GRCh38 RefSeq annotation and assessed GO enrichment using DAVID[75]. Consistent with our previous observations, the segments are enriched for immunity-related genes (e.g., glycoprotein, and EGF-like domain, etc.) but also some signal for cell adhesion and motor function (e.g., microtubule motor activity, dynein heavy chain, domain-1, IQ motif and Laminin G domain, etc.) (**Supplementary Data Table S27**).

Supplementary Data Table S27. GO enrichment analysis of different classes of ILS segments overlapping with exons

	Term	Enrichment score	p_value
CLUSTERED ILS H-B (n=41) Overlapping exons	microtubule motor activity	1.21	9.40E-03
	SH3 domain	1.2	4.30E-02
CLUSTERED ILS H-C (n=36) Overlapping exons	extracellular matrix organization	2.51	3.00E-03
	Cell adhesion	2.21	3.30E-03
	Glycoprotein	1.61	8.10E-03
	Calcium/transmembrane region	1.31	1.00E-04
NON-CLUSTERED ILS H-B Overlapping exons H-B (n=765)	ATP-binding	5.05	9.30E-08
	ECM-receptor interaction	3.69	4.00E-07
	Dynein heavy chain, domain-1	3.54	2.20E-06
	SNF2-related	2.73	2.70E-05
	Laminin G domain	2.71	1.10E-08
	domain: Fibronectin type-III 3	2.55	1.90E-05
	von Willebrand factor, type A	2.39	1.00E-04
	Platelet Amyloid Precursor Protein Pathway	2.13	4.90E-05
	Epidermal growth factor-like domain	2.12	8.80E-07
	Glycoprotein	2.07	8.90E-04
NON-CLUSTERED ILS Overlapping H-C (n=806)	Pleckstrin homology-like domain	5.09	2.70E-06
	ATP-binding	3.65	2.00E-05
	EGF-like domain	2.92	4.10E-07
	Dynein heavy chain, domain-1	2.81	9.40E-05
	Rho guanyl-nucleotide exchange factor activity	2.8	1.30E-04
	WD40/YVTN repeat-like-containing domain	2.65	3.40E-06
	Extracellular matrix	2.49	5.80E-06
	Glycoprotein	2.42	6.50E-05
	IQ motif, EF-hand binding site	2.42	3.30E-05
	compositionally biased region: Cys-rich	2.13	5.80E-05

With respect to the observation of balancing selection, it should be noted that ~5% of the genes associated with ILS show evidence of changes in gene structure (frameshift, premature stop/start losses). For example, restricting our analysis to ILS exons, we observe 77 CDS changes in 51 genes, including stop/start loss. Among these, 18 occur in bonobo, 32 in chimpanzee, and 27 can be assigned to the ancestral Pan lineage (**Supplementary Data Table S28**).

Supplementary Data Table S28. Polymorphic gene disruption and ILS exons

chr	pos	ref	alt	Consequence	SYMBOL	EXON	Protein_ position	Amino_ acids	Lineage
chr1	24082032	T	TGGGGTCACCTTCCAGCCTTACCTTGCAGACCCGGGTGGGGATGGGCTGCTGAG	frameshift_variant	*MYOM3*	18//37	750	N//TQQPIPTRVCKVRLEGDPX	Chimp
chr1	152307613	C	A	stop_gained	*FLG*	3//3	2425	E//*	Chimp
chr1	152308813	CAT	C	frameshift_variant	*FLG*	3//3	2024	H//X	Chimp
chr1	152308819	C	G,CTG	frameshift_variant	*FLG*	3//3	2023	G//QX	Chimp
chr1	152311694	C	T	stop_gained	*FLG*	3//3	1064	W//*	Chimp
chr1	152312127	G	GCC	frameshift_variant	*FLG*	3//3	920	A//GX	Chimp
chr1	152312129	ATG	A	frameshift_variant	*FLG*	3//3	919	H//X	Chimp
chr1	155688246	A	AG	frameshift_variant	*YY1AP1*	1//10	73	P//PX	Chimp
chr1	159313957	G	A	stop_gained	*OR10J3*	1//1	235	Q//*	Pan
chr1	159314580	AC	A	frameshift_variant	*OR10J3*	1//1	27	V//X	Chimp
chr10	21556792	TTG	T	frameshift_variant	*MLLT10*	4//4	131	C//X	Pan
chr11	106746580	G	A	stop_gained	*GUCY1A2*	7//9	634	Q//*	Bonobo
chr11	120236675	T	A	start_lost	*POU2F3*	1//13	1	M//K	Pan
chr11	120236693	CT	C	frameshift_variant	*POU2F3*	1//13	7	A//X	Pan
chr11	130121749	GAGGAAGATGAA	G	frameshift_variant	*APLP2*	6//19	218-221	EEDE//X	Chimp
chr12	48528011	G	T	stop_gained	*OR8S1*	2//2	330	G//*	Chimp
chr12	92707153	A	T	stop_lost	*PLEKHG7*	2//2	174	*//Y	Bonobo
chr13	27988490	CA	C	frameshift_variant	*URAD*	1//2	49	F//X	Chimp
chr13	30713840	T	TGG	frameshift_variant&splice_region_variant	*ALOX5AP*	1//6	39	W//WX	Chimp
chr13	36283606	T	C	start_lost	*CCDC169-SOHLH2*	2//16	1	M//V	Pan
chr13	99201452	AACAC	A	frameshift_variant	*UBAC2*	1//7	14-15	KH//X	Chimp
chr14	20002693	CT	C	frameshift_variant&splice_region_variant	*OR4Q2*	2//3	176	T//X	Pan
chr14	21633911	C	T	stop_retained_variant	*OR10G2*	1//1	311	*	Bonobo
chr14	21633912	A	G	stop_lost	*OR10G2*	1//1	311	*//R	Pan
chr14	21634450	TA	T	frameshift_variant	*OR10G2*	1//1	131	I//X	Bonobo
chr14	67204575	ATG	A	frameshift_variant	*FAM71D*	5//9	133-134	DA//DX	Pan
chr14	67204578	C	CAT	frameshift_variant	*FAM71D*	5//9	134	A//AX	Pan
chr15	99729633	C	T	start_lost	*LYSMD4*	5//5	1	M//I	Pan
chr15	99729634	A	G	start_lost	*LYSMD4*	5//5	1	M//T	Pan
chr16	285429	C	CGGGGGCAGGTACTGGGGTCCAGGGGGAGGGGCAGCTGGAT	frameshift_variant	*PDIA2*	6//11	305	R//RGQVLGSRGRGSWMX	Chimp
chr16	1488466	A	G	start_lost	*PTX4*	1//3	1	M//T	Bonobo
chr16	67210136	C	CCTCTCACCAGGCAGCA,CCTCTCACCAAGCAGCA	frameshift_variant	*LRRC29*	3//7	18	G//VLPGERX	Chimp
chr19	3594926	C	CA	frameshift_variant	*TBXA2R*	4//4	378	M//IX	Pan
chr19	8308290	T	A	start_lost	*CD320*	1//5	1	M//L	Chimp
chr19	40035223	G	A	stop_gained	*ZNF780B*	5//5	546	Q//*	Bonobo
chr19	40035339	CCA	C	frameshift_variant	*ZNF780B*	5//5	506-507	CG//WX	Bonobo
chr19	40035496	G	A	stop_gained	*ZNF780B*	5//5	455	R//*	Bonobo
chr19	42509114	G	A	stop_gained	*CEACAM1*	9//9	526	Q//*	Pan
chr2	70819436	G	A	stop_gained	*CLEC4F*	3//7	63	Q//*	Bonobo
chr2	73700804	A	G	stop_lost	*NAT8B*	1//1	168	*//Q	Pan
chr2	73701259	T	C	stop_lost	*NAT8B*	1//1	16	*//W	Pan
chr2	206705854	G	A	stop_gained	*DYTN*	4//12	106	Q//*	Chimp
chr20	23491884	C	T	stop_gained	*CST8*	2//4	73	Q//*	Bonobo
chr3	31989826	GA	G	frameshift_variant	*ZNF860*	2//2	250	I//X	Bonobo
chr3	31990433	AAACCTTAC	A	frameshift_variant	*ZNF860*	2//2	452-454	KPY//X	Bonobo
chr3	31990444	GTGTAATGAGTGTGGCAAGACCTTCCATCACAATTCAGCCCTTGTAATTCATAAGGCAATTCATACTGGAGAGAAAC	G	frameshift_variant	*ZNF860*	2//2	456-481	CNECGKTFHHNSALVIHKAIHTGEKP//X	Bonobo
chr3	52807083	A	ACAGTCACAGTCACGCAGGATGGGTAAG	stop_gained&inframe_insertion	*ITIH3*	19//22	747	T//TVTVTQDG*A	Chimp
chr4	1644285	G	A	stop_gained	*FAM53A*	5//6	314	Q//*	Chimp
chr4	188091373	T	C	stop_retained_variant	*TRIML2*	7//7	438	*	Pan
chr5	141183092	T	TTG	frameshift_variant	*PCDHB16*	1//1	178	F//FX	Pan
chr5	141183093	CCG	C	frameshift_variant	*PCDHB16*	1//1	179	R//X	Pan
chr5	141183103	AT	A	frameshift_variant	*PCDHB16*	1//1	182	I//X	Pan
chr5	141183107	A	AG	frameshift_variant	*PCDHB16*	1//1	183	H//QX	Pan
chr6	32405084	C	T	stop_gained	*BTNL2*	2//7	94	W//*	Pan
chr6	32405085	C	T	stop_gained	*BTNL2*	2//7	94	W//*	Pan
chr6	32443909	A	G	stop_retained_variant	*HLA-DRA*	4//5	255	*	Pan
chr6	127807218	G	A	stop_gained&splice_region_variant	*THEMIS*	5//7	625	Q//*	Chimp

chr6	132552711	C	T	stop_gained	*TAAR8*	1//1	7	Q//*	Chimp
chr6	132553439	CAA	C	Frameshift_variant (Prufer reported this gene, but this site is poly in bonobo)	*TAAR8*	1//1	250	K//X	Pan
chr6	169668182	G	A	stop_gained	*WDR27*	5//26	154	R//*	Bonobo
chr7	2513246	CAGAT	C	frameshift_variant	*LFNG*	2//9	46-47	TD//X	Pan
chr7	87195121	A	ATTTGGTAAACTGTCATTAGAAT	stop_gained&frameshift_variant	*DMTF1*	20//20	755	D//DLVNCH*NX	Bonobo
chr7	100793973	T	TC	frameshift_variant	*ZAN*	43//48	2647-2648	-//X	Pan
chr7	123877138	C	T	stop_gained	*HYAL4*	5//5	477	R//*	Chimp
chr7	143935465	TC	T	frameshift_variant	*OR2F2*	1//1	78	V//X	Bonobo
chr7	143935552	T	A	stop_gained	*OR2F2*	1//1	107	L//*	Chimp
chr7	143936151	A	T	stop_gained	*OR2F2*	1//1	307	K//*	Bonobo
chr7	152019724	C	T	stop_gained	*GALNTL5*	10//10	419	R//*	Chimp
chr8	30144588	C	T	stop_gained	*MBOAT4*	1//3	5	W//*	Chimp
chr8	144423953	T	TCTCAGGGGCACTGCGGGGCTCCGCCTGGCTGG,A	stop_gained&frameshift_variant	*VPS28*	9//9	212	S//SQPGGAPQCP*X	Chimp
chr8	144423954	G	GGC	frameshift_variant	*VPS28*	9//9	212	S//CX	Chimp
chr9	122554162	A	G	stop_lost	*OR1N2*	1//1	331	*//W	Bonobo
chr9	122675217	CT	C	frameshift_variant	*OR1L3*	1//1	30	L//X	Chimp
chr9	122675289	C	T	stop_gained	*OR1L3*	1//1	54	R//*	Chimp
chr9	122675314	TC	T	frameshift_variant	*OR1L3*	1//1	62	F//X	Chimp
chrX	101162918	ATTCT	A	frameshift_variant	*CENPI*	21//21	741-742	HS//X	Pan
chrX	151648668	C	CTG	frameshift_variant	*PASD1*	9//16	228	P//PX	Chimp

In comparison to all genes in the genome, where we identify 3,384 such polymorphic variants (693 in bonobo, 1,233 in chimpanzee, and 1,458 in Pan lineage) resulting 1,990 gene disruptions, ILS exons (77/1,446 or 5.3%) are significantly enriched when compared to the genome-average (1.5% or 3,384/222,329) ($p < 0.00001$, chi-square test) (**Supplementary Data Table S29**). Interestingly, these results are consistent with long-term balancing selection for gene loss partially explaining the elevated dN/dS ratio, i.e., relaxed selection.

Supplementary Data Table S29. Distribution of polymorphic gene-disruption events in ILS exons versus genome

	bonobo	chimpanzee	pan	total
**ILS exons (1446*)**	18	32	27	77 (51**)
**Genome-wide exons (222329*)**	693	1233	1458	3384 (1990**)

*the number of exons for analysis
**the number of disrupted genes

6. Small structural variant (SV) analyses

6.1 Discovery and genotyping of SVs in bonobo, chimpanzee and gorilla

We used PBSV (https://github.com/PacificBiosciences/pbsv), Sniffles[81], and Smartie-sv[1] to detect insertions and deletions (>50 bp) in chimpanzee, bonobo, and gorilla genomes against the human genome (GRCh38), respectively. An initial set contained 61,078 insertions and 59,246 deletions based on comparisons to the human reference genome. Then, we selected SVs supported by Smartie-sv or at least two other callers, as well as removing the SVs located in tandem repeats. The bonobo-specific SVs only existed in

the bonobo genome but not in the chimpanzee and gorilla genomes. As expected, >80% of the differences are small (<1 kbp in length) with predictable modes at 300 bp and 6 kbp corresponding to Alu and L1 retrotransposition events, respectively (**Extended Data Fig. 3**). Next, we used Paragraph[82] to genotype all bonobo-specific SVs with 10 bonobo, 10 chimpanzee, and 7 gorilla WGS short reads[71]. We calculated FST to identify bonobo-specific fixed SVs. For each SV, if FST ≥ 0.8, we regarded it as a fixed SV. In total, we found 3,606 fixed insertion (3.3 Mbp) and 1,965 fixed deletion (2.36 Mbp) events in the bonobo lineage (**Supplementary Table 44**).

For SV genotyping, we downloaded high-coverage WGS for 10 bonobos, 10 chimpanzees, and 7 gorillas from the previous study[71] and mapped them to the human genome (GRCh38) with BWA (0.7.15). We applied SAMtools (1.9) to sort and fixmate the reads and picard to mark the duplication reads. Next, we used GATK (v3.7-0) to realign indels and SAMtools to remove the reads with mapping quality lower than 30. Finally, we generated 27 high-quality BAM files with coverage greater than 30, and then we used Paragraph to genotype all SVs with the 27 high-quality BAM files.

Likewise, for mobile element genotyping, we mapped 10 bonobo and 10 chimpanzee WGS to the bonobo and chimpanzee genomes, respectively; and we did mapping and filtering to generate high-quality BAM files as above described. We applied both Paragraph and SVTyper[83] to genotype mobile element deletions and used Paragraph to genotype MEIs and calculated the allele frequency (AF) for each MEI deletion/insertion.

6.2 SV annotations

We converted the 5,569 bonobo-specific fixed SVs into VCF format and used the Ensembl Variant Effect Predictor (VEP) to annotate the SVs. In addition, we also converted the SVs' human coordinates to the corresponding bonobo coordinates with liftOver, and then, we used BEDTools to intersect SVs and exons predicted from CAT or supported by our Iso-Seq. To reduce bias, we removed the SVs intersected with only single exon genes. Finally, we found 148 SVs intersected with coding/untranslated regions (UTRs)/splice regions.

We used IGV (http://software.broadinstitute.org/software/igv/) to assess the Iso-Seq coverage for *ADAR1* (**Supplementary Data Fig. S26**). We used minimiro (https://github.com/mrvollger/minimiro) to present the synteny relationship of *LYPD8* and *SAMD9*. Additionally, we used a whole-genome shotgun sequence detection (WSSD) short-read genotyping pipeline to estimate the copy number variations of the *LYPD8* and *SAMD9* regions. The WSSD genotyping results showed that *LYPD8* and *SAMD9* were deleted in the bonobo lineages but not in other great apes (**Extended Data Fig. 6**). Therefore, the short-read mapping and long-read assemblies consistently supported the *LYPD8* and *SAMD9* loss in bonobo.

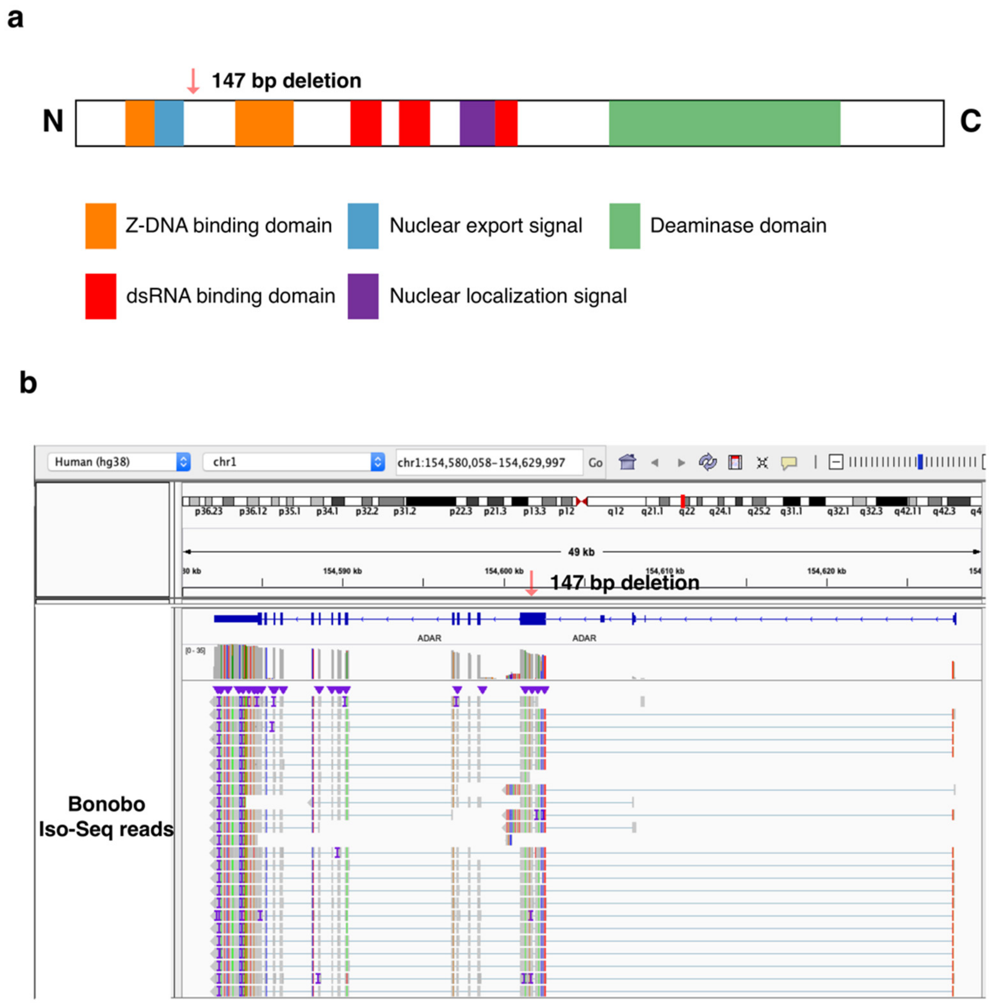

Supplementary Data Figure S26. Gene structure and Iso-Seq reads in *ADAR*. a, Gene structure of *ADAR* shows five different domains and a fixed deletion occurred near nuclear export signal. **b,** IGV screenshot of Iso-Seq reads supporting a deletion in exon2 of *ADAR*.

6.3 SV intersection with ILS regions

To assess SV enrichment or depletion in ILS regions, we intersected the fixed SVs with the 500 bp ILS regions. We found 267 fixed insertions and 34 fixed deletions in ILS regions (~5% genomic regions, 102.69 Mbp). We observed 3,604 fixed insertions and 1,965 fixed deletions in the whole genome (2,029.43 Mbp) and then by chi-square test. We found that fixed insertions are enriched (1.46-fold higher P-value < 0.001; chi-square) but fixed deletions are significantly reduced 0.34-fold lower (P-value < 0.001) in ILS regions. We further investigated the two major common repeat classes and found that both Alu (1.065-fold, P<0.001) and L1 (1.33-fold, P<0.001) elements are significantly higher within ILS regions. These data are consistent with ILS regions in general being under more relaxed selection (**Supplementary Table 43**). The statistical test (chi square test) was performed in R.

6.4 WSSD read-depth genotyping

We used a WSSD read-depth pipeline[84] to genotype all human (GRCh38) RefSeq genes with WGS data of human and nonhuman apes[71,85] and 21,336 genes were successfully genotyped. We calculated the ratio of bonobo copy number (CN) to human CN and the ratio of bonobo CN to chimpanzee CN. If the ratio was greater than 2, we regarded it as expansion; if the ratio was less than 0.5, we regarded it as contraction. If both the ratio of bonobo CN to human CN and the ratio of bonobo CN to chimpanzee CN were greater than 2, we regarded these genes as bonobo-specific expansions. If both the ratio of bonobo CN to human CN and the ratio of bonobo CN to chimpanzee CN were less than 0.5, we regarded these genes as bonobo-specific contraction (**Supplementary Tables 28-30**).

Next, we performed a gene ontology analysis on the bonobo CN changes relative to human or/and chimpanzee. Interestingly, among gene family contractions, all comparisons (bonobo vs. human, bonobo vs. chimpanzee, bonobo vs. chimpanzee, human) showed a significant enrichment (after BH correction) for the pathway *'Maturity onset diabetes of the young'.* For gene family expansions, we observe no significant enrichment for bonobo-specific differences. We observed signals for methylation-dependent chromatin silencing and progesterone when comparing bonobo expansion versus human and immunity differences when comparing bonobo gene family expansion versus chimpanzee (**Supplementary Data Table S30**). The genes underlying the latter, however, correspond to immunoglobulin genes and are often difficult to entangle from somatic variation (VDJ recombination) as opposed to strictly germline differences. Moreover, bonobo–human differences are driven by clustered gene families (i.e., likely single events or a series of mutational events driven by recombination), and thus, these differences are less likely to be functionally informative.

Supplementary Data Table S30. GO enrichment analysis of gene family contractions and expansion in bonobo compared to human and chimpanzee

Term	Overlap	P-value	Adjusted P-value	Genes	Gene_set	Type	Species compared
Maturity onset diabetes of the young	8/26	9.69E-05	0.03	*HHEX;BHLHA15;MAFA;MNX1;INS;NKX2-2;NEUROG3;FOXA2*	KEGG_2019_Human	Contraction	chimp and human
methylation-dependent chromatin silencing (GO:0006346)	4/11	4.43E-06	0.02	*MBD3L4;MBD3L5;MBD3L2;MBD3L3*	GO_Biological_Process_2018	Expansion	human
Progesterone-mediated oocyte maturation	7/99	1.11E-04	0.03	*SPDYE2B;SPDYE2;SPDYE1;SPDYE16;SPDYE3;SPDYE6;SPDYE5*	KEGG_2019_Human	Expansion	human
Fc receptor mediated stimulatory signaling pathway (GO:0002431)	5/135	8.02E-06	0.0037	*IGLV6-57;IGLV3-21;IGLV1-44;IGLV7-43;IGLV3-19*	GO_Biological_Process_2018	Expansion	chimp
regulation of protein processing (GO:0070613)	5/128	6.18E-06	0.0039	*IGLV6-57;IGLV3-21;IGLV1-44;IGLV7-43;IGLV3-19*	GO_Biological_Process_2018	Expansion	chimp
Fc-gamma receptor signaling pathway (GO:0038094)	5/134	7.73E-06	0.0039	*IGLV6-57;IGLV3-21;IGLV1-44;IGLV7-43;IGLV3-19*	GO_Biological_Process_2018	Expansion	chimp
(GO:0002455)	5/125	5.51E-06	0.004	*IGLV6-57;IGLV3-21;IGLV1-44;IGLV7-43;IGLV3-19*	GO_Biological_Process_2018	Expansion	chimp
(GO:0038096)	5/133	7.46E-06	0.0042	*IGLV6-57;IGLV3-21;IGLV1-44;IGLV7-43;IGLV3-19*	GO_Biological_Process_2018	Expansion	chimp
complement activation, classical pathway (GO:0006958)	5/123	5.09E+06	0.0043	*IGLV6-57;IGLV3-21;IGLV1-44;IGLV7-43;IGLV3-19*	GO_Biological_Process_2018	Expansion	chimp
regulation of immune effector process (GO:0002697)	5/114	3.50E-06	0.0045	*IGLV6-57;IGLV3-21;IGLV1-44;IGLV7-43;IGLV3-19*	GO_Biological_Process_2018	Expansion	chimp
regulation of acute inflammatory response (GO:0002673)	5/121	4.70E-06	0.0048	*IGLV6-57;IGLV3-21;IGLV1-44;IGLV7-43;IGLV3-19*	GO_Biological_Process_2018	Expansion	chimp
regulation of humoral immune response (GO:0002920)	5/113	3.36E-06	0.0058	*IGLV6-57;IGLV3-21;IGLV1-44;IGLV7-43;IGLV3-19*	GO_Biological_Process_2018	Expansion	chimp
regulation of complement activation (GO:0030449)	5/109	2.81E-06	0.0072	*IGLV6-57;IGLV3-21;IGLV1-44;IGLV7-43;IGLV3-19*	GO_Biological_Process_2018	Expansion	chimp
Fc receptor signaling pathway (GO:0038093)	5/183	3.48E-05	0.0137	*IGLV6-57;IGLV3-21;IGLV1-44;IGLV7-43;IGLV3-19*	GO_Biological_Process_2018	Expansion	chimp
regulation of protein activation cascade (GO:2000257)	5/108	2.68E-06	0.0137	*IGLV6-57;IGLV3-21;IGLV1-44;IGLV7-43;IGLV3-19*	GO_Biological_Process_2018	Expansion	chimp
Fc-epsilon receptor signaling pathway (GO:0038095)	5/182	3.40E-05	0.0144	*IGLV6-57;IGLV3-21;IGLV1-44;IGLV7-43;IGLV3-19*	GO_Biological_Process_2018	Expansion	chimp
receptor-mediated endocytosis (GO:0006898)	5/188	3.96E-05	0.0144	*IGLV6-57;IGLV3-21;IGLV1-44;IGLV7-43;IGLV3-19*	GO_Biological_Process_2018	Expansion	chimp
serine-type peptidase activity (GO:0008236)	5/220	8.35E-05	0.0481	*IGLV6-57;IGLV3-21;IGLV1-44;IGLV7-43;IGLV3-19*	GO_Molecular_Function_2018	Expansion	chimp

Term: Gene classes enriched; p-value: p-value based on Fisher's test; Overlap: number of genes in the tested set overlapping with the gene category; Adjusted p-value: Benjamini-Hochberg adjusted p-value; Genes: Name of the genes in the overlap; Gene set: Gene ontology class; Type: specifies if the gene set tested is an expansion or a contraction; Species compared: Indicates if the expansion/contraction in bonobo is related to human or chimpanzee.

6.4.1 Comprehensive and systematic read-depth analysis of bonobo/chimpanzee/pan-specific expansion and contraction with HiFi read validation

We performed a genome-wide analysis of gene expansions in both the bonobo and chimpanzee lineages. First, as we described above, we identified copy number expansions and contractions in the *Pan* lineage and classified these as bonobo-specific, chimpanzee-specific, or shared (*Pan*-specific), compared to other hominids. This classification was based initially on short-read Illumina WGS mapping (WSSD) from 27 ape genomes (**Supplementary Table 42**) to the human reference to generate an assembly-independent assessment of copy number in order to focus on species-specific expansions as opposed to polymorphisms. Species-specific or *Pan*-specific events were subsequently confirmed orthogonally by read-depth analysis using the long reads and analysis of whole-genome and targeted long-read assemblies (HiFi and CLR) requiring a diploid CN difference of at least 2. We focused on regions likely to contain genes based on Iso-Seq annotation or by Liftoff analyses (GCA_009914755.2, https://github.com/nanopore-wgs-consortium/CHM13). Liftoff v1.4.2 was performed with the parameters ‘ -flank 0.1 -sc 0.85 -copies’ against each target genome using GRCh38 GENCODE v35 annotations as the source in order to count the number of duplicated loci with corresponding transcript support for each gene in each assembly. To estimate number of assembled copies of each gene independent of Liftoff gene annotations, we aligned 2 kbp chunks of each assembly to GRCh38 with MashMap v2.0[86] and merged adjacent alignments, requiring at least 6.5 kbp of contiguous sequence at 95% sequence identity. The number of assembled macaque loci corresponding to each GENCODE gene model was summarized with BEDTools. Among protein-coding gene family expansions (GRCh38 GENCODE v35), we identified 42 bonobo-specific, 12 chimpanzee-specific, and 142 shared *Pan* expansion candidates. Similarly, we identified 13 bonobo-specific, 6 chimpanzee-specific, and 56 shared *Pan* contraction candidates. For each bonobo gene duplication resolved by long-read assembly, we aligned Iso-Seq data and assessed the number of transcripts to identify predominant isoforms and potential changes in the gene structure (**Supplementary Tables 26 and 27**).

As a final validation and to confirm their organization within the bonobo/chimpanzee genome, we selected five gene family expansions (*CLN3, EIF3C, RGL4, IGLV6-57, SPDYE16*) and four gene loss events (*IGFL1, SAMD9*, *TRAV4, CDK11A*) for experimental validation by FISH (**Supplementary Data Tables S31 and S32**). Fosmid probes (n=9) corresponding to human genomic data were isolated and hybridized against human, bonobo, chimpanzee, gorilla, and orangutan chromosomal metaphase spreads and interphase nuclei. Every hybridization was performed as a co-hybridization experiment combining one clone for expansion and one clone for contraction to be sure that the absence of signals expected for the contraction was due to a real absence of signals and not a technical artefact (**Extended Data Fig. 4**). This analysis confirmed all genome predictions (**Supplementary Data Table S32, Supplementary Data Fig. S17 and Supplementary Fig. 2**) providing the most comprehensive resource of chimpanzee and bonobo gene family expansions. It is noteworthy that three out of four tested gene

expansions show patterns of intrachromosomal interspersion and these are found adjacent to “core duplicons” (e.g., *NPIP* and *GUSBP*), which have been predicted to mediate the formation of interspersed SDs in humans.

Supplementary Data Table S31. Gene functions in expanded and contracted genomic regions

Class	Gene	Description	Function	Phenotype	Notes
Expansion	CLN3	CLN3 Lysosomal/Endosomal Transmembrane Protein, Battenin	This gene encodes a protein that is involved in lysosomal function.	LOF causes neurodegenerative diseases commonly known as Batten disease or collectively known as neuronal ceroid lipofuscinoses (NCLs).	adjacent to NPIP
Expansion	EIF3C	Eukaryotic Translation Initiation Factor 3 Subunit C	EIF3C (Eukaryotic Translation Initiation Factor 3 Subunit C) is a Protein Coding gene.	Diseases associated with EIF3C include Colon Squamous Cell Carcinoma.	adjacent to NPIP
Expansion	RGL4	Ral Guanine Nucleotide Dissociation Stimulator Like 4	This oncogene encodes a protein similar to guanine nucleotide exchange factor Ral guanine dissociation stimulator. The encoded protein can activate several pathways, including the Ras-Raf-MEK-ERK cascade.	Increased expression of this gene leads to translocation of the encoded protein to the cell membrane. RGL4 expression is significantly associated with a variety of tumor-infiltrating immune cells (TIICs), particularly memory B cells, CD8+T cells and neutrophils.	adjacent to GUSBP core duplicon
Expansion	IGLV6-57	Immunoglobulin Lambda Variable 6-57	Protein Coding gene.	no phenotype associated	adjacent to a deletion
Expansion	SPDYE16	Speedy/RINGO Cell Cycle Regulator Family Member E16	Protein Coding gene. Among its related pathways are Oocyte meiosis.	no phenotype associated	high-copy duplicon
Contraction	IGFL1	IGF Like Family Member 1	The protein encoded by this gene is a member of the insulin-like growth factor family of signaling molecules. The encoded protein is synthesized as a precursor protein and is proteolytically cleaved to form a secreted mature peptide. The mature peptide binds to a receptor, which in mouse was found on the cell surface of T cells.	Increased expression of this gene may be linked to psoriasis.	
Contraction	SAMD9	Sterile Alpha Motif Domain Containing 9	This gene encodes a sterile alpha motif domain-containing protein. The encoded protein localizes to the cytoplasm and may play a role in regulating cell proliferation and apoptosis.	Mutations in this gene are the cause of normophosphatemic familial tumoral calcinosis (autosomal recessive)	
Contraction	TRAV4	T Cell Receptor Alpha Variable 4	In a single cell, the T cell receptor loci are rearranged and expressed in the order delta, gamma, beta, and alpha.	no phenotype associated	11 kbp deletion
Contraction	CDK11A	Cyclin Dependent Kinase 11A	This gene encodes a member of the serine/threonine protein kinase family. Members of this kinase family are known to be essential for eukaryotic cell cycle control.	These two genes are frequently deleted or altered in neuroblastoma.	

Supplementary Data Table S32. FISH results for expansions and contractions of bonobo and/or Pan genomes

				Heat map predictions					FISH Results									
**Class**	**Gene**	**Fosmid Clones**	**Coords (hg38)**	**HSA**	**PPA**	**PTR**	**GGO**	**PPY**	**HSA**		**PPA**		**PTR**		**GGO**		**PPY**	
Expansion	CLN3	170215_ABC9_3_2_000041281300_M15	chr16:28479201-28516032	S	D	D	S	S	16p	Single	XVIp	Dup	XVIp	Dup	XVIp	Single	XVIp	Single
Expansion	EIF3C	172343_ABC9_3_5_000044010100_H14	chr16:28687256-28729352	D	D	D	S	S	16p	Dup#	XVIp	Dup	XVIp	Dup	XVIp	Single	XVIp	Single
Expansion	RGL4	171515_ABC9_3_5_000046184500_C13	chr22:23675621-23714508	S	D	D	S	S	1p, 9q, 22q	Dup$	Ip (weak), IXq (weak), XXIIq	Dup	Ip, Iqter, VIIpter, IXq, XIIq	Dup	Ip, IXq, XXIIq	Dup$	XIIq	Single
Expansion	IGLV6-57	ABC8-41202000I5	chr22:22178597-22214773	S	S/D	S	S	S	22q	Single	XXIIq	Single	XXIIq	Single	XXIIq	Single	Acrocentric chrs	Dup$
Expansion	SPDYE16	171515_ABC9_3_5_000043959400_P22	chr7:76507030-76545218	S/D	D	D	S/D	S/D	7q	Dup	VIIq	**Dup**	VIIq	**Dup**	VIIq	**Dup**	VIIq	Dup
Contraction	IGFL1	170215_ABC9_3_2_000043862300_J24	chr19:46195756-46232256	S	del	del/S	S	S	19q	Single	No signal	del	IXXq	Single	XIXq	Single	IXXq	Single
Contraction	SAMD9	ABC8-41156300P24	chr7:93082459-93118602	S	del	S	S	S	7q	Single	No signal	del	VIIq	Single	VIIq	Single	VIIq	Single
Contraction	TRAV4	ABC8-42078300A3	chr14:21716253-21749608	S	S/del	S	S	S	14q	Single	XIVq (weak)	del	XIVq	Single	XIVq	Single	XIVq(weak)	Single
Contraction	CDK11A	ABC8-41133000L6	chr1:1700902-1734122	D	del	S/del	D	S	1p	Dup#	No signal	del	No signal	del	Ip	Dup	Ip	Single

Polymorphic duplication tested in three human (HG00733, GM12813 and GM24385)
$ FISH results different from predictions
In bold highly duplicated pattern signals

6.4.2 EIF4A3 and EIF3C analysis with local assemble from HiFi reads

We targeted the *EIF4A3* region for complete assembly using HiFi sequence data and were able to reconstruct the complete locus in bonobo, chimpanzee, gorilla, and orangutan identifying five full-length gene copies (262 kbp total length) in chimpanzee and six copies in bonobo (310 kbp in bonobo)[87]. In both chimpanzee lineages, the gene families are organized head-to-tail in direct orientation (**Extended Data Fig. 5)**.

We used the high-quality sequence to generate an MSA and then constructed a phylogeny estimating that the initial *EIF4A3* gene duplication occurred in the ancestral lineage of chimpanzee and bonobo approximately 2.9 mya. The locus subsequently expanded before and after chimpanzee and bonobo speciation to create the multiple copies (**Fig. 2**).

Sequence analysis using GeneConv suggests independent gene conversion events in each lineage. A subset of these events correspond to a set of Pan-specific amino-acid changes in the basic ancestral structure of the single ancestral copy that are now common to only chimpanzee and human (**Extended Data Fig. 5**).

As an aside, we investigated the copy number of *EIF4A3* in other mammalian lineages. Specifically, we mapped (blat -stepSize=5 -minScore=1000 -repMatch=2253 -minScore=20 -minIdentity=0) human *EIF4A3* genomic sequence onto genome assemblies of mouse lemur (MicMur2), mouse (mm39), opossum (monDom5), cow (bosTau9), and dog (canFam5). In all other lineages we were able to identify only one copy of *EIF4A3* from each of the species suggesting that the expansion is specific to the *Pan* lineage.

Because of our discovery of a chimpanzee/bonobo expansion of the *EIF4A3* gene family, we focused on the *EIF3C* gene family expansion confirmed by FISH in both chimpanzee and bonobo. Unlike the *EIF4A3* gene family, which expanded in tandem, this locus expanded in an interspersed fashion along the short arm of chromosome XVI (phylogenetic group chromosome 16) likely as a result of its association with *NPIP*. We performed a similar phylogenetic reconstruction (see *EIF4A3* above) and found that while the initial duplication of this locus occurred ~5.01 mya, subsequent duplications occurred independently in the bonobo and chimpanzee lineages (<1.5 mya) (**Extended Data Fig. 5**).

6.5 Bonobo SVs and human-specific SVs

Our previous study[1] used the great ape long-read assemblies to assess human-specific SVs, but the bonobo genome was not included in that analysis. Therefore, we examined how many bonobo SVs overlapped human-specific SVs. We used BEDTools to intersect bonobo SVs and human-specific SVs, and we found 1,007 insertions and 999 deletions cannot be intersected. We then mapped the 2,006 (1,007+999) SV-flanking regions to the bonobo genome and found 986 insertions and 976 deletions could be split-mapped. Finally, we found 21 (1007-986) human-specific deletions and 23 (999-

976) human-specific insertions that showed the same pattern of insertion/deletion indicating that they were no longer human-specific events. Then, we used VEP to annotate the 44 (21+23) SVs and found five SVs were located near genes.

6.6 Lineage-specific SVs disrupting exons or regulatory elements with HiFi read validation

As we descried above, we applied three callers (PBSV, Sniffle, and Smartie-SV) based on a comparison of four genome assemblies (bonobo (Mhudiblu_PPA_v0), chimpanzee (Clint_PTRv2), gorilla (Kamilah_GGO_v0), and human (GRCh38)) to identify SVs and then extracted the bonobo-specific, chimpanzee-specific, and pan-specific SVs, i.e., shared between chimpanzee and bonobo. Using Paragraph[82], we next genotyped all SVs against Illumina WGS data available from 10 bonobos, 10 chimpanzees, and 7 gorillas[71,88]. Based on the genotypes, we calculated the Fst between populations and considered an event as fixed and lineage-specific if Fst >0.8 between populations from different species. The Ensembl VEP was applied[89] to annotate the SVs in order to identify SVs disrupting genes (**Supplementary Data Table S33**) as well as events affecting potential noncoding regulatory DNA. We validated all gene-disruption events by mapping HiFi sequence reads generated from the bonobo, chimpanzee, gorilla, and two human genomes back to GRCh38. Relatively few gene disruptions mediated by structural variation were discovered in the Pan lineage (**Supplementary Fig. 8**) and much more common were structural changes that led to a significant modification of protein structure (**Supplementary Data Fig. S27**).

Supplementary Data Table S33. The fixed ape SVs affecting exons

Lineage-specific	HUMAN-CHR	HUMAN-START	HUMAN-END	SV-TYPE	SIZE	ANNOTATION	GENE	WGAC	WSSD (SDA)	GENE ID	EXON	pLI
bonobo	chr1	154601820	154601966	DEL	147	inframe_deletion	*ADAR*	0	0	ENSG00000160710	2//15	9.91E-02
bonobo	chr1	248739523	248763827	DEL	24305	stop_lost	*LYPD8*	0	0	ENSG00000259823	1-7//7	NA
bonobo	chr11	63119193	63119261	DEL	69	inframe_deletion	*SLC22A24*	0	0	ENSG00000197658	3//10	3.09E-03
bonobo	chr3	195789477	195790190	DEL	714	inframe_deletion	*MUC4*	0	0	ENSG00000145113	2//25	5.45E-16
bonobo	chr7	93077971	93119434	DEL	41464	transcript_ablation	*SAMD9*	0	0	ENSG00000205413	1-3//3	5.21E-30
chimp	chr19	22316718	22316719	INS	84	inframe_insertion	*ZNF729*	84	0	ENSG00000196350	4//4	4.00E-01
chimp	chr9	113425411	113425412	INS	314	stop_gained	*C9orf43*	0	0	ENSG00000157653	10//14	2.27E-10
pan	chr1	248589569	248604503	DEL	14935	transcript_ablation	*OR2T10*	0	0	ENSG00000184022	1-2//2	7.10E-04
pan	chr16	3352155	3359732	DEL	7578	transcript_ablation	*OR2C1*	0	0	ENSG00000168158	1//1	3.46E-05
pan	chr18	11598534	11612147	DEL	13614	transcript_ablation	*SLC35G4*	2028	0	ENSG00000236396	1//1	NA
pan	chr19	41573735	41613036	DEL	39302	transcript_ablation	*CEACAM21*	0	0	ENSG00000007129	1-7//7	1.84E-04
pan	chr19	54076250	54076325	DEL	76	start_lost	*TARM1*	0	0	ENSG00000248385	1//5	1.57E-07
pan	chr19	55881568	55881569	INS	62	stop_gained	*NLRP4*	0	0	ENSG00000160505	10//10	3.45E-01
pan	chr19	57445296	57445376	DEL	81	inframe_deletion	*ZNF749*	0	0	ENSG00000186230	3//3	5.07E-02
pan	chr2	112900651	112935661	DEL	35011	transcript_ablation	*IL37*	2337	0	ENSG00000125571	1-5//5	5.98E-02
pan	chr21	30540154	30565904	DEL	25751	transcript_ablation	*KRTAP19-6*	0	0	ENSG00000186925	1//1	NA
pan	chr21	44681959	44681960	INS	60	inframe_insertion	*KRTAP12-1*	120	0	ENSG00000187175	1//1	NA
pan	chr22	36249298	36275666	DEL	26369	transcript_ablation	*APOL1*	0	0	ENSG00000100342	1-7//7	5.04E-04
pan	chr7	100990639	100991463	DEL	825	inframe_deletion	*MUC12*	400	0	ENSG00000205277	2//12	4.95E-61
pan	chrX	26194188	26194313	DEL	126	inframe_deletion	*MAGEB6*	126	0	ENSG00000157168	2//2	NA
pan	chrX	130215872	130215873	INS	72	inframe_insertion	*ZNF280C*	0	0	ENSG00000176746	14//19	9.99E-01
pan	chrX	141905678	141905679	INS	357	inframe_insertion	*MAGEC1*	0	0	ENSG00000056277	4//4	8.41E-02
gorilla	chr7	48278210	48278211	INS	90	stop_gained	*ABCA13*	0	0	ENSG00000155495	18//62	7.20E-04
gorilla	chr11	77085047	77085048	INS	400	stop_gained	*CAPN5*	0	0	ENSG00000179869	2//13	7.44E-02
gorilla	chr7	75765777	75790779	DEL	25003	transcript_ablation	*CCL26*	0	0	ENSG00000149260	1-4//4	9.85E-03
gorilla	chr18	13100505	13100506	INS	73	frameshift_variant	*CEP192*	0	0	ENSG00000006606	38//45	2.10E-08
gorilla	chr2	27101452	27101553	DEL	102	inframe_deletion	*CGREF1*	0	0	ENSG00000101639	6//6	5.30E-02
gorilla	chr3	97876142	97876213	DEL	72	inframe_deletion	*CRYBG3*	0	0	ENSG00000138028	4//22	5.56E-01
gorilla	chr18	22414776	22418480	DEL	3705	coding_sequence_variant	*CTAGE1*	0	0	ENSG00000080200	1//1	NA
gorilla	chr6	159232153	159232203	DEL	51	inframe_deletion	*FNDC1*	0	0	ENSG00000212710	11//23	2.28E-08
gorilla	chr15	56429178	56429179	INS	50	stop_gained	*MNS1*	0	0	ENSG00000164694	10//10	2.54E-21
gorilla	chrX	40623543	40626128	DEL	2586	coding_sequence_variant	*MPC1L*	0	0	ENSG00000232030	1//1	NA
gorilla	chr2	241096022	241096099	DEL	78	inframe_deletion	*MTERF4*	0	0	ENSG00000138587	4//7	NA
gorilla	chr21	46416323	46416385	DEL	63	inframe_deletion	*PCNT*	0	0	ENSG00000238205	30//47	3.12E-04
gorilla	chr21	13641501	13641502	INS	338	stop_gained	*POTED*	338	0	ENSG00000122085	11//11	3.34E-04
gorilla	chr21	46651843	46651844	INS	57	inframe_insertion	*PRMT2*	0	0	ENSG00000285231	7//7	2.66E-01
gorilla	chr19	35813201	35813202	INS	322	stop_gained	*PRODH2*	0	0	ENSG00000160299	1//11	4.15E-02
gorilla	chr19	35526935	35526936	INS	216	inframe_insertion	*SBSN*	0	0	ENSG00000166351	1//4	2.01E-04
gorilla	chr17	28364355	28364356	INS	313	stop_gained	*SEBOX*	0	0	ENSG00000160310	3//3	NA
gorilla	chr4	70366758	70366759	INS	78	inframe_insertion	*SMR3A*	0	0	ENSG00000250799	3//3	4.03E-01
gorilla	chr4	442521	442522	INS	84	inframe_insertion	*ZNF721*	0	0	ENSG00000189001	3//3	9.90E-02
gorilla	chr3	31990584	31990751	DEL	168	inframe_deletion	*ZNF860*	0	0	ENSG00000274529	2//2	NA

Coordinates based on human GRCh38 genome

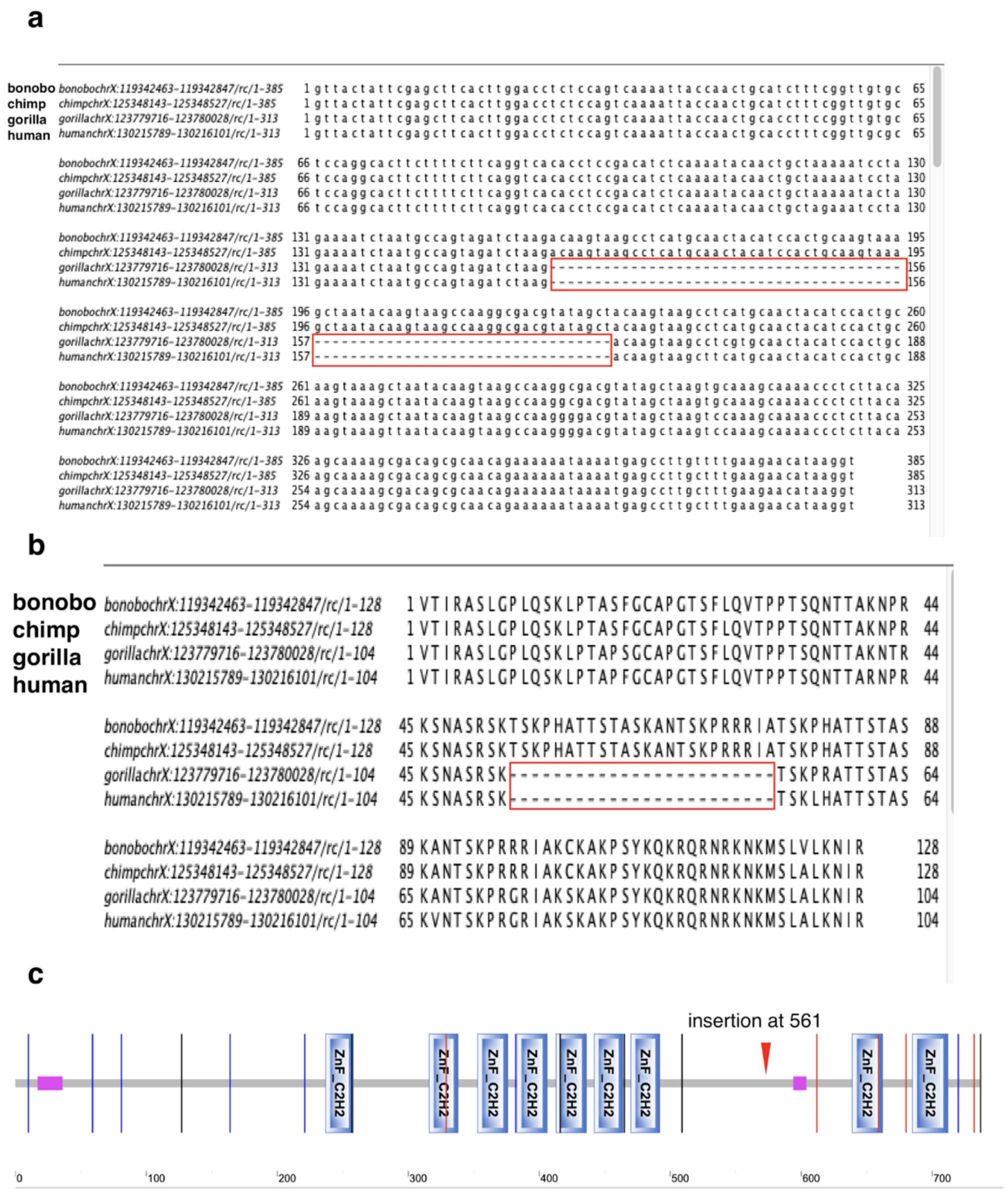

Supplementary Data Figure S27. A Pan-specific fixed genic insertion. a, A 72 bp insertion in the coding sequence of *ZNF280C* in chimpanzee and bonobo based on genomic sequence alignment among bonobo, chimpanzee, gorilla, and human. **b,** A 24 amino acid insertion specific to bonobo and chimpanzee. **c,** Insert occurs at position 561 in the ZNF280C protein.

We also considered the potential loss of noncoding regulatory elements by intersecting lineage-specific SVs with the ENCODE V3[90] catalog of functional elements in humans (**Supplementary Table 44**). We assigned regulatory elements to specific genes if they occurred within the body of the gene (UTR and intron) or the elements are located within 5 kbp downstream/upstream of the genes. We identified 662 disruptions (fixed insertions and deletions) of noncoding regulatory elements in the bonobo lineage and 356 events in the chimpanzee (**Supplementary Table 44**). Gene ontology enrichment analyses were performed using DAVID[75] for SVs associated with lineage-specific gene disruptions or loss of regulatory DNA. For bonobo-specific SVs, we find genes enriched

in membrane regions/topological domain: extracellular (p=2.4E-4), regulation (e.g., phosphate-binding region (p=7.8E-4), zinc finger domain (p=1.5E-2)), and neuron-related proteins (ANK repeats, (p=8.1E-3), synapse (p=4.4E-3), dopaminergic synapse (8.4E-2)). Bonobo contrasts with chimpanzee-specific SVs, which show an enrichment only in the cadherin pathway (p=6.10E-03). Gene loss in the ancestral Pan lineage (shared between chimpanzee and bonobo) show enrichments in postsynaptic membrane (p=1.2E-7), PDZ domain (p=4.5E-5), calcium transport (p=2.E-3), regulation (phosphate-binding region (p=3.8E-3), GTPase activator activity (p=5.4E-3) as well as coronary vasculature development (p=7.9E-2) and facial nerve structural organization (p=4E-2) (**Supplementary Table 46**). Although potentially interesting, it should be noted that the low number of events makes significance of all enrichments relatively modest.

6.7 Indel gene frameshift analyses with HiFi read validation

We also investigated potential gene loss as a result of indel mutation events (<50 bp) since such events are functionally equivalent to large SV events. We initially identified 323 frameshift mutations for 119 genes in the bonobo assembly based on comparison to human GRCh38. These events were identified from the CAT annotation of the bonobo assembly and were filtered to include only events on the default isoform (GENCODE's MANE_select isoform) for each gene. We validated all events using HiFi sequencing data from the same source (Mhudiblu) (**Supplementary Data Table S34**). This was done by using the HiFi data to call variants using FreeBayes and check for consistency in variant calls. As a control, we also analyzed HiFi data from two humans (Yoruban and Puerto Rican samples) and found that only four of these variants were also identified as a frameshift in at least one of the two humans. We excluded these from subsequent analysis. In order to define lineage specificity, we identified frameshift mutations in the chimpanzee and gorilla genomes as described above, and then compared those to the set of bonobo mutations. We identified 423 frameshifts corresponding to 186 genes in gorilla and 328 frameshifts corresponding to 149 genes in chimpanzee (**Supplementary Data Fig. S28**). We used HiFi sequencing data from an outgroup ape (orangutan) to validate lineage specificity. Finally, we also used the 27 WGS ape short reads to genotype these frameshifts by GATK and used the same criteria (Fst≥0.8) to identify the fixed frameshift events in each lineage (**Supplementary Data Fig. S29**). Please note that due to the inability to accurately map short-read Illumina data to duplicate genes, we limited the analysis to potential indels and frameshifts mapping outside of SDs (**Supplementary Data Fig. S28**)—i.e., to unique regions of the ape genome. Similar to the SV analyses, fixed indel events frequently occurred in genes tolerant to mutation or resulted in modifications to the carboxy terminus, with a few exceptions highlighted below (**Supplementary Data Fig. S29**).

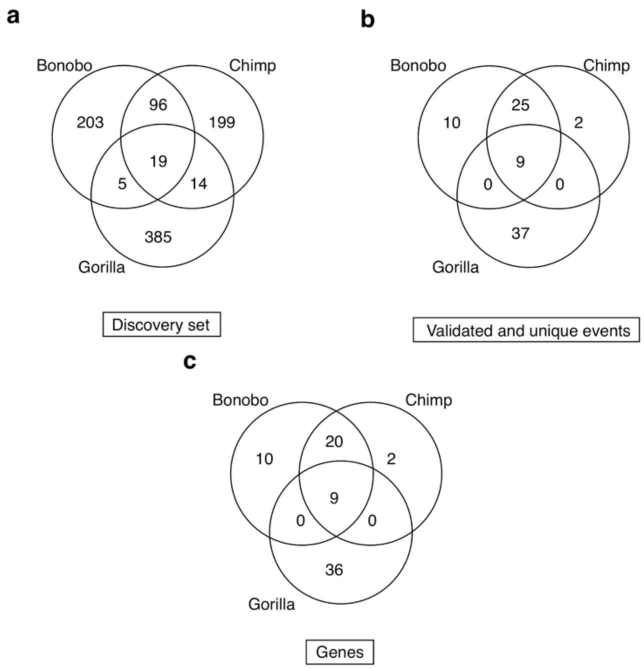

Supplementary Data Figure S28. Fixed indel mutations resulting in gene frameshifts. a, Frameshift mutation events discovered based on CAT annotation of individual ape genomes to human GRCh38. **b,** HiFi-validated frameshift mutations mapping to unique regions of the genome (outside of SDs) and that are fixed in each population based on analysis of Illumina WGS data from 27 ape genomes (**Supplementary Data Table S35**). Fixed mutations show Fst>0.8 for a given lineage. Comparisons between species were made by liftOver to GRCh38. **c,** Venn diagram of fixed lineage-specific and shared gene loss at the level of individual genes based on validated frameshifts in (b).

Supplementary Data Table S34. Fixed frameshifts in the ape lineages with HiFi and WGS validation

Lineage	Genes	Gene ID	Indel type	Human_indel_coords	PLI
bonobo+chimp+gorilla	*WDR78*	ENSG00000152763.17	Deletion	chr1:66924747-66924749	1.89E-03
bonobo+chimp+gorilla	*OR11L1*	ENSG00000197591.3	Deletion	chr1:247840962-247840963; chr1:247840964-247840965	5.79E-02
bonobo+chimp+gorilla	*SCIMP*	ENSG00000161929.15	Deletion	chr17:5210815-5210817	6.48E-03
bonobo+chimp+gorilla	*GNG14*	ENSG00000283980.1	Deletion	chr19:12688250-12688252	NA
bonobo+chimp+gorilla	*OCSTAMP*	ENSG00000149635.3	Deletion	chr20:46541566-46541568	7.13E-04
bonobo+chimp+gorilla	*OR2B2*	ENSG00000168131.4	Deletion	chr6:27911399-27911400; chr6:27911401-27911402	9.32E-03
bonobo+chimp+gorilla	*C12orf60*	ENSG00000182993.5	Deletion	chr12:14823553-14823554; chr12:14823555-14823556	4.82E-02
bonobo+chimp+gorilla	*ZNF843*	ENSG00000176723.10	Deletion	chr16:31436425-31436427; chr16:31436424-31436426	1.35E-03
bonobo+chimp+gorilla	*CMTM5*	ENSG00000166091.21	Deletion	chr14:23378759-23378761	0.32
bonobo	*MTF2*	ENSG00000143033.18	Deletion	chr1:93134088-93134089; chr1:93134092-93134093	1.00
bonobo	*ZNF780B*	ENSG00000128000.16	Deletion	chr19:40035339-40035340; chr19:40035342-40035343	1.28E-02
bonobo	*IGSF23*	ENSG00000216588.9	Deletion	chr19:44627544-44627546	0.13
bonobo	*C1GALT1C1L*	ENSG00000223658.8	Deletion	chr2:43675646-43675647; chr2:43675666-43675667	NA
bonobo	*CLEC4F*	ENSG00000152672.8	Deletion	chr2:70816097-70816099	3.63E-14
bonobo	*ZNF860*	ENSG00000197385.6	Deletion	chr3:31989825-31989826; chr3:31989827-31989828	NA
bonobo	*FBXW12*	ENSG00000164049.14	Deletion	chr3:48373676-48373678	1.27E-04
bonobo	*C3orf49*	ENSG00000163632.14	Deletion	chr3:63831756-63831757; chr3:63831759-63831760	3.59E-08
bonobo	*SLC10A5*	ENSG00000253598.3	Deletion	chr8:81694065-81694067	9.57E-07
bonobo	*SPATA31E1*	ENSG00000177992.10	Deletion	chr9:87887832-87887834	2.70E-02
chimp	*EXD3*	ENSG00000187609.16	Deletion	chr9:137354731-137354732; chr9:137354733-137354734	0.58
chimp	*OR52B6*	ENSG00000187747.2	Deletion	chr11:5581313-5581315	4.20E-03
gorilla	*ZNF404*	ENSG00000176222.9	Deletion	chr19:43874071-43874073	2.44E-05
gorilla	*EDDM13*	ENSG00000267710.9	Deletion	chr19:56272907-56272908; chr19:56272912-56272913	NA
gorilla	*GPX6*	ENSG00000198704.9	Deletion	chr6:28504364-28504366	7.36E-02
gorilla	*GPX6*	ENSG00000198704.9	Insertion	chr6:28504366-28504368	7.36E-02
gorilla	*LRRC27*	ENSG00000148814.18	Deletion	chr10:132348146-132348148	1.48E-04
gorilla	*ZNF556*	ENSG00000172000.7	Deletion	chr19:2878058-2878059; chr19:2878061-2878062	7.72E-04
gorilla	*OR56B1*	ENSG00000181023.8	Deletion	chr11:5737204-5737206	1.13E-09
gorilla	*TMEM63A*	ENSG00000196187.12	Deletion	chr1:225847054-225847056	0.59
gorilla	*PKD2L1*	ENSG00000107593.17	Deletion	chr10:100290026-100290028	2.98E-03
gorilla	*RPEL1*	ENSG00000235376.5	Deletion	chr10:103246471-103246472; chr10:103246473-103246474	1.14E-02
gorilla	*SLC43A1*	ENSG00000149150.9	Deletion	chr11:57494106-57494107; chr11:57494108-57494109	0.32
gorilla	*OR4D11*	ENSG00000176200.1	Deletion	chr11:59503832-59503833; chr11:59503834-59503835	3.85E-06
gorilla	*OR4S1*	ENSG00000176555.1	Deletion	chr11:48306829-48306831	1.58E-03
gorilla	*PLET1*	ENSG00000188771.5	Deletion	chr11:112248801-112248803	4.77E-04
gorilla	*MFAP5*	ENSG00000197614.11	Deletion	chr12:8655821-8655823	5.80E-02
gorilla	*FSCB*	ENSG00000189139.6	Deletion	chr14:44506117-44506118; chr14:44506115-44506116	NA
gorilla	*RNASE8*	ENSG00000173431.2	Deletion	chr14:21058203-21058204; chr14:21058179-21058180	NA
gorilla	*SLC28A2*	ENSG00000137860.12	Deletion	chr15:45253234-45253236	3.54E-06
gorilla	*FGF11*	ENSG00000161958.11	Deletion	chr17:7443125-7443126; chr17:7443127-7443128	6.35E-03
gorilla	*TYK2*	ENSG00000105397.14	Deletion	chr19:10365514-10365515; chr19:10365530-10365531	0.91
gorilla	*ZNF99*	ENSG00000213973.9	Insertion	chr19:22758766-22758769	2.55E-02
gorilla	*ZNF345*	ENSG00000251247.11	Deletion	chr19:36878273-36878274; chr19:36878276-36878277	0.55
gorilla	*SIGLEC6*	ENSG00000105492.16	Deletion	chr19:51531623-51531625	0.21

gorilla	*ZNF614*	ENSG00000142556.19	Deletion	chr19:52013150-52013152	0.10
gorilla	*CDH26*	ENSG00000124215.17	Deletion	chr20:60002854-60002856	2.90E-04
gorilla	*KRTAP25-1*	ENSG00000232263.1	Deletion	chr21:30289385-30289387	NA
gorilla	*KRTAP6-3*	ENSG00000212938.3	Deletion	chr21:30592673-30592674;<br>chr21:30592706-30592707	0.51
gorilla	*KRTAP21-3*	ENSG00000231068.1	Deletion	chr21:30718607-30718609	NA
gorilla	*ENTHD1*	ENSG00000176177.10	Deletion	chr22:39743679-39743681	3.89E-07
gorilla	*ZNF501*	ENSG00000186446.12	Deletion	chr3:44734461-44734463	0.30
gorilla	*TGM4*	ENSG00000163810.12	Deletion	chr3:44901666-44901668	2.67E-20
gorilla	*SLC9C1*	ENSG00000172139.15	Deletion	chr3:112286759-112286760;<br>chr3:112286774-112286775	0.54
gorilla	*COL25A1*	ENSG00000188517.16	Insertion	chr4:109302004-109302006	0.42
gorilla	*OR2B6*	ENSG00000124657.1	Deletion	chr6:27957942-27957944	3.78E-03
gorilla	*GJB7*	ENSG00000164411.12	Deletion	chr6:87284871-87284873	1.09E-06
gorilla	*RAET1G*	ENSG00000203722.8	Deletion	chr6:149916924-149916926	7.40E-02
gorilla	*TTF1*	ENSG00000125482.13	Deletion	chr9:132375929-132375931	8.56E-13
pan	*RFX8*	ENSG00000196460.14	Deletion	chr2:101422427-101422429	4.09E-09
pan	*IFIT1B*	ENSG00000204010.3	Deletion	chr10:89383444-89383446	3.66E-05
pan	*TACC2*	ENSG00000138162.19	Insertion	chr10:122087509-122087511	0.98
pan	*TACC2*	ENSG00000138162.19	Deletion	chr10:122087512-122087514	0.98
pan	*ANKK1*	ENSG00000170209.5	Deletion	chr11:113399454-113399456	4.65E-12
pan	*BLID*	ENSG00000259571.2	Deletion	chr11:122115651-122115653	NA
pan	*ACOD1*	ENSG00000102794.10	Deletion	chr13:76957962-76957963;<br>chr13:76957964-76957965	NA
pan	*ZNF324B*	ENSG00000249471.8	Deletion	chr19:58455695-58455697	0.51
pan	*ZNF324*	ENSG00000083812.12	Deletion	chr19:58471243-58471245	2.24E-02
pan	*CST9L*	ENSG00000101435.5	Deletion	chr20:23568338-23568340	7.76E-12
pan	*EFHB*	ENSG00000163576.18	Deletion	chr3:19918241-19918243	2.60E-10
pan	*EFHB*	ENSG00000163576.18	Insertion	chr3:19918243-19918245	2.60E-10
pan	*FBXW12*	ENSG00000164049.14	Deletion	chr3:48372835-48372836;<br>chr3:48372837-48372838	1.27E-04
pan	*EBLN2*	ENSG00000255423.1	Deletion	chr3:73062243-73062245	NA
pan	*IFT80*	ENSG00000068885.15	Deletion	chr3:160258555-160258556;<br>chr3:160258557-160258558	0.17
pan	*KIF4B*	ENSG00000226650.6	Deletion	chr5:155013914-155013916	1.16E-24
pan	*KIF4B*	ENSG00000226650.6	Insertion	chr5:155013919-155013921	1.16E-24
pan	*TAAR2*	ENSG00000146378.6	Deletion	chr6:132617345-132617347	5.95E-06
pan	*GALNTL5*	ENSG00000106648.14	Deletion	chr7:151982991-151982992;<br>chr7:151982993-151982994	3.04E-16
pan	*GALNTL5*	ENSG00000106648.14	Deletion	chr7:151987221-151987223	3.04E-16
pan	*DMRT3*	ENSG00000064218.5	Deletion	chr9:990499-990501	8.17E-03
pan	*DMRT3*	ENSG00000064218.5	Insertion	chr9:990501-990503	8.17E-03
pan	*SPATA31E1*	ENSG00000177992.10	Deletion	chr9:87887765-87887767	2.70E-02
pan	*ZNF404*	ENSG00000176222.9	Deletion	chr19:43873440-43873441;<br>chr19:43873443-43873444	2.44E-05
pan	*SMR3A*	ENSG00000109208.5	Deletion	chr4:70362131-70362132;<br>chr4:70362129-70362130	0.40

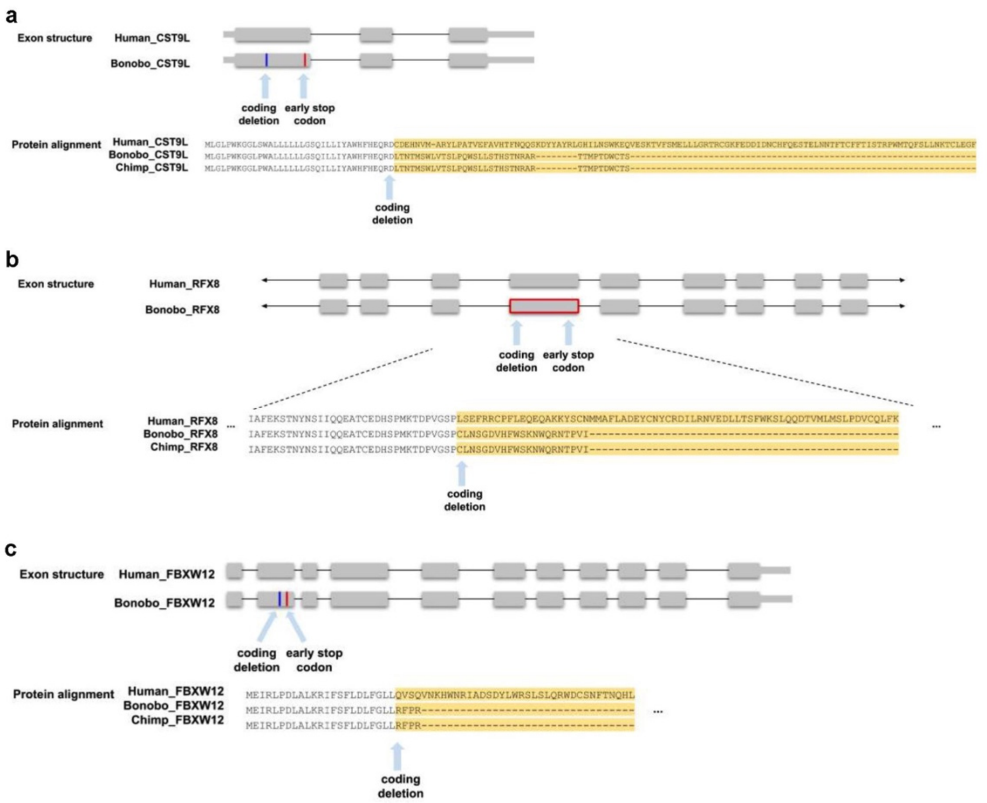

Supplementary Data Figure S29. Fixed gene-disrupting indels in the *Pan* lineage. a, 1 bp deletion in *CST9L* leads to a premature stop codon, event fixed in bonobo and chimpanzee. **b,** 1 bp deletion in *RFX8* leads to a premature stop codon, fixed in bonobo and chimpanzee. **c,** 1 bp deletion in *FBXW12* leads to a premature stop codon, fixed in bonobo and chimpanzee.

7. Mobile element insertion (MEI) analyses

7.1 Transposable elements in Mhudiblu_PPA_v0 versus other primates

We analyzed and compared repeat content of the Mhudiblu_PPA_v0 assembly using a local installation of RepeatMasker (RepeatMasker-Open-4.1.0; accessed March 2020) and the Dfam3 repeat library. We categorized common elements into broad (DNA transposons, LTR transposons, non-LTR transposons), as well as more specific, categories (e.g., LINE/L1, LINE/L2, etc.). We classified full-length MEIs from RepeatMasker output and a customized python script. We defined full-length Alu repeats within a start position of no less than 4 bp from the 5' end and an end position not shorter than 267 bp; full-length LINE-1 elements were at least 6000 bp; full-length ERV elements as ≥7000 bp with two flanking similar LTR elements around the internal ERV sequence; full-length SVA elements as variable in total bp but no less than 50 bp from the 5' end; and an end position no greater than 50 bp from the 3' end of the SVA consensus sequence.

The lineage specificity of full-length Alu insertions in both the bonobo (Mhudiblu_PPA_v0) and chimpanzee (Pan troglodytes; Clint_PTRv2; from NCBI) genomes was determined by extracting 600 bp of 5' and 3' flanking unique sequences

adjacent to each element and comparison to other primate genomes in a sequential BLAT: human (*Homo sapiens;* GRCh38) followed by the chimpanzee or bonobo genomes. We determined lineage specificity by assessing the presence or absence in the target genomes.

The lineage specificity of full-length L1, ERV, and SVA elements was determined by a liftOver analysis of the full-length elements that failed to find syntenic coordinates in the chimpanzee genome. We assigned lineage-specific Alu and full-length LINE elements to subfamilies using Alu element subfamily analysis. COSEG was applied to the lineage-specific Alu insertions obtained from both the bonobo and chimpanzee genome assemblies to determine the subfamily composition. Briefly, Alu and L1 insertions determined to be lineage-specific were aligned via Crossmatch (www.phrap.org/phredphrapconsed.html) with the default settings, then analyzed via COSEG (www.repeatmasker.org/COSEGDownload.html) to determine subfamily structure. The dataset was aligned against the AluY and L1PA2 consensus sequences, respectively. COSEG was then used to group subfamilies. The middle A-rich region of the AluY consensus sequence was excluded from analysis when determining subfamilies, whereas tri- and di-segregating mutations were considered. A group of ten or more identical sequences was considered a separate subfamily. The resulting subfamilies from each assembly were compared for both the Alu and L1 analyses. A network analysis of all subfamilies for both Alu and L1 identified by COSEG was created by uploading the source and target subfamily information into Gephi (v0.9.1).

Subfamily determination for PtERV subfamilies was determined by analyzing the lineage-specific bonobo insertions (previously defined above) by performing a cross_match analysis of all of the insertions compared to one another. The sequence that best described the dataset was then used as a consensus sequence for a COSEG analysis. The resulting analysis gave two subfamilies, which were then split into five subfamilies based on divergence clustering and the pattern of flanking LTR and internal sequence.

SVA subfamilies were determined by analyzing all full-length SVA_D insertions (previously defined above), as these were most likely to contain SVA_PtA, and therefore lineage-specific insertions. Subfamily determination for SVA subfamilies was determined by analyzing the insertions by performing a cross_match analysis of all of the insertions compared to one another. The sequence that best described the dataset in terms of score and length was then used as a consensus sequence for a COSEG analysis.

Of the 774,209 full-length Alu insertions found in the bonobo genome, 3,342 were lineage-specific after a BLAT filter against the human genome, while 1,548 Alu insertions remained after an additional BLAT step against the chimpanzee genome (**Supplementary Data Table S35**). This number is comparable to the 1,497 lineage-specific Alu elements found in the chimpanzee genome with the same pipeline (data not shown).

Supplementary Data Table S35. Lineage-specific expansion of transposable elements in selected primates

Repeat Class	Total full-length	Lineage-specific	Polymorphic	Subfamilies*
Alu	774,209	1,548	346	5/5
LINE1	6,579	487	214	5/5
SVA	1,783	745	336	1/5
PtERV	115	41	3	0/5

*Generated from lineage-specific insertions; the denominator indicates the number of subfamilies discovered, while the nominator indicates the number of subfamilies with all members found exclusively in bonobo

The 1,548 lineage-specific Alu insertions from bonobo were analyzed via COSEG to produce a network of five Alu subfamilies, four of which were most closely identified as AluY subfamilies, while one was identified as an AluSx subfamily (**Extended Data Fig. 3**). It is likely that older Alu insertions were inadvertently kept in this pipeline, while the AluY subfamilies correspond to bonobo-specific expansions. However, of these five, two bonobo-specific subfamilies were already defined, previously called AluY_p1 and AluY_p2[91]. Here, we discovered a new AluY_p2 subfamily, which differs from the original AluY_p2 by three nucleotides. We have named this new subfamily AluY_p2a and renamed the original AluY_p2 subfamily AluY_p2b_Prufer (**Supplementary Data Fig. S30a**).

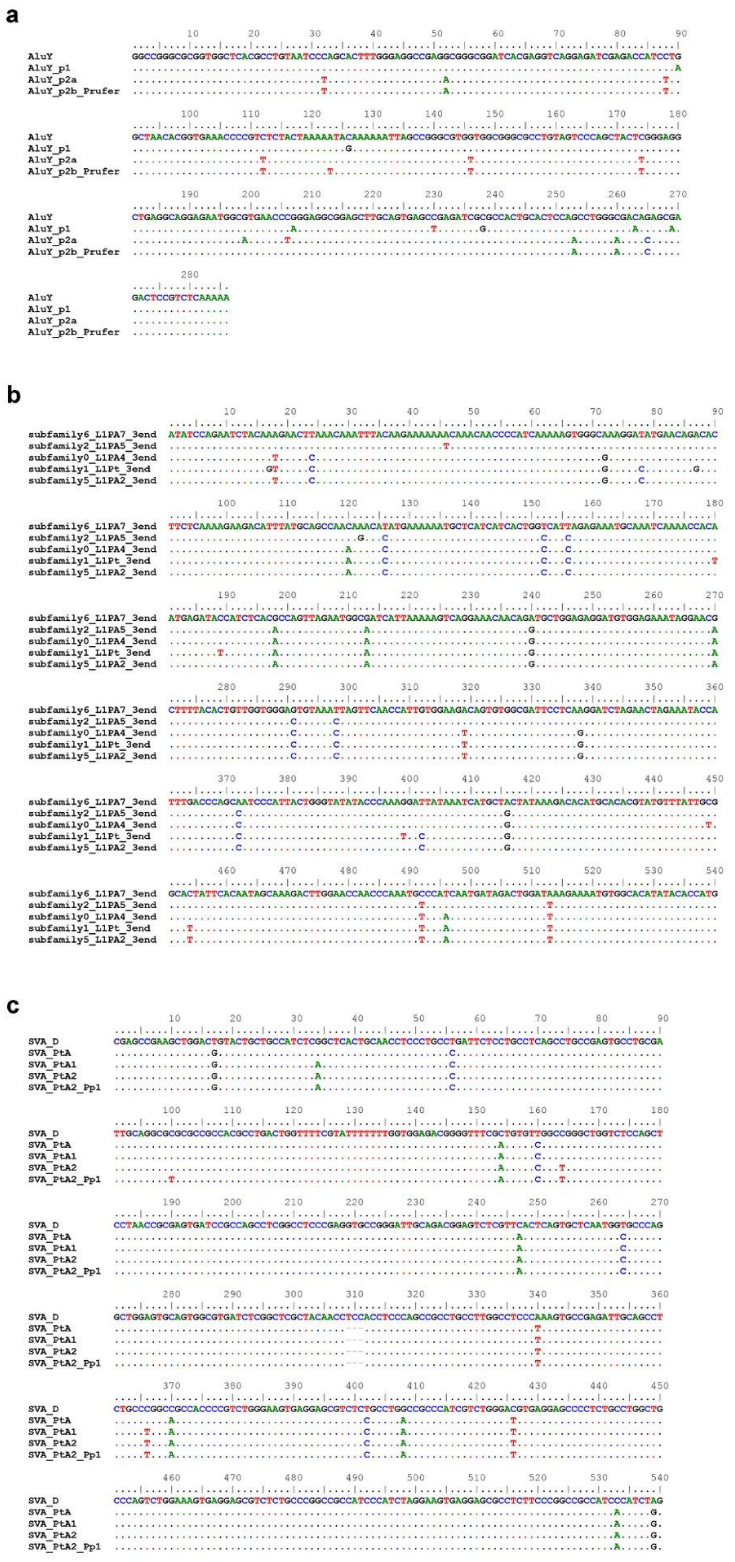

Supplementary Data Figure S30. MSA for (a) Alu, (b) LINE1 (3' end), and (c) SVA elements.

These low numbers contrast the large amount of lineage-specific Alu insertions and corresponding COSEG-defined subfamilies found in the squirrel monkey[92], baboon[93], rhesus (GenBank assembly accession GCF_003339765.1), and human genomes, indicating a reduction of Alu activity in the bonobo lineage. A similar contraction is observed in the chimpanzee genome as well.

After merging split LINE1 sequences in the bonobo assembly, 487 full-length LINE1 sequences were analyzed (**section 7.2**). These full-length L1 elements were analyzed via COSEG for subfamily composition as they did not liftOver to the chimpanzee genome, and most likely represented lineage-specific insertions. When using the L1PA2 3' end as the consensus sequence for COSEG subfamily analysis, seven consensus sequences were produced. Due to limited sequence differences (one nucleotide difference), three subfamilies were collapsed into one, giving a total of five L1 subfamilies (**Supplementary Data Fig. S31a**). Following RepeatMasker identification of the consensus sequences, the majority of the 487 L1 sequences identified most closely to L1PA2 or L1Pt subfamilies. Three subfamilies are most closely identified as L1PA4, L1PA5 and L1PA7, which comprise 118 of the 487 full-length L1 insertions. These subfamilies may have persisted but might not represent lineage-specific L1 subfamily expansions, given the linear evolution of the LINE1 family in primates (e.g., many of these older elements may represent instances that have been deleted via recombination in the chimpanzee reference and therefore do not liftOver). Consistent with the linear expansion of LINE1 elements, the network of COSEG subfamilies also presents itself in a linear fashion.

a

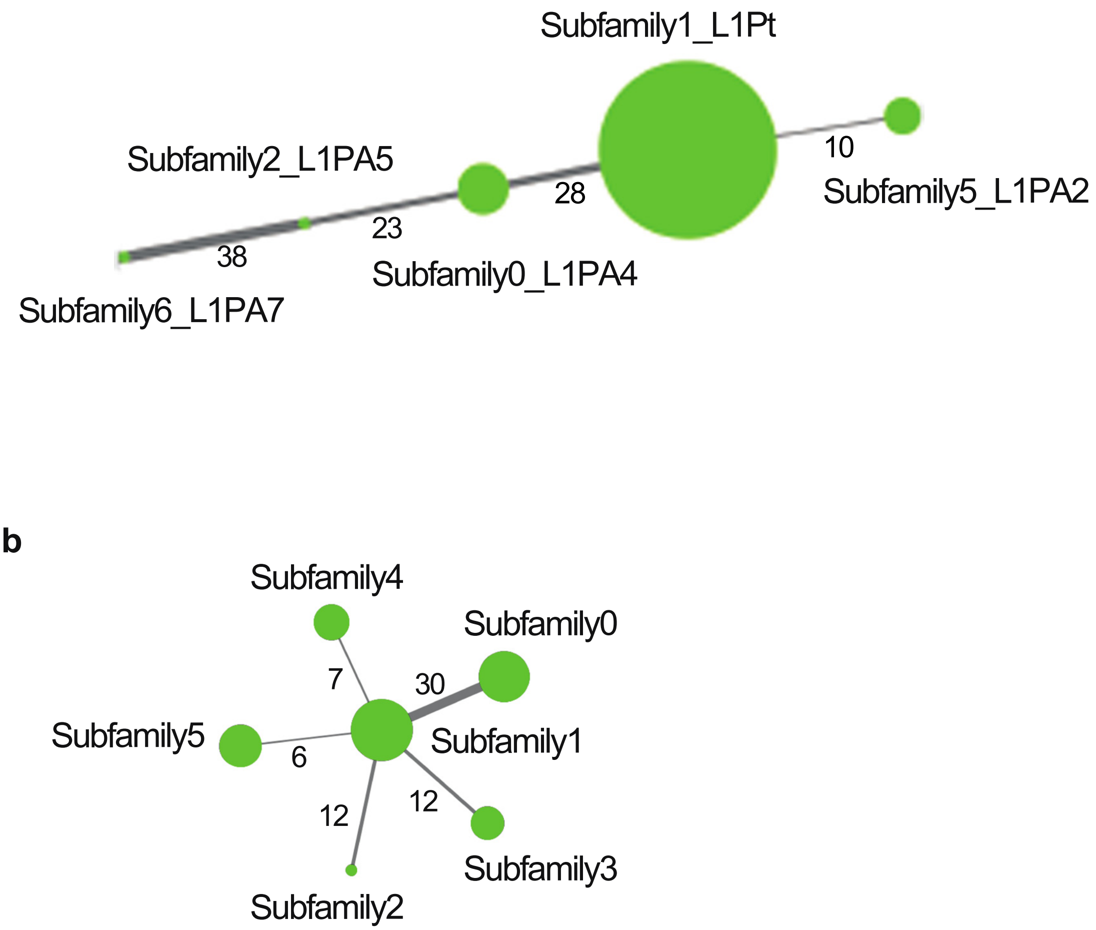

Supplementary Data Figure S31. Transposable element expansion in the bonobo lineage. Subfamily network analyses for **a,** full-length LINE1 sequences using the 3' L1PA2 consensus sequence and **b,** 5' L1PA2 consensus sequence. Related subfamilies are connected by lines. The thickness and number shown on the line reflect the number of mutations occurring between connected nodes. The size of each node corresponds to the relative number of elements in the subfamily indicated.

Within the network, subfamily1 was an exact match to L1Pt (**Supplementary Data Fig. S31a**). Given that three of the five L1 subfamilies belong to older L1PA subfamilies, this indicates that only subfamily5 is a lineage-specific expansion of L1 elements. Subfamily5 is most closely related to L1PA2, but there are several similarities between subfamily5 and L1Pt, indicating that subfamily5 is a novel L1 subfamily specific to the bonobo lineage.

A similar COSEG analysis was performed with a 5' consensus sequence generated from the alignment of all full-length L1PA2 insertions. Using the newly formed 5' L1PA2 consensus sequence, the same 487 L1 insertions were analyzed via COSEG. Similar to the previous 3' analysis, seven subfamilies were generated. Due to sequence similarities, two subfamilies were collapsed into one, with a final total of six subfamilies.

All of the subfamilies from the 3' COSEG analysis had a close match to L1P1. However, 5' L1 sequences are not generally included in libraries for classification purposes. The six L1 subfamilies did not show a linear network and formed a star-like pattern, in contrast to the 3' COSEG L1 subfamilies (**Supplementary Data Figs. S30b and S31b**).

Inspection of full-length SVA insertions derived from SVA_D within the bonobo genome recovered five subfamilies identified by COSEG, four of which belonged to the SVA_PtA subfamily (**Supplementary Data Fig. S32**). After noting the high divergence within the alignments for each subfamily, improvement of the consensus sequences was achieved by re-aligning the full-length SVA_D elements. While the 3' end of the consensi generated by COSEG were an exact match, the VNTR expansion, and therefore length, and divergence of the 5' end indicate the presence of multiple SVA_PtA-related subfamilies. To assess the evolutionary relationship of the five SVA subfamilies, a neighbor-joining tree with 1000 bootstraps was performed (**Supplementary Data Fig. S32**). To ensure that the VNTR region did not influence the phylogenetic tree, it was removed from the nucleotide alignment, and the neighbor-joining tree was redrawn. The result was the same tree as seen in **Supplementary Data Fig. S32**. Comparison of these subfamilies with annotated elements in chimpanzee correlated well with the expected age of the subfamilies, with the SVA_PtA2_Pp1 having no identifiable syntenic copies in chimpanzee (**Supplementary Data Fig. 30c**).

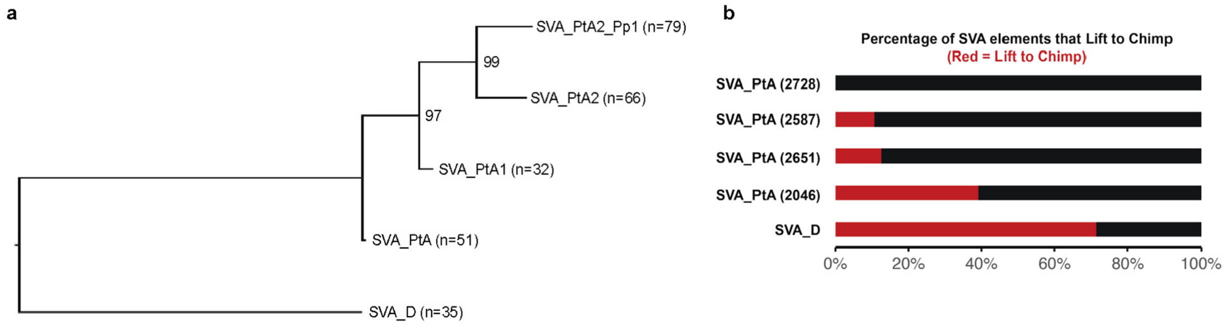

Supplementary Data Figure S32. SVA subfamily comparison in the Pan genus. a, SVA mobile element analysis within the bonobo genome. A neighbor-joining tree rooted with the SVA_D subfamily. The numbers at two of the nodes indicate the bootstrap support from 1000 replicates. The name of the subfamily is based upon a match to the 3' end of established SVA consensus sequences as well as the length in bp of the consensus sequence. Note the absence of a bootstrap value for the branch between SVA_PtA and SVA_PtA1. In this instance, the length of the VNTR placed the SVA_PtA after the SVA_D root. **b,** liftOver of elements to the chimpanzee genome. The majority of instances in the SVA_D subfamily lift (red bar), while SVA_PtA (2728), referred to as SVA_PtA2_pp1 in (a) appears to be bonobo-specific.

We next examined PTERV1, an endogenous retrovirus found in chimpanzee, bonobo, and gorilla but not orangutan or human due to ILS[94,95]. The investigation of full-length PtERV elements within the bonobo genome revealed the presence of two subfamilies as identified by COSEG. However, the divergence of the insertions within those two subfamilies indicated the presence of subfamilies within those identified by COSEG. Based on COSEG and nucleotide divergence, five subfamilies were identified. Following the generation of a neighbor-joining tree, a split was observed between those subfamilies that contained the LTR of PtERV1a and that of PtERV1c. What differed was

the RepeatMasker-identified internal sequence (**Supplementary Fig. 3**). The youngest subfamily identified by a low divergence contains an internal PtERV1a sequence with flanking PtERV1c LTR elements (data not shown).

7.2 Annotation of full-length L1 elements

In order to examine the evolution of active LINE-1 elements in the bonobo genome, we filtered RepeatMasker annotations for full-length (>6,000 nt) L1 (the active lineage of LINE-1 in primates) elements in the new bonobo assembly. In the RepeatMasker annotations we found that most full-length L1s contained internal "Sat-1_TSy" (tarsier-specific satellite element) annotations that prevented RepeatMasker from joining L1 subparts. As this annotation is taxonomically inconsistent with bonobo, we concatenated adjacent L1 annotations within 5 bp of one another to generate full-length L1s. Mapping of these L1s to consensus versions from the UCSC Repeat Browser[96] showed good coverage across the consensus L1 sequences, verifying that these small subparts do in fact together constitute full-length elements that mobilized in the bonobo lineage and that the Sat-1_Tsy annotation is artifactual (**Supplementary Fig. 4**).

7.3 New bonobo assembly reveals previously hidden active young L1s

The active lineage of L1 in primates (L1PA) evolves in waves with younger families deriving from older families (**Supplementary Fig. 5a**). L1PA4 elements were active prior to the great ape ancestor and are ancestral to the L1Pt family that is active in the Pan lineage. The previous panpan1.1 assembly identified very few full-length (>6000 nt) L1PA2 and no L1Pt elements in bonobo. However, the long-read Mhudiblu_PPA_v0 identifies a comparable amount of old (980 L1PA4 in panpan1.1, 950 in Mhudiblu_PPA_v0) elements, but many more young (793 L1PA2 and 413 L1Pt in Mhudiblu_PPA_v0 vs 50 L1PA2 and 0 L1Pt in panpan1.1) full-length elements missed previously (**Supplementary Fig. 5b**).

To determine why these elements were not identified in the original panpan1.1 assembly, we took 1 kbp of sequence flanking every full-length L1PA4 and younger element in Mhudiblu_PPA_v0 and mapped these paired sequences to panpan1.1 (**Supplementary Fig. 5c**). The majority of young L1PA elements contained internal gaps or discordant mappings, indicating that these elements posed a significant challenge for the short-read panpan1.1 assembly. Comparison between the two genome assemblies resulted in the recovery of 43 L1Pt elements completely missing in panpan1.1 assembly, although genotyping of 10 additional bonobo individuals showed that only two of those elements were fixed insertions in the bonobo population suggesting that many of these elements are insertion polymorphisms between the two bonobos used as the source for each genome assembly (**Supplementary Fig. 5d**). Mapping of panpan1.1 gaps to the Mhudiblu_PPA_v0 assembly further demonstrated the bias against proper assembly of evolutionary recent sequence, as younger elements were missing proportionally more bases than older ones (**Supplementary Fig. 5e**). LiftOver of these newly identified elements to other great apes showed the expected syntenic relationships (**Supplementary Fig. 5f**), further demonstrating that these newly

identified young L1s are properly assembled in the new genome and evolutionarily young.

L1 elements engage in “evolutionary arms races” with KRAB-ZNF proteins, which bind sequence-specific motifs within the retroelement and recruit transcriptional repression machinery[97]. Previous studies have shown that two KRAB-ZNF proteins, in particular ZNF93 and ZNF649, have evolved to repress L1PA4 elements and were subsequently escaped through combinations of deletions and point mutations[98]. The ZNF93 escape, for example, was mediated by a single, large 129 bp deletion that occurred in the great ape ancestor (during the time period when L1PA3 was active)[98]. Bonobo L1Pt elements also carry this deletion, and additional coverage drops (when bonobo L1Pt elements are aligned to a consensus L1PA4) consistent with the established active L1HS family in humans. These results provide additional confidence for classifications of these elements as young (**Supplementary Fig. 5g**), and suggest that most mutational patterns are shared between humans, chimpanzees, and bonobos.

7.4 Polymorphism of MEI families in chimpanzee and bonobo

In order to examine polymorphisms of young bonobo MEI families, we first generated lists of putative lineage-specific insertions of L1Pt, SVA, and PtERV1 elements. These lists were generated by taking the elements that did not liftOver between bonobo and chimpanzee assemblies. We also used a list of lineage-specific Alu insertions as generated in section 7.1. We then genotyped the coordinate intervals (in bonobo and chimpanzee as appropriate) of each element in these lists using Paragraph and SVTyper with 10 bonobos and 9 chimpanzees (as described in section 6.1). If either approach identified a deletion in these coordinates (AF > 0), we considered the MEI polymorphic. Elements identified on chrY and scaffolds were discarded from the analysis as all chimpanzees genotyped were female and the Mhudiblu reference genome is also female. The fraction of polymorphic elements is reported in **Extended Data Fig. 3**. Chi-squared tests were performed comparing the number of polymorphic and non-polymorphic instances in bonobo versus chimpanzee, as well as comparing PTERV1 to all other elements within each species, and adjusted for the total number of tests using the Bonferroni correction. A complete set of adjusted p-values for these comparisons is presented in **Supplementary Data Table S36**.

Supplementary Data Table S36. P-values for polymorphic MEI comparisons

Comparison (chi-squared)	Adjusted p-values (Bonferroni)
Chimp PtERV1 vs Bonobo PtERV1	1.00E+00
Chimp L1Pt vs Bonobo L1Pt	1.29E-05
Chimp SVA vs Bonobo SVA	6.51E-04
Chimp Alu vs Bonobo Alu	3.91E-18
Chimp PtERV1 vs Chimp Alu	2.62E-74
Chimp PtERV1 vs Chimp SVA	3.79E-19
Chimp PtERV1 vs Chimp L1Pt	2.17E-18
Bonobo PtERV1 vs Bonobo Alu	6.86E-35
Bonobo PtERV1 vs Bonobo SVA	1.89E-62
Bonobo PtERV1 vs Bonobo L1Pt	1.27E-08

Full-length L1 repeats are more complete in Mhudiblu_PPA_v0 compared to panpan1.1. Sequences flanking the L1 insert can either map concordantly between the two assemblies (~6000 nt apart (black)), concordantly but with an internal gap in panpan1.1 (red), discordantly (pink), or adjacently (brown). Younger families (L1Pt) show greater disparity and more likely to be completely represented in Mhudiblu_PPA_v0.

For both L1Pt and PtERV1, we also generated complete lists of syntenic and non-syntenic insertions (identified by reciprocal liftOver) and in these cases also looked for insertions in syntenic loci at insertions that appeared lineage specific when comparing reference genomes. Briefly, we used Cactus liftOver chains to lift the 500 nt flanking an MEI insertion, confirmed that the sequences were contiguous, and did not overlap an equivalent MEI annotation in the target genome. We then looked for evidence of polymorphic insertions using the mapped MEI sequence as ALT and used Paragraph to genotype all insertions. Graphs representing the syntenic relationships for L1Pt and PtERV are shown in **Supplementary Data Fig. S33**.

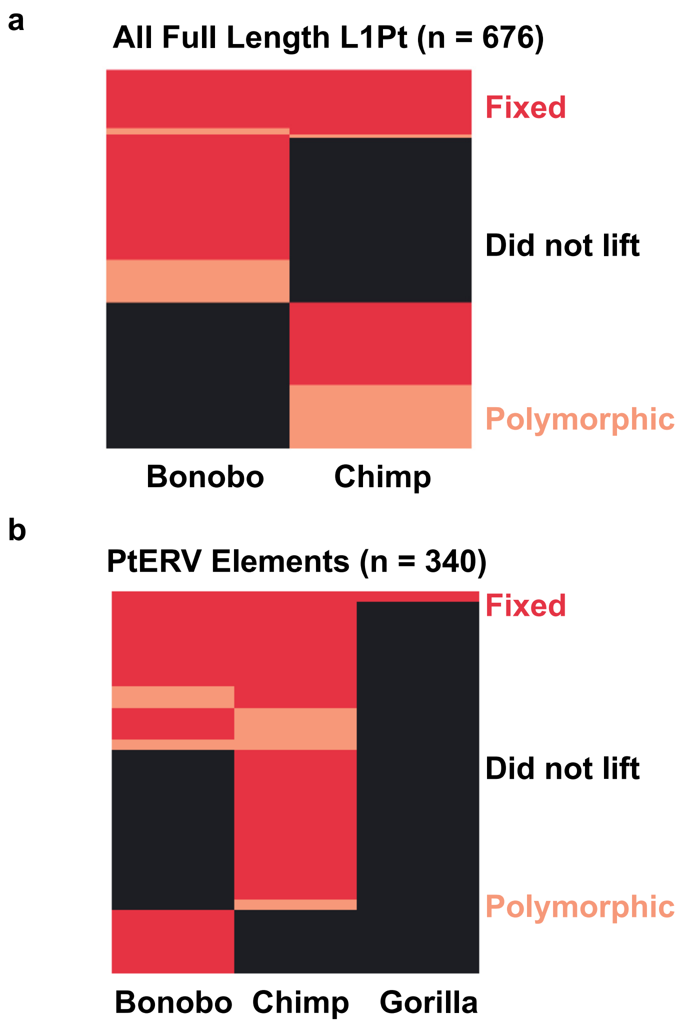

Supplementary Data Figure S33. Representation of the syntenic relationships for L1Pt and PtERV. **a,** All full-length L1Pt elements (n = 676) recovered from bonobo and chimpanzee. Rows with red in both columns are elements fixed in both species. Black rows indicate that no syntenic L1 element match was identified in the corresponding reference genome for that particular L1Pt. Pink rows indicate that the locus is polymorphic in genotyping data from 10 bonobos and 9 chimpanzees. **b,** PTERV1 elements identified in bonobo and chimpanzee were lifted across gorilla, bonobo, and chimpanzee reference genomes and genotyped with data from 10 bonobos and 9 chimpanzees. The PTERV1 founder element is identified at a synthetic locus across all three genomes (top red bar across all three columns indicates the element is present in all species). Chimpanzee- and bonobo-specific elements (red = present, black = absent), as well as polymorphic sites (pink), were also identified.

7.5 Summary of MEI analysis

The new assembly allows for a more in-depth analysis of MEIs because most associated gaps are now resolved (**Extended Data Fig. 3**). This is especially the case for the youngest high-identity MEIs whose discovery allows for the first comparison of rates of insertion and polymorphism between chimpanzee and bonobo (**Extended Data Fig. 3**). Analysis of primate-specific L1s, for example (**Supplementary Fig. 5b**), reveals many full-length copies of the youngest, mobilization-competent bonobo L1s (L1PA2 and L1Pt). Almost all of these (93% of L1PA2, 96% of L1Pt) were fragmented in

panpan1.1 (**Supplementary Fig. 5c-e**). We now find that the number of full-length L1Pt elements in the bonobo genome (413 L1Pt) is similar to chimpanzee (383 L1Pt) and 15-25% more than the number of the youngest L1 family in humans (330 L1HS). These counts are consistent with experimental measurements of retrotransposition rates in primate iPSCs that suggest that human-specific L1s are more potently controlled by restriction factors[99].

An analysis of lineage-specific Alu elements within the bonobo genome identifies 1,548 full-length MEIs, corresponding to five subfamilies (**Extended Data Fig. 3**). Two of these subfamilies are novel, while the other three are a perfect or near-perfect match to the previously identified AluY_p1 or AluY_p2 subfamily (**Extended Data Fig. 3**). The number of lineage-specific elements is nearly identical to that of chimpanzee (n = 1,492) indicting a similarly low rate (**Supplementary Data Table S35**) of Alu retrotransposition among *Pan* lineages when compared to humans (where the rate has doubled) and the rhesus genome (where the Alu insertion rate is ~10-fold) (**Extended Data Fig. 3**). Inspection of full-length SVA insertions derived from SVA_D within the bonobo genome recovered five subfamilies identified by COSEG, four of which belonged to the SVA_PtA subfamily (**Supplementary Data Fig. S32**). Syntenic comparison of these subfamilies with annotated elements in chimpanzee correlated well with the expected age of the subfamilies, with most SVA_D elements shared and the SVA_PtA2_Pp1 having no identifiable syntenic copies in chimpanzee. Unlike other mobile elements that show a lower amount of polymorphism in bonobo when compared to chimpanzee consistent with their SNV genetic diversity[88], we find that SVA elements show a higher degree of polymorphism (**Extended Data Fig. 3**) in bonobo (45%) when compared to chimpanzee (35%) ($p < 6.5 \times 10^{-4}$). Finally, we examined PtERV1, an endogenous retrovirus found in chimpanzee, bonobo, and gorilla but not orangutan or human due to ILS[94,95]. Gorillas and chimpanzees/bonobos share one syntenic insertion of a solo PtERV1 LTR (chr19:49873962-49874340[1]), indicating that a single founder virus invaded the Homininae common ancestor but expanded independently in gorillas and the *Pan* species, before being quickly suppressed by host restriction factors[100,101]. We identified 216 PtERV1 elements in the bonobo genome of which only 120 contained internal (non-LTR) sequence and divided them into two subfamilies. Of the 216, 54 were absent in the reference chimpanzee genome, while 135 of the 277 PtERV1 instances in the chimpanzee were absent in the Mhudiblu_PPA_v0 genome. Only 7% (16/216) of bonobo PtERV1 are polymorphic, significantly less ($p < 1 \times 10^{-5}$) than the rates of most other active mobile elements where polymorphism rates range from 23-45% (**Extended Data Fig. 3**). The fact that chimpanzee shows an indistinguishable low rate of polymorphism for PtERV1 (9%) suggests relatively little activity since *Pan* divergence.

<u>**8. Bonobo genomic diversity analysis and bonobo archaic introgressed regions analysis**</u>

8.1 Genomic diversity among bonobo, chimpanzee and human

Using minimap2, we aligned the chimpanzee (Clint_PTRv2), human (GRCh38), and bonobo (Mhudiblu_PPA_v0) genomes in 1 Mbp windows and computed pairwise nucleotide divergence for autosomes separately from the X chromosome considering SNVs as well as SNV+INDEL differences combined (**Supplementary Data Fig. S34**). The primary statistics including the mean are highly consistent (see below). We investigated outliers (regions of excess divergence as suggested by the bimodal peak) on the X chromosome in smaller 100 kbp bins and find that they correspond primarily to regions of duplications and inversions where optimal pairwise alignments are more difficult to construct (**Supplementary Data Fig. S35**). The overall nucleotide divergence between chimpanzee and bonobo based on the latest genome assemblies is 0.421±0.086 for autosomes and 0.311±0.060% for the X chromosome (**Supplementary Table 6**).

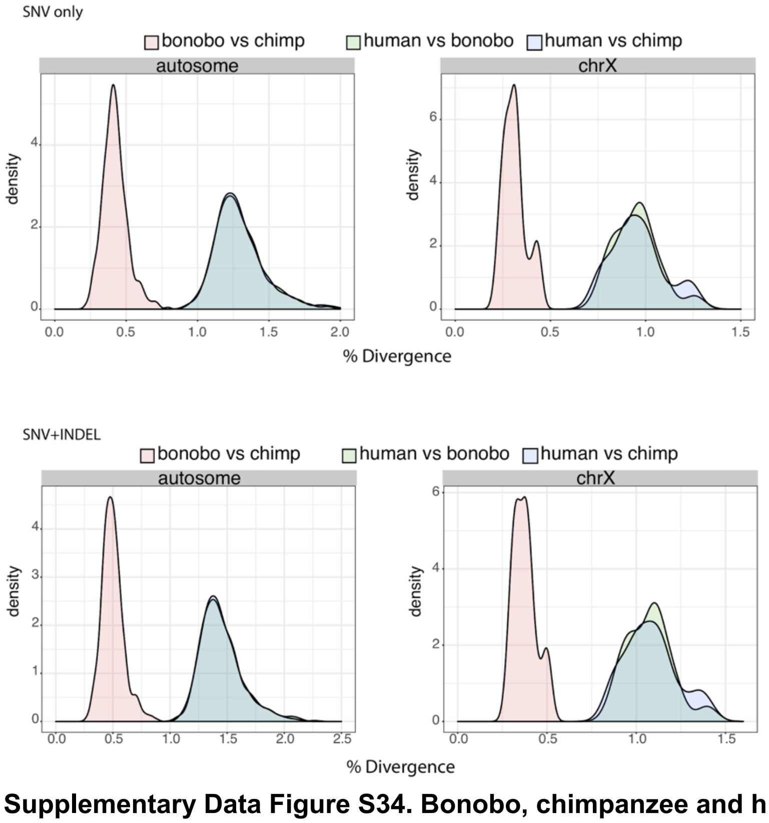

Supplementary Data Figure S34. Bonobo, chimpanzee and human nucleotide divergence. Panels show genome-wide SNV (top) and SNV+INDEL (bottom) divergence based on comparisons between the chimpanzee (Clint_PTRv2), bonobo (Mhudiblu_PPA_v0), and human genomes (GRCh38). The divergence was calculated in 1 Mbp non-overlapping windows across all autosomes and chromosome X (excluding X and Y homologous regions, analyzed region: chrX:93120350-155700620).

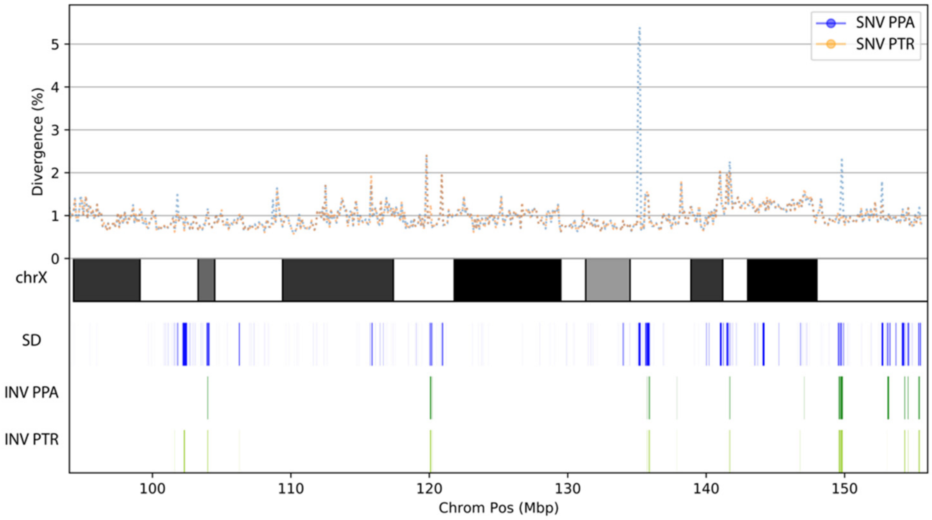

Supplementary Data Figure S35. Divergence outliers on the X chromosome. Chimpanzee (orange, Clint_PTRv2) and bonobo (blue dashed lines, Mhudiblu_PPA_v0) divergence compared to human (GRCh38) X chromosome. The divergence was calculated based on analysis of non-overlapping 100 kbp windows across the X chromosome (excluding X and Y homologous regions). Regions of excess divergence frequently correspond to annotated segmental duplications (SDs, blue) or inverted (INV, green) segments in the chimpanzee genomes.

8.2 Bonobo archaic introgressed regions analysis

We intersected all archaic regions (1,579 segments, 72.67 Mbp) identified by Kuhlwilm and colleagues (see Table S7 in [62]), with fixed SVs and bonobo-specific gene expansions/contractions. We identify 52 fixed deletions (48.2 kbp) and 103 fixed insertions (98.2 kbp) overlapping archaic regions of introgression—none of which disrupted coding sequencing (**Supplementary Data Table S37**). Based on human ENCODE v3 annotation[102], we find five fixed insertions and eight fixed deletions overlapping introgressed regions and potential regulatory DNA (**Supplementary Data Table S37**).

To test for potential enrichment or depletion, we performed a simulation as follows: We binned the bonobo genome into 46 kbp windows (excluding regions where SVs could not be called such as centromeres) and randomly selected 1,579 windows (46 kbp*1579=72.6 Mbp). We computed the number of intersected fixed insertions and deletions as well as the number of the intersected expanded and contracted genes, constructing a distribution of observed events based on 1000 simulations (**Supplementary Data Fig. S36**). We find no evidence of an enrichment of fixed insertions (p-value=0.168) or fixed deletions (p=0.479) among archaic introgressed segments. While we find no bonobo-specific expansions within archaic introgressed regions consistent with expectations (p=0.38), we do identify five specific contractions (AL513128.2, *ACD*, *SMIM32*, *LEFTY2*, and *PTF1A*) representing a significant depletion (p=0).

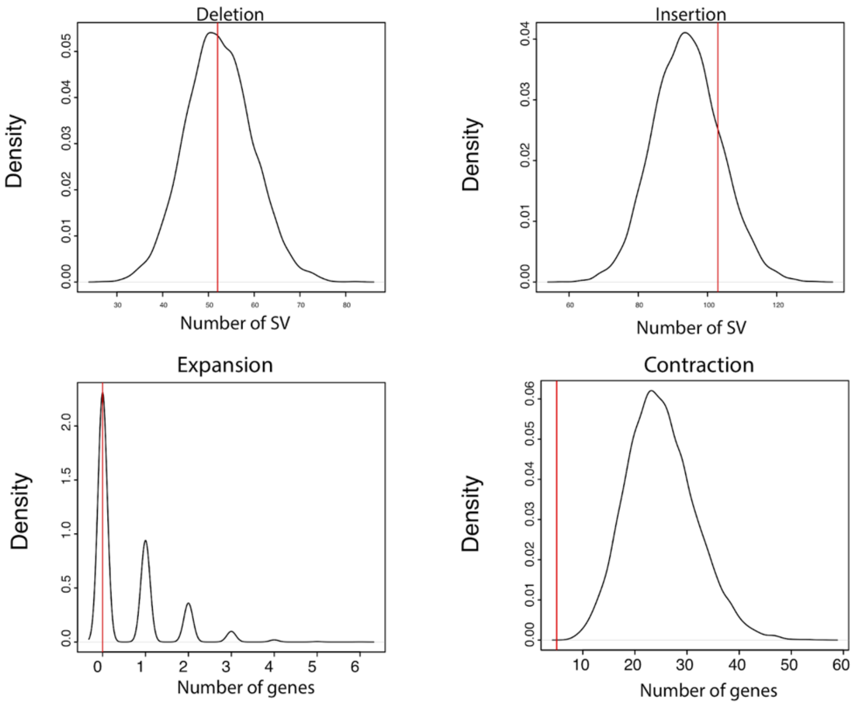

Supplementary Data Figure S36. Introgressed versus SV regions in bonobo. We compared previously identified introgressed regions in bonobo (1,579 segments, 72.67 Mbp) identified by Kuhlwilm and colleagues (see Table S7 in [62]) with regions of structural variation in the bonobo genome. We considered four bonobo categories: **a,** fixed deletions, **b,** fixed insertions, **c,** gene family expansions, and **d,** gene family contractions and identified 155 overlaps (**Supplementary Table 30**). We then performed simulations to assess the significance of overlap. No category showed significance other than gene family contractions, which were significantly depleted in inferred archaic introgressed regions[62].

Supplementary Data Table S37. The intersection of archaic regions and the fixed bonobo SVs and bonobo-specific gene expansions/contractions

Hg38_CHR	START	END	SV ID	SV type	SV len	Introgressed_CHR	START	END	Annotation	genes	ENCODE_CHR	START	END	EH38D	EH38E	CCRE2020
chr12	79619095	79619096	chr12-79619095-INS-3814	INS	3814	chr12	79590000	79630000	intron_variant	*PAWR*	chr12	79619040	79619383	EH38D2581658	EH38E1627386	dELS
chr13	98508970	98508971	chr13-98508970-INS-1671	INS	1671	chr13	98490000	98530000	intron_variant	*STK24*	chr13	98508693	98509039	EH38D2683120	EH38E1691700	dELS
chr14	63563689	63563690	chr14-63563689-INS-329	INS	329	chr14	63540000	63580000	upstream_gene_variant	*AL136038.2*	chr14	63563648	63563997	EH38D2727834	EH38E1720568	dELS
chr21	22711061	22711062	chr21-22711061-INS-68	INS	68	chr21	22680000	22720000	intergenic_variant	*NA*	chr21	22710738	22711067	EH38D3328551	EH38E2133253	dELS
chr7	130894987	130894988	chr7-130894987-INS-60	INS	60	chr7	130860000	130900000	intron_variant&non_coding_transcript_variant	*AC016831.1*	chr7	130894941	130895285	EH38D4031127	EH38E2590655	dELS,CTCF-bound
chr1	235613896	235614669	chr1-235613896-DEL-774	DEL	774	chr1	235590000	235630000	intron_variant	*GNG4*	chr1	235613591	235613913	EH38D2293865	EH38E1434404	pELS
chr1	235613896	235614669	chr1-235613896-DEL-774	DEL	774	chr1	235590000	235630000	intron_variant	*GNG4*	chr1	235614462	235614761	EH38D2293866	EH38E1434405	pELS,CTCF-bound
chr18	5796713	5796888	chr18-5796713-DEL-176	DEL	175	chr18	5790000	5830000	intron_variant&non_coding_transcript_variant	*MIR3976HG*	chr18	5796835	5797176	EH38D2977591	EH38E1897042	dELS
chr19	31119429	31119616	chr19-31119429-DEL-188	DEL	188	chr19	31080000	31120000	intron_variant&non_coding_transcript_variant	*AC020912.1*	chr19	31119578	31119735	EH38D3054513	EH38E1948538	dELS
chr3	58537527	58541961	chr3-58537527-DEL-4435	DEL	4435	chr3	58530000	58600000	upstream_gene_variant	*ACOX2*	chr3	58537452	58537626	EH38D3433780	EH38E2206425	pELS
chr3	58537527	58541961	chr3-58537527-DEL-4435	DEL	4435	chr3	58530000	58600000	upstream_gene_variant	*ACOX2*	chr3	58537777	58538124	EH38D3433781	EH38E2206426	pELS
chr3	58537527	58541961	chr3-58537527-DEL-4435	DEL	4435	chr3	58530000	58600000	upstream_gene_variant	*ACOX2*	chr3	58539103	58539364	EH38D3433782	EH38E2206427	DNase-H3K4me3
chr6	53821045	53822473	chr6-53821045-DEL-1429	DEL	1429	chr6	53790000	53860000	intron_variant	*LRRC1*	chr6	53821085	53821348	EH38D3851951	EH38E2474132	DNase-H3K4me3
chr6	53821045	53822473	chr6-53821045-DEL-1429	DEL	1429	chr6	53790000	53860000	intron_variant	*LRRC1*	chr6	53822462	53822765	EH38D3851953	EH38E2474133	dELS,CTCF-bound
chr8	41587044	41587122	chr8-41587044-DEL-79	DEL	79	chr8	41550000	41590000	intron_variant	*GPAT4*	chr8	41586800	41587136	EH38D4086504	EH38E2627263	dELS
chr9	104868788	104868840	chr9-104868788-DEL-53	DEL	53	chr9	104850000	104890000	intron_variant	*ABCA1*	chr9	104868678	104868989	EH38D4221244	EH38E2713984	dELS
chr9	26131399	26133462	chr9-26131399-DEL-2064	DEL	2064	chr9	26130000	26170000	intergenic_variant	*NA*	chr9	26132733	26133022	EH38D4181843	EH38E2688252	CTCF-only, CTCF-bound

8.3 100 neurobehavioral genes intersection with bonobo-specific SVs and ILS

We investigated the 100 genes associated with neurobiology and social cognition suggested by Staes and colleagues[66] and intersected them with fixed SVs and regions where there was evidence of ILS. We identified 24 fixed deletions and 26 fixed insertions mapping near these genes (15 and 18 genes, respectively), although we note that all 50 SVs mapped to introns and none intersected any predicted coding sequence. Similarly, we identified 79 genes with a nearby signal of ILS, but again all were intronic. Next, we performed a simulation (100 replicates) selecting 100 RefSeq genes at random and computed the number of genes overlapping SVs and regions of ILS. The analysis initially suggested that Staes gene set was highly enriched for both SVs and ILS; however, we also noted that the genes were significantly larger than a random set of genes (typical for genes associated with neurodevelopment). Once we controlled for gene size, we find that neither the number of fixed deletions (p=0.07) nor insertions (p=0.65) are significantly enriched. Interestingly, the number of ILS segments is lower than expected for these 100 genes (p=0.03) perhaps reflecting the action of selection (**Supplementary Data Fig. S37**).

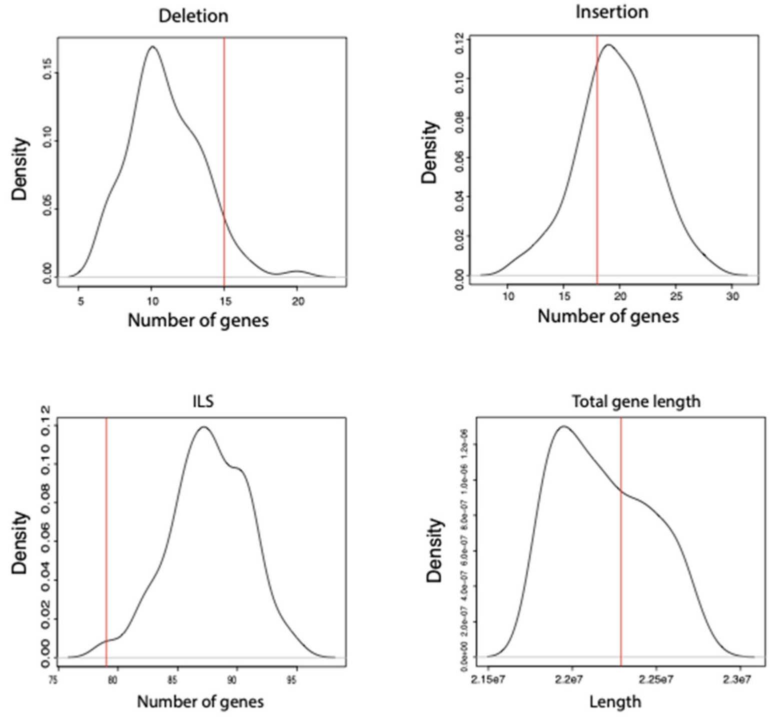

Supplementary Data Figure S37. Neurobehavioral genes, ILS and SV. Staes and colleagues[66] identified 100 candidate genes that might account for neurobehavioral differences between bonobo and chimpanzee. We intersected the 100 candidate genes with our fixed SVs and 500 bp ILS regions and identified 15 genes near 26 fixed deletions, 18 genes near 26 fixed insertions, and 33 genic regions overlapping the 500 bp ILS windows, but none of the events intersected an exon. We performed a simulation intersecting 100 genes matched for gene length from RefSeq. We find that neither the number of fixed deletions (p=0.07) nor insertions (p=0.65) are significantly enriched. Notably, the number of ILS segments is lower than expected for these 100 genes (p=0.03), perhaps reflecting the action of selection.

9. Selection analysis with new sequenced assemblies using bonobo and chimpanzee WGS

9.1 Tajima's D and SweepFinder2 analyses

For the population genetic approaches, we performed a genome-wide analysis for selective sweeps based on Illumina WGS mapped to the bonobo and chimpanzee long-read genome assemblies, namely: Mhudiblu_PPA_v0 and panTro6 (**Supplementary Table 42**). To identify potential sweeps, we applied two different site frequency spectrum (SFS)-based approaches, which search for an excess of rare variants. Briefly, Tajima's D infers the difference between the estimates of $\Theta\pi$, the pairwise differences among individuals, and Θw, based on the number of segregating sites[103]. By contrast, SweepFinder2[104,105] computes a composite likelihood ratio between the likelihood of the presence of a selective sweep at a given position and of the neutral model, modeled by the SFS of the tested sample. The latter method is more suitable for the detection of recent and stronger directional selection events.

Tajima's D was calculated in genomic windows of 10 kbp based on Illumina WGS data from 10 unrelated bonobos and 10 chimpanzees (**Supplementary Table 42**). We limited the analysis to biallelic variants with a QUAL score > 30 and where genomic data were available for at least seven individuals for each species over that region of the genome. All the analyses were performed with VCFtools 0.1.16. The Tajima's D score distribution was similar between chimpanzee and bonobo (**Supplementary Data Fig. S38**). The Manhattan plot of the Tajima's D values are shown in **Supplementary Data Fig. S39**.

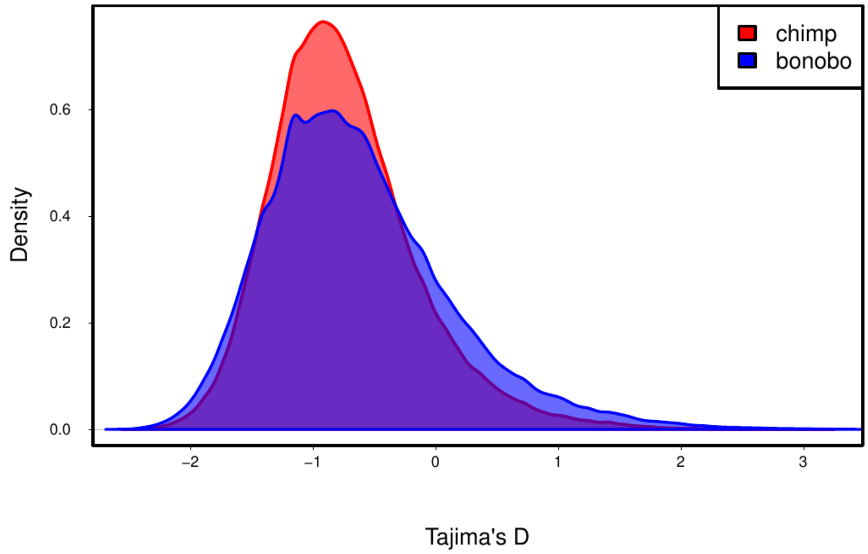

Supplementary Data Figure S38. Density curves for the Tajima's D values inferred in 10 kbp genomic windows. For each species we extracted the top 100 windows, both for positive and negative values.

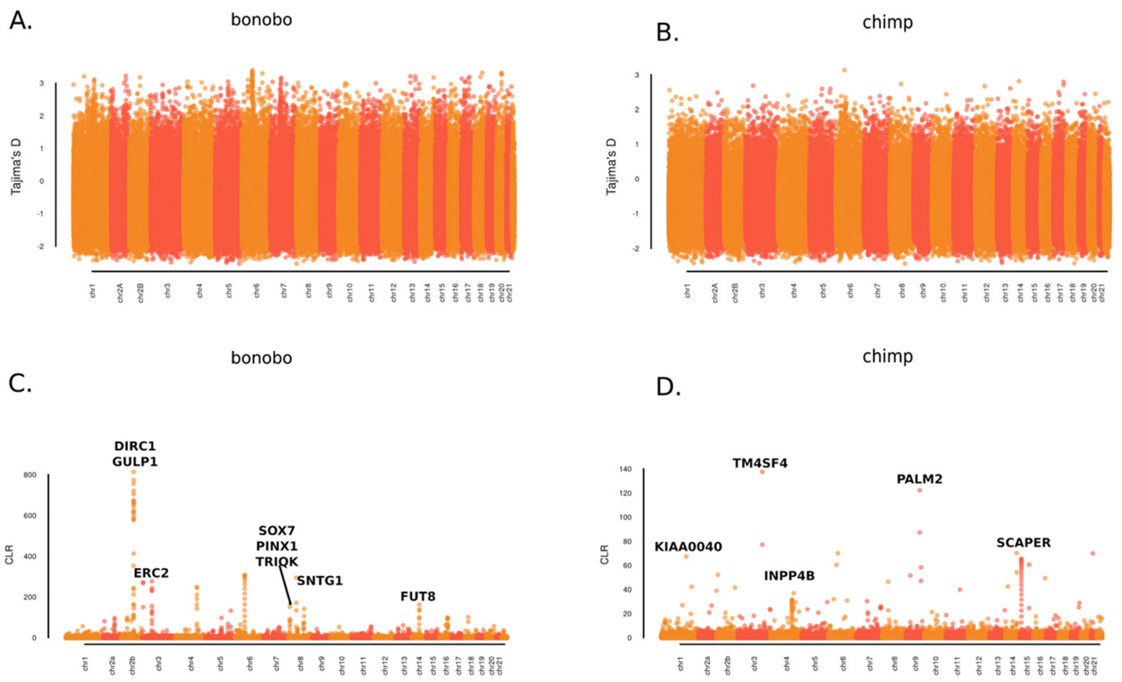

Supplementary Data Figure S39. An overview of Tajima's D (A-B) and SweepFinder2 analysis (C-D) in bonobo and chimpanzee. The Manhattan plot shows Tajima's D (**a & b**) and Composite Likelihood Ratio (**c & d**) for Tajima's D and SweepFinder2 analysis, respectively.

We considered the top 100 genomic windows (negative Tajima's D) and intersected those with underlying genes (**Supplementary Table 15**). In bonobo, we found 64 discrete windows overlapping with 81 genes. We observe potential selective sweeps for *CADM2* (cell adhesion molecule 2, 2 windows D= -2.33 and -2.38, respectively)—a synaptic gene thought to be important in differentiation of synapses and behavioral responses[106] and *EIF4E3* (Eukaryotic Translation Initiation Factor 4E Family Member 3, D=-2.39141)—a gene whose protein product interacts with the 5' mRNA cap at the initial phase of the protein synthesis. The complementary analysis in chimpanzee showed signal for *FOXP2* (D= -2.3)—a transcription factor gene implicated in language development in humans but also shown to be under potential positive selection in chimpanzee[107] **(Supplementary Table 19**).

We also considered potential signatures of balancing selection (top 100 positive Tajima's D values) and intersected these with genes, retrieving 69 genes overlapping with 61 discrete windows (**Supplementary Table 16**). The genes included well-known examples of balancing selection such as MHC genes (*HLA-DPA1* and *HLA-DP2*, two window with D = 2.89 and 3.09) in addition to novel candidates such as *GPC5* (2 windows with D=3.1 and D=3.2, respectively) in bonobo and *KMT2C* (2 windows, D=2.16 and D=2.32), *MSH4* (2 windows, D=2.32 and D=2.15), and *OCA2* (D=2.13) genes in chimpanzee. Interestingly, *GPC5* (glypican 5) is a cell surface heparan sulfate proteoglycan important in cell growth and division while *OCA2* encodes the melanocyte P protein important in hair and skin pigmentation in humans and a subset of other primates (**Supplementary Table 20**).

SweepFinder2 has the advantage over summary-based statistics like Tajima's D in that it controls from the local neutral mutation using the SFS and has the potential to identify more recent evidence of selection[105]. This more advanced method has been shown to result in much higher sensitivity for detection of selective sweeps[108] (compare **Supplementary Data Fig. S39a and c**). We analyzed the genome using 10 kbp discrete windows for both chimpanzee and bonobo in the absence of recombination given the uncertainty of recombination rate differences and report the top 100 candidate regions (**Supplementary Table 17 and 21**).

For bonobo, we observed the strongest signal for chromosome 2b (75820999-76221031), within a region containing *DIRC1* (Disrupted In Renal Carcinoma 1) and *GULP1* (GULP PTB Domain Containing Engulfment Adaptor 1). *DIRC1* is expressed at low level in several tissues, while *GULP1* encodes an adapter protein involved in the phagocytosis of apoptotic cells and is ubiquitously expressed. High SweepFinder2 composite likelihood ratio (SCLR) values were also observed for three windows (chr8: 46946928-47006932) within *SNTG1*, encoding for the neuronal syntrophin protein associated with subcellular localization of proteins and neurotrophic signaling (**Supplementary Data Fig. S23**). On the same chromosome, putative selected regions are also observed in association with *PINX1* (PIN2/TERF1-interacting telomerase inhibitor 1) encoding a telomerase inhibitor and *SOX7* (SRY-related HMG-box 7), a transcription factor associated with embryonic development and in the determination of the cell fate, and *TRIQK* (triple QxxK/R motif-containing protein)—another gene potentially important in embryonic development. For chimpanzee, we observed the strongest signal for *TM4SF4* (Transmembrane 4 L Six Family Member 4) (chr3:147550781-147570782), encoding a transmembrane protein of the tetraspanin family thought to be important for cell proliferation especially in the gut (**Supplementary Data Fig. S23**).

9.2 dN/dS positive selection

We also searched for evidence of an excess of amino acid replacements in protein-coding genes in the bonobo and hominid lineages. We applied a branch-site model of selection to all single-copy orthologs for 12,175 single-copy gene orthologs (identified by Orthofinder[109]) based on available RefSeq annotations of human, chimpanzee, bonobo, and gorilla; 2,322 single-copy orthologs showed some evidence of selection based on the aBSREL (adaptive branch-site random effects likelihood_ model implemented in the HyPhy software package with Bonferroni correction (false discovery rate < 0.05)[110]. We then applied the PAML branch-site model to estimate selection of 2,322 single-copy orthologs, manually excluding alignment and isoform ambiguities. We identified 45 single-copy orthologs as significant using both the aBSREL model (HyPhy) and branch-site model (PAML). We classified genes into two categories: those with multiple amino acid replacements (n≥5) and the others likely resulting from a single mutational event (n<5) (**Supplementary Data Tables S38 and S39**). Inspection of the latter suggested that multiple amino acid replacements changes most from a single frameshift event producing a cluster of amino-acid replacements (e.g., *IFT80*) (**Supplementary Data Fig. S40**).

Supplementary Data Table S38. Summary of genes in the Pan lineage with excess amino acid replacement

	bonobo	chimp	pan	total
Multiple events (n>=5)	20	15	5	40
Single amino acid changes (n<5)	2	2	1	5
All	22	17	6	45

Supplementary Data Table S39. Candidate genes showing excess of amino acid replacement on specific branches

Lineage	Gene	HUMAN_refseq	BONOBO_refseq	CHIMP_refseq	GORILLA_refseq	ORANGUTAN_refseq	Alignment
bonobo	*BAIAP2L1*	NM_018842.5	XM_034963621.1	XM_016945059.2	XM_031006653.1	XM_002817703.4	Single amino acid changes
bonobo	*SLC15A5*	NM_001170798.1	XM_034935426.1	XM_001142606.4	XM_031000605.1	XM_002822990.3	
chimp	*EXD3*	NM_017820.5	XM_034929641.1	XM_024346011.1	XM_031014734.1	XM_024252353.1	
chimp	*STRC*	NM_153700.2	XM_034938649.1	XM_024353823.1	XM_031006451.1	XM_024232864.1	
pan	*VSIG8*	NM_001013661.1	XM_034938323.1	XM_016949587.2	XM_031011334.1	XM_002809931.2	
bonobo	*C17orf99*	NM_001163075.2	XM_034942992.1	XM_511708.6	XM_031010589.1	XM_002827888.1	Successive amino acid changes
bonobo	*C2CD4C*	NM_001136263.2	XM_034950970.1	XM_016934474.2	XM_031006675.1	XM_024237544.1	
bonobo	*CD6*	NM_006725.5	XM_034932717.1	XM_001144310.3	XM_031016447.1	XM_024255879.1	
bonobo	*COA6*	NM_001206641.3	XM_034949257.1	XM_001152917.4	XM_004028612.3	XM_002809287.3	
bonobo	*FLT4*	NM_182925.5	XM_034961238.1	XM_518160.5	XM_031011037.1	XM_024247110.1	
bonobo	*GMNC*	NM_001146686.3	XM_034955648.1	XM_016942503.2	XM_031009347.1	XM_002814416.3	
bonobo	*GPAA1*	NM_003801.4	XM_034953574.1	NM_001280127.1	XM_004047660.3	XM_002819548.2	
bonobo	*GPX7*	NM_015696.5	XM_034952613.1	NM_001145837.1	XM_004025805.3	XM_002810818.3	
bonobo	*GUCY2C*	NM_004963.4	XM_034934987.1	XM_528746.6	XM_031000932.1	XM_002822972.2	
bonobo	*MYLK4*	NM_001347872.2	XM_034961355.1	XM_024357006.1	XM_031011896.1	XM_002816349.3	
bonobo	*NOS2*	NM_000625.4	XM_034942227.1	XM_024350675.1	XM_019028405.2	XM_024235442.1	
bonobo	*NOTCH2*	NM_024408.4	XM_034954795.1	XM_024354924.1	XM_031008833.1	XM_009245522.2	
bonobo	*PGC*	NM_002630.4	XM_034962076.1	XM_016955459.1	XM_004043998.3	NM_001145471.1	
bonobo	*PTPRCAP*	NM_005608.3	XM_008954165.2	XM_009423559.3	XM_004051640.3	XM_002821448.4	
bonobo	*PXMP2*	NM_018663.3	XM_034934932.1	XM_016924698.1	XM_031016629.1	XM_024256533.1	
bonobo	*SIGLEC15*	NM_213602.3	XM_034943572.1	XM_512109.7	XM_004059362.3	XM_003778966.3	
bonobo	*SIVA1*	NM_006427.4	XM_034938269.1	XM_510197.7	XM_004055782.3	XM_002825158.3	
bonobo	*SLC22A24*	NM_001136506.2	XM_034934157.1	XM_016921100.2	XM_019035732.2	XM_024254451.1	
bonobo	*TMPRSS11F*	NM_207407.2	XM_034959551.1	XM_024356448.1	XM_004038740.3	XM_002814738.2	
bonobo	*TRIM58*	NM_015431.4	XM_034950407.1	XM_009441849.3	XM_004028728.3	XM_002809204.4	
chimp	*AWAT2*	NM_001002254.1	XM_003816891.2	XM_016942825.1	XM_004064321.1	XM_024240428.1	
chimp	*CFAP47*	NM_001304548.2	XM_003805971.4	XM_024353026.1	XM_019019001.2	XM_024240715.1	
chimp	*COX10*	NM_001303.4	XM_024926171.2	XM_024350477.1	XM_031003401.1	NM_001133552.1	
chimp	*CTRC*	NM_007272.3	XM_003806260.3	XM_016948900.2	XM_004024717.3	XM_002811451.3	
chimp	*DEPP1*	NM_007021.4	XM_003816749.4	XM_016918164.2	XM_004049323.3	XM_024254056.1	
chimp	*DNAJC14*	NM_032364.6	XM_034935691.1	XM_016923255.2	XM_019038594.2	XM_009247875.2	
chimp	*FAM240A*	NM_001195442.2	XM_008971808.2	XM_024355267.1	XM_019023162.2	XM_009239104.2	
chimp	*LONRF2*	NM_198461.4	XM_014343901.2	XM_003949866.4	XM_004031509.3	XM_002811696.3	
chimp	*MDFIC2*	NM_001364677.1	XM_024928364.2	XM_024355269.1	XM_019023242.2	XM_024245392.1	
chimp	*OC90*	NM_001080399.3	XM_003830096.2	XM_016959023.2	XM_004047537.3	XM_024251084.1	
chimp	*P2RY11*	NM_002566.5	XM_034944455.1	XM_009434582.3	XM_004059978.2	XM_009252768.2	
chimp	*PATE1*	NM_138294.3	XM_003819904.3	XM_024347554.1	XM_004052386.1	XM_002822663.3	
chimp	*RBP2*	NM_004164.3	XM_008951938.2	XM_016942060.2	XM_019023530.1	XM_002814102.2	
chimp	*TCP10L*	NM_144659.7	XM_034963244.1	NM_001044377.1	XM_019017592.2	XM_024239407.1	
chimp	*TYR*	NM_000372.5	XM_003832989.2	XM_001136041.2	XM_031006243.1	XM_002822337.3	
pan	*ACOD1*	NM_001258406.2	XM_034936349.1	XM_016925652.2	XM_004054615.3	XM_002824350.4	
pan	*IFT80*	NM_020800.3	XM_003830626.3	NM_001279914.1	XM_019023794.2	XM_024244226.1	
pan	*KIF25*	NM_030615.3	XM_034963269.1	XM_024357516.1	XM_031012390.1	XM_009242446.2	
pan	*MED31*	NM_016060.3	XM_003810140.5	XM_523838.6	XM_004058424.3	XM_002826922.4	
pan	*SMIM20*	NM_001145432.2	XM_034959538.1	XM_024356026.1	XM_031010560.1	XM_009239854.1	

a

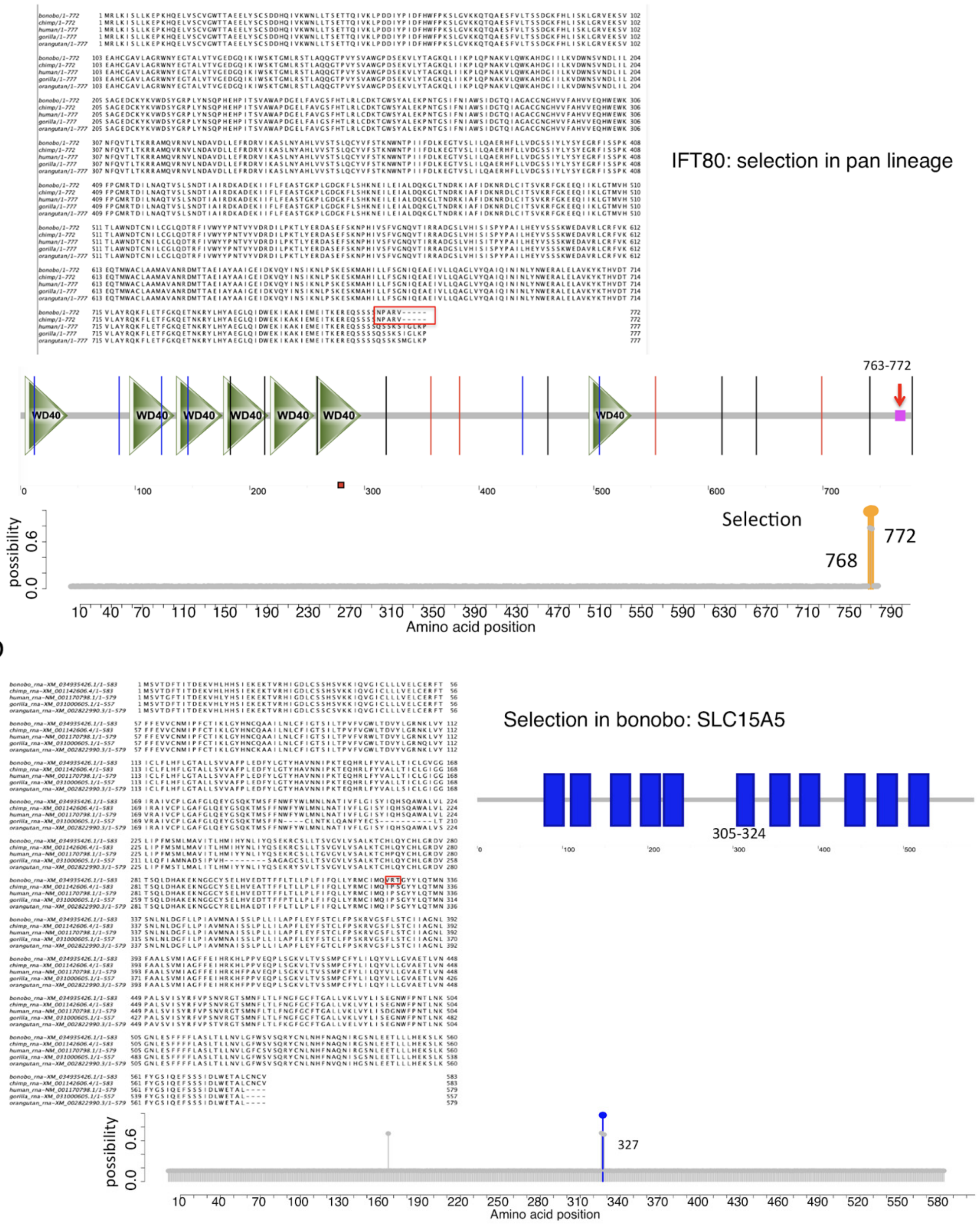

Supplementary Data Figure S40. Candidate positive selection genes with excess amino acid replacement. a, Multiple protein sequence alignment (top panel) shows signals of positive selection (PAML, bottom panel) in *IFT80* in the Pan lineage (chimpanzee and bonobo) resulting in a cluster of amino acid replacements in the carboxy terminus (middle panel). *IFT80* is involved in the function of motile and sensory cilia and bone development. **b,** An example of a gene under positive selection (PAML, bottom panel) encoding the SLC15A5 protein with three amino acid replacement changes (top left) mapping to a transmembrane domain (top right). The gene is highly expressed in fat tissue and is

associated with dicarboxylic aminoaciduria and hydranencephaly. 95% selection possibility from PAML model is shown in orange, 99% selection possibility from PAML model is shown in blue.

9.3 Comparison of candidate genes among positive selection tests

We compared the various tests for positively selected genes to determine if any genes were observed by more than one test (**Supplementary Table 18 and Supplementary Data Fig. S41**).

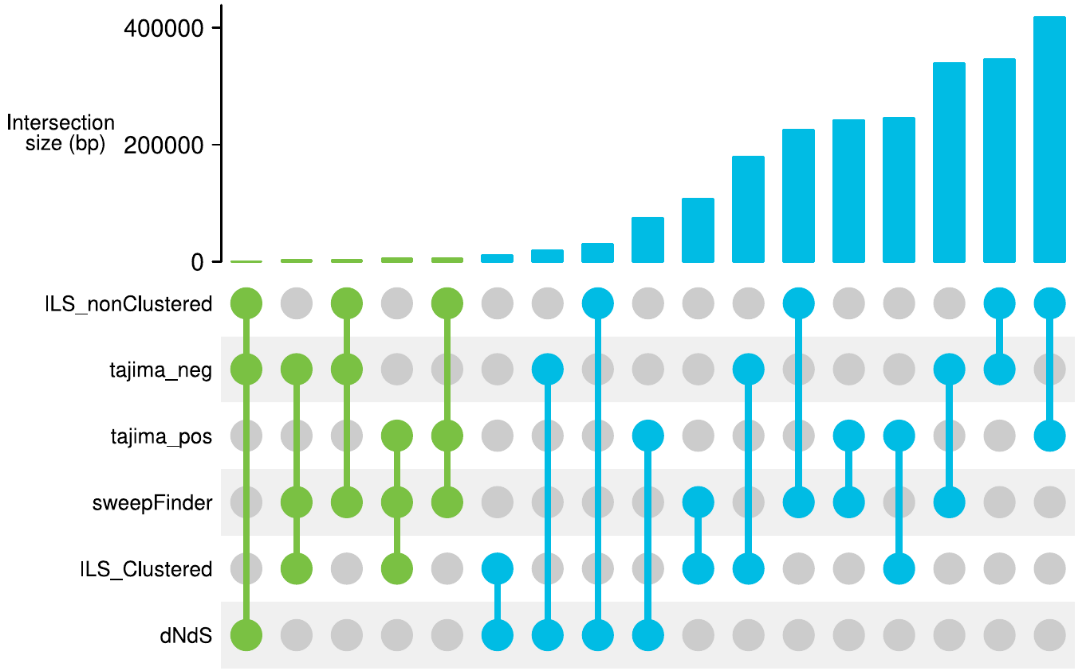

Supplementary Data Figure S41. Upset plot of multiple intersections among selection tests and ILS coordinates. The barplot shows the amount of overlapping base pairs resulting from the intersection of the tests/ILS scan indicated by the connecting points.

We were specifically interested in genes that showed evidence of positive selection by both negative Tajima's D values and SweepFinder2, focusing on the top 1% of signals (**Supplementary Table 18 and 22**). Among the intersecting 50 windows for bonobo, we identified two genes related to lipid metabolism: 2-arachidonoyl-glycerol, an endocannabinoid (interacting with cannabinoid receptors) (*DAGLA* = chr11: 56979557 - 57046589, Tajima's D value=-1.99, SCLR= 13.5) and *ABHD2* = chr15: 67780452-67891154. Tajima's D value=-2.29, SCLR= 8.54). Of note, we also identified signatures of positive selection for *CAMK2D* (chr4: 106083972- 106103972, Tajima's D = -2.11, SCLR = 6.99), an upstream regulator of *DAGLA* activity suggesting that the pathway may be under selection in bonobo.

We also identified a putative selected window within *CEP164* (chr11: 112185192-112205192, Tajima's D= -2.02, SCLR= 15.7), involved in microtubule organization. Within the chimpanzee lineage, we found both signals of selection corresponding to the *GRIA4* (chr11:101388489-101694639, Tajima's D= -1.92, SCLR=3.84), which encodes for the glutamate receptor and found evidence of selection in genes related to chromatin

structure: *PHF2* (chr9:65812964-65914169, Tajima's D=-2.07, SCLR= 9.64) and *HIST1H1C* (chr6:19089567-19090347, Tajima's D= -2.36, SCLR = 5.24).

Based on this intersection set of genes (n=21), we searched for gene ontology and gene expression enrichment. For gene ontology enrichment analysis, we applied enrichr[111], testing our gene set against five different annotations libraries (KEGG_2019_Human, GO_Molecular_Function_2018, GO_Biological_Process_2018, GO_Cellular_Component_2018, and Panther_2016[112]) as described for expansions and contractions (**section 6.4.1**). Acylglycerol lipase activity (GO Molecular Function 2018), Lipase activity (GO Molecular Function 2018) and 2-arachidonoylglycerol biosynthesis[112] were significantly enriched GO categories (**Supplementary Data Table S40**). By contrast, no GO category was enriched for positively selected genes (n=32) in chimpanzee.

Supplementary Data Table S40. GO enrichment analysis of putative selected genes in bonobo

	Overlap	P-value	Adjusted P-value	Odds Ratio	Combined Score	Genes	Gene_set
acylglycerol lipase activity (GO:0047372)	2/11	5.7E-05	2.1E-03	2.3E+02	2280.8	*DAGLA; ABHD2*	GO_Molecular_Function_2018
lipase activity (GO:0016298)	2/43	9.2E-04	1.7E-02	5.1E+01	357.7	*DAGLA; ABHD2*	GO_Molecular_Function_2018
2-arachidonoylglycerol biosynthesis Homo sapiens P05726	1/6	6.3E-03	1.9E-02	2.0E+02	1012.6	*DAGLA*	Panther_2016

Gene classes enriched; p-value: p-value based on Fisher's test; Overlap: number of genes in the tested set overlapping with the gene category; Adjusted p-value: Benjamini-Hochberg adjusted p-value; Genes: Name of the genes in the overlap; Gene set: Gene ontology class.

9.4 MHC locus selection. Mhudiblu_PPA_v0 and panpan1.1 comparison

We performed a detailed analysis of the MHC locus with a specific focus on evidence of selection between our study and the previous study. We began by first comparing the degree of completion in this region and found 291 gaps in the previous assembly (red bars, **Supplementary Data Fig. S42**) versus two gaps in the Mhudiblu assembly (purple bars).

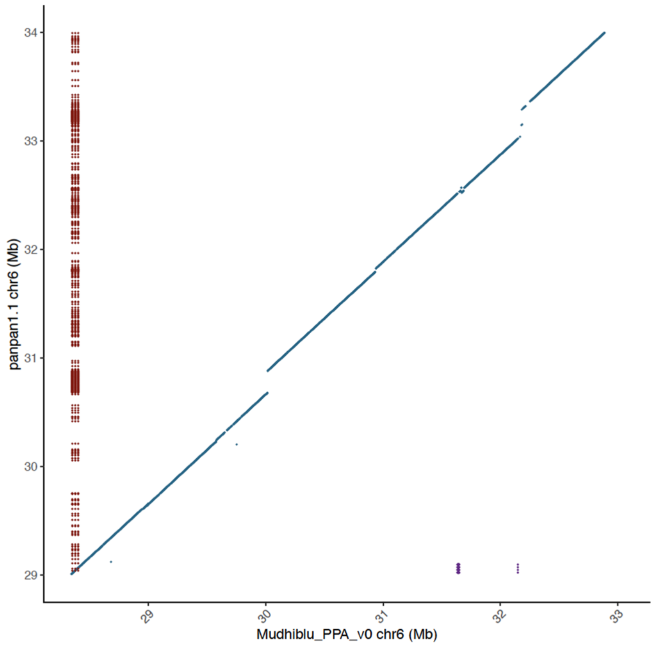

Supplementary Data Figure S42. Dot matrix comparison of MHC region. The MHC region of the Mhudiblu_PPA_v0 bonobo assembly compared with the panpan1.1 bonobo assembly from Prufer et al. (2012)[28]. The current bonobo assembly contig gaps are shown along the x-axis in purple. The Prufer et al. (2012) assembly is represented along the y-axis, with the contig gaps shown in red. In the MHC region, there are two gaps in the Mhudiblu_PPA_v0 assembly and 291 gaps in the Prufer et al. assembly[28]. Alignment between the two genomes is represented in blue with each dot representing 1 kbp of alignment.

As expected, we observed strong signals of balancing selection (Tajima's D values for the two significant 10 kbp windows chr6:32650000-32660000 and chr6:32660000-32670000 are 2.89 and 3.10, respectively) and clustered ILS of various topologies across multiple regions within the MHC locus (**Extended Data Fig. 8**). These findings are generally consistent with previous reports from Prufer and colleagues[28]. The strongest signals were observed for bonobo orthologs of the MHC genes (*HLA-DPA1* and *HLA-DP2*).

The previous study, however, showed regions of reduced diversity in bonobo based on a comparison to chimpanzee. We do not find compelling evidence that these sites are under positive selection based on SweepFinder2 or Tajima's D analyses. We further followed this up by directly comparing the genetic diversity (pi) bonobo versus chimpanzee. With one exception, we observed no regions of significantly reduced diversity. The one exception where both chimpanzee and bonobo show a reduction of single-nucleotide polymorphisms (SNPs) corresponds to an SD (chr6: 26666991-27002570) where SNPs were removed in our VCF due to paralogy. Overall, SNP diversity is reduced across the region in bonobo when compared to chimpanzee and there are five regions (red arrows) (**Extended Data Fig. 8)** where diversity is the greatest between chimpanzee and bonobo. Three of these correspond to regions identified by the previous study; however, they are not among the top 1% of genome windows showing positive selection.

References

1 Kronenberg, Z. N. *et al.* High-resolution comparative analysis of great ape genomes. *Science* **360**, doi:10.1126/science.aar6343 (2018).

2 Marchetto, M. C. *et al.* Species-specific maturation profiles of human, chimpanzee and bonobo neural cells. *Elife* **8**, doi:10.7554/eLife.37527 (2019).

3 Dougherty, M. L. *et al.* Transcriptional fates of human-specific segmental duplications in brain. *Genome Res* **28**, 1566-1576, doi:10.1101/gr.237610.118 (2018).

4 Chin, C. S. *et al.* Nonhybrid, finished microbial genome assemblies from long-read SMRT sequencing data. *Nat Methods* **10**, 563-569, doi:10.1038/nmeth.2474 (2013).

5 Walker, B. J. *et al.* Pilon: an integrated tool for comprehensive microbial variant detection and genome assembly improvement. *PLoS One* **9**, e112963, doi:10.1371/journal.pone.0112963 (2014).

6 Haplotype-based variant detection from short-read sequencing (arXiv, 2012).

7 Hsieh, P. *et al.* Adaptive archaic introgression of copy number variants and the discovery of previously unknown human genes. *Science* **366**, eaax2083, doi:10.1126/science.aax2083 (2019).

8 Huddleston, J. *et al.* Reconstructing complex regions of genomes using long-read sequencing technology. *Genome Res* **24**, 688-696, doi:10.1101/gr.168450.113 (2014).

9 Ventura, M. *et al.* Gorilla genome structural variation reveals evolutionary parallelisms with chimpanzee. *Genome Res* **21**, 1640-1649, doi:10.1101/gr.124461.111 (2011).

10 Stanyon, R. *et al.* Primate chromosome evolution: ancestral karyotypes, marker order and neocentromeres. *Chromosome Res* **16**, 17-39, doi:10.1007/s10577-007-1209-z (2008).

11 Lichter, P., Jauch, A., Cremer, T. & Ward, D. C. Detection of Down syndrome by in situ hybridization with chromosome 21 specific DNA probes. *Prog Clin Biol Res* **360**, 69-78 (1990).

12 Porubsky, D. *et al.* Recurrent inversion toggling and great ape genome evolution. *Nat Genet* **52**, 849-858, doi:10.1038/s41588-020-0646-x (2020).

13 Tuzun, E. *et al.* Fine-scale structural variation of the human genome. *Nat Genet* **37**, 727-732, doi:10.1038/ng1562 (2005).

14 Yunis, J. J. & Prakash, O. The origin of man: a chromosomal pictorial legacy. *Science* **215**, 1525-1530 (1982).

15 Goidts, V. *et al.* Complex patterns of copy number variation at sites of segmental duplications: an important category of structural variation in the human genome. *Hum Genet* **120**, 270-284, doi:10.1007/s00439-006-0217-y (2006).

16 Szamalek, J. M., Goidts, V., Cooper, D. N., Hameister, H. & Kehrer-Sawatzki, H. Characterization of the human lineage-specific pericentric inversion that distinguishes human chromosome 1 from the homologous chromosomes of the great apes. *Hum Genet* **120**, 126-138 (2006).

17 Catacchio, C. R. *et al.* Inversion variants in human and primate genomes. *Genome Res* **28**, 910-920, doi:10.1101/gr.234831.118 (2018).

18 Dutrillaux, B., Rethoré, M. O. & Lejeune, J. [Analysis of the karyotype of Pan paniscus. Comparison with other Pongidae and man (author's transl)]. *Humangenetik* **28**, 113-119, doi:10.1007/BF00735743 (1975).
19 Stanyon, R., Chiarelli, B., Gottlieb, K. & Putton, W. A phylogenetic and taxonomic status of *Pan paniscus*: a chromosomal perspective. *American Journal of Physical Anthropology* **69**, 489-498 (1986).
20 Falconer, E. *et al.* DNA template strand sequencing of single-cells maps genomic rearrangements at high resolution. *Nat Methods* **9**, 1107-1112, doi:10.1038/nmeth.2206 (2012).
21 Sanders, A. D. *et al.* Characterizing polymorphic inversions in human genomes by single-cell sequencing. *Genome Res* **26**, 1575-1587, doi:10.1101/gr.201160.115 (2016).
22 Ghareghani, M. *et al.* Strand-seq enables reliable separation of long reads by chromosome via expectation maximization. *Bioinformatics* **34**, i115-i123, doi:10.1093/bioinformatics/bty290 (2018).
23 Porubský, D. *et al.* Direct chromosome-length haplotyping by single-cell sequencing. *Genome Res* **26**, 1565-1574, doi:10.1101/gr.209841.116 (2016).
24 Porubsky, D. *et al.* breakpointR: an R/Bioconductor package to localize strand state changes in Strand-seq data. *Bioinformatics* **36**, 1260-1261, doi:10.1093/bioinformatics/btz681 (2020).
25 Chaisson, M. J. P. *et al.* Multi-platform discovery of haplotype-resolved structural variation in human genomes. *Nat Commun* **10**, 1784, doi:10.1038/s41467-018-08148-z (2019).
26 Hills, M., O'Neill, K., Falconer, E., Brinkman, R. & Lansdorp, P. M. BAIT: Organizing genomes and mapping rearrangements in single cells. *Genome Medicine* **5**, 82, doi:10.1186/gm486 (2013).
27 Rhie, A., Walenz, B. P., Koren, S. & Phillippy, A. M. Merqury: reference-free quality and phasing assessment for genome assemblies. *bioRxiv*, 2020.2003.2015.992941, doi:10.1101/2020.03.15.992941 (2020).
28 Prüfer, K. *et al.* The bonobo genome compared with the chimpanzee and human genomes. *Nature* **486**, 527-531, doi:10.1038/nature11128 (2012).
29 Kent, W. J. BLAT--the BLAST-like alignment tool. *Genome Res* **12**, 656-664, doi:10.1101/gr.229202 (2002).
30 Prufer, K. *et al.* The bonobo genome compared with the chimpanzee and human genomes. *Nature* **486**, 527-531, doi:10.1038/nature11128 (2012).
31 Sanders, A. D. *et al.* Characterizing polymorphic inversions in human genomes by single-cell sequencing. *Genome Res* **26**, 1575-1587, doi:10.1101/gr.201160.115 (2016).
32 Porubsky, D. *et al.* Direct chromosome-length haplotyping by single-cell sequencing. *Genome Res* **26**, 1565-1574, doi:10.1101/gr.209841.116 (2016).
33 Antonacci, F. *et al.* Characterization of six human disease-associated inversion polymorphisms. *Hum Mol Genet* **18**, 2555-2566 (2009).
34 Garg, S. *et al.* Chromosome-scale, haplotype-resolved assembly of human genomes. *Nat Biotechnol*, doi:10.1038/s41587-020-0711-0 (2020).
35 Porubsky, D. *et al.* Fully phased human genome assembly without parental data using single-cell strand sequencing and long reads. *Nat Biotechnol*, doi:10.1038/s41587-020-0719-5 (2020).

36 Gordon, D. *et al.* Long-read sequence assembly of the gorilla genome. *Science* **352**, aae0344, doi:10.1126/science.aae0344 (2016).
37 He, Y. *et al.* Long-read assembly of the Chinese rhesus macaque genome and identification of ape-specific structural variants. *Nat Commun* **10**, 4233, doi:10.1038/s41467-019-12174-w (2019).
38 Cordaux, R. & Batzer, M. A. The impact of retrotransposons on human genome evolution. *Nat Rev Genet* **10**, 691-703, doi:10.1038/nrg2640 (2009).
39 Erwin, J. A., Marchetto, M. C. & Gage, F. H. Mobile DNA elements in the generation of diversity and complexity in the brain. *Nat Rev Neurosci* **15**, 497-506, doi:10.1038/nrn3730 (2014).
40 Gianfrancesco, O., Bubb, V. J. & Quinn, J. P. SVA retrotransposons as potential modulators of neuropeptide gene expression. *Neuropeptides* **64**, 3-7, doi:10.1016/j.npep.2016.09.006 (2017).
41 Zody, M. C. *et al.* Evolutionary toggling of the MAPT 17q21.31 inversion region. *Nature genetics* **40**, 1076-1083 (2008).
42 Dennis, M. Y. & Eichler, E. E. Human adaptation and evolution by segmental duplication. *Curr Opin Genet Dev* **41**, 44-52, doi:10.1016/j.gde.2016.08.001 (2016).
43 Florio, M. *et al.* Human-specific gene ARHGAP11B promotes basal progenitor amplification and neocortex expansion. *Science* **347**, 1465-1470, doi:10.1126/science.aaa1975
science.aaa1975 [pii] (2015).
44 Ju, X. C. *et al.* The hominoid-specific gene TBC1D3 promotes generation of basal neural progenitors and induces cortical folding in mice. *Elife* **5**, doi:10.7554/eLife.18197 (2016).
45 Bailey, J. A., Yavor, A. M., Massa, H. F., Trask, B. J. & Eichler, E. E. Segmental duplications: organization and impact within the current human genome project assembly. *Genome Res* **11**, 1005-1017, doi:10.1101/gr.gr-1871r (2001).
46 Vollger, M. R. *et al.* Long-read sequence and assembly of segmental duplications. *Nat Methods* **16**, 88-94, doi:10.1038/s41592-018-0236-3 (2019).
47 Bailey, J. A. *et al.* Recent segmental duplications in the human genome. *Science* **297**, 1003-1007, doi:10.1126/science.1072047 (2002).
48 Kelley, D. R. & Salzberg, S. L. Detection and correction of false segmental duplications caused by genome mis-assembly. *Genome Biol* **11**, R28, doi:10.1186/gb-2010-11-3-r28 (2010).
49 Fiddes, I. T. *et al.* Comparative Annotation Toolkit (CAT)-simultaneous clade and personal genome annotation. *Genome Res* **28**, 1029-1038, doi:10.1101/gr.233460.117 (2018).
50 Armstrong, J. *et al.* Progressive alignment with Cactus: a multiple-genome aligner for the thousand-genome era. *bioRxiv*, 730531, doi:10.1101/730531 (2019).
51 Paten, B. *et al.* Cactus: Algorithms for genome multiple sequence alignment. *Genome Res* **21**, 1512-1528, doi:10.1101/gr.123356.111 (2011).
52 Frankish, A. *et al.* GENCODE reference annotation for the human and mouse genomes. *Nucleic Acids Res* **47**, D766-D773, doi:10.1093/nar/gky955 (2019).
53 Nachtweide, S. & Stanke, M. Multi-Genome Annotation with AUGUSTUS. *Methods Mol Biol* **1962**, 139-160, doi:10.1007/978-1-4939-9173-0_8 (2019).
54 Altschul, S. F., Gish, W., Miller, W., Myers, E. W. & Lipman, D. J. Basic local alignment search tool. *J Mol Biol* **215**, 403-410, doi:10.1016/S0022-2836(05)80360-2 (1990).

55 Kapustin, Y., Souvorov, A., Tatusova, T. & Lipman, D. Splign: algorithms for computing spliced alignments with identification of paralogs. *Biol Direct* **3**, 20, doi:10.1186/1745-6150-3-20 (2008).
56 Lowe, T. M. & Eddy, S. R. tRNAscan-SE: a program for improved detection of transfer RNA genes in genomic sequence. *Nucleic Acids Res* **25**, 955-964, doi:10.1093/nar/25.5.955 (1997).
57 Nawrocki, E. P. *et al.* Rfam 12.0: updates to the RNA families database. *Nucleic Acids Res* **43**, D130-137, doi:10.1093/nar/gku1063 (2015).
58 Stanke, M., Diekhans, M., Baertsch, R. & Haussler, D. Using native and syntenically mapped cDNA alignments to improve de novo gene finding. *Bioinformatics* **24**, 637-644, doi:10.1093/bioinformatics/btn013 (2008).
59 Dumas, L. J. *et al.* DUF1220-domain copy number implicated in human brain-size pathology and evolution. *Am J Hum Genet* **91**, 444-454, doi:10.1016/j.ajhg.2012.07.016 (2012).
60 Koren, S. *et al.* Canu: scalable and accurate long-read assembly via adaptive. *Genome Res* **27**, 722-736, doi:10.1101/gr.215087.116 (2017).
61 Ruan, J. & Li, H. Fast and accurate long-read assembly with wtdbg2. *Nat Methods* **17**, 155-158, doi:10.1038/s41592-019-0669-3 (2020).
62 Kuhlwilm, M., Han, S., Sousa, V. C., Excoffier, L. & Marques-Bonet, T. Ancient admixture from an extinct ape lineage into bonobos. *Nat Ecol Evol* **3**, 957-965, doi:10.1038/s41559-019-0881-7 (2019).
63 Rosati, A. G. Heterochrony in chimpanzee and bonobo spatial memory development. *Am J Phys Anthropol* **169**, 302-321, doi:10.1002/ajpa.23833 (2019).
64 Liu, W. *et al.* Wild bonobos host geographically restricted malaria parasites including a putative new Laverania species. *Nat Commun* **8**, 1635, doi:10.1038/s41467-017-01798-5 (2017).
65 Diogo, R., Molnar, J. L. & Wood, B. Bonobo anatomy reveals stasis and mosaicism in chimpanzee evolution, and supports bonobos as the most appropriate extant model for the common ancestor of chimpanzees and humans. *Sci Rep* **7**, 608, doi:10.1038/s41598-017-00548-3 (2017).
66 Staes, N. *et al.* Evolutionary divergence of neuroanatomical organization and related genes in chimpanzees and bonobos. *Cortex* **118**, 154-164, doi:10.1016/j.cortex.2018.09.016 (2019).
67 Haeussler, M. *et al.* The UCSC Genome Browser database: 2019 update. *Nucleic Acids Res* **47**, D853-D858, doi:10.1093/nar/gky1095 (2019).
68 Löytynoja, A. & Goldman, N. webPRANK: a phylogeny-aware multiple sequence aligner with interactive alignment browser. *BMC Bioinformatics* **11**, 579, doi:10.1186/1471-2105-11-579 (2010).
69 Mao, Y., Hou, S., Shi, J. & Economo, E. P. TREEasy: An automated workflow to infer gene trees, species trees, and phylogenetic networks from multilocus data. *Mol Ecol Resour* **20**, doi:10.1111/1755-0998.13149 (2020).
70 Scally, A. *et al.* Insights into hominid evolution from the gorilla genome sequence. *Nature* **483**, 169-175, doi:10.1038/nature10842 (2012).
71 Prado-Martinez, J. *et al.* Great ape genetic diversity and population history. *Nature* **499**, 471-475, doi:10.1038/nature12228 (2013).
72 Yang, Z. PAML 4: phylogenetic analysis by maximum likelihood. *Mol Biol Evol* **24**, 1586-1591, doi:10.1093/molbev/msm088 (2007).

73 Huang, d. W. *et al.* Extracting biological meaning from large gene lists with DAVID. *Curr Protoc Bioinformatics* **Chapter 13**, Unit 13.11, doi:10.1002/0471250953.bi1311s27 (2009).

74 Langergraber, K. E. *et al.* Generation times in wild chimpanzees and gorillas suggest earlier divergence times in great ape and human evolution. *Proc Natl Acad Sci U S A* **109**, 15716-15721, doi:10.1073/pnas.1211740109 (2012).

75 Huang, d. W., Sherman, B. T. & Lempicki, R. A. Systematic and integrative analysis of large gene lists using DAVID bioinformatics resources. *Nat Protoc* **4**, 44-57, doi:10.1038/nprot.2008.211 (2009).

76 Leffler, E. M. *et al.* Revisiting an old riddle: what determines genetic diversity levels within species? *PLoS Biol* **10**, e1001388, doi:10.1371/journal.pbio.1001388 (2012).

77 Ségurel, L. *et al.* The ABO blood group is a trans-species polymorphism in primates. *Proc Natl Acad Sci U S A* **109**, 18493-18498, doi:10.1073/pnas.1210603109 (2012).

78 Cheng, X. & DeGiorgio, M. Detection of Shared Balancing Selection in the Absence of Trans-Species Polymorphism. *Mol Biol Evol* **36**, 177-199, doi:10.1093/molbev/msy202 (2019).

79 Teixeira, J. C. *et al.* Long-Term Balancing Selection in LAD1 Maintains a Missense Trans-Species Polymorphism in Humans, Chimpanzees, and Bonobos. *Mol Biol Evol* **32**, 1186-1196, doi:10.1093/molbev/msv007 (2015).

80 Leffler, E. M. *et al.* Multiple instances of ancient balancing selection shared between humans and chimpanzees. *Science* **339**, 1578-1582, doi:10.1126/science.1234070 (2013).

81 Sedlazeck, F. J. *et al.* Accurate detection of complex structural variations using single-molecule sequencing. *Nat Methods* **15**, 461-468, doi:10.1038/s41592-018-0001-7 (2018).

82 Chen, S. *et al.* Paragraph: a graph-based structural variant genotyper for short-read sequence data. *Genome Biol* **20**, 291, doi:10.1186/s13059-019-1909-7 (2019).

83 Chiang, C. *et al.* SpeedSeq: ultra-fast personal genome analysis and interpretation. *Nat Methods* **12**, 966-968, doi:10.1038/nmeth.3505 (2015).

84 Sudmant, P. H. *et al.* Evolution and diversity of copy number variation in the great ape lineage. *Genome Res* **23**, 1373-1382, doi:10.1101/gr.158543.113 (2013).

85 Mallick, S. *et al.* The Simons Genome Diversity Project: 300 genomes from 142 diverse populations. *Nature* **538**, 201-206, doi:10.1038/nature18964 (2016).

86 Jain, C., Koren, S., Dilthey, A., Phillippy, A. M. & Aluru, S. A fast adaptive algorithm for computing whole-genome homology maps. *Bioinformatics* **34**, i748-i756, doi:10.1093/bioinformatics/bty597 (2018).

87 Cheng, H., Concepcion, G. T., Feng, X., Zhang, H. & Li, H. Haplotype-resolved de novo assembly using phased assembly graphs with hifiasm. *Nat Methods* **18**, 170-175, doi:10.1038/s41592-020-01056-5 (2021).

88 de Manuel, M. *et al.* Chimpanzee genomic diversity reveals ancient admixture with bonobos. *Science* **354**, 477-481, doi:10.1126/science.aag2602 (2016).

89 McLaren, W. *et al.* The Ensembl Variant Effect Predictor. *Genome Biol* **17**, 122, doi:10.1186/s13059-016-0974-4 (2016).

90 Schreiber, J., Bilmes, J. & Noble, W. S. Completing the ENCODE3 compendium yields accurate imputations across a variety of assays and human biosamples. *Genome Biol* **21**, 82, doi:10.1186/s13059-020-01978-5 (2020).

91 Florio, M. *et al.* Human-specific gene ARHGAP11B promotes basal progenitor amplification and neocortex expansion. *Science* **347**, 1465-1470, doi:10.1126/science.aaa1975 (2015).
92 Baker, J. N. *et al.* Evolution of Alu Subfamily Structure in the Saimiri Lineage of New World Monkeys. *Genome Biol Evol* **9**, 2365-2376, doi:10.1093/gbe/evx172 (2017).
93 Steely, C. J. *et al.* Analysis of lineage-specific. *Mob DNA* **9**, 10, doi:10.1186/s13100-018-0115-6 (2018).
94 Yohn, C. T. *et al.* Lineage-specific expansions of retroviral insertions within the genomes of African great apes but not humans and orangutans. *PLoS Biol* **3**, e110, doi:10.1371/journal.pbio.0030110 (2005).
95 Varki, A., Geschwind, D. H. & Eichler, E. E. Explaining human uniqueness: genome interactions with environment, behaviour and culture. *Nature reviews* **9**, 749-763 (2008).
96 Fernandes, J. D. *et al.* The UCSC repeat browser allows discovery and visualization of evolutionary conflict across repeat families. *Mob DNA* **11**, 13, doi:10.1186/s13100-020-00208-w (2020).
97 Jacobs, F. M. *et al.* An evolutionary arms race between KRAB zinc-finger genes ZNF91/93 and SVA/L1 retrotransposons. *Nature* **516**, 242-245, doi:10.1038/nature13760 (2014).
98 Fernandes, J. D. *et al.* KRAB Zinc Finger Proteins coordinate across evolutionary time scales to battle retroelements. *bioRxiv*, 429563, doi:10.1101/429563 (2018).
99 Marchetto, M. C. N. *et al.* Differential L1 regulation in pluripotent stem cells of humans and apes. *Nature* **503**, 525-529, doi:10.1038/nature12686 (2013).
100 Perez-Caballero, D., Soll, S. J. & Bieniasz, P. D. Evidence for restriction of ancient primate gammaretroviruses by APOBEC3 but not TRIM5alpha proteins. *PLoS Pathog* **4**, e1000181, doi:10.1371/journal.ppat.1000181 (2008).
101 Kaiser, S. M., Malik, H. S. & Emerman, M. Restriction of an extinct retrovirus by the human TRIM5alpha antiviral protein. *Science* **316**, 1756-1758, doi:10.1126/science.1140579 (2007).
102 Snyder, M. P. *et al.* Perspectives on ENCODE. *Nature* **583**, 693-698, doi:10.1038/s41586-020-2449-8 (2020).
103 Tajima, F. Statistical method for testing the neutral mutation hypothesis by DNA polymorphism. *Genetics* **123**, 585-595 (1989).
104 DeGiorgio, M., Huber, C. D., Hubisz, M. J., Hellmann, I. & Nielsen, R. SweepFinder2: increased sensitivity, robustness and flexibility. *Bioinformatics* **32**, 1895-1897, doi:10.1093/bioinformatics/btw051 (2016).
105 Nielsen, R. Molecular signatures of natural selection. *Annu Rev Genet* **39**, 197-218, doi:10.1146/annurev.genet.39.073003.112420 (2005).
106 Stagi, M., Fogel, A. I. & Biederer, T. SynCAM 1 participates in axo-dendritic contact assembly and shapes neuronal growth cones. *Proc Natl Acad Sci U S A* **107**, 7568-7573, doi:10.1073/pnas.0911798107 (2010).
107 Nye, J., Mondal, M., Bertranpetit, J. & Laayouni, H. A fully integrated machine learning scan of selection in the chimpanzee genome. *NAR Genom Bioinform* **2**, lqaa061, doi:10.1093/nargab/lqaa061 (2020).
108 Pavlidis, P. & Alachiotis, N. A survey of methods and tools to detect recent and strong positive selection. *J Biol Res (Thessalon)* **24**, 7, doi:10.1186/s40709-017-0064-0 (2017).

109 Emms, D. M. & Kelly, S. OrthoFinder: phylogenetic orthology inference for comparative genomics. *Genome Biol* **20**, 238, doi:10.1186/s13059-019-1832-y (2019).
110 Smith, M. D. *et al.* Less is more: an adaptive branch-site random effects model for efficient detection of episodic diversifying selection. *Mol Biol Evol* **32**, 1342-1353, doi:10.1093/molbev/msv022 (2015).
111 Kuleshov, M. V. *et al.* Enrichr: a comprehensive gene set enrichment analysis web server 2016 update. *Nucleic Acids Res* **44**, W90-97, doi:10.1093/nar/gkw377 (2016).
112 Mi, H. *et al.* PANTHER version 16: a revised family classification, tree-based classification tool, enhancer regions and extensive API. *Nucleic Acids Res* **49**, D394-D403, doi:10.1093/nar/gkaa1106 (2021).
